# A new map of the rat isocortex and proisocortex: cytoarchitecture and M_2_ receptor distribution patterns

**DOI:** 10.1007/s00429-023-02654-7

**Published:** 2023-06-15

**Authors:** Hossein Haghir, Anika Kuckertz, Ling Zhao, Javad Hami, Nicola Palomero-Gallagher

**Affiliations:** 1 Institute of Neuroscience and Medicine (INM-1), Research Centre Jülich, 52425 Jülich, Germany; 2 Department of Anatomy and Cell Biology, School of Medicine, Mashhad University of Medical Sciences, Mashhad, Iran; 3 Medical Genetic Research Center (MGRC), School of Medicine, Mashhad University of Medical Sciences, Mashhad, Iran; 4 Faculty of Medicine, HMU Health and Medical University Potsdam, 14471 Potsdam, Germany; 5 C. & O. Vogt Institute of Brain Research, Heinrich-Heine-University Düsseldorf, 40225 Düsseldorf, Germany

**Keywords:** Brain mapping, Acetylcholine, Receptor architecture, Amygdala, Hippocampus, Basal ganglia, Thalamus, Brainstem

## Abstract

Neurotransmitters and their receptors are key molecules in information transfer between neurons, thus enabling inter-areal communication. Therefore, multimodal atlases integrating the brain’s cyto- and receptor architecture constitute crucial tools to understand the relationship between its structural and functional segregation. Cholinergic muscarinic M_2_ receptors have been shown to be an evolutionarily conserved molecular marker of primary sensory areas in the mammalian brain. To complement existing rodent atlases, we applied a silver cell body staining and quantitative in vitro receptor autoradiographic visualization of M_2_ receptors to alternating sections throughout the entire brain of five adult male Wistar rats (three sectioned coronally, one horizontally, one sagittally). Histological sections and autoradiographs were scanned at a spatial resolution of 1 µm and 20 µm per pixel, respectively, and files were stored as 8 bit images. We used these high-resolution datasets to create an atlas of the entire rat brain, including the olfactory bulb, cerebellum and brainstem. We describe the cyto- and M_2_ receptor architectonic features of 48 distinct iso- and proisocortical areas across the rat forebrain and provide their mean M_2_ receptor density. The ensuing parcellation scheme, which is discussed in the framework of existing comprehensive atlasses, includes the novel subdivision of mediomedial secondary visual area Oc2MM into anterior (Oc2MMa) and posterior (Oc2MMp) parts, and of lateral visual area Oc2L into rostrolateral (Oc2Lr), intermediate dorsolateral (Oc2Lid), intermediate ventrolateral (Oc2Liv) and caudolateral (Oc2Lc) secondary visual areas. The M_2_ receptor densities and the comprehensive map of iso-and proisocortical areas constitute useful tools for future computational and neuroscientific studies.

**Supplementary Information:**

The online version contains supplementary material available at 10.1007/s00429-023-02654-7.

## Introduction

Rodents, in particular rats, are the most widely used animal models in neuroscience research (Keifer and Summers 2016). Thus, the rat cerebral cortex is probably the most extensively studied cortical structure. Due to the structural and functional heterogeneity of the cerebral cortex, it is crucial to have a detailed and reliable map to enable precise localization.

Brodmann (1909) identified two principal subdivisions within the mammalian cerebral cortex based on differences in laminar developmental timelines: the homogenetic cortex, in which areas display the basic six-layered structure both in embryonic and adult stages, and the heterogenetic cortex, with a different layering in early and adult stages. Homogenetic and heterogenetic cortex correspond to the isocortex and allocortex, respectively, as defined by Vogt (1910) based on histological criteria, and are separated by the transitional mesocortex. The proisocortex constitutes the portion of the mesocortex which directly abuts the isocortex, whereas the periallocortex is adjacent to the allocortex (Brodmann 1909; Stephan 1975).

The rat iso- and proisocortex have been parcellated into numerous areas based mostly on cell body stained sections (Palomero-Gallagher and Zilles 2015; Paxinos and Watson 2013; Swanson 2004; Zilles 1985), and the most widely used atlas presents both Nissl- and acetylcholinesterase-stained sections (Paxinos and Watson 2013). The resulting maps, however, are mainly based on the analysis of a single modality, frequently of Nissl-stained sections. Differences in cytoarchitecture are often very difficult to be detected by researchers with less experience in this special anatomical field. This restriction becomes particularly obvious when the available cortical maps are compared. The comparison shows similarities in the parcellation of many brain regions, although often different names were used for the same cortical area (Table 1). However, there are also differences, and the main reason for the discrepancies is not the nomenclature used by different authors, but the different delineation criteria leading to different numbers of cortical areas.

**Table 1:**
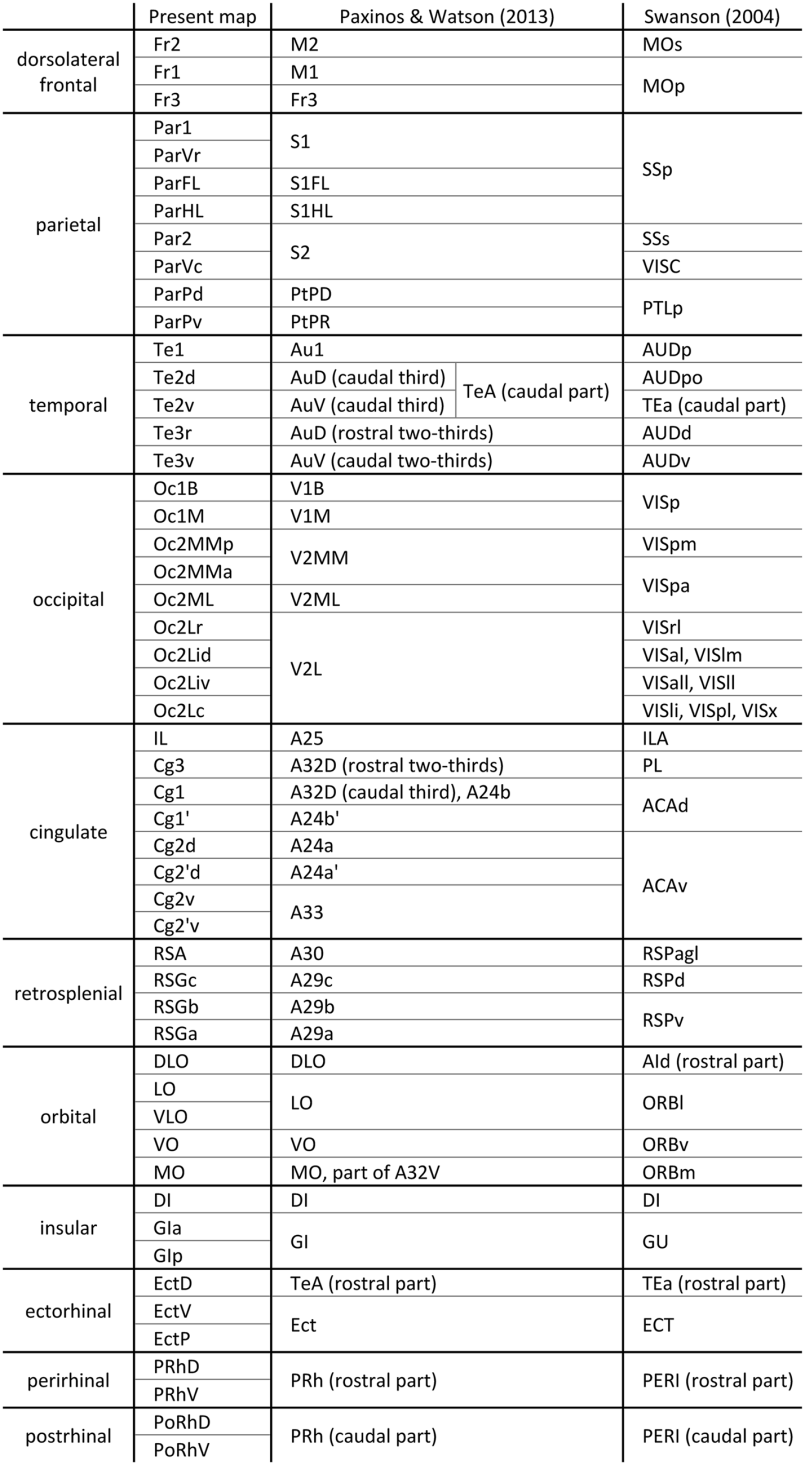
Correspondences between the areas identified in the present study and those shown in the maps of Paxinos and Watson (2013) and of Swanson (2004)

To overcome the difficulties caused by the nearly exclusive cytoarchitectonic maps, the present observation shows the quantitative distribution of a key molecule of signal transmission, i.e. the cholinergic muscarinic binding site of the M_2_ receptor, in serial sections throughout the complete isocortex and proisocortex of the rat. To bridge the gap between previous cytoarchitectonic maps and the current parcellation scheme, the M_2_ receptor distribution is compared with alternating Nissl-stained sections of the same brains. In contrast to all presently available atlases of this part of the rat cortex, the receptor-based parcellation has several advantages:

- It is based on visualization of a functionally relevant molecule of signal transmission, i.e. on the heterogeneous regional and laminar distribution of the M_2_ receptor, which has been proven in previous studies of rodent brains (Palomero-Gallagher and Zilles 2015; Schubert et al. 2016).
- The M_2_ receptor clearly highlights the thalamo-cortical input layers of the primary visual, somatosensory and auditory areas at least in all primate brains (Zilles and Palomero-Gallagher 2017a), and thus may help to identify those areas also in the rat brain, by its exceptionally high density in those areas.
- This receptor is also heterogeneously distributed throughout other isocortical areas, and thus provides delineation criteria depending on its differential quantitative binding of the specific ligand. This enables detailed parcellations and a mapping of all isocortical areas, which exceeds and improves previous attempts while simultaneously providing functionally relevant information.
- Quantitative differences in receptor density enable an objective description of the similarities and differences between the mapped cortical areas.

Aim of the present study was to provide a detailed parcellation of the rat iso- and proisocortex using the heterogeneous distribution of the cholinergic muscarinic M_2_ receptor densities, a comparison with cytoarchitectonic parcellations, and a uniform and easy to understand topographical nomenclatural system, which does not presume functional identifications. The latter aspect does not, however, exclude comparisons with functional data. It simply avoids a premature confusion of anatomically based names of cortical areas with those cortical units which had been identified by other methods.

## Materials and methods

Five adult male Wistar rats (250 g) were decapitated under isoflurane narcosis, the brains were carefully removed from the skull, immediately deep frozen in isopentane at − 50 °C, and placed in pre-cooled plastic bags and stored in a deep freezer at − 80 °C until further processing. All animal procedures and husbandry were approved by the institutional animal welfare committee at the Research Centre Jülich and were carried out in accordance with the guidelines of the European Communities Council Directive for the care and use of animals for scientific purposes.

Each rat brain was serially sectioned at 20 μm thickness using a cryostat microtome (Leica Microsystems, Germany) at − 18 to − 20 °C. Blockface images were obtained for one of the rats (R8) during sectioning (Schubert et al. 2016). Three of the brains were sectioned in the coronal plane (R8, R865, R5), one in the sagittal plane (R874) and another horizontally (R875). Brains to be sectioned in the coronal plane were attached with Tissue Freezing Medium (Leica) to the cryotome chuck by the brainstem and most posterior portion of the cerebellum. The brain to be sectioned in the sagittal plane was attached by the cortex of the right hemisphere containing the posterior portion of Par2 and anterior part of the temporal region, and the brain to be sectioned in the horizontal plane was attached by the brainstem and cortex containing the basal forebrain. Rats R8, R874 and R875 were sectioned in their entirety, yielding a total of 1370, 553 and 312 sections, respectively. The forebrain of R865 was also entirely processed, but sections were only obtained from the rostral portion of the cerebellum, yielding a total of 897 sections. All sections were thaw-mounted onto pre-cooled silanized glass slides. The sections of R8 were organized in series of adjoining triplets of which the first section was used for visualization of cell bodies and cytoarchitecture approach, the second one was processed for quantitative in vitro receptor autoradiography for M_2_ receptors, and the last one was preserved unprocessed. Each of the adjoining series into which sections of R865 were organized encompassed 24 sections, of which the 7th and 23rd were processed for the visualization of M_2_ receptors and of cell bodies, respectively. Sections from R874 and R875 were also organized into groups of 24, but here every and 9th and 21st section were processed for the visualization of M_2_ receptors and of cell bodies, respectively. Thus, there were 40 µm between each section processed for M_2_ receptor visualization in R8, but 480 µm in R865, R874 and R875. For brain R5 we processed groups of sections, each containing 18 sections, obtained from 19 rostro-caudal levels spread throughout the brain. Of these levels, three were taken from the olfactory bulb, 14 from the forebrain and the remaining two from the cerebellum.

The histological sections were stained with a silver staining technique for cytoarchitectonic analysis (Merker 1983), which results in a higher contrast and more intense visualization of cell bodies than the widely used cresyl-violet stainin of Nissl substance. The procedure of quantitative in vitro receptor autoradiography used in this study to demonstrate the densities (in fmol/mg protein) of the agonistic receptor binding sites of the cholinergic muscarinic M_2_ receptor, has been published in detailed previously (Palomero-Gallagher and Zilles 2018). In summary, during a 20 min pre-incubation step at room temperature in 20 mM HEPES-Tris (pH 7.5) containing 10 mM MgCl_2_ and 300 nM pirenzepine, sections were rehydrated and endogenous substances removed. Sections were then incubated 60 min at room temperature in the main incubation buffer with 1.7 nM [^3^H]-oxotremorine-M (PerkinElmer, USA) to visualize the total agonistic binding sites of the M_2_ receptor. Finally, the binding procedure was stopped and surplus tritiated ligand as well as buffer salts were eliminated by a rinsing step (2 × 2 min) in ice-cold buffer followed by a dip in distilled water. Sections were air dried and exposed together with plastic scales of increasing and known radioactivity concentrations against beta-radiation-sensitive films, which were developed after 15 weeks.

Cell body stained sections were scanned using a light microscope (Axioplan 2 imaging, ZEISS, Germany) equipped with a motor-operated stage controlled by the Axiovision (Zeiss, Germany) image analyzing system applying a 6.3 × 1.25 objective (Planapo®, Zeiss, Germany), and a CCD camera (Axiocam MRm, ZEISS, Germany), resulting in images with an in-plane resolution of 1 µm per pixel, and 8-bit grey resolution (Palomero-Gallagher and Zilles 2018). M_2_ receptor autoradiographs were digitized using an image acquisition and processing system Axiovision (Zeiss, Germany) consisting of a source of homogenous light and a high resolution CCD-camera (Axiocam MRm, Zeiss, Germany) with an S-Orthoplanar 60-mm macro lens (Zeiss, Germany) corrected for geometric distortions, and resulting in images with an in-plane resolution of 5 µm per pixel and 8-bit grey resolution. Quantification of receptor densities was performed using the in house software AnaRec (Impieri et al. 2019), which enables computation of the mean grey value of all pixels encompassed by a specific cortical area, and transformation of this value into a receptor concentration per unit protein (fmol/mg protein). For further details see Palomero-Gallagher and Zilles (Palomero-Gallagher and Zilles 2018). For visualization purposes, autoradiographs were subjected to linear contrast enhancement, colour coding and median (Palomero-Gallagher and Zilles 2018).

Receptor densities were extracted separately for the left and right hemisphere. We used a non-parametric permutation test (1000 permutations; (Nichols and Holmes 2002) and an FDR correction (Benjamini and Hochberg 1995) to identify possible significant interhemispheric differences in receptor densities.

## Results

**Fig. 1:**
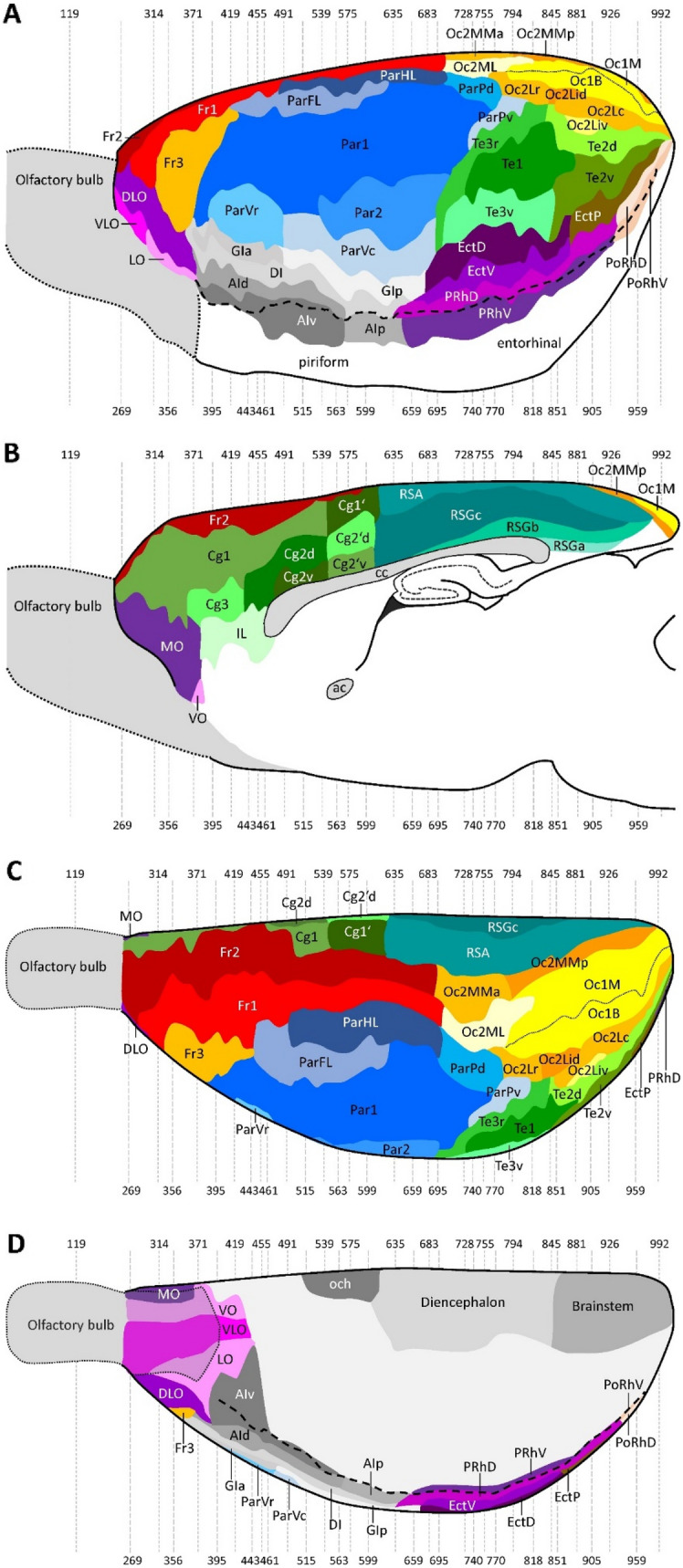
Lateral (**A**), medial (**B**), dorsal (**C**) and ventral (**D**) schematic views of the brain of rat #8 with the colour-coded map of isocortical areas and neighboring mesocortical region (proisocortical and periallocortical areas). The allocortex is not shown. Dashed black line indicates the course of the rhinal fissure. Dotted black line highlights the border between the nasal and temporal portions of the primary visual area (Oc1B and Oc1M, respectively). Dashed grey lines and numbers indicate the positions of sections shown in Fig. 2 and in the Supplementary Material. To avoid cluttering, the numbers have been placed above the lines with short dashes, and below the lines with long dashes. For abbreviations see the Supplementary Material

**Fig. 2:**
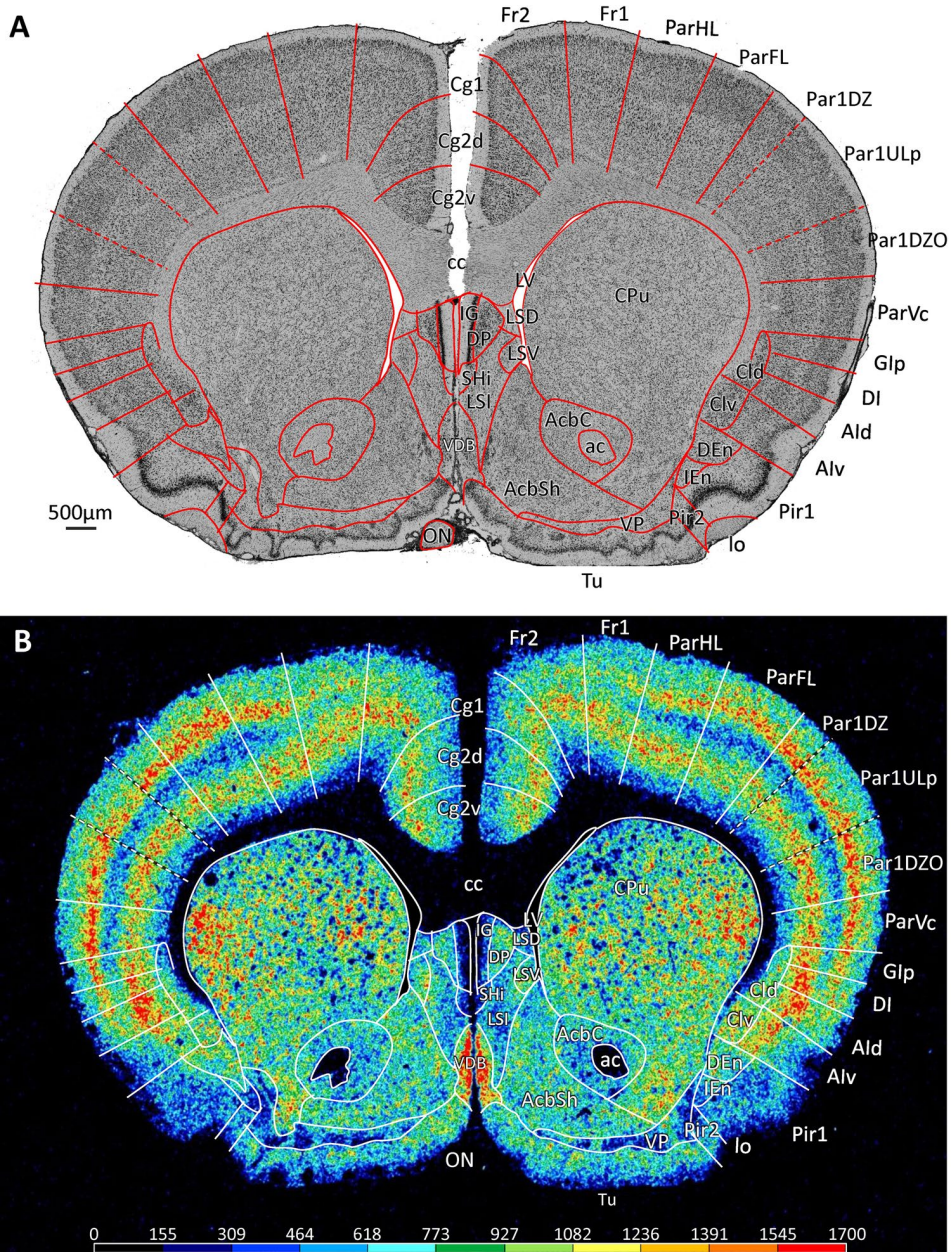
Coronal sections through the brain of rat #8 showing the distribution of (**A**) cell bodies in section #355, and of (**B**) the cholinergic muscarinic M_2_ receptor in section #356. Colour scale indicates receptor binding site concentrations in fmol/mg protein. Dotted lines mark artificial tissue loss. For abbreviations and for all 39 sections through the rat brain, including the olfactory bulb and the cerebellum, see the Supplementary Material

**Fig. 3:**
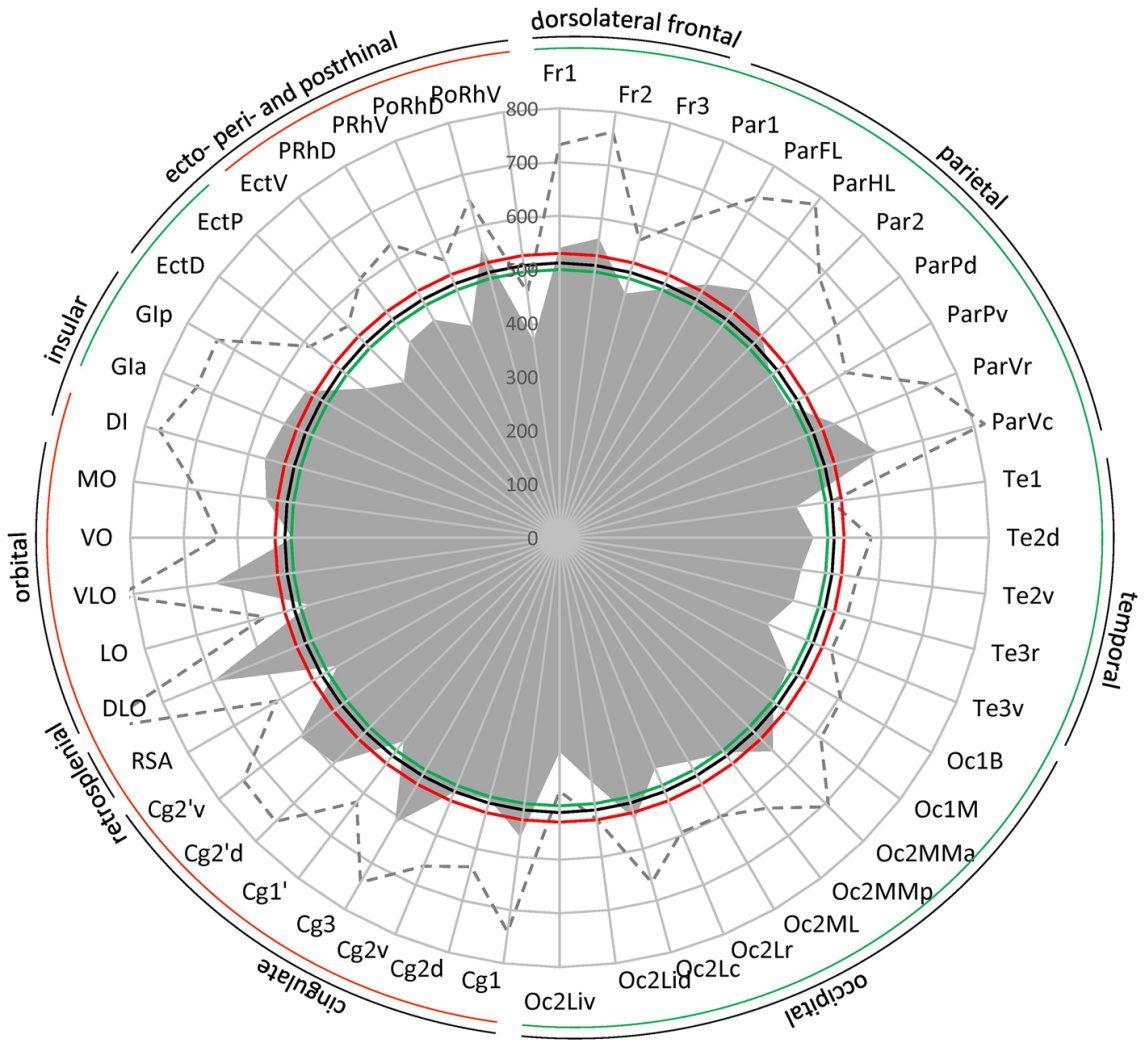
Mean M_2_ receptor density plus standard deviation (dashed line) in fmol/mg protein. Values averaged over all cortical layers and all sections containing a given area are displayed as a polar coordinate plot. The thick black line indicates the average receptor density across all areas, the green and red lines designate the mean receptor density across the isocortical and proisocortical areas, respectively

All topographical relations of the rat brain parcellation are depicted in the map presented in Fig. 1A–D. Figure 2 and levels c1-c39 of the Supplementary Material display overviews of coronal sections through different rostro-caudal levels of the rat brain, and which were processed for the visualization of cell bodies (Fig. 2A; top panel in each level of the Supplementary Material) or of regional and laminar differences in the densities of the M_2_ receptor (Fig. 2B; bottom panel in each level of the Supplementary Material). With a comparable organization, levels s1-s5 and h1-h9 of the Supplementary Material display overviews of sections through different medio-lateral and dorso-ventral levels, respectively, processed for the visualization of M_2_ receptor and cell body distribution patterns. Labelling of the muscarinic cholinergic M_2_ receptors with [^3^H]-oxotremorine-M revealed their heterogeneous distribution throughout the rat brain, although without significant interhemispheric differences (Fig. 3 and Table 2), with highest densities found in the olfactory bulb (Supplementary Material, levels c1-c3, s5, h6-h9) and lowest ones in the cerebellum, including the dentate (DN) and interposed (IP) nuclei (Supplementary Material, level c36, s1-s5, h1-h8). Differences in M_2_ receptor densities not only reveal borders between isocortical areas (the focus of this manuscript), but also enable identification of layers in the superior colliculus (SC; Supplementary Material, levels c25-c30, s4-s5, h2-h4) and nuclei in the amygdala (e.g., higher densities in the anterior basolateral nucleus [BLA] than in the central [Ce] nuclei; Supplementary Material, levels c18-c22), thalamus (e.g., high densities in the anteroventral [AV], rhomboid [Rh], reuniens [Re] and mediodorsal [MD] thalamic nuclei, and low densities in the medial geniculate nucleus [MGN] as well as in the ventral anterolateral- [VAL], posterolateral [VPL] and posteromedial [VPM] nuclei; Supplementary Material, levels c17-c28, s4-s5, h5-h8), basal ganglia (e.g., higher densities in the caudate putamen [CPu] and low densities in the globus pallidus; Supplementary Material, levels c8-c25, s2-s3, h5-h6) or brainstem (e.g., high densities in the pontine gray [PG] hypoglossal nucleus [XII], and low densities in the pontine [PnC] and tegmental reticular nuclei [TRN], as well as in the spinal nucleus of the trigeminal nerve [SpV]; Supplementary Material, levels c30-c39,s5), and of regions within the hippocampal formation (e.g., increasing density gradient when moving from the dentate gyrus through the CA3-CA1 regions to the subicular complex; Supplementary Material, levels c18-c31, s1-s5, h3-h8).

**Table 2:** Mean absolute M_2_ receptor density (in fmol/mg protein; ± standard deviation) separately for the left and right hemispheres, and averaged over both

	Left	Right	p-value	Mean
Fr1	536 ± 196	545 ± 193	0.4625	540 ± 193
Fr2	551 ± 207	574 ± 197	0.3656	562 ± 200
Fr3	466 ± 111	476 ± 99	0.4336	470 ± 103
Par1	492 ± 142	510 ± 145	0.3626	501 ± 142
ParFL	533 ± 207	556 ± 171	0.3866	545 ± 187
ParHL	579 ± 221	582 ± 194	0.5195	580 ± 203
Par2	520 ± 136	544 ± 172	0.3477	532 ± 153
ParPd	502 ± 180	490 ± 145	0.5145	495 ± 154
ParPv	466 ± 111	523 ± 137	0.1728	492 ± 123
ParVr	535 ± 202	576 ± 203	0.3047	556 ± 192
ParVc	608 ± 192	620 ± 231	0.4685	614 ± 205
Te1	445 ± 76	443 ± 57	0.4895	444 ± 66
Te2d	473 ± 115	472 ± 107	0.5035	473 ± 108
Te2v	455 ± 112	452 ± 105	0.5155	454 ± 106
Te3r	437 ± 108	463 ± 101	0.2777	451 ± 103
Te3v	409 ± 150	429 ± 113	0.3936	419 ± 129
Oc1B	479 ± 126	502 ± 107	0.3257	490 ± 115
Oc1M	498 ± 125	506 ± 100	0.4605	502 ± 112
Oc2MMa	559 ± 150	565 ± 167	0.4865	562 ± 147
Oc2MMp	503 ± 136	505 ± 135	0.5864	504 ± 131
Oc2ML	468 ± 97	503 ± 160	0.3327	481 ± 118
Oc2Lr	456 ± 139	474 ± 131	0.4256	464 ± 130
Oc2Lc	558 ± 134	529 ± 119	0.2967	542 ± 122
Oc2Lid	460 ± 82	456 ± 67	0.4785	458 ± 71
Oc2Liv	383 ± 94	420 ± 25	0.3367	398 ± 74
Cg1	565 ± 178	558 ± 192	0.5015	561 ± 180
Cg2d	512 ± 137	497 ± 130	0.4106	504 ± 131
Cg2v	514 ± 153	512 ± 157	0.5145	512 ± 150
Cg3	603 ± 126	621 ± 139	0.3896	612 ± 129
Cg1'	461 ± 150	496 ± 156	0.2977	477 ± 144
Cg2'd	594 ± 166	593 ± 153	0.4545	593 ± 155
Cg2'v	606 ± 154	611 ± 119	0.5195	608 ± 134
RSA	468 ± 133	486 ± 133	0.3347	476 ± 132
DLO	653 ± 251	748 ± 215	0.2218	698 ± 232
LO	472 ± 81	492 ± 99	0.3616	481 ± 87
VLO	652 ± 195	645 ± 170	0.4945	649 ± 179
VO	498 ± 144	498 ± 144	0.5525	498 ± 138
MO	549 ± 143	552 ± 134	0.4955	551 ± 131
DI	558 ± 207	578 ± 207	0.4006	568 ± 204
GIa	541 ± 193	569 ± 171	0.4076	555 ± 177
GIp	553 ± 190	538 ± 203	0.4486	545 ± 192
EctD	450 ± 132	462 ± 132	0.4116	456 ± 130
EctP	405 ± 162	413 ± 148	0.4635	410 ± 148
EctV	451 ± 161	468 ± 141	0.4076	459 ± 150
PRhD	468 ± 167	469 ± 163	0.5045	468 ± 162
PRhV	425 ± 132	429 ± 140	0.4985	427 ± 133
PoRhD	540 ± 71	579 ± 111	0.2348	560 ± 92
PoRhV	380 ± 121	361 ± 85	0.4306	369 ± 93

Nominal p-values resulting from the analysis of left–right differences in receptor densities are provided since there were no significant interhemispheric differences even before correction for multiple comparisons

### Frontal cortex

The frontal cortex comprises the

- Dorsolateral frontal cortex with isocortical areas Fr1, Fr2 and Fr3
- Cingulate cortex with proisocortical areas Cg1, Cg1’, Cg2d, Cg2’d, Cg2v, Cg2’v and Cg3
- Orbital cortex with proisocortical areas DLO, LO, MO, VO and VLO.

#### Topography

Areas Fr1-Fr3 belong to the dorsolateral frontal cortex (Fig. 1A–C) and are characterized by the lack of a prominent layer IV (dysgranular cortex). They are thus clearly delineable from the adjacent parietal areas, which have a distinct and thick layer IV (Fig. 2A; Supplementary Material, levels c2-c20, s3-s4, h1-h6). The Fr-region is found on the dorsolateral and dorsomedial surfaces of the hemisphere and occupies its frontal third. This region is surrounded by the agranular cingulate cortex medially, and the agranular orbital cortex basolaterally (Fig. 1A–C). The occipital cortex follows caudally. Areas Fr1-Fr2 are visible as two longitudinal stripes. Fr2 is the medial and longer strip, which is placed on the dorsomedial surface of the hemisphere and extends from the frontal pole to the occipital cortex. Fr2 is bordered medially by cingulate areas Cg1 and Cg1’. Laterally, Fr2 is bordered mainly by Fr1, except for the most rostral part, where it abuts the dorsolateral orbital cortex (DLO). Fr1 is a long cortical strip located on the dorsolateral surface of the hemisphere, where it adjoins Fr2 rostromedially and extends parallel to Fr2 towards the occipital cortex. Laterally, Fr1 is delimited from anterior to posterior by DLO, Fr3, and parietal areas ParFL and ParHL, respectively. Therefore, Fr3 is the most postero-lateral area of the frontal cortex, and is bordered laterally by DLO and caudally by parietal (Par1 and ParFL) and insular dysgranular (DI) cortices.

The cingulate cortex roughly spans the rostral half of the medial surface of the hemisphere, where it occupies the most dorsal part of the cortex and is delimited caudally by the retrosplenial cortex (Fig. 1B, C). Ventrally, the rostral portion of the cingulate cortex is delimited by orbital area MO and the infralimbic cortex (IL), and the posterior portion by the corpus callosum. The most dorsal portion of the cingulate cortex is occupied rostrally by Cg1 and caudally by Cg1’, and both areas encroach over a short distance on the most medial part of the dorsal hemispheric surface. Cg1 is located ventro-medial to Fr2, and extends rostro-caudally from the rostral pole to the end of the rostral fourth of the corpus callosum, where it is followed caudally by area Cg1’. Cg1 is bordered ventrally (from anterior to posterior) by MO, Cg3 and Cg2d. Cg1’ is delimited dorso-laterally by frontal area Fr2 and ventrally by Cg2’d. The ventral portion of the cingulate cortex is occupied (from rostral to caudal) by areas Cg3, Cg2 and Cg2’, whereby Cg3 does not extend to the rostral pole. Areas Cg2 and Cg2’ can each be subdivided into dorsal and ventral parts (Cg2d/Cg2v and Cg2’d/Cg2’v, respectively). Cg3 is a small area located between Cg1 and IL, and delimited caudally by Cg2d. IL is a periallocortical area and thus not topic of the present study. A very thin indusium griseum, which is not shown on our map, is located below Cg2v and separates it from the corpus callosum.

The orbital cortex occupies the ventral surface of the frontal pole and encroaches onto the ventral part of the medial and lateral surfaces (Fig. 1A, D). From lateral to medial, it consists of 5 areas DLO, LO, VLO, VO and MO. Rostro-caudally, LO extends from the middle of the orbital region toward its caudal end; therefore, in the anterior part of the orbital cortex LO is absent. DLO is found on the ventro-lateral surface of the hemisphere. It is bordered dorsally by frontal areas Fr1-Fr3 and ventrally by VLO in the anterior, and LO in the posterior part of the orbital cortex. MO is located on the medial aspect of the hemisphere below the cingulate cortex and medial to VO. VLO occupies the central position between VO medially and LO laterally.

#### Cytoarchitecture

**Fig. 4:**
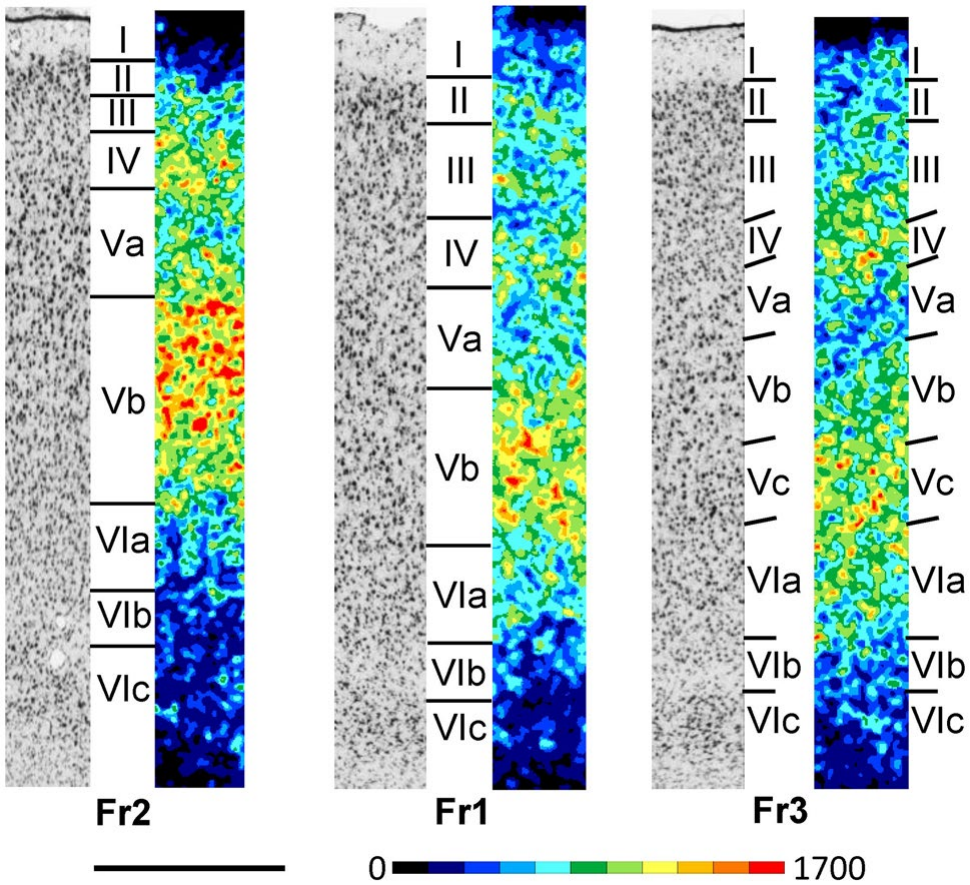
Magnifications depicting the laminar distribution pattern of cell bodies (left side) and of M_2_ receptor binding sites (right side) in areas Fr1, Fr2 and Fr3 of the rat dorsolateral frontal cortex. Roman numerals indicate cortical layers. Scale bar 500 μm

The most conspicuous feature of the dorsolateral frontal cortex is its highly dysgranular cytoarchitecture, i.e. an incipient layer IV is clearly identifiable, and the presence of large and densely packed pyramidal cells in layer V (Figs. 2A and 4; Supplementary Material, levels c2-c20, s3-s4, h1-h6). Layer IV is particularly thin and inconspicuous in areas Fr1 and Fr2 (Fig. 4). The infragranular stratum (layers V-VI) of these areas is consistently broader than the supragranular layers (layers I-III). Layer II has a higher cell packing density in Fr1 and Fr2 compared to Fr3. Layer III can be distinguished from layer II by the lower cell packing density of layer III. Layer V can be subdivided in Fr1 and Fr2 into sublayers Va-Vb by the occurrence of the largest pyramidal cells in layer Va. Fr3 is characterized by a tripartite layer V, with larger pyramids in Va and Vb than in Vc, and a slightly lower cell packing density in Vb than in Va. Layer VI can be subdivided in all Fr areas into sublayers VIa-VIc by the lower packing density of neurons in sublayer VIb.

**Fig. 5:**
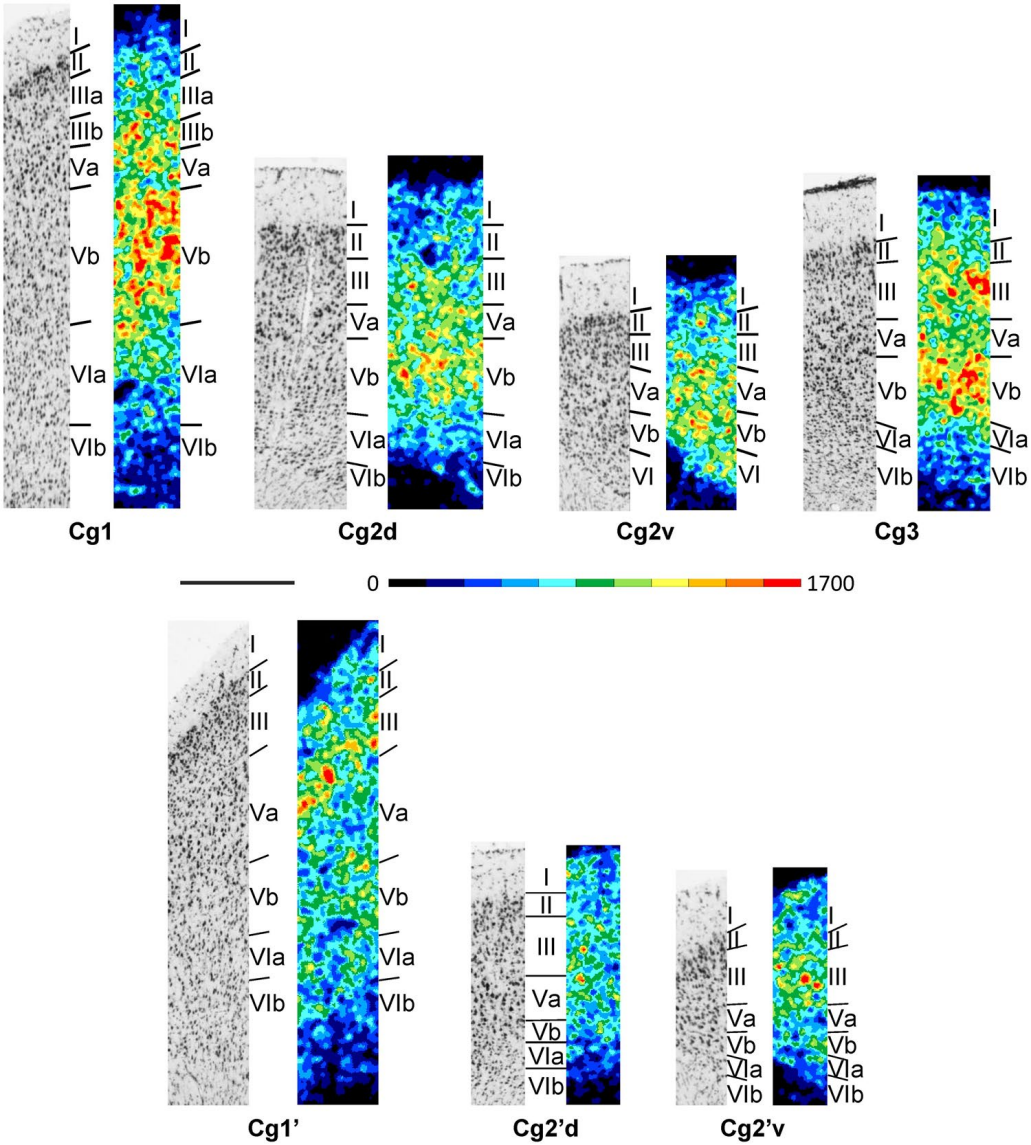
Magnifications depicting the laminar distribution pattern of cell bodies (left side) and of M_2_ receptor binding sites (right side) in areas Cg1, Cg2d, Cg2v, Cg1’, Cg2’d, Cg2’v and Cg3 of the rat cingulate cortex. Roman numerals indicate cortical layers. Scale bar 500 μm

The thickness of the cortical ribbon of the cingulate cortex notably varies between its areas (Figs. 2A and 5; Supplementary Material, levels c4-c16, s4-s5, h1-h5). The broadest cortex is found in Cg1 and Cg1’, the narrowest in Cg2v and Cg2’v. The thickness of Cg3 is comparable to that of Cg2d, and both areas can be distinguished by the overall higher packing density in the former than in the latter area, and by the slightly smaller layer Va pyramids in Cg3 than Cg2d. In all areas of the cingulate cortex layer V comprises larger neurons, but with a lower packing density, than does layer VI. Layer II of Cg1 is narrow and cell dense, and thus can be clearly delineated from layer III. In contrast, in Cg1’ the border between layers II and III is blurred. Layers V and VI of Cg1 and Cg1’ can be subdivided into two sublayers. In both areas layers Va and VIa have larger neurons than Vb and VIb, respectively, though this is more obvious in Cg1 than in Cg1’. Areas Cg2d, Cg2v, Cg2’d, and Cg2’v have a relatively broader layer II than that of Cg1 or Cg1’, and its border to layer III is blurry. Layer VI can be subdivided into a cell sparser VIa and cell denser VIb in Cg2d and Cg2’d, but not in Cg2v or Cg2’v. The most conspicuous differences between anterior (i.e., Cg1, Cg2d, Cg2v) and posterior (i.e., Cg1’, Cg2’d, Cg2’v) subdivisions of the cingulate cortex are found in the densities of their M_2_ receptors, which will be described below.

**Fig. 6:**
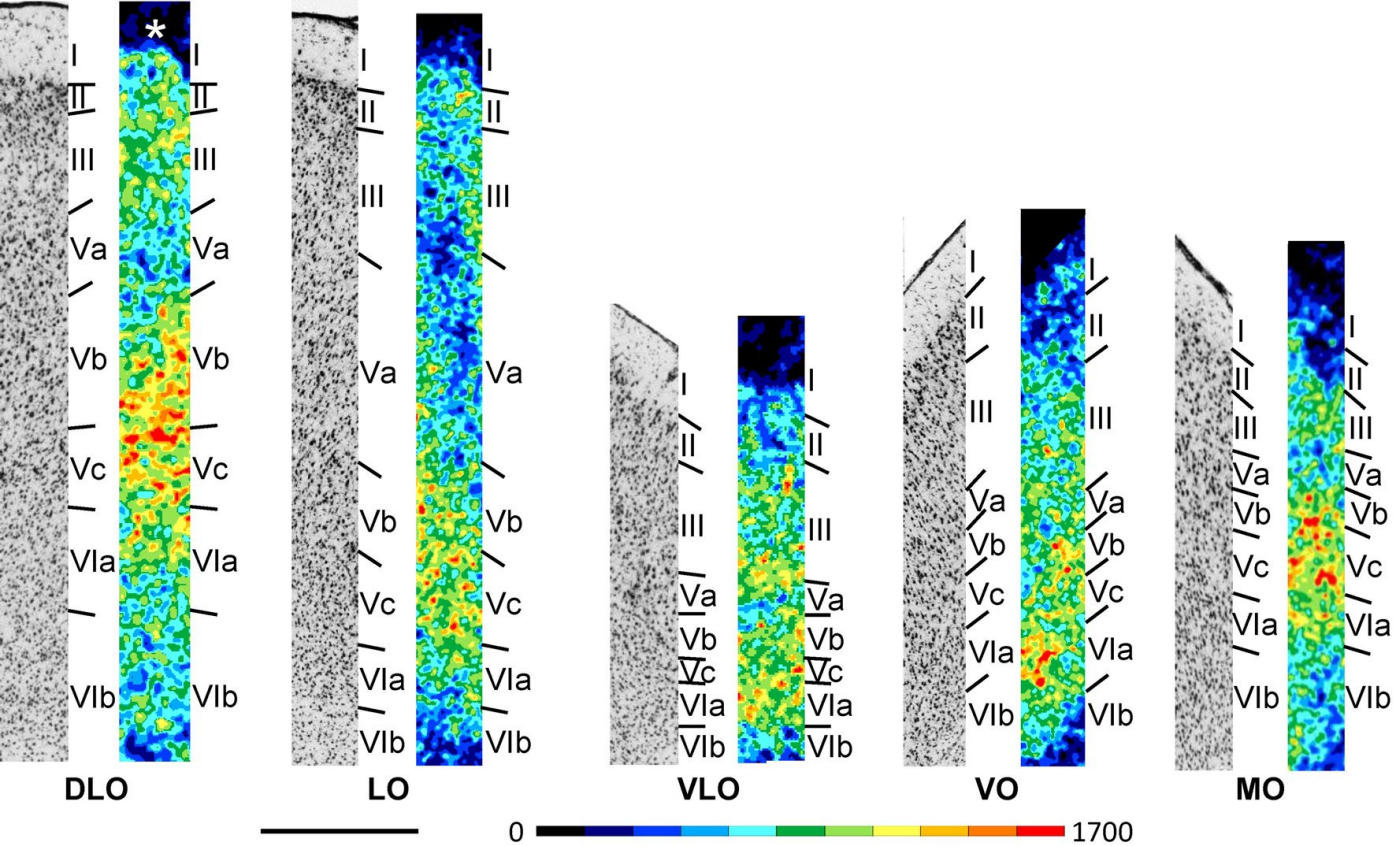
Magnifications depicting the laminar distribution pattern of cell bodies (left side) and of M_2_ receptor binding sites (right side) in areas DLO, LO, VLO, VO, and MO of the rat orbital cortex. Scale bar 500 μm. Roman numerals indicate cortical layers. Asterisk indicates missing tissue in layer I of DLO

In the rat orbital cortex (Figs. 2A and 6; Supplementary Material, levels c2-c7, s4-s5, h5-h7), it is difficult to provide data on absolute cortical thickness and the relative width of the different layers. The real width of the cortex and its layers cannot be measured in the histological sections because the plane of sectioning runs obliquely to the cortical surface due to the bending of this cortical region. Layer II of all orbital areas has a higher cell packing density compared to layer III, this difference in cell density is least prominent in MO and VLO. Sublayer Va contains the largest pyramidal cells of all orbital cortical areas, and sublayer Vb a lower cell packing density than that of Vc. Layer VI has two parts: sublayer VIa with a high cell packing density and sublayer VIb with a low packing density. A clear delineation of the different orbital areas is difficult in cell-body stained sections. Therefore, the presented parcellation of these areas is mainly based on differences in the expression of M_2_ receptors.

#### Receptor architecture

The dorsolateral frontal, cingulate and orbital regions can be easily delineated from each other based on differences in mean and laminar M_2_ receptor densities (Figs. 3, 4, 5 and 6). Area Fr3 presents a lower mean M_2_ receptor density than that of laterally adjacent orbital area DLO, and this difference is particularly obvious in deeper layer V. The M_2_ receptor density in Fr2 is lower than that of medially adjacent cingulate area Cg1, particularly due to the higher supragranular density in Cg1 than in Fr2. Orbital area MO contains a clearly lower M_2_ receptor density than either Cg1 or Cg3.

In the dorsolateral frontal cortex (Figs. 2B and 4; Supplementary Material, levels c2-c20, s3-s4, h1-h6)*,* the mean M_2_ receptor density is higher in Fr2 than in Fr1 or Fr3 (Fig. 3), and this is particularly true for layer Vc (Fig. 4). In all Fr-areas, layer Vb shows the highest, and layers VIb and VIc the lowest M_2_ receptor density. Layer IV is distinguishable in all three areas by its higher density than that of adjacent layers, particularly of Va, though this difference is least prominent in Fr1 and most clearly visible in Fr3. Fr3 differs from Fr1 and Fr2 by the lowest receptor density in the superficial part of layer V. Within frontal areas Fr1 and Fr2, an increasing gradient in the density of M_2_ receptors was observed when moving caudally, that could be explained by motor somatotopic organization.

In the cingulate cortex (Figs. 2B and 5; Supplementary Material, levels c4-c16, s4-s5, h1-h5), the highest and lowest mean density of M_2_ receptors is found in Cg3 and Cg1’, respectively (Fig. 3), and this is particularly obvious in layer Vb (Fig. 5). Whereas in areas Cg1, Cg2d, and Cg2v layer Vb contains a higher M_2_ receptor density than layer III, the opposite holds true for their caudal counterparts Cg’1, Cg2’d, and Cg2’v, respectively. Interestingly, Cg3 has equally high receptor densities in layers III and Vb. The densities of layers I and II of Cg3 are similar. In the infragranular stratum of Cg3, layer V shows a higher density than layer VI in all cingulate areas.

All areas of the orbital cortex (Figs. 2B and 6; Supplementary Material, levels c2-c7, s4-s5, h5-h7) present a lower M_2_ receptor density in layer Va than in layers Vb or Vc, and this difference is particularly obvious in LO. The most lateral of the orbital areas, DLO, contains the highest mean M_2_ receptor density, and medially adjacent area LO the lowest (Fig. 3). VLO differs from all other orbital areas by its layer III receptor density, which is comparable to that found in its layers Vb and Vc. Layer Vc of MO contains a higher M_2_ receptor density than that of laterally adjacent VO.

### Parietal cortex

#### Topography

The parietal isocortex of the rat occupies more than half of the dorsolateral surface of the hemisphere (Fig. 1A–C). It is delimited rostro-medially by the frontal cortex and ventrally by the insular cortex. It has common borders with the temporal cortex postero-laterally, the occipital cortex postero-dorsally, and cingulate area Cg1’ and the retrosplenial agranular cortex medially (Fig. 1A–C). The parietal cortex can be divided into three regions:

- A large central parietal region comprising the highly granular primary somatosensory cortex with areas Par1, ParFL (forelimb area) and ParHL (hindlimb area), and the second somatosensory cortex with area Par2. Par1 is delimited rostrally by Fr3, ventrally (from rostral to caudal) by dysgranular insular area DI, rostrally and caudally by ventral parietal areas (ParVr and ParVc, respectively) and Par2. Dorsally, Par1 shares borders with ParFL and ParHL, whereas caudally it is delimited by posterior parietal areas ParPd and ParPv as well as temporal area Te3r. ParHL occupies a thin strip of cortex on the dorsolateral portion of the hemisphere medial to ParFL and lateral and caudal to Fr1. ParHL is delimited caudally by ParPd. Par2 is surrounded by Par1, ventrocaudal parietal area ParVc and temporal area Te3r.
- A posterior parietal region, ParP, with dorsal and ventral subdivisions (ParPd and ParPv, respectively), which occupies a narrow strip of cortex running in a dorsoventral direction caudal to the primary parietal areas Par1 and ParHL. ParPd is located lateral to medial occipital area Oc2ML and is delimited medio-caudally by lateral occipital area Oc2Lr, and ventrally by Par 1 and ParPv. Oc2Lr is found dorsal to ParPv, which is located rostral and dorsal to temporal area Te3r.
- A ventral parietal region between the central parietal region and the insular cortex comprises rostral area ParVr and caudal area ParVc. ParVr is bordered rostrally and ventrally by Par1 and granular insular area GIa, respectively, and is followed caudally by ParVc, which abuts the ventrally located granular insular area GIp and caudally located areas Te3r and EctD.

#### Cytoarchitecture

**Fig. 7:**
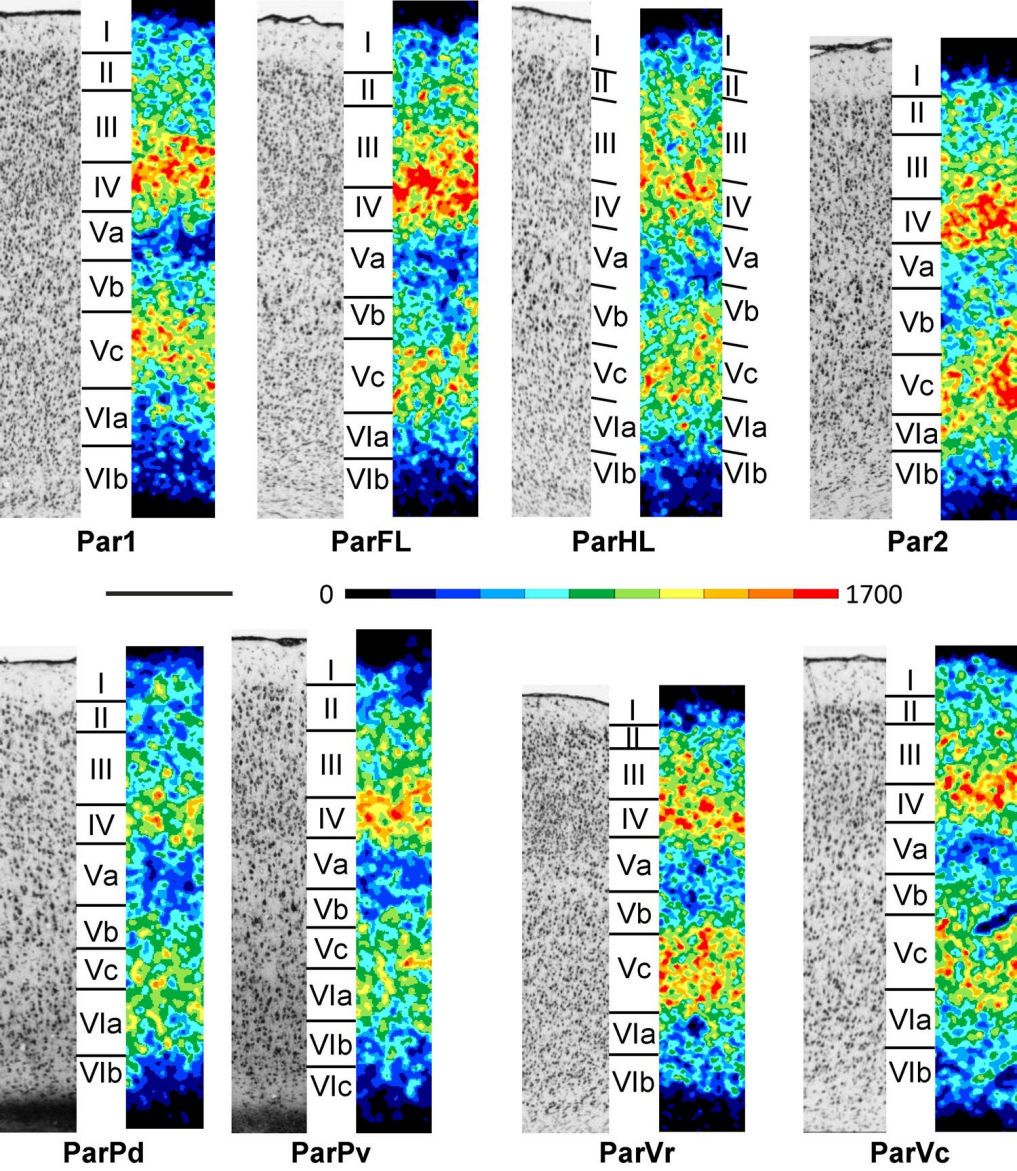
Magnifications depicting the laminar distribution pattern of cell bodies (left side) and M_2_ receptor binding sites (right side) in parietal areas of the rat brain. The magnification for Par1 was taken from the region of the upper lip representation (Par1ULp). Roman numerals indicate cortical layers. Scale bar 500 μm

In general, a well-developed layer IV is the most conspicuous cytoarchitectonic feature of all areas of the isocortical parietal region (Fig. 7). Additionally, layer V is divided into three sublayers: Va shows the lowest and Vc the highest packing density of all three sublayers; Va presents small to medium sized pyramidal cells, Vb contains the largest and Vc the smallest pyramids. The entire layer V is broader and contains more sparsely packed and larger neurons than layer VI. Layer VI is subdivided into a sublayer VIa with more and larger cells than VIb. The thickness of the cortical ribbon of the parietal cortex notably varies only slightly between its areas, whereby ParVr is clearly narrower than the remaining areas (Figs. 2A and 7; Supplementary Material, levels c6-c25, s1-s3, h1-h7).

The areas of the central region show the prototypical cytoarchitecture of the primary sensory cortex in the rat brain, i.e. a wide granular layer IV (Figs. 2 and 7; Supplementary Material, levels c6-c25, s1-s3, h1-h7). This layer can be delineated from layers II-III by its distinctly higher cell packing density. Layer IV also differs from sublayer Va, which appears as a pale stripe with a low packing density of small to medium sized pyramidal cells. The thickness of layer IV changes in the different parts of Par1, and thus may lead to further somatotopically-related subdivisions such as that of the barrel field (Par1BF; Supplementary Material, levels c16-c23). Within Par1BF, layer IV is characterized by a patch-like appearance due to modular differences in the packing density of granule cells (Supplementary Material, levels c16-c23). Area Par2 also has a conspicuous layer IV, though less prominent than in Par1, and with slightly larger cells. Additionally, layers III and V of Par2 are cell sparser than those of Par1 (Fig. 7; Supplementary Material, levels c13-c21). Furthermore, layer V pyramids of Par2 are larger than those of Par1. Layer IV of ParFL and ParHL is less prominent compared to that of Par1, and this is particularly true for ParHL. Layer Vb is thicker in ParHL than ParFL, reaches a slightly higher cell density, as does layer Va, and contains larger pyramids (Fig. 7; Supplementary Material, levels c8-c20).

The posterior parietal region contains areas ParPd and ParPv, which present a narrower supragranular and a broader infragranular layers than the rostrally adjoining parietal areas (Supplementary Material, levels c21-c25, s3, h1-h4). Furthermore, posterior parietal areas are characterized by a low cell packing density in layer II, which is considerably broader in ParPv than in ParPd (Fig. 7). Layer IV is not conspicuous, and this is particularly true for ParPd. Layers Va and Vc are more cell sparse in ParPd than in ParPv. Layer VI presents evenly spaced middle-sized neurons in both ParPd and ParPv.

**Fig. 8:**
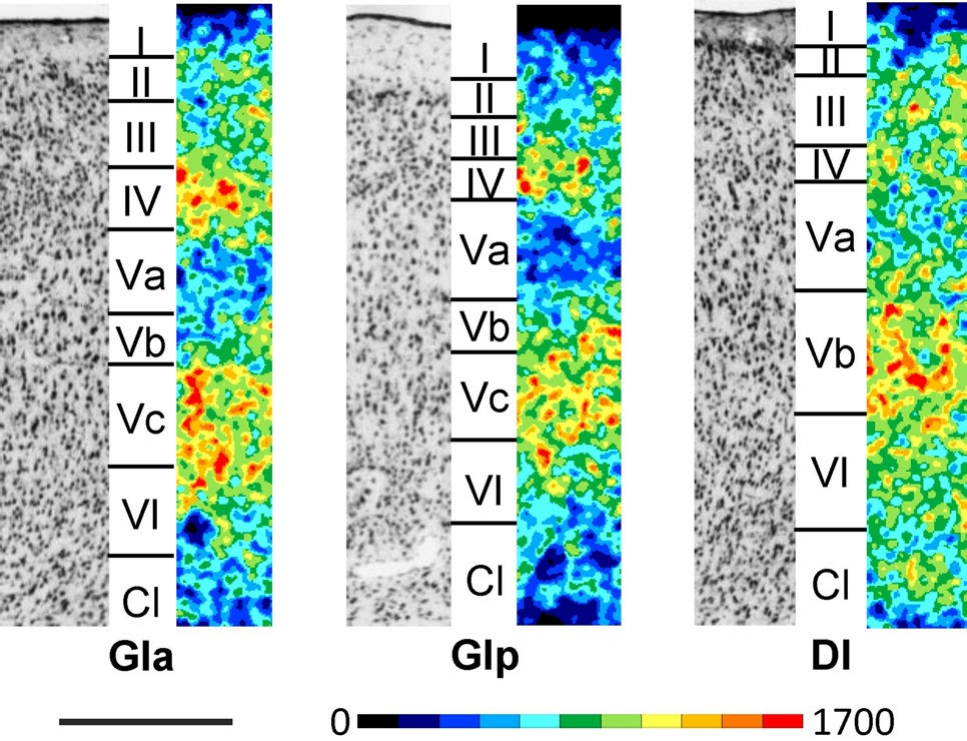
Magnifications depicting the laminar distribution pattern of cell bodies (left side) and M_2_ receptor binding sites (right side) in areas GIa, GIp, DI, AId, AIv and AIp of the rat insular cortex. Roman numerals indicate cortical layers. Scale bar 500 μm

The ventral region consists of areas ParVr and ParVc (Supplementary Material, levels c21-c25, h7). This region is characterized by a relatively thin supragranular layers, which distinguishes it from the central parietal region, particularly from Par2 (Fig. 7). Layer II is more easily separable from layer III in ParVc than ParVr because of the lower cell packing density in layer III of ParVc. ParVr has a higher cell density in layers IV and VI than does ParVc. Layer V of ParVc contains more sparsely distributed and larger neurons than that of ParVr. Layers Va, Vb and Vc are more conspicuous in ParVr than in ParVc. ParVr differs from Par2 by its slightly higher cell density in the supragranular layers. ParVr can be distinguished from the ventrally adjoining insular cortex (area GIa; Fig. 8) by its higher cell density in sublayer Va and smaller cells in sublayers Vb/c. ParVc is difficult to segregate cytoarchitectonically from GIp, but the receptor distribution between both areas is clearly different (see below).

#### Receptor architecture

The parietal region mainly differs from the rostrally adjoining frontal region by its higher density of M_2_ receptors in layer IV (Fig. 2; Supplementary Material, levels c6-c20, s3, h1-h4). Additionally, receptor density is lower in layer Va of the parietal compared with the frontal areas. Layer Va of the parietal cortex is also wide and clearly visible as an obvious stripe of low density (Fig. 7). The temporal cortex has a generally lower receptor density in layer IV than the parietal cortex (Supplementary Material, levels c20-c32, s1-s2, h3-h7). The low-density stripe in layer Va is even more obvious when progressing from the parieto-frontal border to the insular cortex (Fig. 2B; Supplementary Material, levels c6-c18). The occipital cortex shows a lower density in layer IV than the parietal cortex, with the notable exception of the anterior occipital areas (Supplementary Material, levels c20-c25, s3, h1-h3).

All areas of the *central parietal region* show a trilaminar receptor density pattern in layer V, with a very low density in layer Va, a moderate one in layer Vb and a relatively high density in layer Vc (Figs. 2B and 7; Supplementary Material, levels c6-c23, s1-s3, h1-h7). Although somatotopic representations within Par1 are associated with variations of M_2_ receptor densities in layer IV (Supplementary Material, levels c6-c23, s1-s3, h1-h7), in general terms these values are higher than those found in layer IV of the adjacent areas. Sublayer Vb of ParHL has a higher density than that of ParFL. Layer IV of Par2 also presents slight variations in M_2_ receptor densities, though not as conspicuous as those seen in layer IV of Par1.

Areas of the posterior parietal region are characterized, as those of the central and ventral regions, by a broad band with a low M_2_ receptor density which corresponds with cytoarchitectonic layer Va (Fig. 7; Supplementary Material, levels c21-c24,s2-s3, h1-h4). ParPd and ParPv are also characterized by the lowest overall concentrations within the parietal region, and this is particularly true for ParPv (Figs. 3, 7).

Areas ParVr and ParVc of the ventral parietal region are receptor architectonically similar to Par2, but their layer IV receptor density is slightly lower (Fig. 7). Furthermore, the difference in receptor densities between supragranular and infragranular layers is not as large in ParVr and ParVc as in the remaining parietal areas. Mean M_2_ receptor density is higher in ParVc than in ParVr (Fig. 3).

### Insular cortex

The insular cortex, also known as claustrocortex, contains granular to dys- and agranular areas (GI, DI and AI, respectively), thus encompassing isocortical (GI), proisocortical (DI), and periallocortical (AI) areas (Fig. 1A). All insular areas are underlied by the claustrum.

#### Topography

The insular cortex of the lissencephalic rat is located on the ventrolateral surface of the hemisphere, neighbours the anterior part of the rhinal sulcus, and extends from the orbitofrontal cortex rostrally to the perirhinal and ectorhinal cortices caudally (Fig. 1A). It is bounded dorsally mainly by ventral parietal areas ParVr and ParVc. However, at its most rostral portion, it shares a small border with areas Fr3 and Par1. Ventrally, the insular cortex is delimited by the piriform cortex. The insular cortex can be divided dorsoventrally into three longitudinal stripes according to its cytoarchitectonic criteria:

- A dorsal granular strip which can be further subdivided into an anterior area (GIa) located below ParVr and a posterior, slightly larger, area (GIp) found below ParVc. GIp is delimited caudally by the ectorhinal cortex.
- A middle dysgranular strip composed of a single area (DI), which in its most rostral portion wraps around GIa and thus also shares a (very short) common border with Par1.
- A ventral agranular part adjacent to the piriform cortex. The rostral two-thirds of the agranular insular cortex are further subdivided into a dorsal (AId) and a ventral (AIv) agranular cortex, but the posterior third constitutes a single region (AIp). Thus, AId is placed between DI dorsally and AIv ventrally. AIv wraps around the rhinal fissure, including both its dorsal and ventral banks. Areas AId, AIv and AIp belong to the periallocortex and will therefore not be further discussed in this study.

#### Cytoarchitecture

The granular insular cortex (GI) has a well-developed layer IV, which is visible as a dark band of closely packed granular cells which is in sharp contrast to the cell sparse superficial part of layer V directly beneath it (Figs. 2A and 8; Supplementary Material, levels c6-c18, s1-s3, h5-h6). Layers II, III and IV of GI tend to become narrower when they approach the border of the ventrally placed DI. These layers have a higher cell packing density in GIa than GIp, and layer I is broader in GIp than GIa. Furthermore, layer Vb pyramids are smaller in GIp than in GIa. Thus, interface between layers Vb and Vc is easier to identify in the rostral than in the caudal portion of GI.

The cytoarchitecture of DI is comparable to that of AI, but an incipient layer IV can be defined by the presence of some scattered granular cells at the interface between layers III and V, which contain large pyramidal neurons (Fig. 8). The border between layers II and III of DI is blurry, and the very thin and discontinuous layer IV of DI gradually disappears when it reaches the border with the agranular insular cortex. Layer V has two distinct parts, a superficial layer Va with a lower, and a deep layer Vb with a higher cell density.

#### Receptor architecture

Insular areas can be distinguished from the dorsally and ventrally adjacent parietal and piriform cortex, respectively, by their higher M_2_ receptor density in the deeper layers (Figs. 2B; Supplementary Material, levels c6-c18, s1-s3, h5-h6). Interestingly, granular, dysgranular and agranular insular areas differ in their M_2_ laminar distribution patterns (Fig. 8). GIa and GIp present comparably high M_2_ receptor densities in layers IV and Vc, and a conspicuously lower density in layer Va. The mean M_2_ receptor density of GIa is higher than that of GIp. The M_2_ receptor density of layer IV in DI is clearly lower than that of Vb, and only slightly higher than that of Va.

### Temporal cortex

Five isocortical areas have been defined within the temporal cortex: primary auditory area Te1 and secondary auditory areas Te2d, Te2v, Te3r and Te3v.

#### Topography

The temporal cortex occupies the posterior third of the dorsolateral surface of the rat cerebral hemisphere (Fig. 1A and C). It extends between the occipital and ectorhinal cortex and lies caudal to ParVc, and caudo-ventral to Par1, Par2 and ParPv. Area Te1 occupies a central position in the anterior half of the temporal cortex and is completely surrounded by the secondary temporal cortex, which encompasses the subdivisions of areas Te2 and Te3. Area Te3r is found rostral and dorsal to Te1, and is delimited rostrally (from ventral to dorsal) by ParVc, Par2 and Par1, rostro-dorsally by ParPv, and dorsally by occipital area Oc2Lr. Area Te3V is ventral to Te1, dorsal to ectorhinal area EctD, and delimited rostrally and caudally by Te3r and Te2v, respectively. Area Te2d is found dorso-caudal to Te1; it is delimited by Te3r rostrally, and by lateral occipital areas Oc2Lid, Oc2Liv and Oc2Lc dorsally. The most posterior portion of Te2d shares a common border with postrhinal area PoRhD. Area Te2v is delimited rostrally by Te3v and ventrally mainly by ectorhinal area EctP, although most rostrally it shares a border with EctD.

#### Cytoarchitecture

**Fig. 9:**
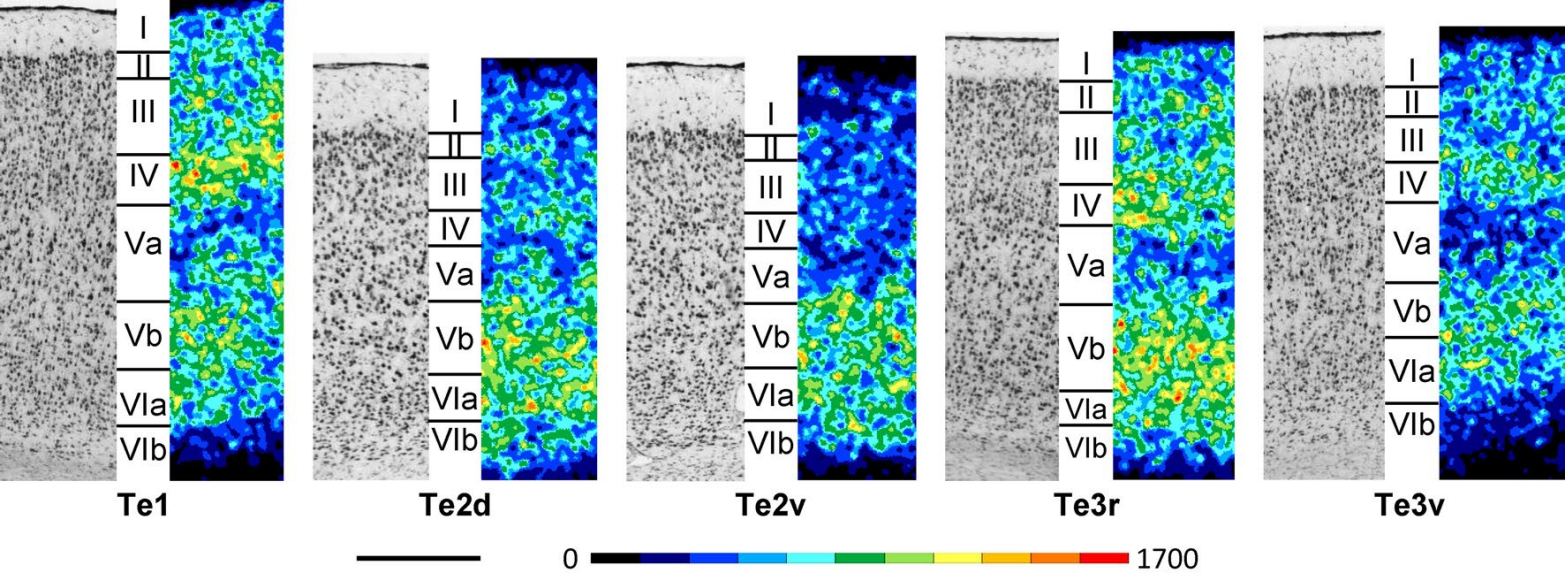
Magnifications depicting the laminar distribution pattern of cell bodies (left side) and M_2_ receptor binding sites (right side) in areas Te1, Te2d, Te2v, Te3r and Te3v of the rat temporal cortex. Roman numerals indicate cortical layers. Scale bar 500 μm

Cortex of the temporal region is characterized in general by narrower supragranular than infragranular layers and the presence of a prominent layer IV (Fig. 9; Supplementary Material, levels c20-c32, s2-s2, h3-h7). Layer V contains large pyramidal neurons and can be subdivided into sublayers Va with a lower, and Vb with a higher cell packing density. Layer VI contains smaller neurons than layer V and can also be subdivided into sublayers VIa with a higher, and VIb with a lower cell packing density. The border between layers II and III is faint and definable based on a difference in neuronal size rather than cell packing density. Layer IV of Te1 is broader and more densely packed than that of Te2 or Te3. Layers III and V of Te1 present a higher and lower cell packing density, respectively, than those of the remaining temporal areas. Layer III pyramids are larger in Te1 than in Te2. Layer V pyramids are larger in Te2d than in Te2v, but smaller than those of Te3r or Te3v. The latter areas differ by their layer Vb, which is more densely packed in Te3r, as well as their layers IV and Va, which are more densely packed in Te3v.

#### Receptor architecture

In general, the density of M_2_ receptors decreases in both a dorsoventral and a rostro-caudal direction in the temporal cortex (Fig. 9; Supplementary Material, levels c22-c32, s2-s2, h3-h7), so that Te2d contains the highest and Te3v the lowest mean density values (Fig. 3). Layer IV of Te1 and Te3r presents a conspicuously higher M_2_ receptor density than that of areas Te2d, Te2v or Te3v. The deeper layers of Te3r present a higher M_2_ receptor density than those of Te1, but the superficial layers of Te1 contain more M_2_ receptors than those of Te3r or Te3v. Te3r differs from caudally adjacent Te2d by the lower M_2_ receptor density in the latter region, particularly in its supragranular layers. Whereas the superficial and deep layers of Te3v contain comparable M_2_ receptor densities, in caudally adjacent Te2v the deeper layers present a higher concentration of M_2_ receptors.

### Occipital cortex

#### Topography

The occipital isocortex is located on the caudal third of the dorsal and dorsolateral surfaces of the hemisphere, and close to the occipital pole also encroaches onto its medial surface (Fig. 1A–C). It can be divided into a primary occipital cortex (Oc1), which is surrounded by the secondary occipital cortex (Oc2). Oc1 encompasses the monocular primary visual area (Oc1M) and laterally adjacent binocular primary occipital area (Oc1B). Oc2 can be divided into *three medial* (Oc2ML mediolateral secondary occipital cortex, and Oc2MM mediomedial secondary occipital cortex with anterior and posterior portions [Oc2MMa and Oc2MMp, respectively]) and four lateral areas (Oc2Lr rostrolateral secondary occipital cortex, Oc2Lc caudolateral secondary occipital cortex, Oc2Lid intermediate dorsolateral secondary occipital cortex, and Oc2Liv intermediate ventrolateral secondary occipital cortex).

Medial to the rostral tip of Oc1 lies area Oc2ML, which is delimited rostrally by frontal area Fr1, medially by areas Oc2MMa and Oc2MMp, and laterally (from rostral to caudal) by areas ParPd, Oc2Lr and Oc1B. Oc2MMa is delimited rostrally by Fr1 and Fr2, medially by the agranular retrosplenial cortex (RSA) and caudally by Oc2MMp, which occupies a long strip of occipital cortex extending between RSA and Oc1M.

Occipital cortex lateral to Oc1B is occupied mainly by areas Oc2Lr and Oc2Lc, which are separated from each other by the small wedge-shaped area Oc2Lid. Thus, Oc2Lid is delimited medially by Oc1B, rostrally by Oc2Lr, laterally by Te2d, and latero-caudally by Oc2Lc. A further small occipital area, Oc2Liv, is intercalated between Oc2Lc and temporal area Te2d. Area Oc2Lr abuts ParPd rostrally, and shares common borders medially with Oc2ML and Oc1B, and laterally with areas ParPd, ParPv and Te3r. The most rostral portion of Oc2Lc is delimited medially by Oc2Lid, and laterally (from rostral to caudal) by Te2d and Oc2Liv. The caudal half of Oc2Lc is located between Oc1B and Te2d.

#### Cytoarchitecture

**Fig. 10:**
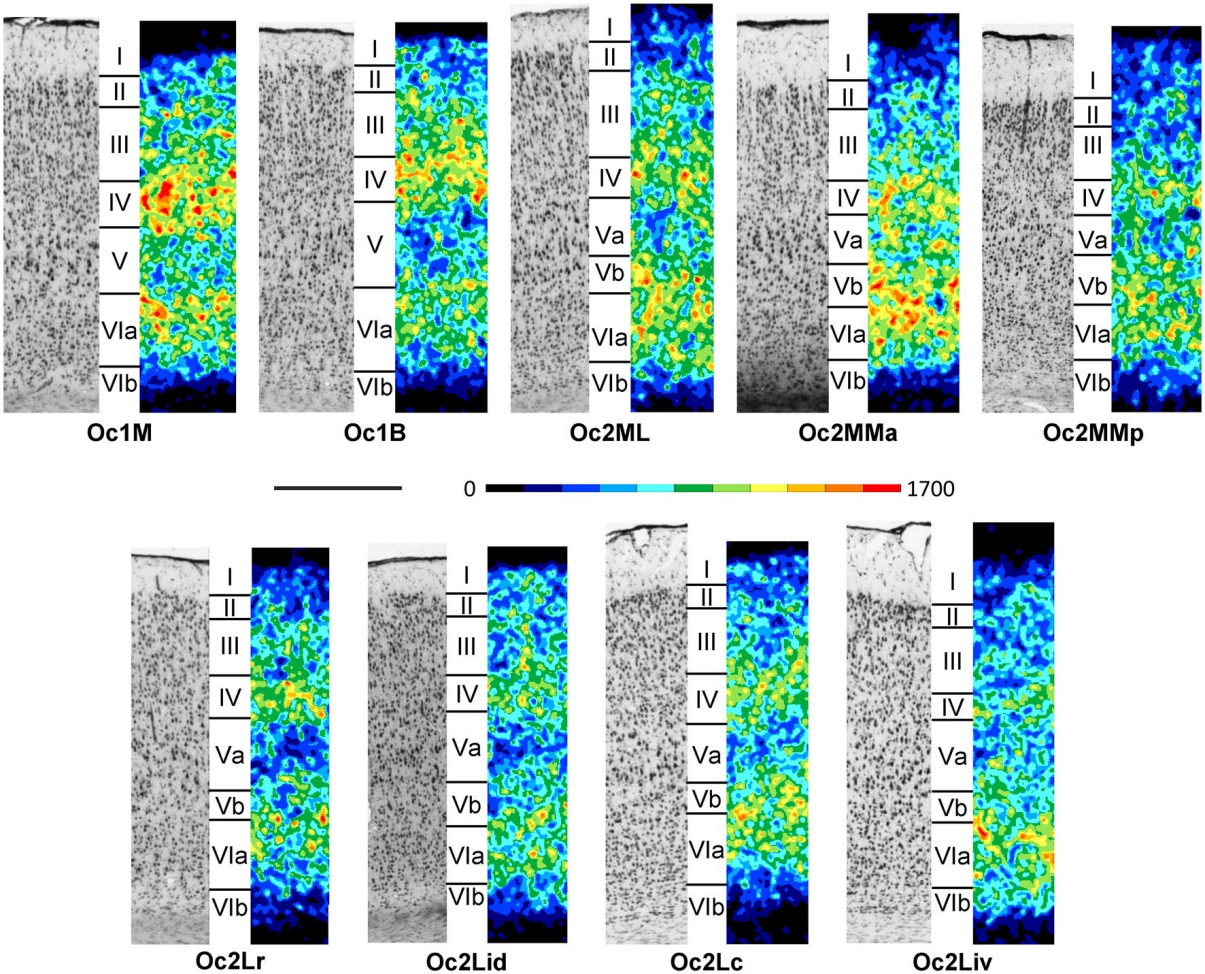
Magnifications depicting the laminar distribution pattern of cell bodies (left side) and M_2_ receptor binding sites (right side) in areas Oc1M, Oc1B, Oc2ML, Oc2MMa, Oc2MMp, Oc2Lr, Oc2Lid, Oc2Lc and Oc2Liv of the rat occipital cortex. Roman numerals indicate cortical layers. Scale bar 500 μm

Areas of the occipital cortex are characterized in general by one of the broadest layer IV of the rat brain. Within the occipital cortex, Oc1 has the most conspicuous inner granular layer (Fig. 10; Supplementary Material, levels c20-c33, s3, h1-h3). The packing density of granule cells in layer IV of Oc1M is a little higher than that in Oc1B, and cell bodies tend to be generally larger in Oc1M than in Oc1B. Layers II and III are not easily distinguishable from each other in either Oc1 subdivision, but layers II/III are thinner in Oc1B than in Oc1M. Layer V of Oc1M has a lower cell packing density than that of Oc1B.

Areas Oc2ML and Oc2MM of the medial secondary occipital cortex (Fig. 1) have a narrower and less cell dense layer IV than that of Oc1 areas, as well as a sublaminated layer V, with larger pyramids in Va than in Vb (Fig. 10). Granule cells are larger and more densely packed in layer IV of Oc2ML than in those of Oc2MMa or Oc2MMp, and the opposite holds true for layer V pyramids. Layer II is slightly denser than layer III in the three areas, and this difference is more obvious in Oc2ML. The main differences between Oc2MMa and Oc2MMp are the higher cell packing density in layer II of Oc2MMp and the larger layer V pyramids in Oc2MMa.

Areas of the lateral secondary occipital cortex are characterized by relatively small layer III pyramids, resulting in a blurred border with layer IV, and the presence of a layer Va with larger pyramids than those in Vb (Fig. 10). Layer VI can be also subdivided into a sublayer VIa with higher and a sublayer VIb with lower cell packing density and smaller neurons. Layer IV is broadest in Oc2Lc and narrowest in Oc2Liv. Oc2Lc also presents the largest layer Va pyramids of all lateral secondary occipital areas. The overall cell packing density in Oc2Lr is lower than that of Oc2Lid, though this feature is most obvious in Va. In contrast to Oc2Lc, Oc2Liv has a fairly well-defined layer II, which can be distinguished from layer III due to the lower cell packing density in the latter layer.

#### Receptor architecture

All occipital areas show a bilaminar distribution of the M_2_ receptor with one maximum in layer IV and a second one in layer VIa or in layers Vb and VIa (Fig. 10; Supplementary Material, levels c20-c33, s3, h1-h3). Throughout the secondary occipital cortex layer Va presents a lower M_2_ receptor density than does Vb. Interestingly, M_2_ densities are higher in layer Vb of medial than of lateral Oc2 areas. Medial occipital areas are generally characterized by a higher mean M_2_ receptor density than that of lateral occipital areas (Fig. 3). Layers IV, V and VIa of Oc1M present a higher M_2_ receptor density than those of Oc1B. In Oc2ML the two M_2_ receptor maxima reach comparable values, whereas in Oc2MMa and Oc2MMp layers Vb and VIa present a higher density than does layer IV. Furthermore, M_2_ receptor densities are lower in layer Va of Oc2ML than in those of Oc2MM subdivisions. Oc2Mma and Oc2MMp can be identified by the higher density of M_2_ receptors in the former than in the latter area. When moving from Oc2Lr over Oc2Lid to Oc2Lc, M_2_ receptor densities show an increasing gradient in layer VIa (Fig. 10). Layers III and IV of Oc2Liv present a lower M_2_ receptor density than those of Oc2Lc.

### Retrosplenial cortex

The retrosplenial cortex constitutes a mesocortical brain region which in the rat encompasses the so-called agranular (RSA) and granular (RSG) retrosplenial areas. RSA is classified as proisocortex, whereas RSG is a periallocortical area, and is thus not subject of the present analysis.

#### Topography

RSA lies on the dorsomedial surface of the rat hemisphere and is delimited medially by RSG, rostrally by cingulate area Cg1’ and laterally (from rostral to caudal) by Fr2 and Oc2MM (Fig. 1B, C). Close to the occipital pole RSA is replaced caudally by area Oc2MM.

#### Cytoarchitecture

**Fig. 11:**
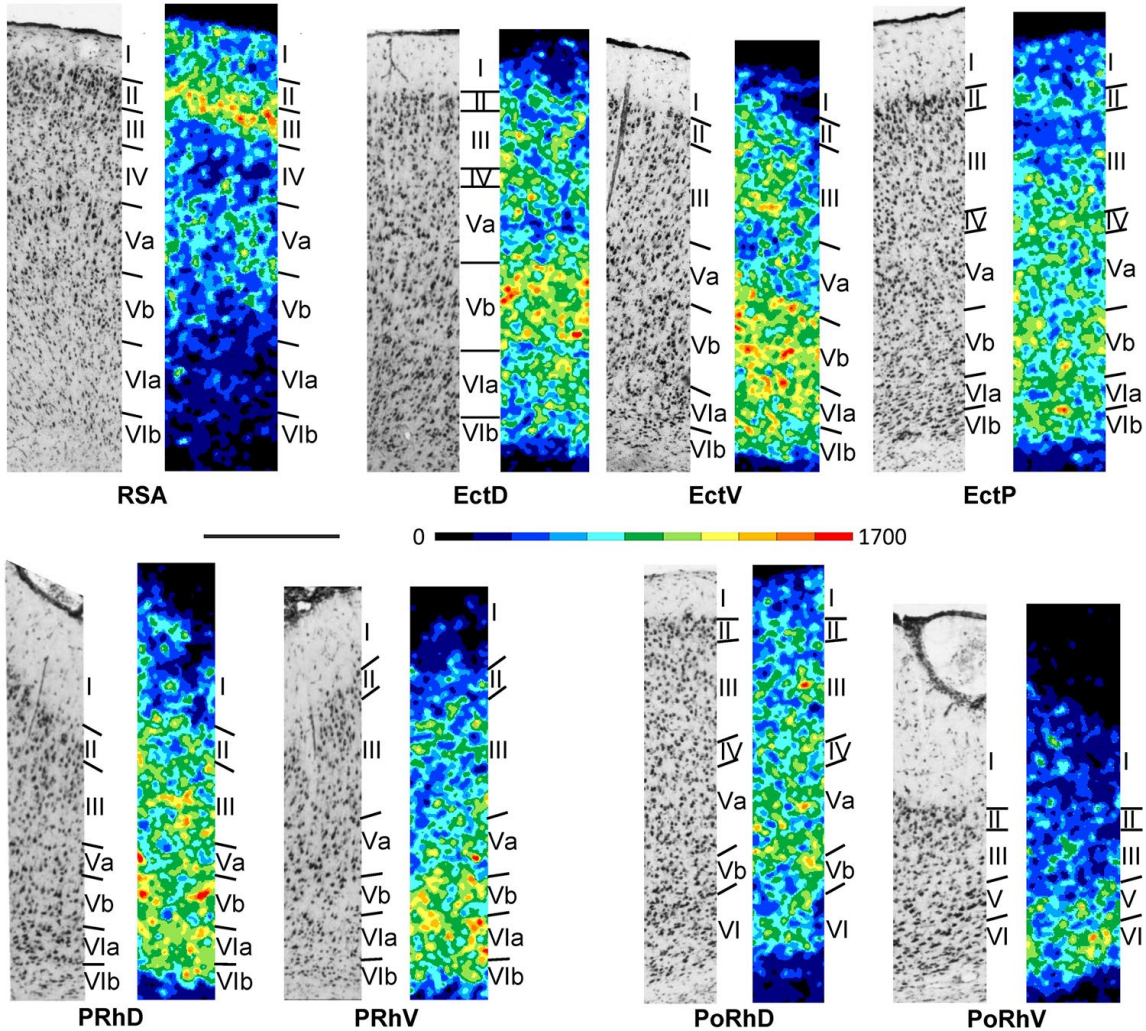
Magnifications depicting the laminar distribution pattern of cell bodies (left side) and M_2_ receptor binding sites (right side) in the agranular retrosplenial area (RSA), in the dorsal (EctD), ventral (EctV) and posterior (EctP) subdivisions of the ectosplenial cortex, as well as in the dorsal and ventral subdivisions of the perirhinal (PRhD, PRhV, respectively) and postrhinal (PoRhD, PoRhV, respectively) cortex. Roman numerals indicate cortical layers. Scale bar 500 μm

Layer II of RSA is broad and its border with layer III is blurred. Layer IV is dysgranular, with loosely packed medium-sized granular shaped pyramids (Fig. 11; Supplementary Material, levels c17-c32, s3-s5-, h1-h4). Layer V is subdivided into sublayers Va and Vb clearly identifiable by the much larger pyramids in the former than in the latter sublayer. Layer VI of RSA is also sublaminated, whereby VIb contains larger but less densely packed neurons than does VIa.

#### Receptor architecture

Layer III is characterized by a conspicuously higher M_2_ receptor density than any other layer of RSA, thus enabling its clear delineation from layer II (Fig. 11; Supplementary Material, levels c17-c32, s3-s5-, h1-h4). Layers IV-VI present only moderate-to-low M_2_ receptor densities, which do not emphasize the sublamination of layers V or VI.

### Ecto-, peri-, and postrhinal cortex

#### Topography

This region of the rat brain comprises ventrolateral cortex along the posterior half of the rhinal sulcus. It extends in the rostro-caudal direction from the insular cortex to the occipital pole. The rhinal region is bordered dorsally mainly by areas Te3v and Te2v (rostrally and caudally, respectively), except for its most rostral part, which is adjoined by Te3r and ParVc. Ventrally, the rhinal cortex is neighboured from anterior to posterior by the piriform and entorhinal cortices, respectively (Fig. 1A, D). The rhinal cortex can be divided into two longitudinal stripes:

- The dorsal longitudinal stripe extends from the insular cortex to the occipital pole and contains the ectorhinal cortex (Ect), which encompasses isocortical and proisocortical areas.
- The ventral longitudinal stripe wraps around the rhinal fissure, including both its dorsal and ventral banks and is occupied by proisocortical areas. The anterior two-thirds of this stripe constitute the perirhinal cortex (PRh). Its posterior third is occupied by the postrhinal cortex (PoRh).

The anterior half of the length of the ectorhinal cortex (Ect) is further subdivided into a dorsal (EctD) and a ventral ectorhinal area (EctV). Its posterior half is formed by a single posterior ectorhinal area (EctP). Dorsally EctD is mainly delimited by Te3v, though its most rostral portion borders with Te3r and ParVc, and its most caudal portion abuts Te2v. The ventral ectorhinal cortex (EctV) lies between EctD and PRh. PRh can be further subdivided into a dorsal part (PRhD), which lies ventral to EctV and EctP, and a ventral part (PRhV), which is located dorsal to the piriform (part of the allocortex) and entorhinal (part of the periallocortex) cortex. *PoRh* can also be further subdivided into a dorsal part (PoRhD), which lies mainly ventral to EctP except for its most caudal part, which neighbours Te2v and then Te2d, and a ventral part (PoRhV), which is located dorsal to the entorhinal cortex. The postrhinal cortex rises steeply and wraps obliquely around the caudal pole of the hemisphere. Thus, its detailed analysis is difficult to perform in coronal sections because most of it is obliquely arranged and thus, tangentially sectioned.

#### Cytoarchitecture

Layer IV in the ectorhinal cortex is generally inconspicuous and fades away when moving ventrally (Fig. 11; Supplementary Material, levels c19-c32, s1-s2, h4-h7). Thus, EctD and EctP can be classified as a dysgranular isocortical areas, whereas EctV is agranular and part of the proisocortex. EctD has a broader but less densely packed layer II and a slightly more prominent layer IV than does EctP. Layer III of EctP is broader than that of EctD and characterized by a slight increasing gradient in the packing density of its pyramids. Pyramids in layer Vb of EctD present a slight columnar organization, which is not visible in the corresponding layers of EctV or EctP.

The perirhinal cortex is agranular and differs from the ectorhinal cortex mainly by its thinner layer V and broader layer I (Fig. 11). Additionally, layers II and III are distinctly separated in the ectorhinal cortex, whereas in the perirhinal cortex the border between them is blurred. PRhD differs from PRhV by its patchy layer II (its cells form clumps), lower packing density of neurons in the deep part of layer III (which gives the appearance of a cell-sparse gap between layers III and Va) and smaller pyramidal neurons in layers Va, Vb and VIa.

The postrhinal cortex contains a dorsal dysgranular area PoRhD and a ventral agranular area PoRhV. Both areas differ from the perirhinal cortex by their thicker layer II, higher packing density in layers V and VI, and smaller pyramidal cells in layer V (Fig. 11).

#### Receptor architecture

The ecto-, peri and postrhinal cortex can also be divided into distinct areas when analysing the density and laminar distribution pattern of M_2_ receptors (Fig. 11; Supplementary Material, levels c19-c32, s1-s2, h4-h8). Within this brain region, area PoRhD presents the highest, and PoRhV the lowest mean M_2_ receptor density (Fig. 3). In EctD and EctV, sublayer Va appears as a distinct low-density band in M_2_ receptor autoradiographs; layers II-III (and IV when present) above this low-density band show a moderate and homogenous receptor density, which cannot be subdivided into distinct layers. In both areas sublayer Vb shows a high receptor density. The most prominent difference between EctD and EctV is found in layer VIa, which presents a higher receptor density in the latter than in the former area. Furthermore, in EctV sublayer VIa shows a higher receptor density than sublayer VIb, whereas EctV presents a homogenous receptor density throughout layer VI. EctP contains a lower receptor density, particularly in layer Vb, compared to EctD and EctV.

EctD and EctP differ from Te2v by its lower M_2_ receptor density in the infraganular layers. ParVc and GIp can be distinguished from EctD by their very prominent and high receptor density in layer IV. DI in comparison with EctV shows a higher receptor density in layer Va.

The perirhinal cortex differs from EctV by its laminar distribution of M_2_ receptors, particularly in its lower density in layers Va and VIb. The most obvious difference between PRhD and PRhV is the lower receptor density in layer III of PRhV than PRhD.

The postrhinal cortex differs from the perirhinal cortex by an overall lower receptor density, which is particularly obvious in layers V-VI. PoRhD has a higher receptor density in layers I-V, but a lower density in layer VI compared to PoRhV.

## Discussion

The expression of transmitter receptors is a powerful tool for mapping the cerebral cortex in the human brain (for a comprehensive review see (Palomero-Gallagher et al. 2015). The pattern of the regionally heterogenous receptor distribution frequently matches cytoarchitectonic subdivisions, or can lead to even finer parcellations. Particularly, the cholinergic muscarinic M_2_ receptor is an excellent indicator of cortical segregation, since it not only shows borders of cortical areas at exactly the same places as cytoarchitectonic analysis, but can additionally distinguish between cortical types (e.g. isocortex vs. allocortex and sensory vs. motor vs. association cortex), indicate the hierarchical position of an area within a specific functional system, and provide valuable information on the evolutionary constancy of cholinergic neurotransmission in primary sensory areas (Palomero-Gallagher and Zilles 2019; Zilles and Palomero-Gallagher 2017a, b). Since the muscarinic M_2_ receptor is an important molecule for improving the signal-to-noise ratio in cortical areas (Lucas-Meunier et al. 2003; Sillito and Kemp 1983), this receptor is also an important modulator of brain functions. The relationship between the distribution of multiple neurotransmitter receptors, including that of the M_2_ receptor, and the cyto-and myeloarchitectonic segregation throughout the rat isocortex has been subject of previous studies (Palomero-Gallagher and Zilles 2004, 2015). However, these studies were based on a sparser covering of the isocortex than that chosen for the present study and did not comprehensively cover the proisocortex. Thus, our knowledge of the cortical distribution of the M_2_ receptor in the rat brain remains incomplete. The present study maps for the first time the regional and laminar distributions of this receptor in serial sections through the rat entire isocortex and its neighbouring proisocortical areas. It also serves as a validation of cytoarchitectonic mapping.

For the areas described in this study, we have coined a relatively simple nomenclature which builds on topographical relationships as initially proposed by Zilles (1985) and expanded in Palomero-Gallagher and Zilles (Palomero-Gallagher and Zilles 2004, 2015), which strictly avoids any functional implications. In the past, various nomenclatures have been used for the cortical areas of the rat (Palomero-Gallagher and Zilles 2015; Paxinos and Watson 1986, 2013; Swanson 2004; Zilles 1985). Different names for the same cortical area were applied, which emphasized either its histological, topographical or functional aspect. This can lead to confusions and misinterpretations because the location of the functional data is frequently not mapped to architectonical findings in the same brain. Since the present observation is solely based on structural (cyto- and chemoarchitectonic) data, we apply a nomenclatural system which is based on the simple topographical division of the rat cerebral cortex in frontal, orbital, insular, cingulate, parietal, temporal, rhinal and occipital regions without inferring functional implications by the name of the areas. This does not mean, however, that the areas identified in the present study cannot be tentatively interpreted by comparing the structural with functional data (see below).

### Frontal cortex

Dorsolateral frontal areas Fr1, Fr2 and Fr3 of the present study were found to be dysgranular, i.e. an incipient inner granular layer (layer IV) is clearly identifiable, and is most prominent in Fr3. Therefore, areas Fr1-Fr3 do not match the typical agranular appearance of motor cortical areas in primates. Interestingly, granularity of the rodent motor cortex has been subject of debate, as has that of the primate motor cortex (Garcia-Cabezas and Barbas 2014). Although some authors consider the rodent motor cortex to lack a layer IV (Beaulieu 1993; Donoghue and Parham 1983; Donoghue and Wise 1982; Swanson 2004), our identification of an incipient layer IV in areas Fr1-Fr3 provides further support for the classification of this brain region as being dysgranular in nature (Cho et al. 2004; Krieg 1946a; Palomero-Gallagher and Zilles 2015; Skoglund et al. 1997; Yamawaki et al. 2014; Zilles and Wree 1995).

Lateral frontal areas Fr1 and Fr3 and medial frontal area Fr2 are comparable to the lateral precentral (PrCl) and medial precentral (PrCm) areas of Krettek and Price (1977), respectively, by location and extent, and were identified as motor areas. Interestingly, Swanson (2004) cklassifies this cortex as somatomotoric in nature, as PrCl and PrCm correspond to his primary somatomotor (MOp) and secondary somatomotor (MOs) areas, respectively. Donoghue and Wise (1982) named these two parts of frontal cortex lateral agranular (AGl) area and medial agranular (AGm) area according to their architectonic appearance as agranular cortex, whereas Paxinos and Watson (2013) identified three different architectonical fields in the frontal cortex of the rat and used a mixture of functional (primary motor, M1 and secondary motor, M2) and topographical (Fr3) nomenclatural terms (Table 1).

AGl and AGm have been considered as parts of the primary motor cortex because both project to the spinal cord (Wise et al. 1979). However, stimulation evoked movements revealed representation fields for all body parts in AGl but not in AGm (Donoghue and Wise 1982; Neafsey et al. 1986). Neafsey et al. (1986) showed that the part of AGm which extends rostral to Agl is probably the premotor and supplementary motor cortex, while the part of AGm which lies medial to Agl is the frontal eye field and the vibrissae motor area. Thus, the frontal eye field, which is located in the medial frontal cortex, would occupy the posterior part of our Fr2, whereas its anterior part would contain the premotor and supplementary motor cortex. Although Donoghue and Wise (1982) and Neafsey et al. (1986) noticed that there are some features within AGl that would allow its subdivision into two parts, they proposed to consider the cytoarchitectonic differences (agranular vs. dysgranular) as signs of somatotopic subdivisions of the primary motor cortex. This observation is in acordance with our findings of variations in M_2_ receptor densities within Fr1 and Fr2, which we also interprest as reflecting somatotopic motor subdivisions. As in the present analysis, Zilles (1985) and Palomero-Gallagher and Zilles (Palomero-Gallagher and Zilles 2004, 2015) subdivided AGl into two cortical fields, namely Fr1 and Fr3, based first on differences in cyto- and myeloarchitecture, and in the latter studies also including a receptor architectonic analysis. Fr1 and Fr3 areas would constitute two subdivisions of AGl, and represent two different somatotopic subfields of the primary motor cortex in electrophysiological studies (Donoghue and Wise 1982). The motor head field, including tongue, lips and jaw, are represented in a region of the lateral frontal cortex coextensive with Fr3 in the present map (Fabri and Burton 1991a; Hall and Lindholm 1974; Neafsey et al. 1986; Tandon et al. 2008). The representation field for wrist and digits of the forelimb, called rostral forelimb area (RFA), is located medial to the head field of Fr3 in Fr1 (Neafsey and Sievert 1982). Medial to RFA is the motor representation field of the vibrissae. A second forelimb area for the proximal parts (shoulder and elbow) was found caudal to RFA (Neafsey et al. 1986; Tandon et al. 2008). Trunk, neck and hind-limb are represented in Fr1 caudal to CFA. The medial frontal cortex is represented by Fr2 in the present study. This area is most probably the equivalent of AGm (Donoghue and Wise 1982) and PrCm (Zilles et al. 1980).

In contrast to the situation found in the sensory functional systems, in which the primary areas show the highest density of M_2_ receptors, the primary motor cortex (coextensive with our areas Fr1 and Fr3) shows a lower M_2_ receptor density than the premotor and supplementary motor cortices (located within our area Fr2). This feature is not unique to the rat brain, but has also been described in the human and macaque monkey brains (Geyer et al. 1998; Rapan et al. 2021; Zilles and Palomero-Gallagher 2017a, b), and thus seems to constitute an evolutionary constant in the molecular organization of networks subserving sensory and motor functions.

Within the cingulate cortex we identified areas Cg1, Cg1’, Cg2d, Cg2v, Cg2’d, Cg2’v and Cg3. Area Cg3 is comparable in location to the rostral two thirds of area A32D of Paxinos and Watson (Paxinos and Watson 2013), whereas the caudal third of A32D, together with their A24b, corresponds to our area Cg1. Areas Cg2d and Cg2v constitute dorsal and ventral subdivisions of our previously identified area Cg2 (Palomero-Gallagher and Zilles 2015), and are the equivalent of A24a and A33, respectively, of Paxinos and Watson (2013). Likewise, Cg2’d and Cg2’v are found within our previously described Cg2’ (Palomero-Gallagher and Zilles 2015), and correspond to A24a’ and A33, respectively, of Paxinos and Watson (2013). We found the distribution of M_2_ receptors to clearly segregate Cg2d and Cg2v from Cg2’d and Cg2’v, respectively, with considerably higher densities in the former than in the latter areas. In the mouse brain areas Cg2d and Cg2v differ from their caudal counterparts in the density and topography of their connectivity patterns (Fillinger et al. 2017, 2018). Vogt and Paxinos (2014) proposed that the portion of rodent cingulate cortex occupied by areas A24a’ and A24b’ should be considered homolog of the primate midcingulate cortical region (Vogt et al. 2003, 2005). Interestingly, a recent meta-analysis indicates a specific role of rodent midcingulate cortex in the regulation of task monitoring and visuospatial orientation, functions which have also been associated with the human midcingulate cortex (van Heukelum et al. 2020).

In accordance with numerous previously published maps of the rat *orbital cortex* (e.g., (Kondo and Witter 2014; Krettek and Price 1977; Murphy and Deutch 2018; Price 2007; Ray and Price 1992; Van De Werd and Uylings 2008), we identified five proisocortical areas based on differences in cytoarchitecture and M_2_ receptor distribution patterns. Interestingly, Paxinos and Watson (2013) only identify four areas, whereby our area DLO is comparable in location and extent to their equally named area, but their MO is smaller than ours, because it is replaced by their area A32V, which reaches much further rostrally than does our area Cg3. Importantly, we identify area VLO between VO and LO, whereas Paxinos and Watson (2013) do not, so that their areas VO and LO share a common border. Based on the location, extent and relative size of these two areas in the map of Paxinos and Watson (2013), it seems that their LO encompasses our areas VLO and LO. It is more plausible that this region of the rat orbital cortex covers three areas (rather than two) given that VO, VLO and LO not only differ in their cytoarchitecture as revealed by classical histological stainings and in their molecular structure as determined by immunohistochemical and receptor autoradiographic analyses (present study; (Kondo and Witter 2014; Linley et al. 2013; Van De Werd and Uylings 2008), but also in their connectivity patterns (Kondo and Witter 2014; Krettek and Price 1977; Murphy and Deutch 2018; Price 2007; Ray and Price 1992).

### Parietal cortex

The parietal cortex is named granular (or in parts dysgranular) cortex because it is, histologically, characterized by the presence of a more or less prominent inner granular layer (layer IV). The parietal cortex receives, processes, integrates and interprets sensory information pertaining touch, vibration, pain and temperature from skin and joints, and thus constitutes the somatosensory cortex of the rat (for a comprehensive review see (Ebner and Kaas 2015).

#### The central parietal region

Among the three currently existing anatomical rat brain atlases (Paxinos and Watson 2013; Swanson 2004; Zilles 1985), that of Swanson (2004) has the simplest parcellation for parietal cortex; which he divided into two areas: SSp (primary somatosensory) and SSs (secondary somatosensory). SSp in Swanson’s map encompasses our areas Par1, ParHL and ParFL whereas SSs is equivalent of Par2 in our map. Zilles (1985) defined four areas in the central parietal region: Par1, FL, HL and Par2, whereby areas FL and HL are equivalent of ParFL and ParHL in our map. Paxinos and Watson (2013) provided the most detailed of the three parcellation maps, with identification of all somatotopic representations within the central parietal regions, and although it is an anatomical rat brain atlas, the parcellation and nomenclature systems used are mostly derived from electrophysiological studies (e.g., Chapin and Lin 1984; Dawson and Killackey 1987; Welker 1971). Paxinos and Watson (2013) divided the central parietal region into two main areas: primary somatosensory area S1 and secondary somatosensory area S2, which are equivalent of Par1 and Par2 in our map, respectively. Within S1, they identified and named somatotopic representations for different body parts based on electrophysiological studies, as well as two narrow dysgranular areas which were delineated and named according to histological and topographical features.

Par1 is the topographical equivalent of the electrophysiologically identified S1. S1 in rats, like other mammals, contains a complete and inverted somatotopic representation of the contralateral half of body, located along the mediolateral extension of the parietal cortex (Krubitzer et al. 2011; Santiago et al. 2007). Because of the higher sensory receptor density in the head region, including vibrissae, lips and tongue, there is a huge magnification of the representation for these areas on the parietal cortex which has been denominated “barrel field” (Chapin and Lin 1984; Santiago et al. 2007). This area has a characteristic feature in histological Nissl staining, the so-called “barrels” (Woolsey and Van der Loos 1970; Woolsey et al. 1975), which are separated from each other by cell-sparse dysgranular regions called septa (Santiago et al. 2007; Woolsey and Van der Loos 1970; Woolsey et al. 1975). It is of note that rat’s whiskers not only compensate for the poverty of visual inputs to the rat brain (Petersen 2007), but can be used to manipulate objects in order to recognize them (stereognosia), a function for which in primates use their hands (Krubitzer et al. 2011). Par1 is characterized by conspicuously higher M_2_ receptor densities in layer IV but lower densities in layer Va than adjacent areas. Furthermore, the somatotopic representations are associated with variations in the density of M_2_ receptors in layer IV, which are particularly high in the vibrissae representation area Par1BF. The dysgranular zones described by Chapin and Lin (1984) can also be clearly identified due to their thinner layer IV, which presents a and slightly higher M_2_ receptor density than that of adjacent portions of Par1.

ParFL and ParHL are somatotopic representations of the forelimb and hindlimb within Par1, respectively. However, they are classified as distinct areas, unlike the other sensory somatotopic representations in Par1, because electrophysiological studies have demonstrated an overlap within these regions between sensory and motor representations of the forelimb and hindlimb (Hall and Lindholm 1974; Krubitzer et al. 2011; Santiago et al. 2007).

Par2 is equivalent of S2 in electrophysiological maps (Benison et al. 2007; Brett-Green et al. 2004; Fabri and Burton 1991a; Remple et al. 2003; Welker and Sinha 1972). Electrophysiological studies revealed that S2 lies just below the vibrissae, nose and upper lips representations in S1, and contains a somatotopic representation of the contralateral body which is a mirror reversal of the somatotopic map in S1. Thus, in contrast to S1, the somatotopic map in S2 is upright (Fabri and Burton 1991a; Krubitzer et al. 2011; Remple et al. 2003). Interestingly, although layer IV of Par2 presents variations in M_2_ receptor density, they are not as pronounced as those described for layer IV of Par1, and we were not able to establish a correlation with somatotopic representations. This could be due to the fact that receptive fields in S2 are generally larger and less responsive in the anesthetized rat than those in S1 (Krubitzer et al. 2011; Remple et al. 2003). Par2 has a generally lower M_2_ receptor density than that of Par1. Indeed, the fact that the primary sensory areas show significantly higher M_2_ receptor densities than surrounding areas is an evolutionarily conserved characteristic of mammalian sensory cortices (Zilles & Palomero-Gallagher 2017a).

#### The ventral parietal region

The ventral parietal region (ParV) and its subdivisions (ParVr and ParVc) were identified and named by Palomero-Gallagher and Zilles (Palomero-Gallagher and Zilles 2004, 2015) based on cyto- and receptor architectonic features. Our ParVr is located within the ventro-rostral portion of area S1 of Paxinos and Watson (2013) and SSp of Swanson (2004). Our ParVc occupies the same topographic location as the visceral area (VISC) of Swanson (2004), and as the most ventral part of area S2 of Paxinos and Watson (2013). We believe that our classification of this band of cortex as belonging to the parietal region rather than to the insular cortex is correct, because ParVr and ParVc do not cover the claustrum. Furthermore, their M_2_ receptor distribution pattern, in particular the conspicuously high density in layer IV, is more similar to that of the remaining parietal than of the insular areas. Additionally, our identification further supports the existence of a third representation of the contralateral body just lateral to S2, which was characterized based on electrophysiological and microinjection tracing studies and named the parietal ventral area (PV; Fabri and Burton 1991a; Remple et al. 2003). The somatotopic map in PV is similar in size and shape to that of S2, but it is inverted, as is the case in S1 (Fabri and Burton 1991a; Krubitzer et al. 2011; Remple et al. 2003). The PV regions of forelimb and hindlimb are continuous with those described for S2, and are found at a location occupied by our area ParVc. The representation area for upper and lower incisors located rostral to S2 and to the upper lip representation in PV (Remple et al. 2003) would be equivalent of our ParVr, which lies ventral to Par1.

#### The posterior parietal region

Caudal to Par1 we identified a posterior parietal region which corresponds to area PTLp of Swanson (2004) and areas PtPD and PtPR of Paxinos and Watson (2013). The posterior parietal region in our map is also in topographical agreement with the posterior parietal cortex of physiological studies, which was found to act as a multimodal association cortex (Lee et al. 2011; Reep and Corwin 2009). There are some homologies between the posterior parietal region of the rat and the posterior parietal cortex of primates in location, functions and connections (Bushara et al. 1999; Reep et al. 1994; Reep and Corwin 2009; Torrealba and Valdes 2008). It receives extensive afferents from dysgranular zones within S1 cortex (Fabri and Burton 1991b; Lee et al. 2011), visual cortex and non-primary sensory nuclei of thalamus such as lateral dorsal, lateral posterior and posterior nuclei (Reep et al. 1994). The posterior parietal cortex has reciprocal connections with retrosplenial, visual and motor cortices (Corwin and Reep 1998; Reep et al. 1994), and is interconnected with Par2 in a topographically organized manner (Olsen et al. 2019). Single neuron recordings in freely behaving rats have demonstrated the involvement of the posterior parietal cortex in navigating the environment (Nitz 2009) and in spatial attention, as has been established for the posterior parietal cortex in primates (Bucci 2009; Corwin and Reep 1998; Reep and Corwin 2009; Torrealba and Valdes 2008). Furthermore, lesions to posterior parietal cortex were used to successfully develop a rat model of human hemi-neglect syndrome (Reep et al. 2004).

### Insular cortex

We divided the insular cortex rostro-caudally into two subregions and dorsoventrally into three longitudinal stripes according to cytoarchitectonic criteria and differences in M_2_ receptor distribution patterns: a dorsal granular part with areas GIa and GIp, a middle dysgranular part composed of area DI, and a ventral agranular part with areas AId, AIv and AIp, whereby the granular and agranular cortex can each be divided into rostral and caudal portions. In the primate brain, only the granular part of the insular cortex has been classified as being isocortical, whereas the dysgranular and agranular portions are considered to belong to the proisocortex and periallocortex, respectively (Mesulam and Mufson 1985). Areas GIa and GIp occupy the part of insular cortex identified as gustatory area (GU) by Swanson (2004) and granular insular area (GI) by Paxinos and Watson (2013) and Van De Werd and Uylings (2008), and area DI is the equivalent of the equally named areas of these authors. Our parcellation of AId, AIv and AIp is in complete agreement with that of Swanson (2004) and Paxinos and Watson (2013). The parcellation of the agranular insular cortex by Van De Werd and Uylings (2008) differs slightly in nomenclature, since their areas AId1 and AId2 correspond to our areas AId and AIv, respectively.

The insular cortex plays a crucial role in the processing of interoceptive, gustatory, olfactory, auditory, somatosensory, and nociceptive stimuli, as well as in the coding of emotional and affective states (for a comprehensive review see (Livneh and Andermann 2021). The cytoarchitectonic segregation of the insular cortex is reflected in its connectivity and functional topography, since two rosto-caudally arranged distinct subregions have been identified, each of which contains granular, dysgranular and agranular areas, which differ in their connectivity patterns (Mathiasen et al. 2015; Shi and Cassell 1998b). The anterior portion of the insula is involved in higher associative sensory functions associated e.g., with food reward value, pain modulation or short-term memory for odour location (DeCoteau et al. 1997; Di Pietro et al. 2004; Jasmin et al. 2003; Ragozzino and Kesner 1999; Saper 2002), whereas the posterior part receives direct input from the gustatory and visceral thalamic nuclei (in particular area DI) and is involved in viscerosensory perception (Cechetto and Saper 1987; Saper 2002; Shi and Cassell 1998b). Shi and Cassell (1998a, b) further subdivided the posterior insular cortex into a more rostrally located “posterior” and a more caudally located “parietal” insular domain based on a differential connectivity with the anterior insular areas and the secondary somatosensory cortex. However, these differences do not seem to be associated with changes in cortical architecture. Although visceral and sensory input from the tongue are topographically organized within area DI, there is a considerable degree of overlap between the representations of these two sensory modalities (Cechetto and Saper 1987; Kosar et al. 1986; McDonald et al. 1999).

### Temporal cortex

We here identified a primary temporal area (Te1) in the central position within the anterior half of the temporal cortex and surrounded by secondary and association temporal cortex (areas Te2 and Te3), which can be further divided into dorsal (areas Te2d and Te3r) and ventral (Te2v and Te3v) components. This differs from our previous map of the temporal cortex (Palomero-Gallagher and Zilles 2015) in that we then identified a further temporal area, TeV, which (as discussed below) in the present map we have redefined as ectorhinal cortex.

Although the three currently existing anatomical rat brain atlases (Paxinos and Watson 2013; Swanson 2004; Zilles 1985) agree in that the temporal cortex includes a “core and belt complex”, there are discrepancies concerning the number, extent and nomenclature of belt areas. Our area Te1 corresponds to Au1 of Paxinos and Watson (2013) and AUDp of Swanson (2004) and is the equivalent of the functionally defined primary auditory cortex. Our Te3r and Te3v cover approximately the rostral two-thirds of areas AuD and AuV of Paxinos and Watson (2013), (2004) respectively, the caudal third being occupied by the most rostral portion of our areas Te2d and Te2v, respectively. Areas Te2d and Te2v continue further caudally along cortex which in the map of Paxinos and Watson (2013) is occupied completely by the posterior portion of their area TeA (Table 1). In the map of Swanson (2004) areas Te3r and Te3v are found at a location which topologically corresponds to his AUDd and AUDv, respectively, whereas Te2d and Te2v would correspond to his AUDpo and the posterior portion of his TEa, respectively. The rostral part of area TeA/TEa of Paxinos and Watson (2013) and Swanson (2004) is occupied by our ectorhinal area EctD (Table 1).

In contrast to the primate brain, where all primary sensory areas are characterized by the highest density of M_2_ receptors (Zilles and Palomero-Gallagher 2017a), in the rat temporal cortex M_2_ receptor densities decrease continuously in the dorsoventral direction. Thus, Te2d has the highest, Te1 has only moderate, and Te2v has the lowest density of M_2_ receptors, and this situation could be due to the considerably higher degree of myelination of Te1 compared to Te2 or Te3 (Palomero-Gallagher and Zilles 2015; Zilles 1985), whereas in the human cortex areas surrounding the primary auditory cortex also present a high degree of myelination (Palomero-Gallagher and Zilles 2019).

### Occipital cortex

The occipital cortex contains the visual cortex of the rat and can be divided into a primary occipital area (Oc1) or primary visual area (V1) in the centre of the occipital cortex, which is surrounded by a secondary occipital cortex (Oc2) or secondary visual cortex (V2). Similar to the situation described for the auditory system, while all currently existing comprehensive maps of the rat cortex are in agreement with the relative location of the primary visual cortex, however, there are considerable differences regarding how the secondary occipital cortex lateral and medial to Oc1 is organized. All maps depict a subdivision of the primary visual cortex into two areas which form two rostro-caudally oriented parallel bands along the posterior third of the dorsal surface of hemisphere: our areas Oc1M and Oc1B correspond to the equally named areas of Zilles (1985) and Palomero and Zilles (2015), areas V1M and V1B of Paxinos and Watson (2013), respectively, and area VISp of Swanson (2004). Area Oc1M, which lies medial to Oc1B, receives visual input from the temporal part of the contralateral visual hemifield just through the contralateral eye and thus constitutes the monocular portion of the primary visual cortex, while Oc1B receives visual input from the nasal part of the contralateral visual hemifield through both eyes, and constitutes the binocular portion of the primary visual cortex (Zilles et al. 1984). As described for the primate brain (Zilles and Palomero-Gallagher 2017a), the rat primary visual cortex is characterized by a considerably higher density of muscarinic cholinergic M_2_ receptors. However, in the rat brain these higher densities are restricted to layer IV, the main recipient of thalamo-cortical projections, and which is also particularly targeted by cholinergic innervation (Eckenstein et al. 1988).

Analysis of the present dataset, which provides a much tighter sampling of the M_2_ receptors than previously available (Palomero-Gallagher and Zilles 2004, 2015), led to the subdivision of Oc2MM into areas Oc2MMa and Oc2MMp, and confirmed the existence of Oc2ML. Oc2Mma and Oc2MMp correspond to area V2MM of Paxinos and Watson (2013), and Oc2ML to their V2ML. Interestingly, our Oc2MMp is comparable to VISpm of Swanson (2004), and our areas Oc2Mma and Oc2ML to his VISpa. Electrophysiological and tracer studies revealed the existence of three retinotopically organized fields medial to the primary visual cortex and with distinct connectivity patterns (Espinoza and Thomas 1983; Montero 1993): posteromedial (PM), anteromedial (AM), and anterior (A) areas, which would be functional correlates of our Oc2MMp, Oc2Mma and Oc2ML, respectively.

The present study also resulted in considerable changes concerning the parcellation and classification of cortex located lateral to Oc1. Whereby at this location we previously only identified a single occipital area, namely Oc2L (Palomero-Gallagher and Zilles 2004, 2015; Zilles 1985), which corresponds to V2L of Paxinos and Watson (2013), we have now defined areas Oc2Lr, Oc2Lid, Oc2Liv and Oc2Lc (Table 1). Area Oc2L of Zilles (1985) encompasses Oc2Lr, Oc2Lc and Oc2Lid, whereas Oc2Lr occupies cortex which we previously identified as ParPC (Palomero-Gallagher and Zilles 2004, 2015). Oc2Lr was previously classified as a parietal area because its layer V pyramids are clearly larger than those of occipital areas, and only slightly smaller than those of the adjacent parietal cortex. However, the tighter sampling of M_2_ receptors revealed that it shares more similarities with occipital than with parietal areas based on relative differences in laminar receptor densities. Most importantly, layer Va of Oc2Lr does not stand out by its conspicuously lower density of M_2_ receptors than adjacent layers, as is the case in the parietal cortex. Thus, as is the case in occipital areas, the border between layers Va and Vb of Oc2Lr is not clearly revealed by the M_2_ receptor. Furthermore, the considerably lower M_2_ density in layer IV of Oc2Lr than of parietal areas further warrants its classification as occipital cortex. Our area Oc2Lr most probably corresponds to rostrolateral visual area (VISrl) of Swanson (2004), whereas our Oc2Lc covers cortex occupied by his intermediolateral (VISli], posterolateral (VISpl) and VISx visual areas. Our area Oc2Lid encompasses the anterolateral (VISal) and mediolateral (VISlm) visual areas of Swanson (2004), whereas our area Oc2Liv covers his anterior laterolateral (VISall) and laterolateral (VISll) visual areas (Table 1). Our parcellation of the lateral occipital cortex is in accordance with the results of electrophysiological and connectivity studies (Espinoza and Thomas 1983; Montero 1993; Olavarria and Montero 1984), which identified anterolateral (AL), lateromedial (LM) and laterolateral (LL) areas comparable in location to our Oc2Lr, Oc2Lid and Oc2Liv, respectively. We were not, however, able to identify architectonic correlates of their laterointermediate (LI) and posterolateral (PL) areas (Espinoza and Thomas 1983; Montero 1993; Olavarria and Montero 1984), since both areas are located within our Oc2Lc.

### Retrosplenial cortex

On the dorsomedial surface of the hemisphere, medial to the secondary occipital area Oc2MM and the most posterior portion of Fr2, we identified RSA, the proisocortical portion of the retrosplenial cortex, and equivalent of A30 of Paxinos and Watson (2013), and RSPagl of Swanson (2004). RSA is involved in spatial learning processes (Pothuizen et al. 2009) and has also been designated as the dysgranular subdivision of the retrosplenial cortex (Pothuizen et al. 2009; Vogt and Peters 1981), since a layer IV is present, although variable in thickness and invaded by layer III and layer V pyramids. RSA presents a conspicuous stripe of high M_2_ receptor density in layer III, but very low values in layers IV-VI. The superficial layers of RSA are characterized, together with layers V and IV, by a high density of cholinergic terminals (Eckenstein et al. 1988), and also receive projections from RSG (Tsai et al. 2022), the postsubiculum and the laterodorsal nucleus (van Groen and Wyss 1992).

### Ecto-, peri-, and postrhinal cortices

Cortex around (and dorsal to) the posterior half of the rhinal sulcus encompasses areas interconnecting the hippocampal formation and entorhinal cortex with the lateral occipitotemporal and medial prefrontal association cortex, and are involved in memory and sensory perception processing (Burwell and Amaral 1998; Burwell et al. 1995; de Curtis and Pare 2004; Doan et al. 2019; Fernandez and Tendolkar 2006; Hwang et al. 2018). In the primate brain, Brodmann (1909) defined a perirhinal area 35 and an ectorhinal area 36 in the vicinity of the rhinal sulcus and mentioned the existence of homologous areas in the rodent brain. Krieg (Krieg 1946b, c) adopted this nomenclature for the rat brain, where he identified two longitudinally oriented strips—36 dorsally and 35 ventrally—with distinct cytoarchitectonic features. Differences in connectivity later resulted in the subdivision of Krieg’s area 35 into a rostral portion which was named perirhinal area and a posterior portion designated postrhinal area (Deacon et al. 1983).

Interestingly, the maps of Paxinos and Watson (2013) and of Swanson (2004) depict an ectorhinal area (Ect and ECT, respectively) dorsally and a single perirhinal area (PRh and PERI, respectively) ventrally, whereas Burwell et al. (1995) propose a principal rostro-caudal subdivision of this brain region rather than a dorsoventral one. They identified a perirhinal region rostrally which includes a dorsal area 36 and a ventral area 35, and a posterior region composed of the postrhinal area, which they consider to be the rat homolog of macaque parahippocampal areas TF and TH (Burwell et al. 1995). More recently, Burwell (2001) described cytoarchitectonic and histochemical criteria enabling the definition of dorsal and ventral subdivisions within area 35 (35d and 35v, respectively) and the postrhinal cortex (PORd and PORv, respectively), as well as a tripartition of area 36 into one posterior (36p) and two rostral (36d dorsally and 36v ventrally) subdivisions. Our identification of dorsal and ventral subdivisions within the perirhinal and the postrhinal regions is in accordance with areas 35d/35v and PORd/PORv, respectively, identified by Burwell (2001). We delineated three areas within the ectorhinal cortex: EctP covering its caudal half, and delimited rostrally by EctD and EctV. Both EctD and EctP are dysgranular, and classified as part of the isocortex, whereas EctV is an agranular proisocortical area (Burwell et al. 1995). Our areas EctD, EctV and EctP correspond to areas 36d, 36v and 36p of Burwell (2001), respectively, whereby EctP extends slightly further caudally than does 36p.

In our previous map (Palomero-Gallagher and Zilles 2015) we had identified cortex now occupied by the ectorhinal areas as area TeV, since its M_2_ receptor distribution pattern more closely resembles that of the dorsally adjacent temporal areas than that of the perirhinal cortex, and Brodmann (1909) and Krieg (Krieg 1946b, c) had assigned their area 36 to the temporal region, and not to the rhinal region. However, we here decided to apply the widely used term ectorhinal because this cortical region seems to share more connectivity and functional similarities with the perirhinal and postrhinal areas than with temporal areas Te2v or Te3v. Indeed, the ectorhinal and perirhinal areas, but not the temporal association cortex (which roughly corresponds to our areas Te2v, Te2d, and Te3v), are found in the top 20^th^ percentile of the degree, strength, betweenness and closeness centrality measures as revealed by the strength of intrahemispheric connectivity of rat brain areas (Swanson et al. 2017). Thus, ectorhinal and perirhinal areas are thought to constitute hubs in the rat macro-connectome together with AIp, the entorhinal cortex, and the lateral and basolateral amygdalar nuclei (Swanson et al. 2017). Functionally, the ectorhinal and perirhinal areas have been associated in rats with the processing of visual and tactile stimuli (Nishio et al. 2018; Ramos 2014), whereas the temporal association cortex is involved in higher order auditory but not visual processes (Davis and McDaniel 1993; Ellard 1998; Feigin et al. 2021), and is also activated by auditory-driven maternal behaviour (Tasaka et al. 2020).

Concluding, the present study provides a comprehensive parcellation of the entire rat iso- and proisocortex based on the analysis of cell bodies and of the muscarinic cholinergic M_2_ receptor as a functionally relevant molecule and evolutionarily conserved marker of primary sensory areas (Zilles and Palomero-Gallagher 2017a). We describe the cyto-and M_2_ receptor architectonic features enabling the identification of 48 distinct areas across the frontal, parietal, temporal, occipital, cingulate, retrosplenial, orbital, insular, ectorhinal, perirhinal, and postrhinal regions and provide the mean M_2_ receptor densities extracted from each of the identified areas. This data, together with the atlas provided in the Supplementary Material, in which we have also labelled components of the hippocampal formation as well as multiple subcortical and brain stem nuclei, constitutes a useful tool for future computational and experimental neuroscientific studies.

## Supplementary Information

Below is the link to the electronic supplementary material.

Supplementary file1 (PDF 56679 kb)

## Data Availability

All data generated or analysed during this study are included in this published article and its supplementary information files.
