# Supplementary material for: A new map of the rat isocortex and proisocortex: cytoarchitecture and M2 receptor distribution patterns
Source: Brain Struct Funct. 2023 Jun 15;229(8):1795–822. doi: 10.1007/s00429-023-02654-7 (PMC11485150; doi:10.1007/s00429-023-02654-7)

A new map of the rat isocortex and proisocortex: Cytoarchitecture and M_2 receptor distribution patterns

(Supplementary material)

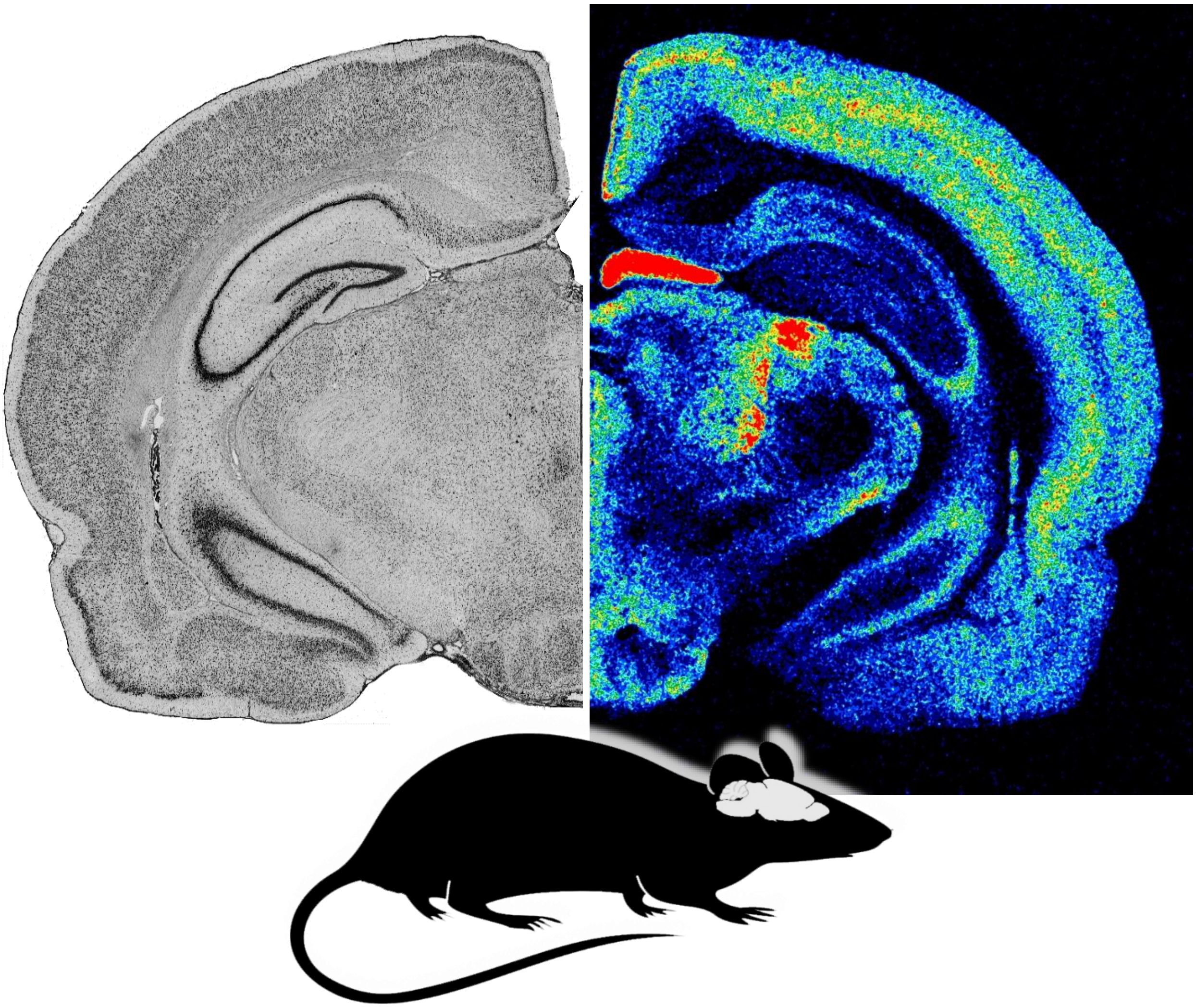

Hossein Haghir*, Anika Kuckertz*, Ling Zhao, Javad Hami,

Karl Zilles, Nicola Palomero-Gallagher

*Authors contributed equally

JÜLICH Forschungszentrum

INDEX

3V	third ventricle
4V	fourth ventricle

A

AA	anterior amygdaloid area
ac	anterior commissure
AcbC	accumbens nucleus, core
AcbR	accumbens nucleus, rostral pole
AcbSh	accumbens nucleus, shell
ACo	amygdala, anterior cortical nucleus
AD	anterodorsal thalamic nucleus
AE	amygdalo-entorhinal transition area
AHiAL	amygdalohippocampal area, anterolateral part
AHiPL	amygdalohippocampal area, posterolateral part
AHiPM	amygdalohippocampal area, posteromedial part
AId	insular cortex, agranular part, dorsal region
AIp	insular cortex, agranular part, posterior region
AIv	insular cortex, agranular part, ventral region
AM	anteromedial thalamic nucleus
AmbC	nucleus ambiguus, compact part
AO	anterior olfactory nucleus
AOd	anterior olfactory nucleus, dorsal part
AOe	anterior olfactory nucleus, external part
AOl	anterior olfactory nucleus, lateral part
AOm	anterior olfactory nucleus, medial part
AOp	anterior olfactory nucleus, posterior part
AOv	anterior olfactory nucleus, ventral part
AOvp	anterior olfactory nucleus, ventroposterior part
APir	amygdalopiriform transition area
aq	aqueduct
ASt	amygdalostriatal transition area
AV	anteroventral thalamic nucleus

B

BLA	amygdala, basolateral nucleus, anterior part
BLP	amygdala, basolateral nucleus, posterior part
BLV	amygdala, basolateral nucleus, ventral part
BMA	amygdala, basomedial nucleus, anterior part
BMP	amygdala, basomedial nucleus, posterior part

C

CA1	CA1 field of hippocampus
CA2	CA2 field of hippocampus
CA2/3	CA2 and CA3 fields of hippocampus
CA3	CA3 field of hippocampus
CA4	CA4 field of hippocampus
CB	cell bridges of the ventral striatum
Cb	cerebellum
cbw	cerebellar white matter
cc	corpus callosum
CC	central canal
CE	caudal entorhinal field
Ce	amygdala, central nucleus
CeC	amygdala, central nucleus, capsular part
CeL	amygdala, central nucleus, lateral division
CeM	amygdala, central nucleus, medial division
CG	central gray
Cg1	cingulate cortex, area 1
Cg1'	cingulate cortex, area 1'
Cg2d	cingulate cortex, area 2, dorsal part
Cg2'd	cingulate cortex, area 2', dorsal part
Cg2v	cingulate cortex, area 2, ventral part
Cg2'v	cingulate cortex, area 2', ventral part
Cg3	cingulate cortex, area 3
cic	commissure of inferior colliculi
Cl	claustrum, anterior part
Cld	claustrum, dorsal part
Clv	claustrum, ventral part
CM	central medial thalamic nucleus
cpd	cerebral peduncle
CPu	caudate-Putamen (striatum)
CS	superior central raphe nucleus
csc	commissure of superior colliculi
cst	corticospinal tract
cu	cuneate fascicle
CUN	cuneiform nucleus
CxA	amygdala-cortex transition zone

D

DC	dorsal cochlear nucleus
DEn	dorsal endopiriform nucleus
df	dorsal fornix
DI	insular cortex, dysgranular part
DIE	dorsal intermediate entorhinal field
DIp	insular cortex, posterior dysgranular part
DLE	dorsal lateral entorhinal field
DLO	orbital cortex, dorsolateral part
DMX	dorsal motor nucleus of vagus nerve
DN	dentate nucleus
DP	dorsal peduncular cortex
DR	dorsal raphe
dscp	superior cerebellar peduncle
DTr	dorsal transition zone
DTT	Dorsal tenia tecta

E

EctD	ectorhinal cortex, anterior part, dorsal region
EctP	ectorhinal cortex, posterior part
EctV	ectorhinal cortex, anterior part, ventral region
ECU	external cuneate nucleus
em	external medullary lamina

F

f	fornix
FC	fasciola cinerea
FD	fascia dentata
fi	fimbria of hippocampus
fr	fasciculus retroflexus
Fr1	frontal cortex, area 1
Fr2	frontal cortex, area 2
Fr3	frontal cortex, area 3

G

Gi	gigantocellular reticular nucleus
Gia	insular cortex, granular part, anterior region

Gip	insular cortex, granular part, posterior region
GP	globus pallidus
Gr	gracile nucleus

I

I	amygdala, intercalated nucleus
IAM	interanteromedial thalamic nucleus
Ic	internal capsule
IC	inferior colliculus
icp	inferior cerebellar peduncle
IEn	intermediate endopiriform nucleus
III	oculomotor nucleus
IL	infralimbic cortex
IO	inferior olivary complex
IP	interposed nucleus
IPN	interpeduncular nucleus

L

LaDL	amygdala, lateral nucleus, dorsolateral part
LaVL	amygdala, lateral nucleus, ventrolateral part
LaVM	amygdala, lateral nucleus, ventromedialpart
LC	locus coeruleus
Ld	lambdoid septal zone
LD	lateral dorsal thalamic nucleus
LDT	laterodorsal tegmental nucleus
LG	lateral geniculate complex
LHb	lateral habenular nucleus
lo	lateral olfactory tract
LO	orbital cortex, lateral part
Lot	nucleus of the lateral olfactory tract
LP	lateral posterior thalamic nucleus
LRN	lateral reticular nucleus
LSD	lateral septal nucleus, dorsal part
LSI	lateral septal nucleus, intermediate part
LSV	lateral septal nucleus, ventral part
LTer	lemina terminalis
LV	lateral ventricle
LVe	lateral vestibular nucleus

M

mcp	middle cerebellar peduncle
MD	mediodorsal thalamic nucleus
MDL	Mediodorsal thalamic nucleus, lateral part
MDRN	medullary reticular nucleus
ME	medial entorhinal field
MeAD	amygdala, medial nucleus, anterodorsal part
MeAV	amygdala, medial nucleus, anteroventral part
Med	medial cerebellar nucleus
MePD	amygdala, medial nucleus, posterodorsal part
MePV	amygdala, medial nucleus, posteroventral part
MGN	medial geniculate nucleus
MHb	medial habenular nucleus
ML	medial mammillary nucleus, lateral part
ml	medial lemniscus
mlf	medial longitudinal fascicle
MM	medial mammillary body
MO	orbital cortex, medial part
MS	medial septal nucleus
mt	mammillothalamic tract
MV	medial vestibular nucleus

N

NLL	nucleus of the lateral lemniscus
NTB	nucleus of the trapezoid body
NTS	nucleus of the solitary tract
Nv	navicular nucleus of the basal forebrain

O

OB	olfactory bulb
Oc1B	binocular (nasal) primary occipital area
Oc1M	monocular (temporal) primary occipital area
Oc2Lr	rostrolateral secondary occipital cortex
Oc2Lc	caudolateral secondary occipital cortex
Oc2Lid	intermediate dorsolateral secondary occipital cortex
Oc2Liv	intermediate ventrolateral secondary occipital cortex
Oc2ML	mediolateral secondary occipital cortex
Oc2MMa	mediomedial secondary occipital cortex, anterior portion
Oc2MMp	mediomedial secondary occipital cortex, posterior portion
och	optic chiasm
ON	optic nerve
opt	optic tract
ov	olfactory ventricle

P

PaF	parafascicular nucleus
PAG	periaqueductal gray
Par1	parietal cortex, area 1
Par1BF	parietal cortex, area 1, barrelfield representation
Par1DZ	parietal cortex, area 1, dysgranular zone
Par1DZO	parietal cortex, area 1, oral dysgranular zone
Par1Sh	parietal cortex, area 1, shoulder representation
Par1ULp	parietal cortex, area 1, upper lip representation
Par2	parietal cortex, area 2
ParFL	parietal cortex, forelimb area
ParHL	parietal cortex, hindlimb area
ParPd	parietal cortex, posterior part, dorsal region
ParPv	parietal cortex, posterior part, ventral region
ParVc	parietal cortex, ventroposterior part
ParVr	parietal cortex, ventroanterior part
PaSd	parasubiculum, dorsal part
PaSv	parasubiculum, ventral part
PB	parabrachial nucleus
pc	posterior commissure
PF	parafascicular thalamic nucleus
PG	pontine gray
Pi	pineal gland
Pir1	piriform cortex, area 1
Pir2	piriform cortex, area 2
PLCo	amygdala, posterolateral cortical nucleus
pm	peincipal mammillary tract
PMCo	amygdala, posteromedial cortical nucleus
Pn	pontine nuclei
PnC	pontine reticular nucleus, caudal part

PoRhD	postrhinal cortex, dorsal part
PoRhV	postrhinal cortex, ventral part
PoS	postsubiculum
Pr	nucleus prepositus
PRhD	perirhinal cortex, dorsal part
PRhV	perirhinal cortex, ventral part
ProS	prosubiculum
ProStr	area prostriata
PrS	presubiculum
PrV	principal sensory trigeminal nucleus
py	pyramidal tract

R

Re	reuniens nucleus
Rh	rhomboid nucleus
RM	nucleus raphe magnus
RN	red nucleus
RSA	retrosplenial cortex, agranular part
RSGa	retrosplenial cortex, granular part, region a
RSGb	retrosplenial cortex, granular part, region b
RSGc	retrosplenial cortex, granular part, region c
Rt	reticular thalamic nucleus
RtTg	Reticulotegmental nucleus

S

S	subiculum
SAG	sagulum nucleus
SC	superior colliculus
scp	superior cerebellar peduncle
SEZ	subependymal zone
SFi	septofimbrial nucleus
SHi	septohippocampal nucleus
SHy	septohypothalamic nucleus
sm	stria medullaris thalami
SMT	submedial thalamic nucleus
SN	substantia nigra
Sol	nucleus of the solitary tract
SpIV	spinal vestibular nucleus
sptV	spinal tract of the trigeminal nerve
SpV	spinal nucleus of the trigeminal nerve
st	stria terminalis
ST	bed nucleus of stria terminalis
STIA	bed nucleus of stria terminalis, intraamygdaloid division
Sub	submedius thalamic nucleus
SuV	supratrigeminal nucleus

T

Te1	temporal cortex, area 1
Te2d	temporal cortex, area 2, dorsal part
Te2v	temporal cortex, area 2, ventral part
Te3r	temporal cortex, area 3, rostral part
Te3v	temporal cortex, area 3, ventral part
TRN	tegmental reticular nucleus
TS	triangular septal nucleus
Tu	olfactory tubercle
Tz	nucleus of the trapezoid body

V

VA	ventral anterolateral thalamic nucleus
VC	ventral cochlear nucleus
VDB	nucleus of the vertical limb of diagonal band
VEn	ventral endopiriform nucleus
Vhc	ventral hippocampal commissure
VIE	ventral intermediate entorhinal field
VIIn	facial nerve
VIIIn	vestibulocochlear nerve
VL	ventrolateral thalamic nucleus
VLO	orbital cortex, ventrolateral part
VM	ventromedial thalamic nucleus
Vma	motor nucleus of the trigeminal nucleus, magnocellular part
VMH	ventromedial hypothalamic nucleus
Vn	trigeminal nerve
VO	orbital cortex, ventrol part
VP	ventral pallidum
VPL	ventral posterorolateral thalamic nucleus
VPM	ventral posteromedial thalamic nucleus
vsc	ventral spinocerebellar tract
VTN	ventral tegmental nucleus
VTT	ventral tenia tecta

X

XII	Hypoglossal nucleus

Z

ZI	zona incerta

All sections are 20µm thick. Distance between sections is given by the difference between section numbers.

In muscarinic cholinergic M_2 receptor autoradiographs:

- Color scale indicates receptor densities in fmol/mg protein
- Dashed lines indicate somatotopic subdivisions within area Par1
- Dotted lines or asterisks mark sectioning/binding artifacts

Coronal series

Level c1

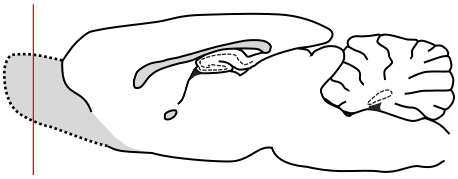

Section 118

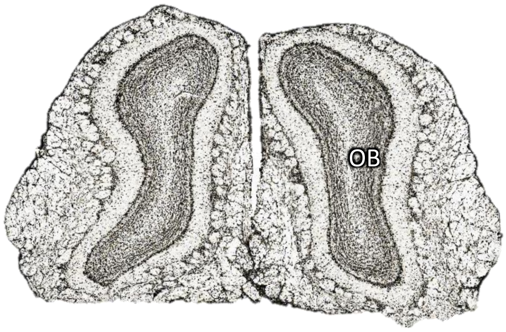

500µm

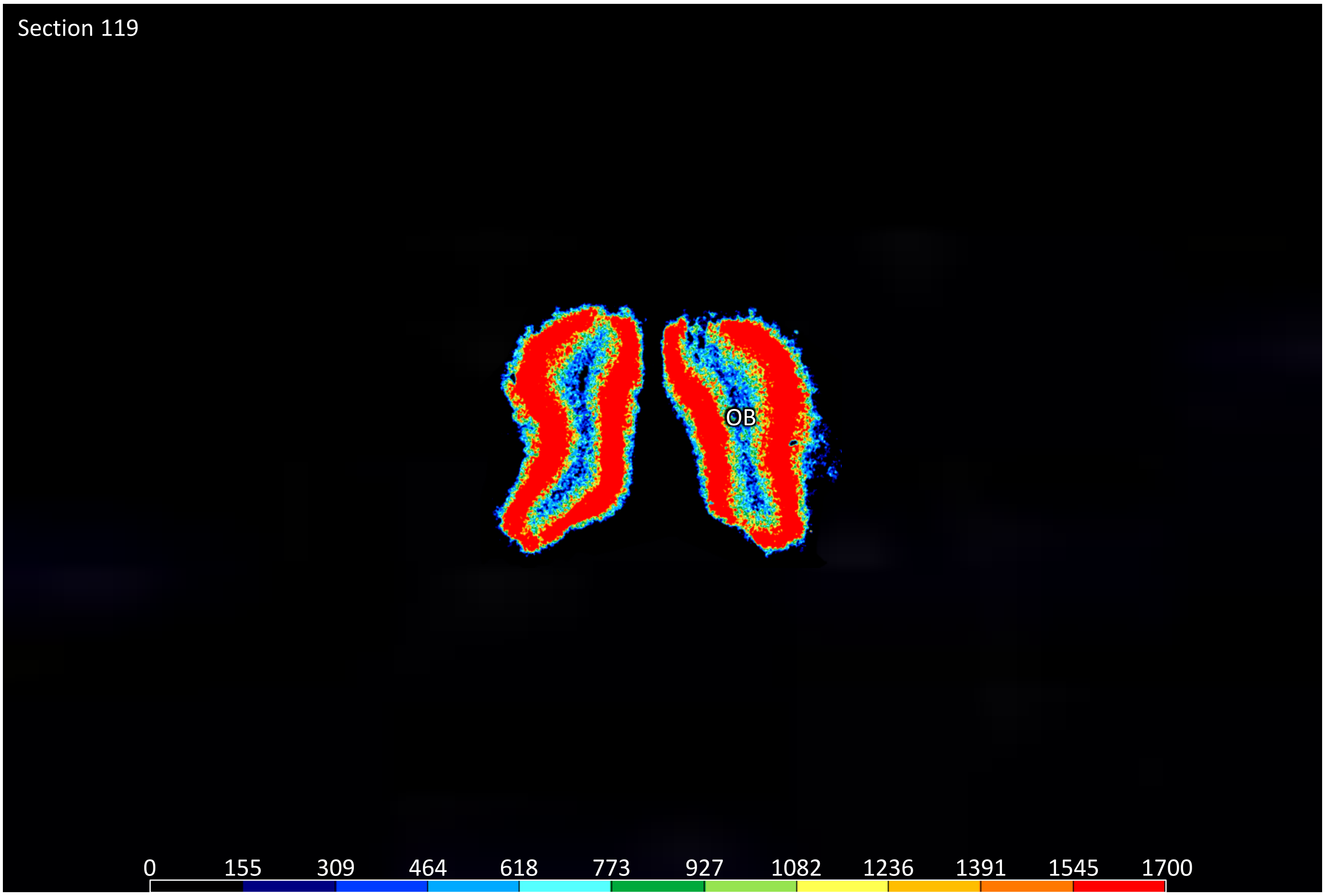

Level c2

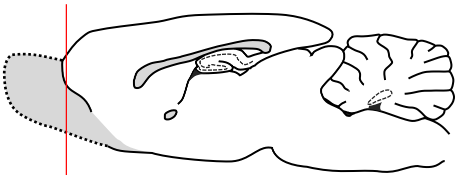

Section 268

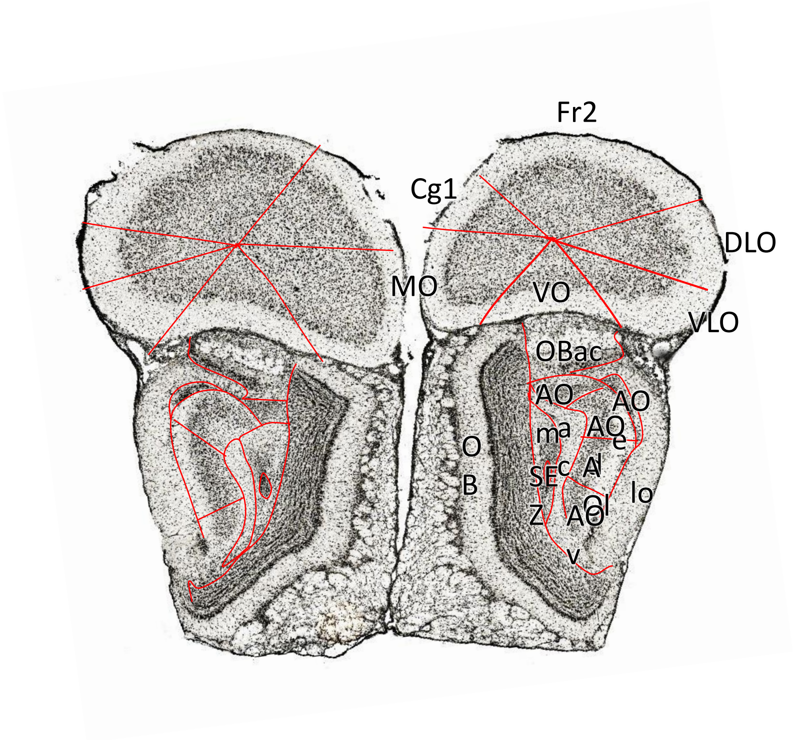

500µm

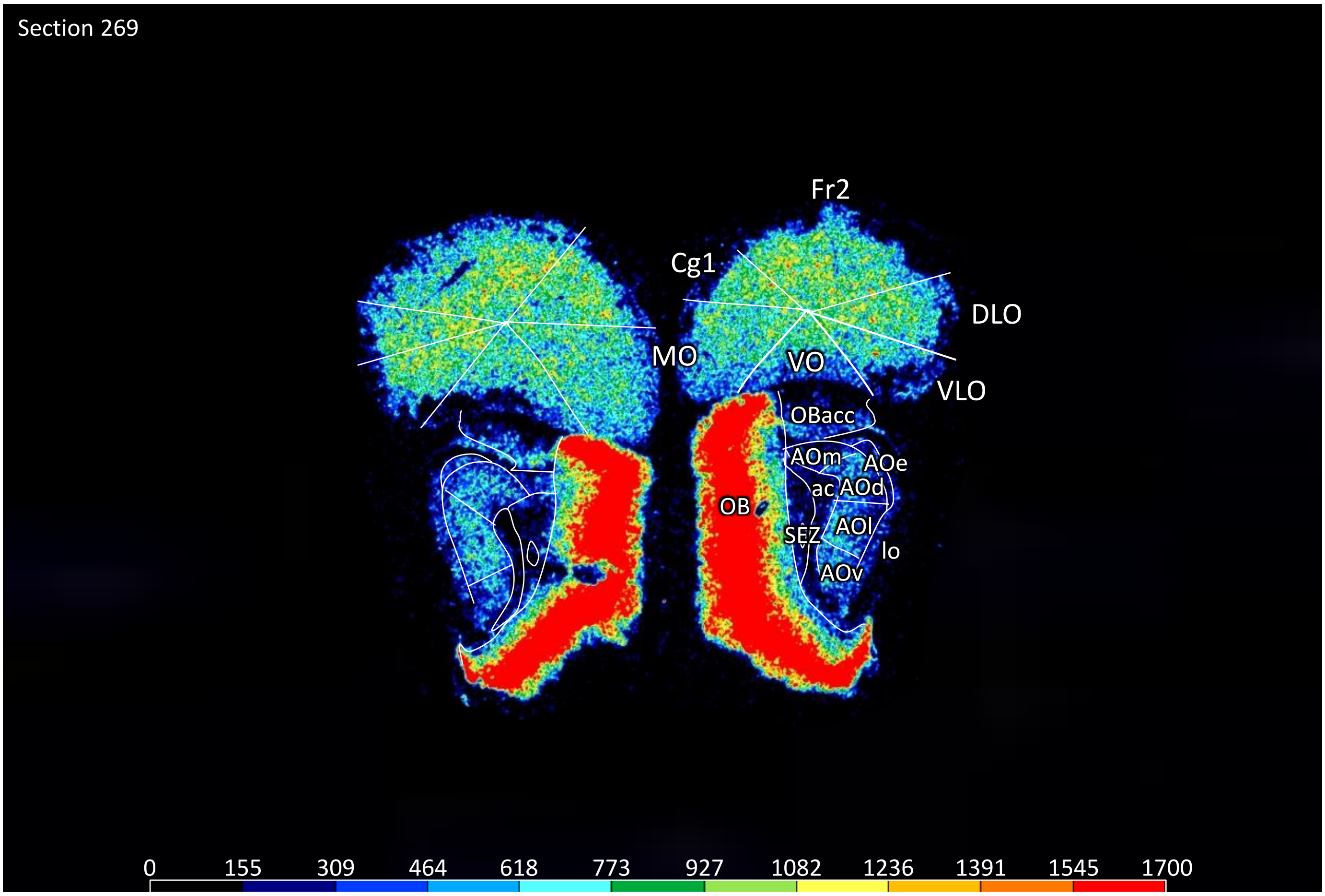

Level c3

Section 313

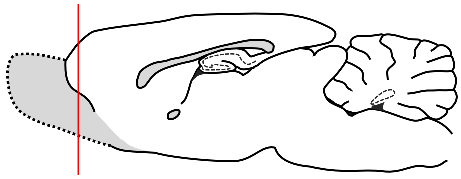

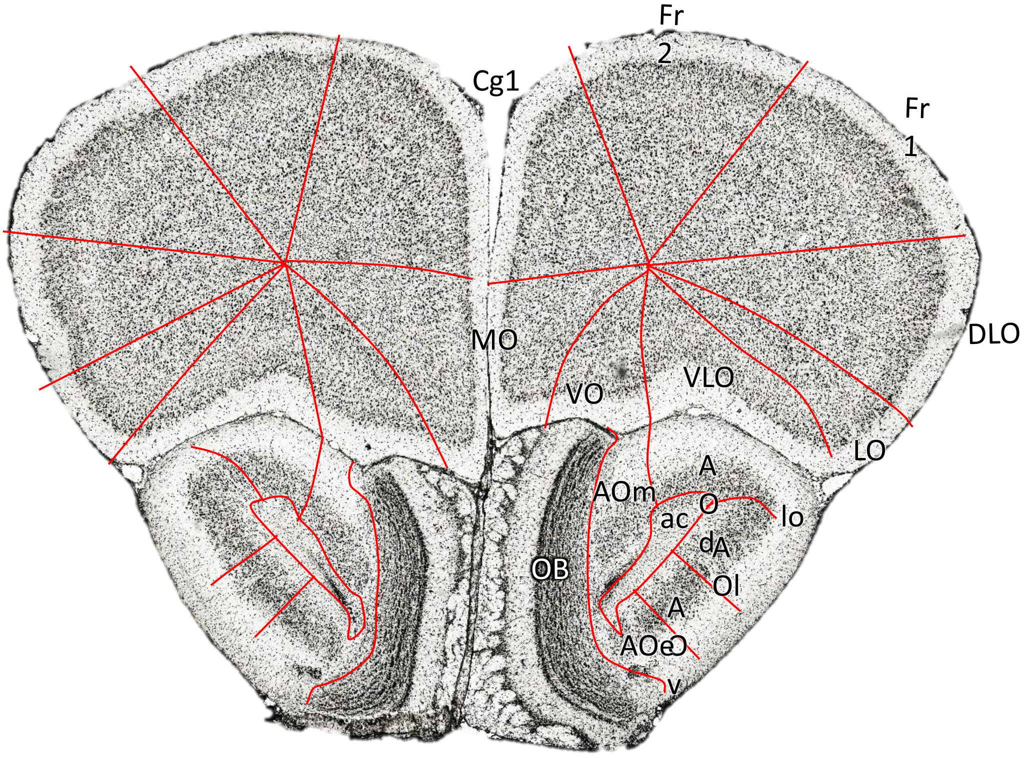

500µm

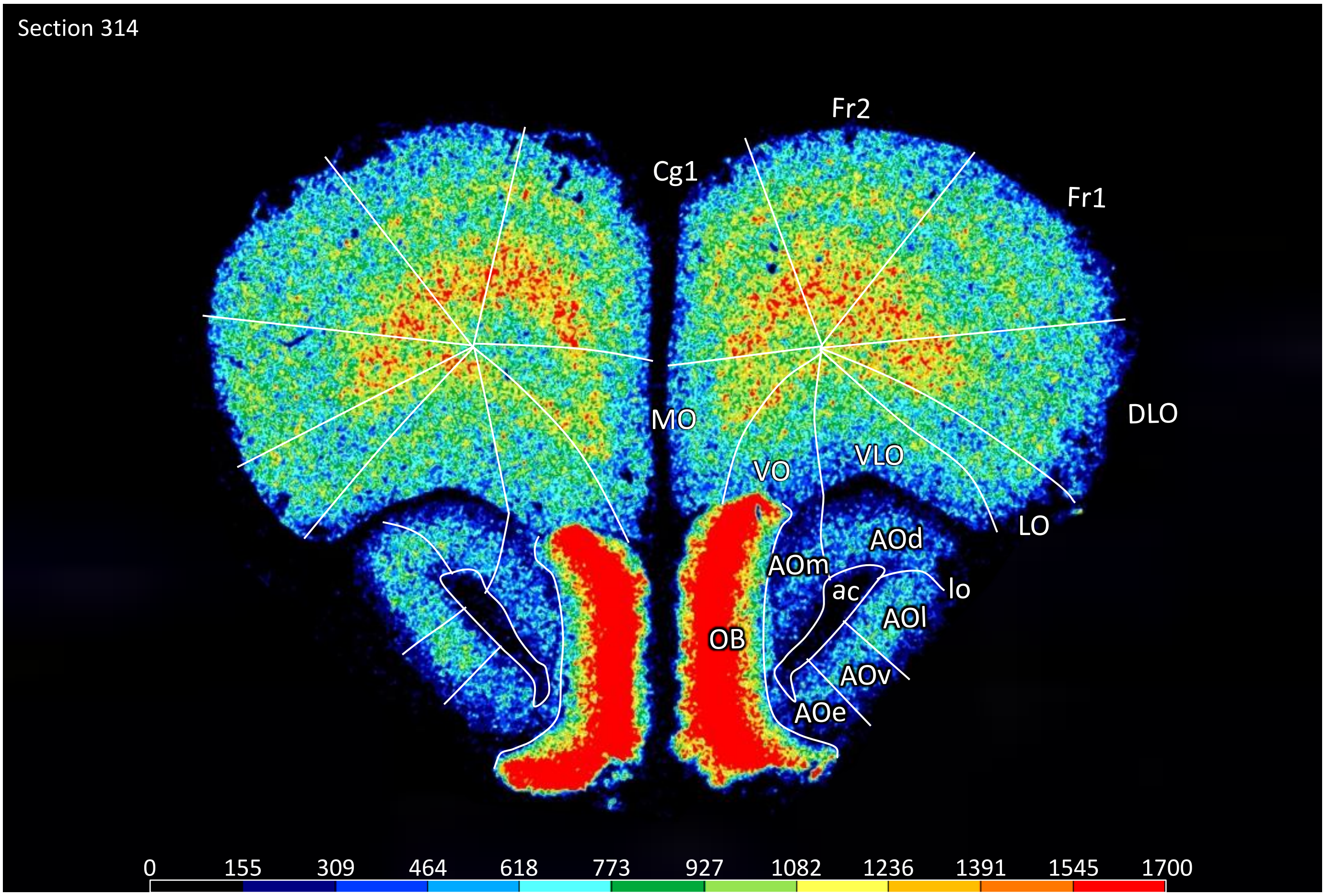

Level c4

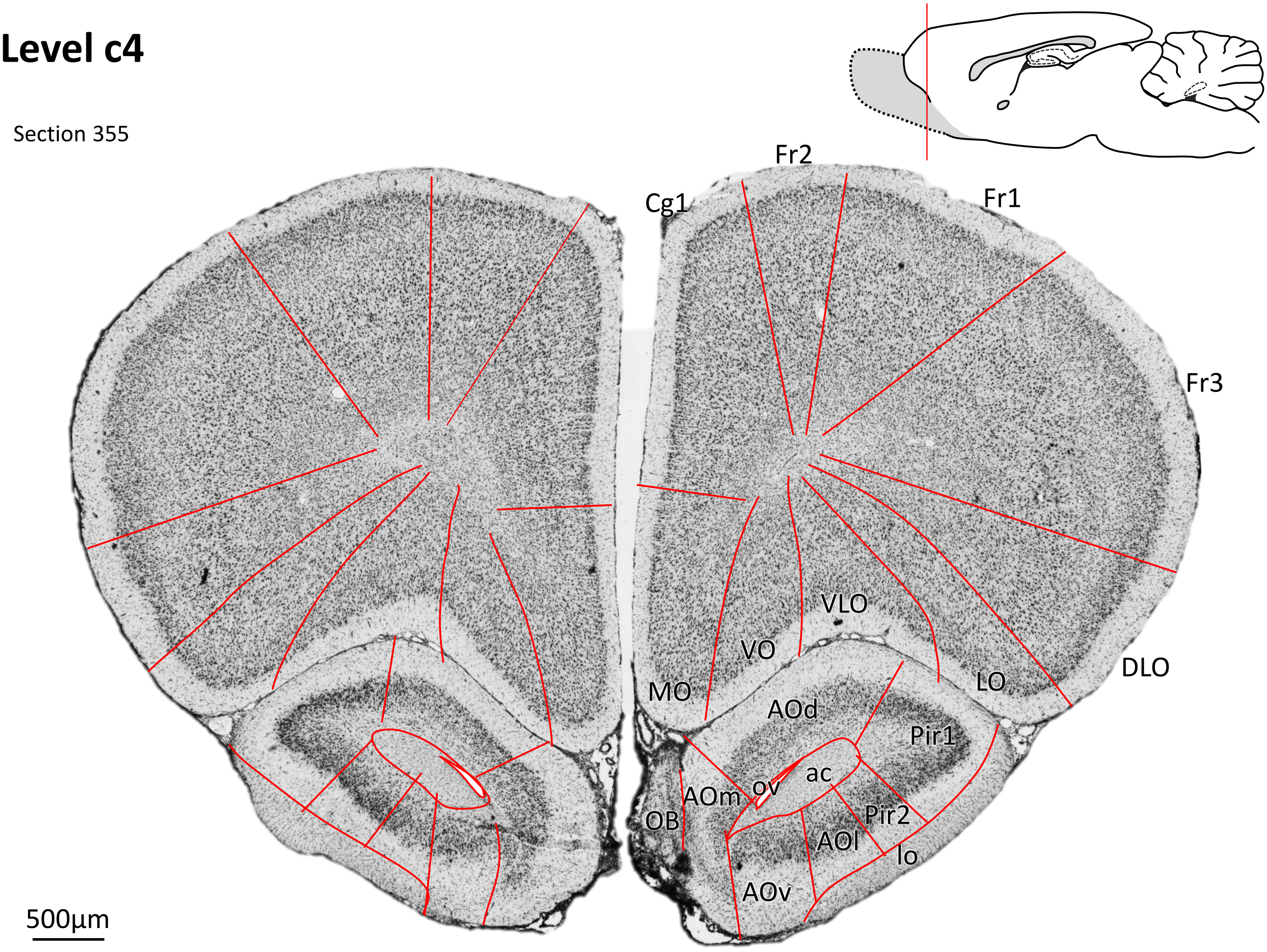

Section 355
Fr2
Cg1
Fr1
Fr3
VLO
VO
MO
AOd
LO
DLO
Pir1
ac
ov
AOm
OB
Pir2
AOl
lo
AOv
500µm

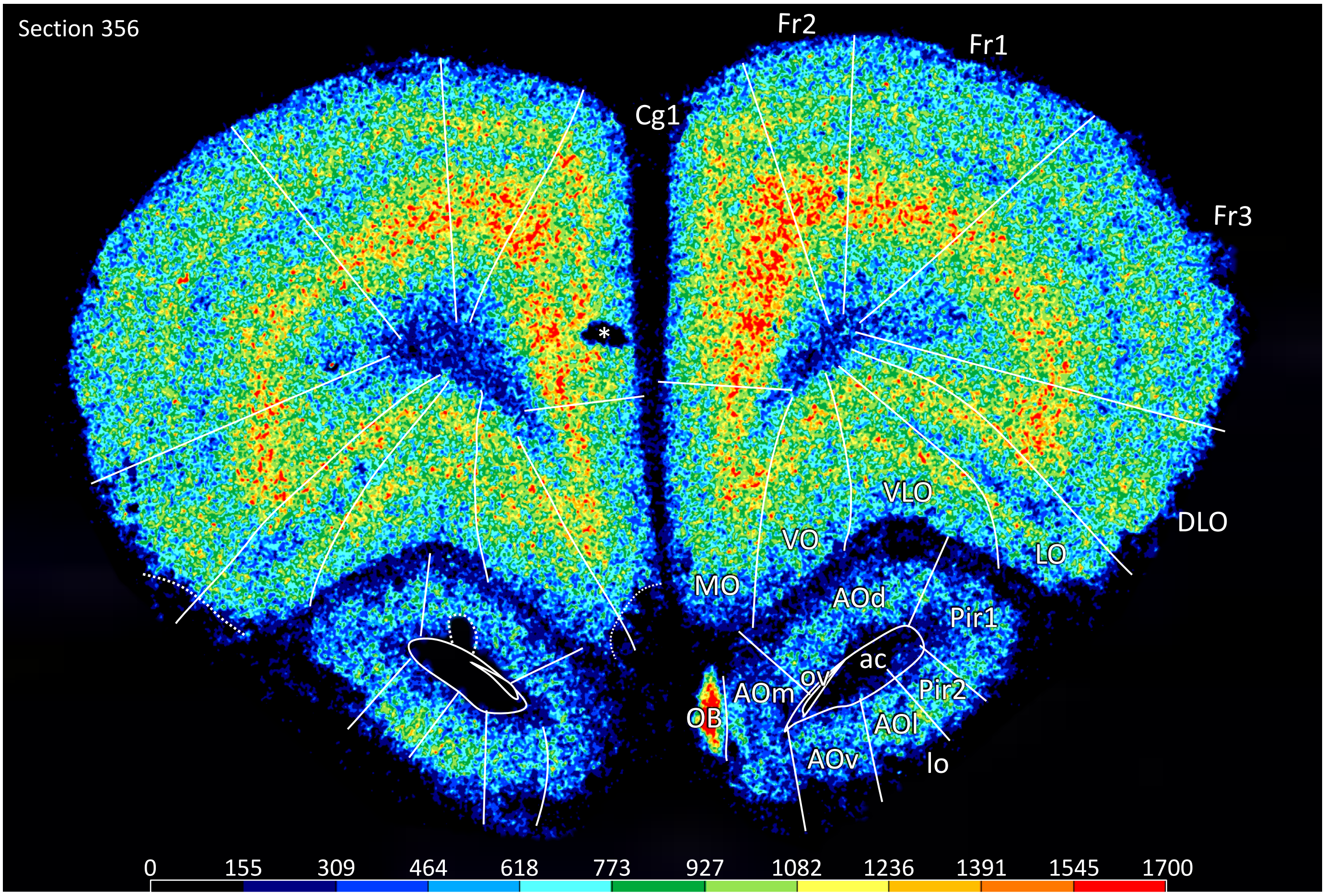

Section 356
Fr2
Fr1
Cg1
Fr3
VLO
VO
DLO
LO
MO
AOd
Pir1
ac
ov
AOm
Pir2
OB
AOl
AOv
lo
0
155
309
464
618
773
927
1082
1236
1391
1545
1700

Level c5

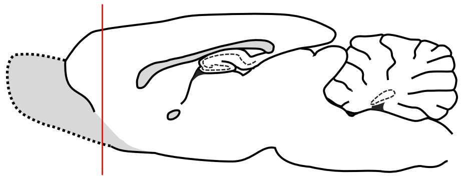

Section 370

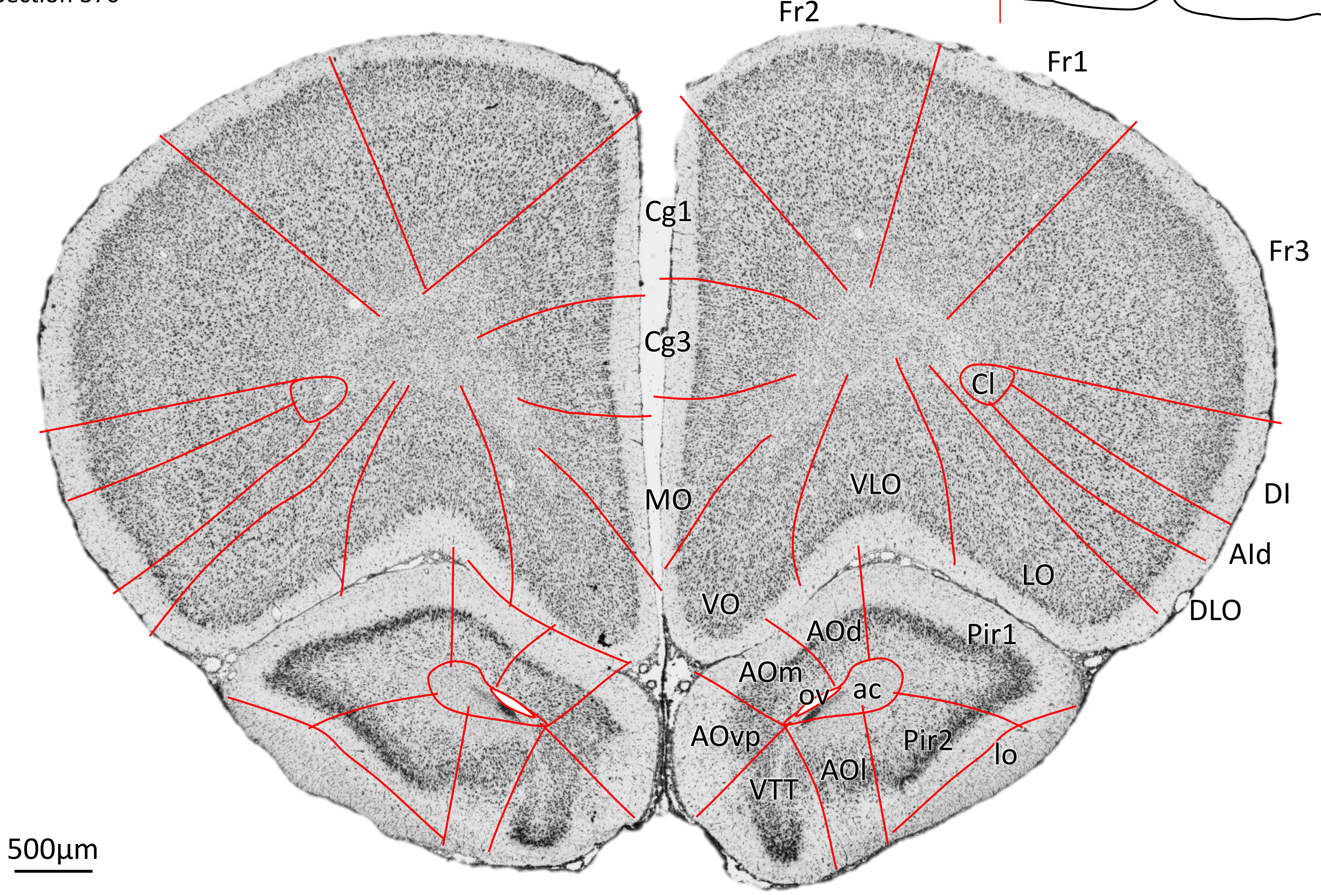

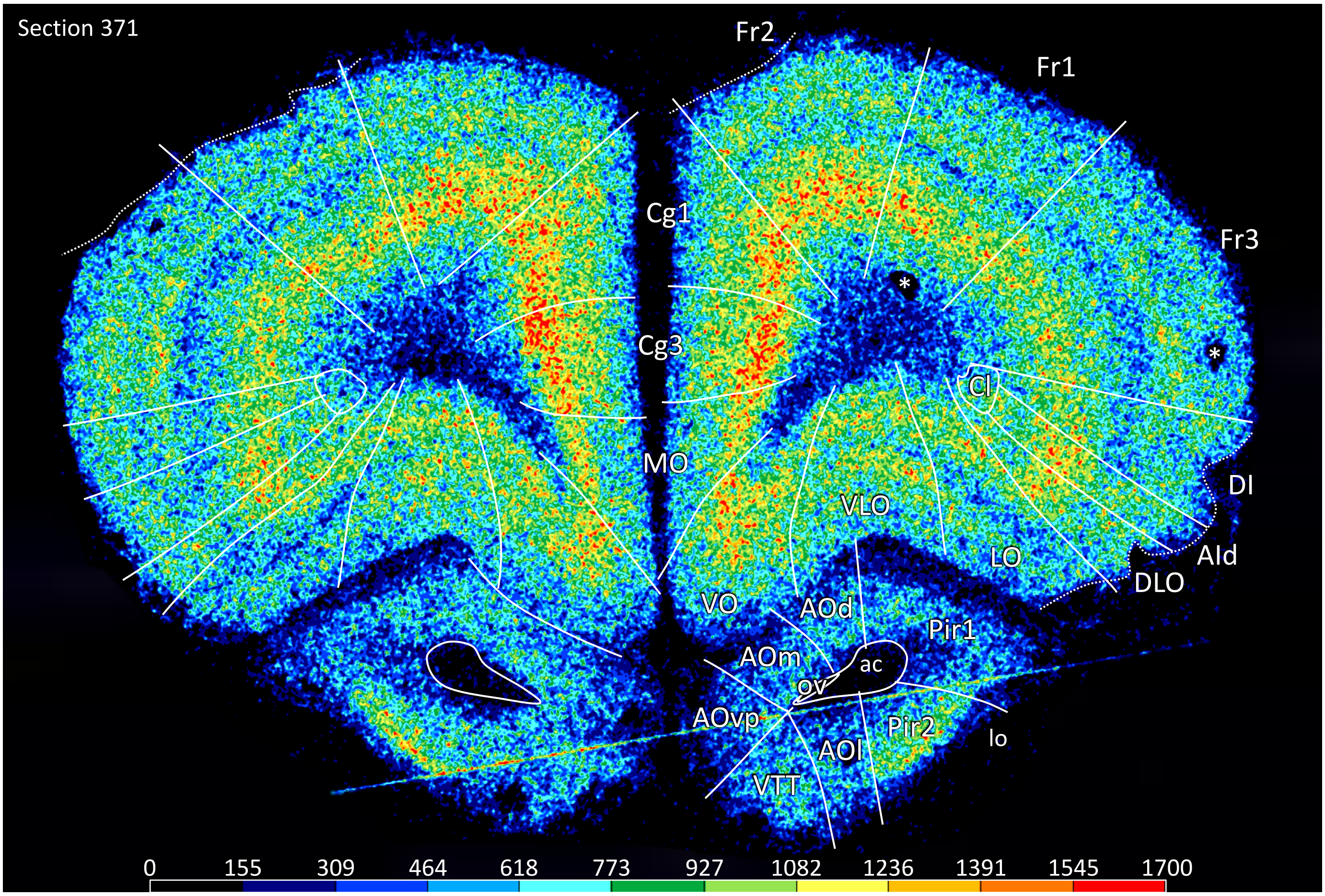

Level c6

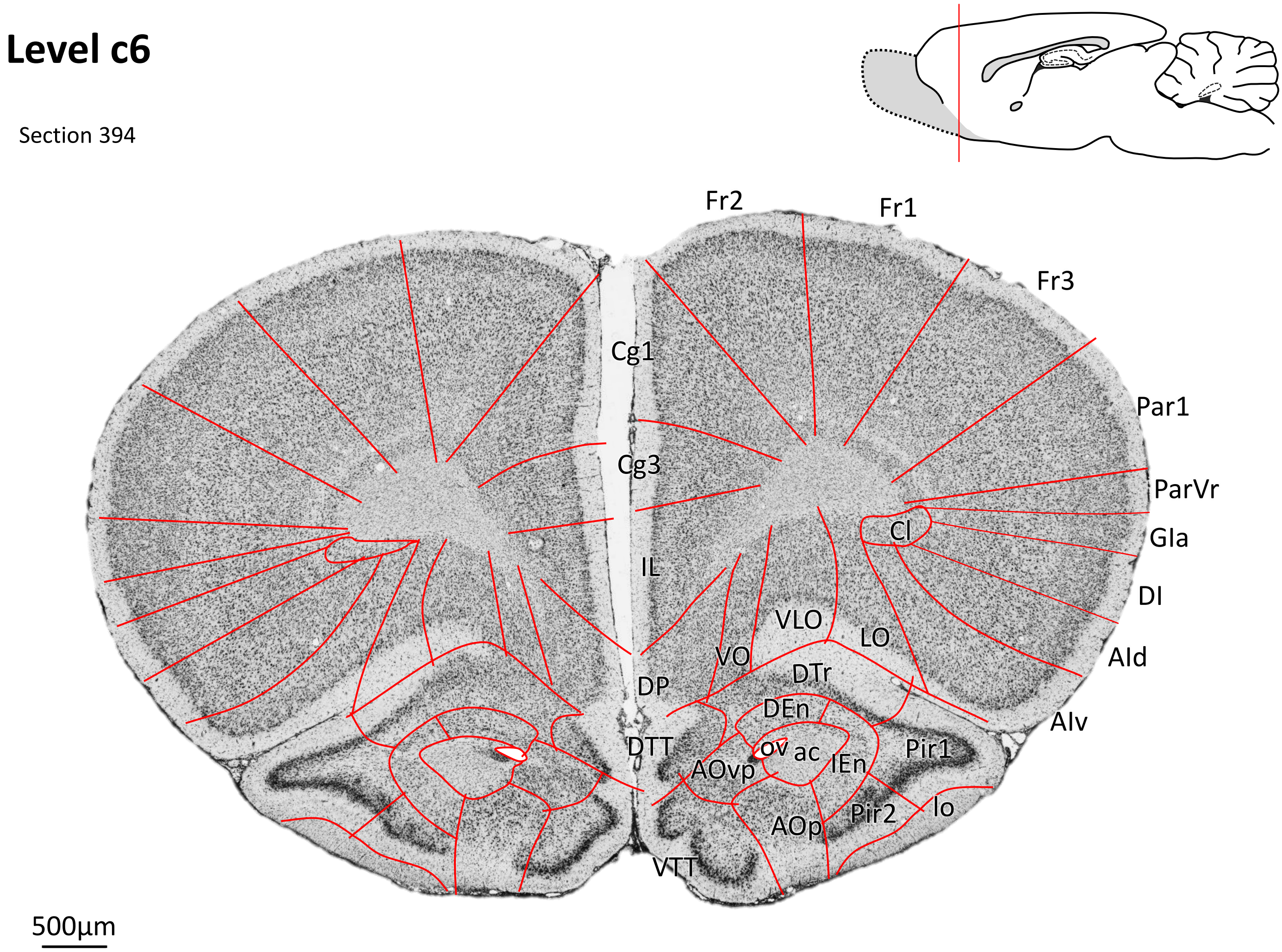

Section 394
Fr2
Fr1
Fr3
Cg1
Par1
Cg3
ParVr
Cl
Gla
IL
DI
VLO
LO
VO
AId
DP
DTr
DEn
AIv
DTT
ov
ac
IEn
Pir1
AOvp
AOp
Pir2
lo
VTT
500µm

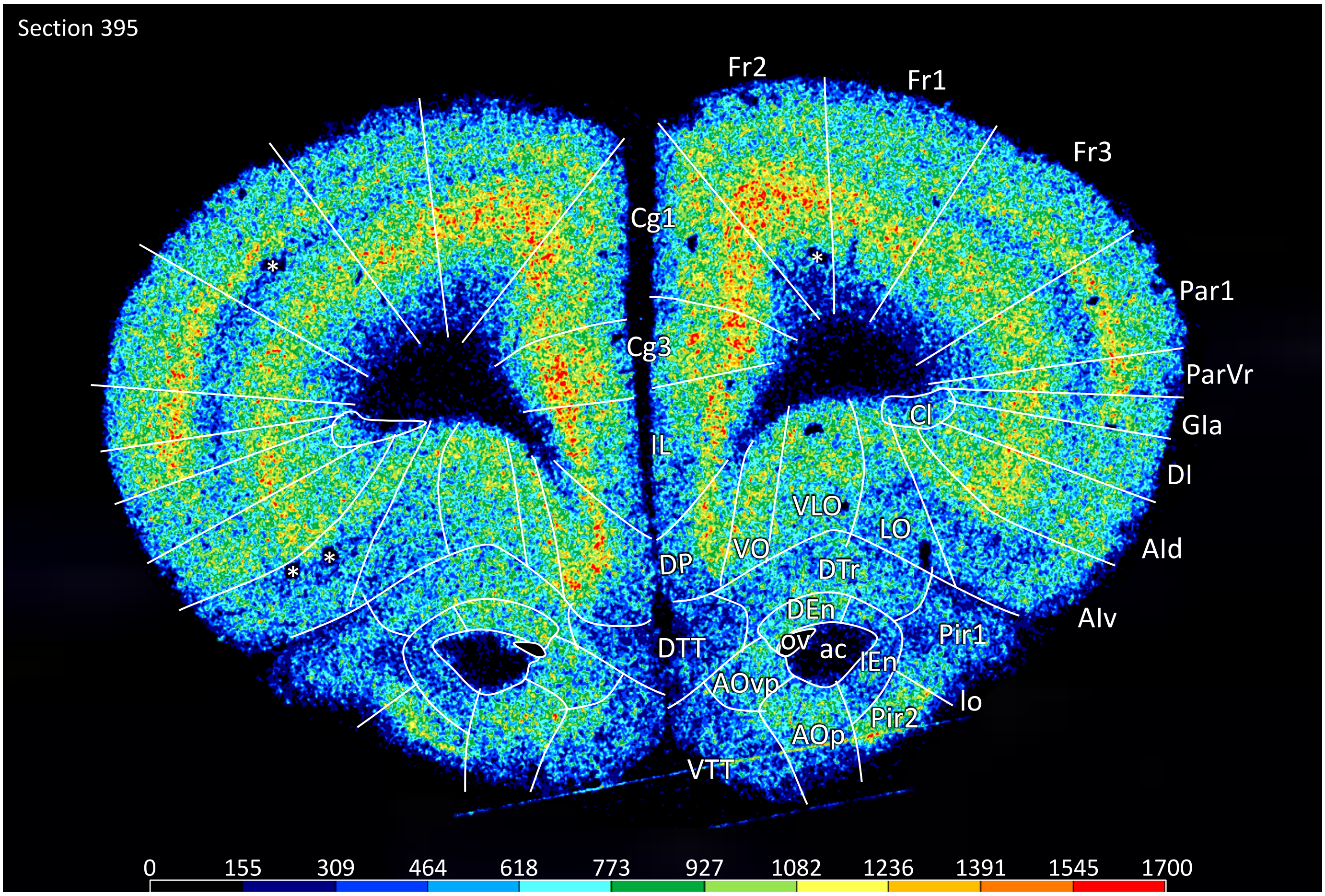

Section 395
Fr2
Fr1
Fr3
Cg1
Par1
Cg3
ParVr
Cl
Gla
IL
DI
VLO
LO
VO
DP
AId
DTr
DEn
AIv
DTT
ov
ac
IEn
Pir1
AOvp
lo
Pir2
AOp
VTT
0
155
309
464
618
773
927
1082
1236
1391
1545
1700

Level c7

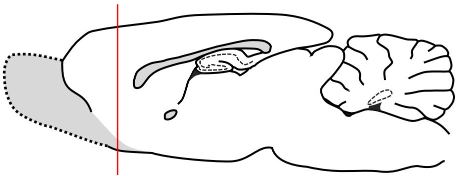

Section 418

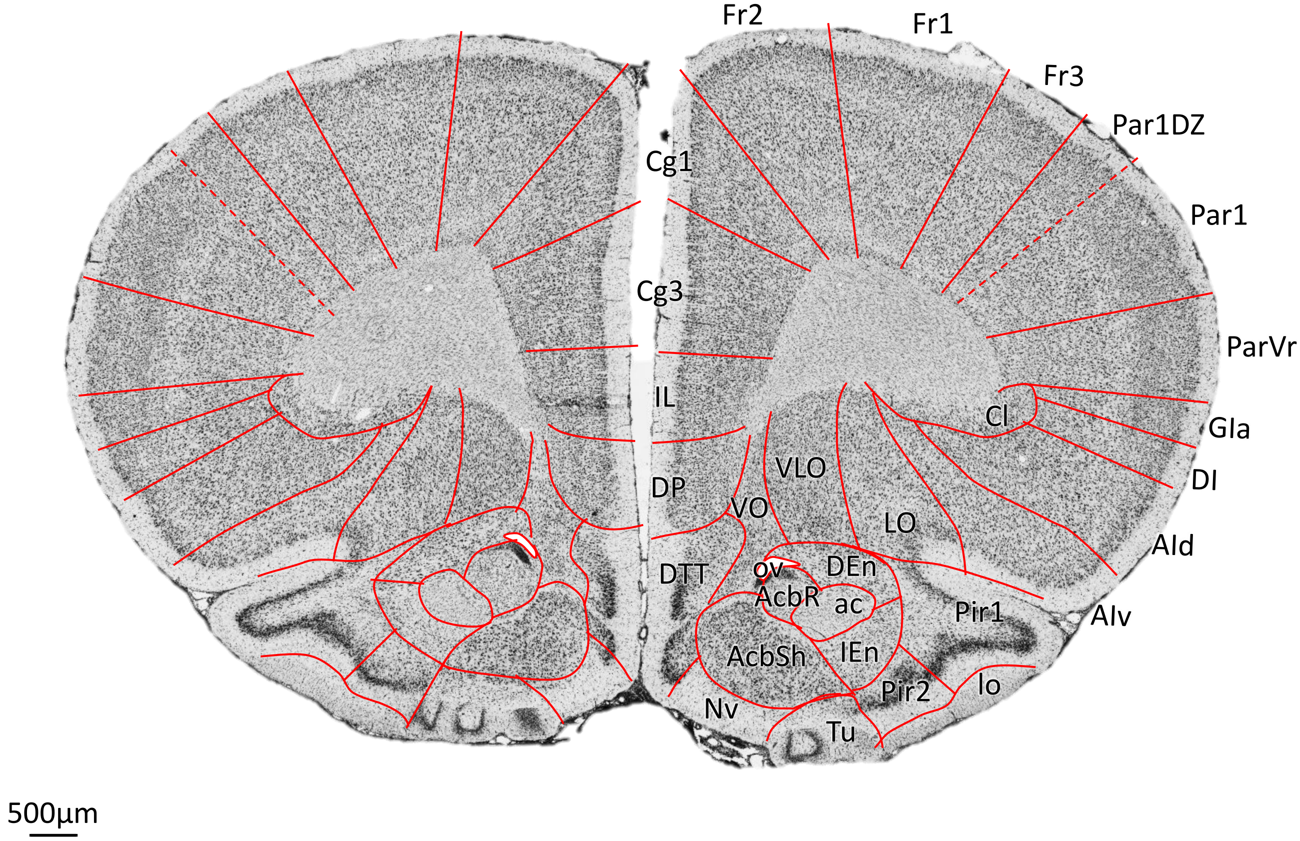

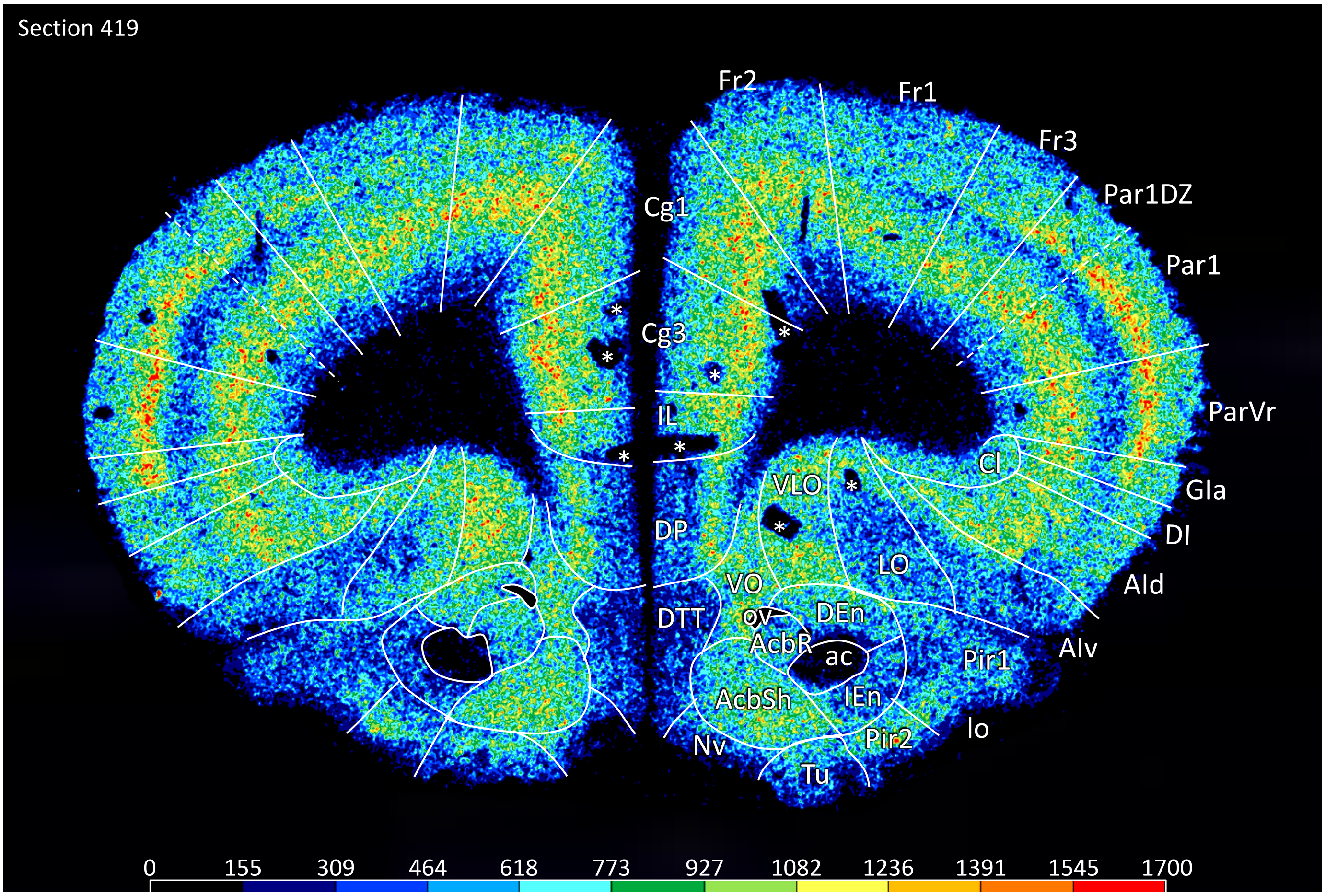

Level c8

Section 442

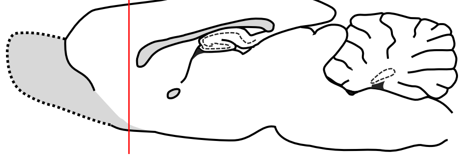

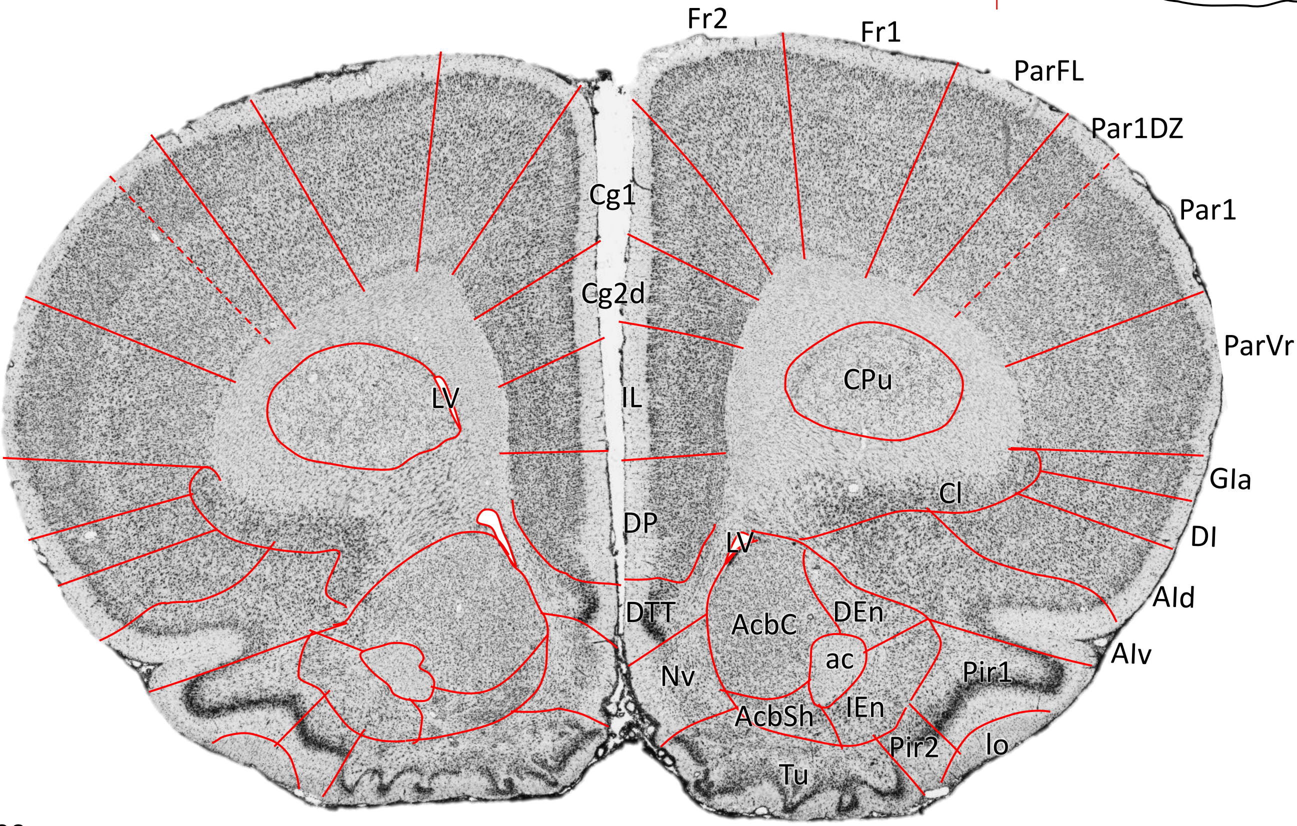

500µm

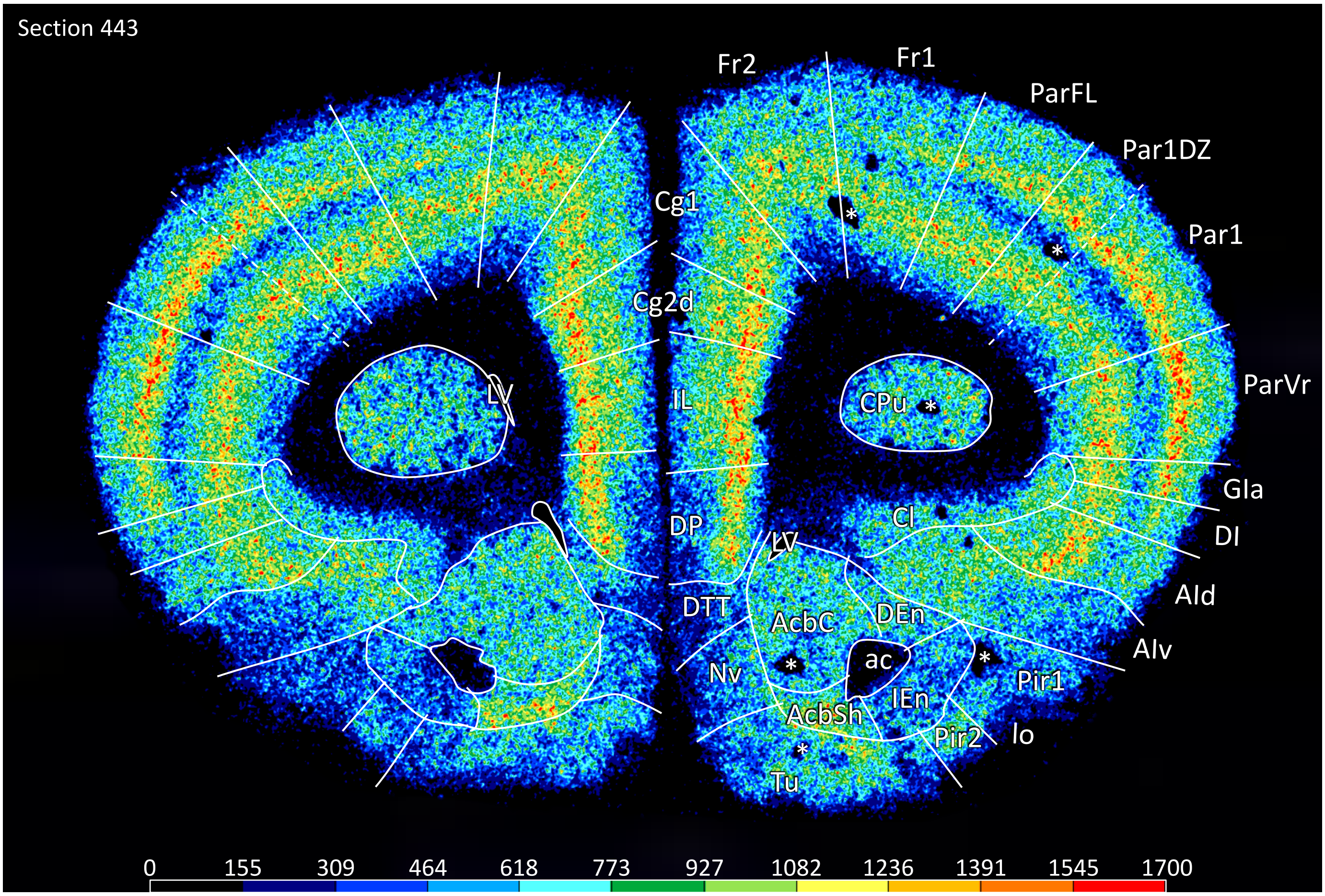

Level c9

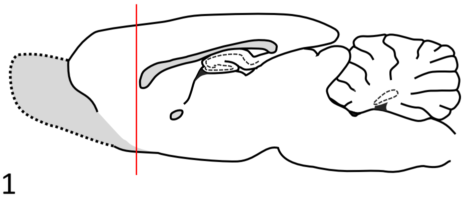

Section 454

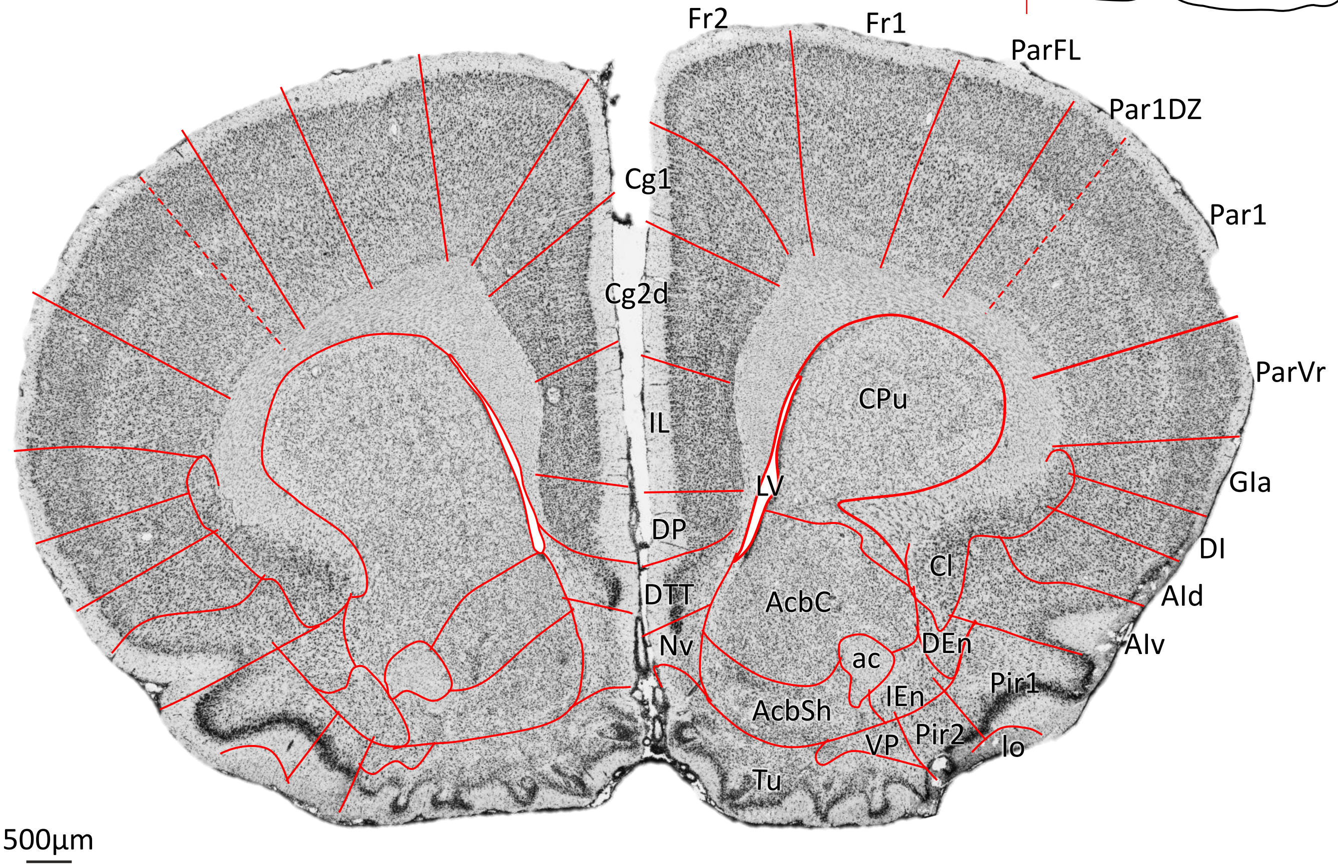

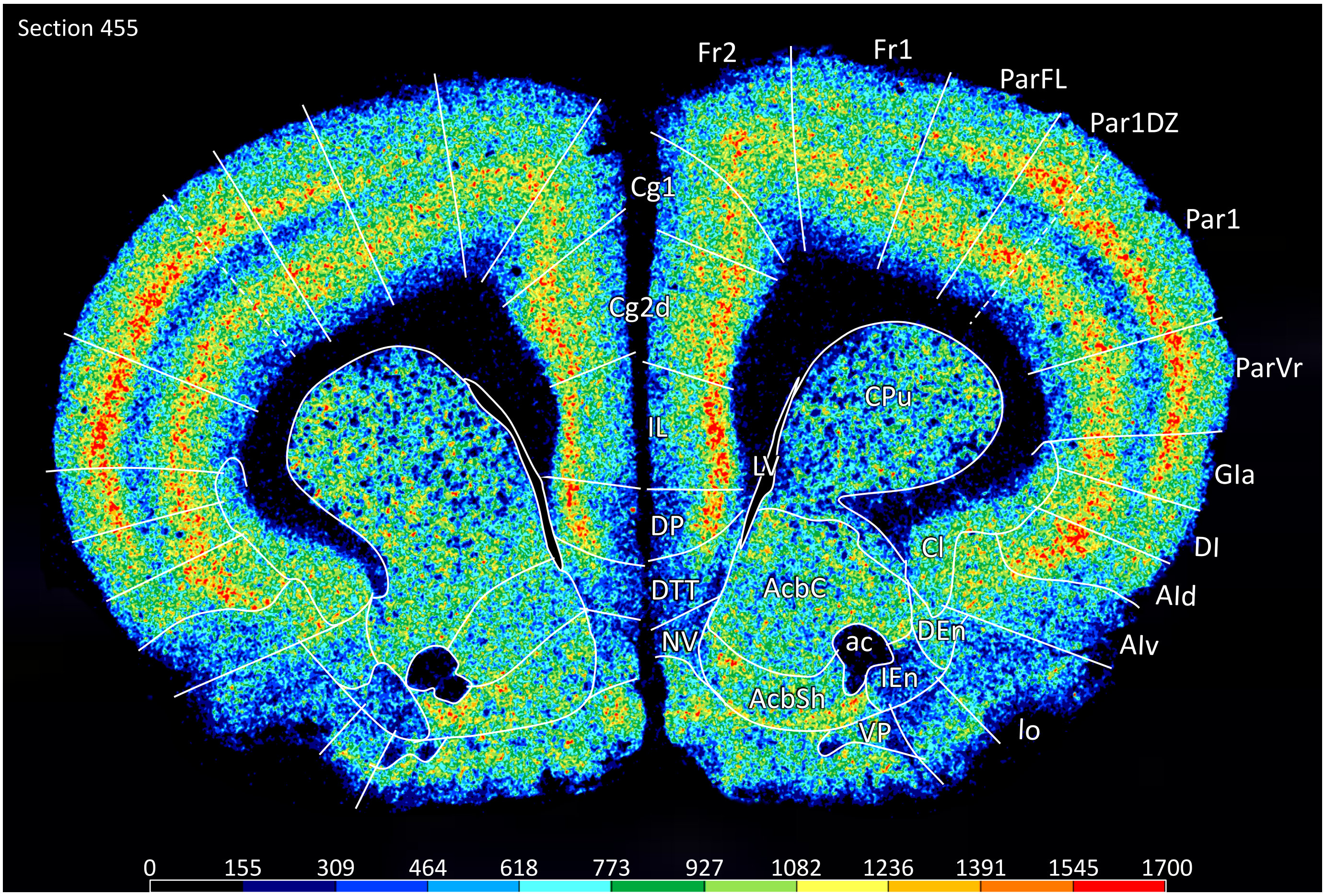

Level c10

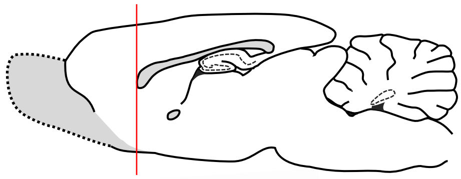

Section 460

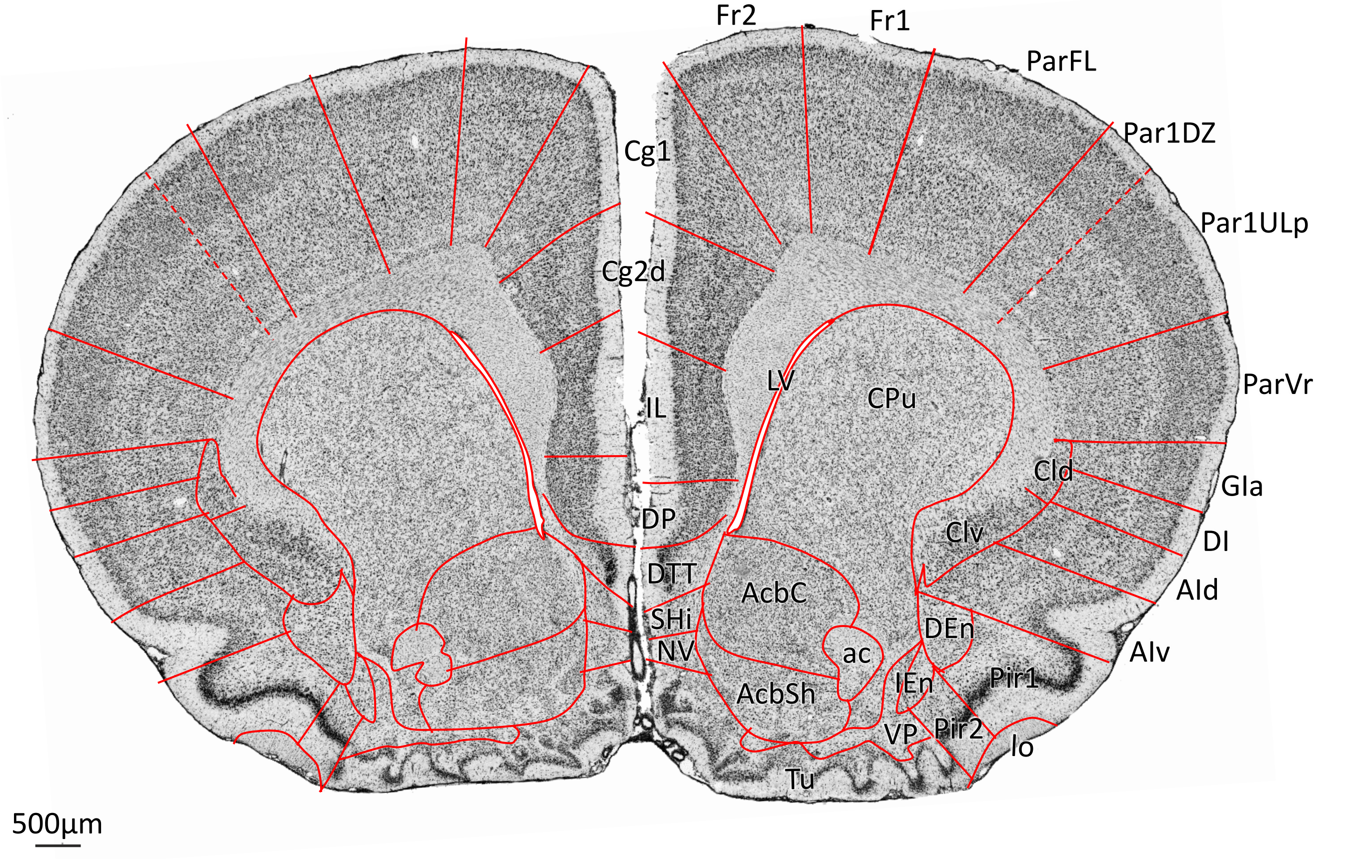

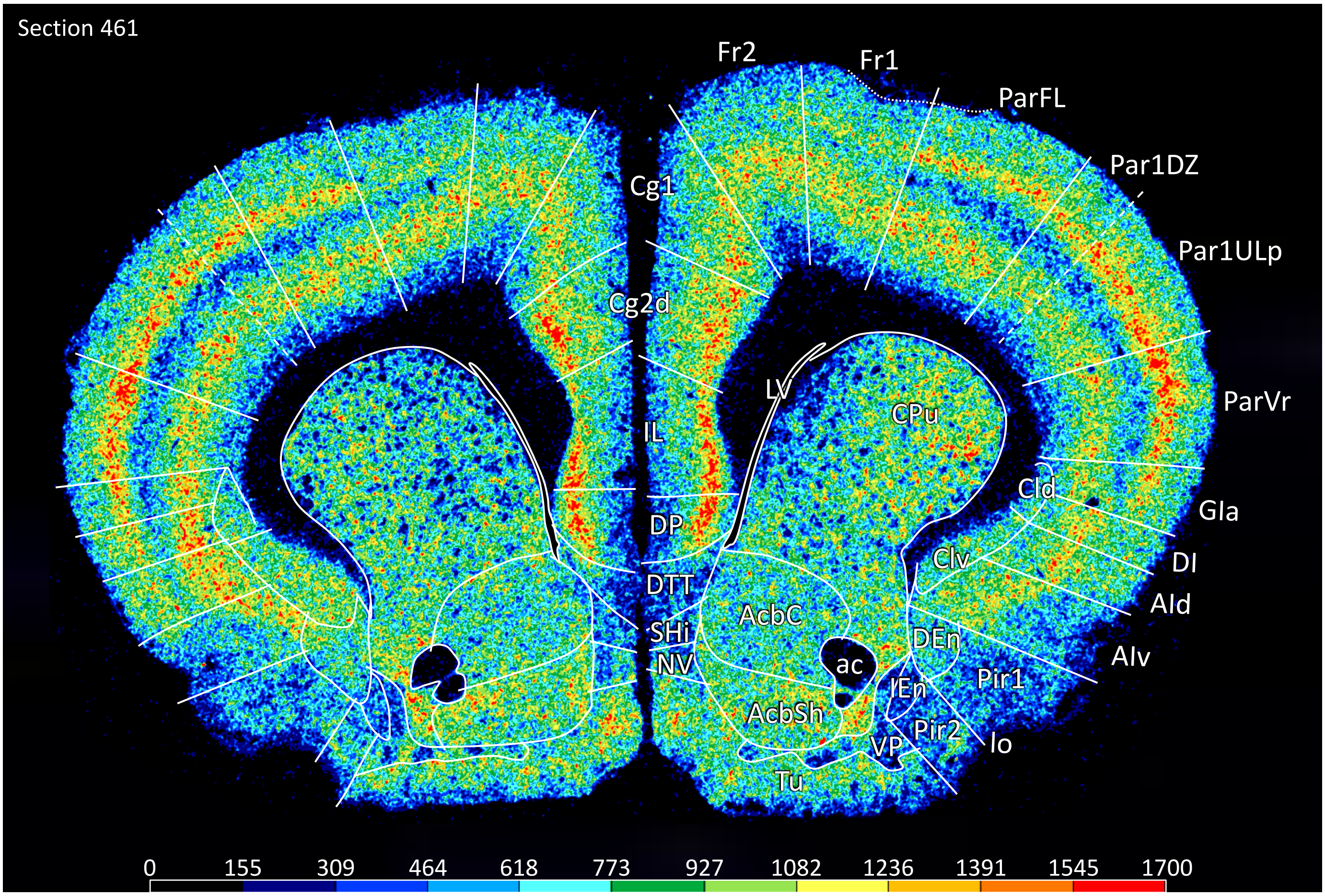

Level c11

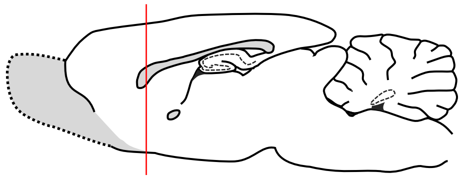

Section 490

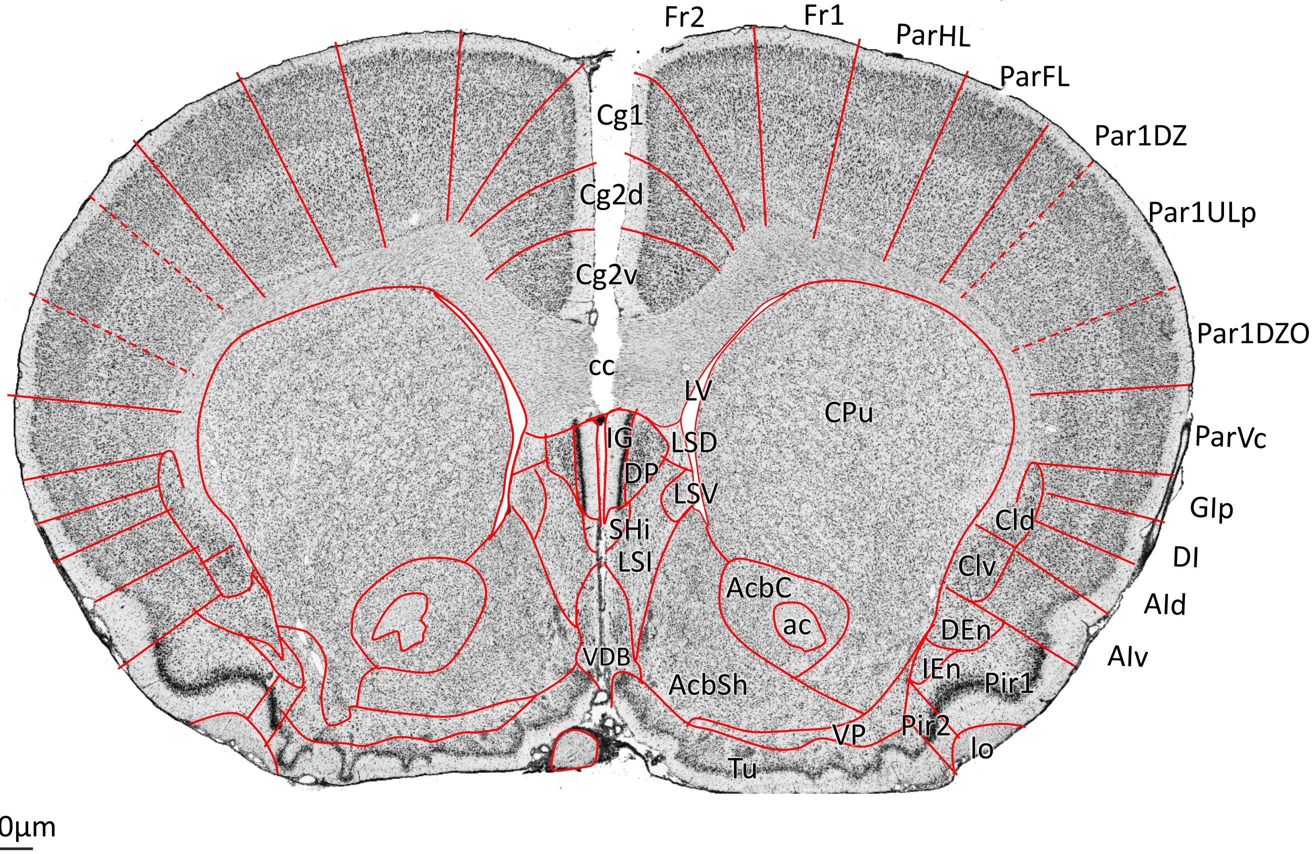

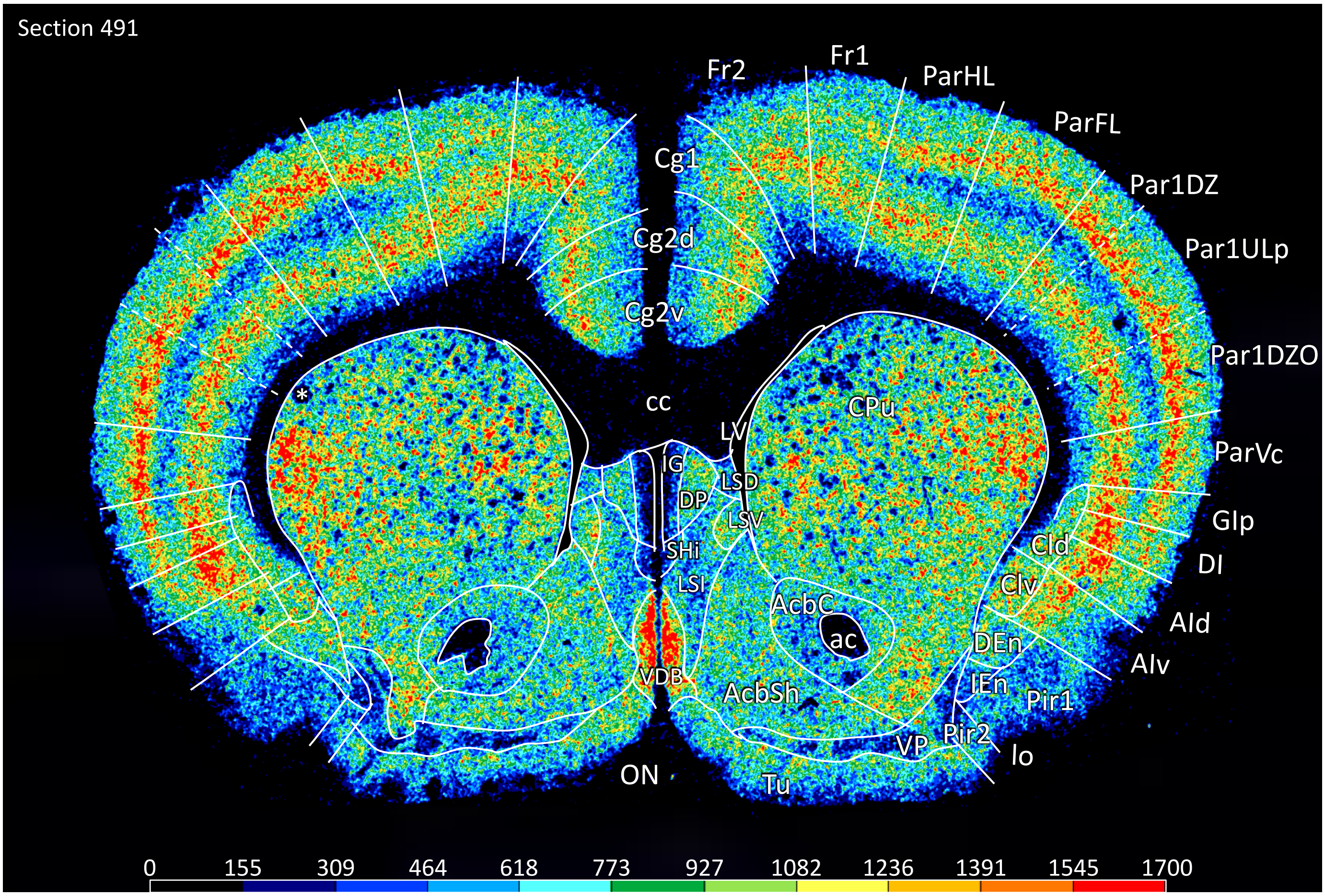

Level c12

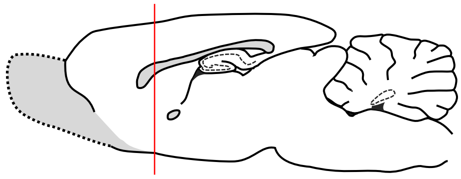

Section 514

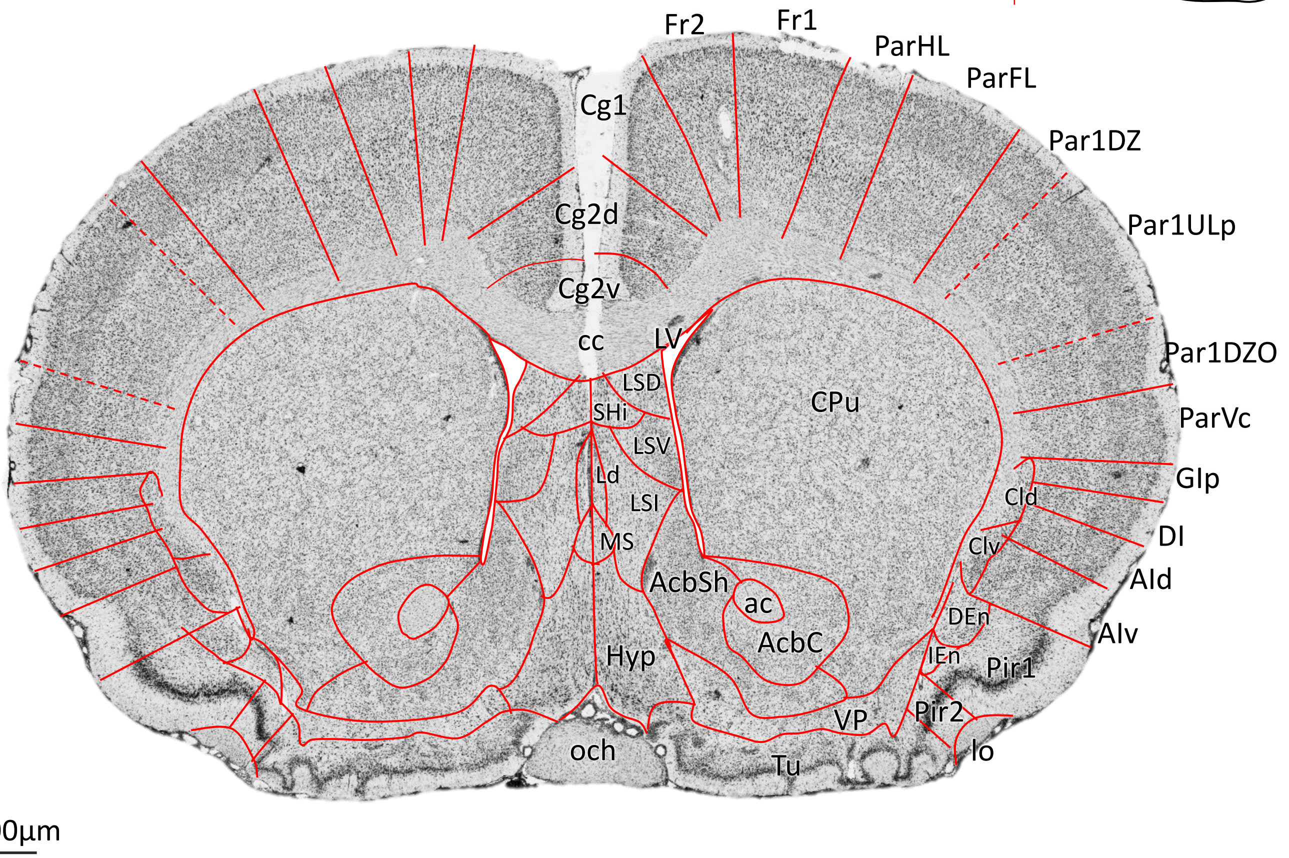

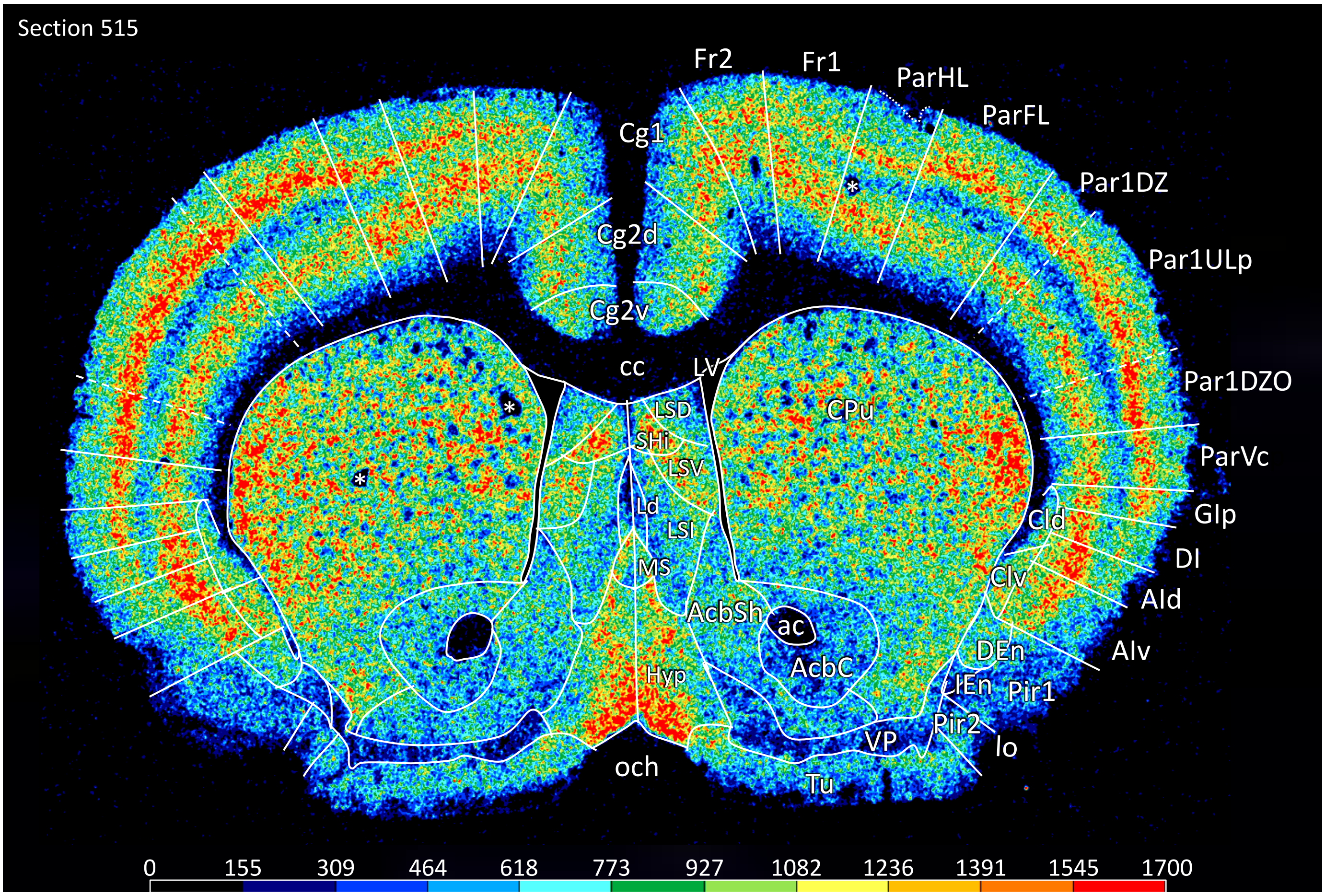

Level c13

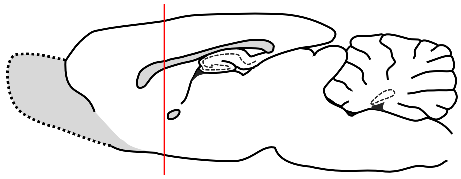

Section 538

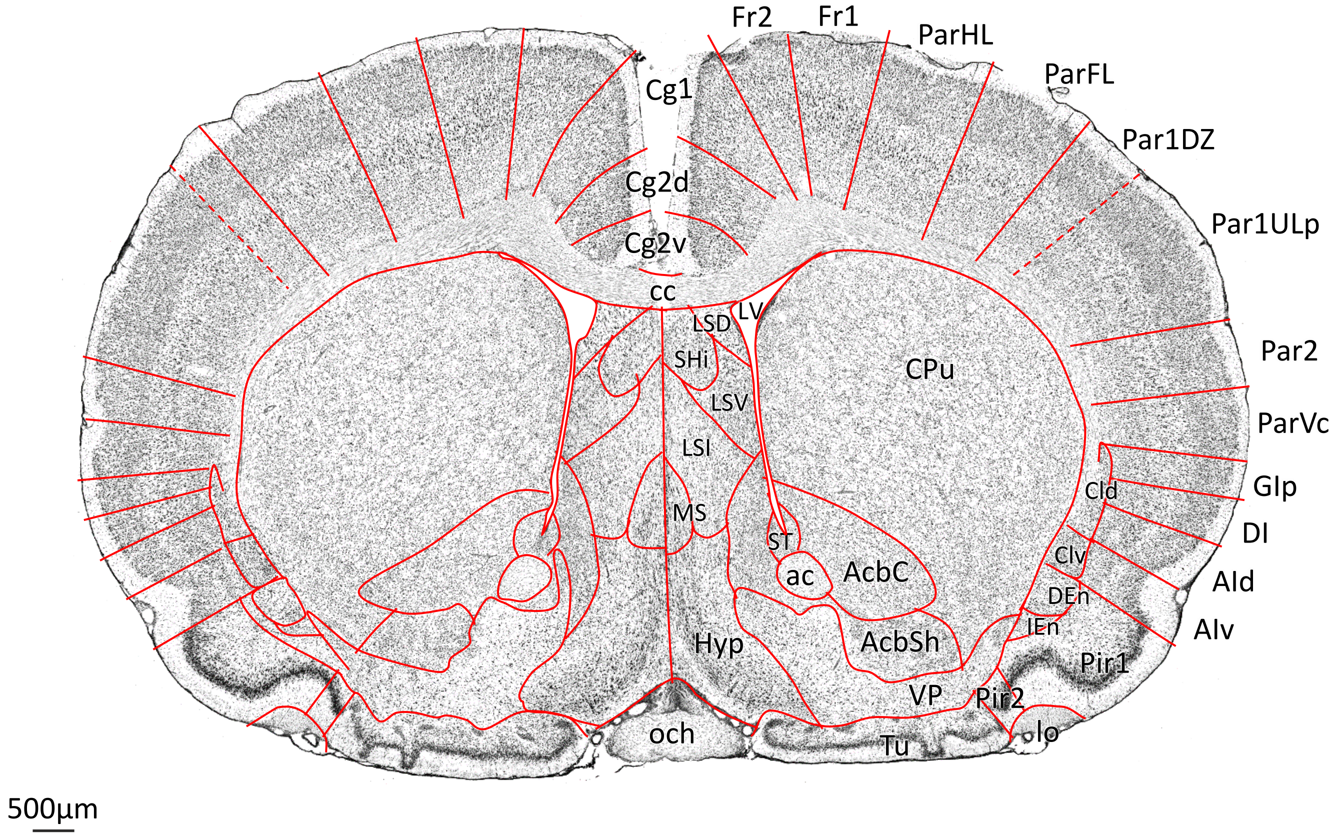

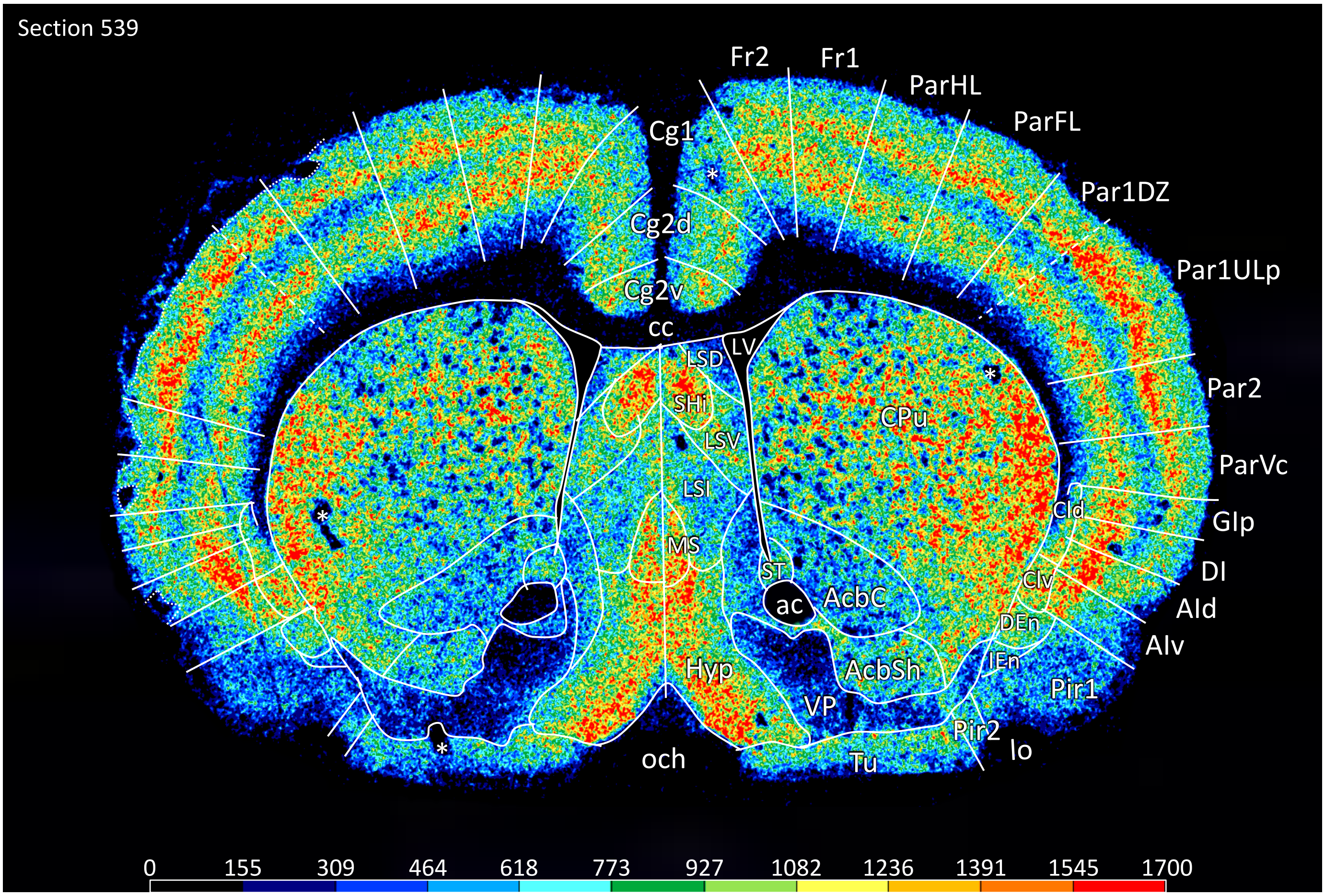

Level c14

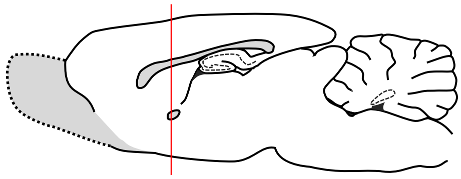

Section 562

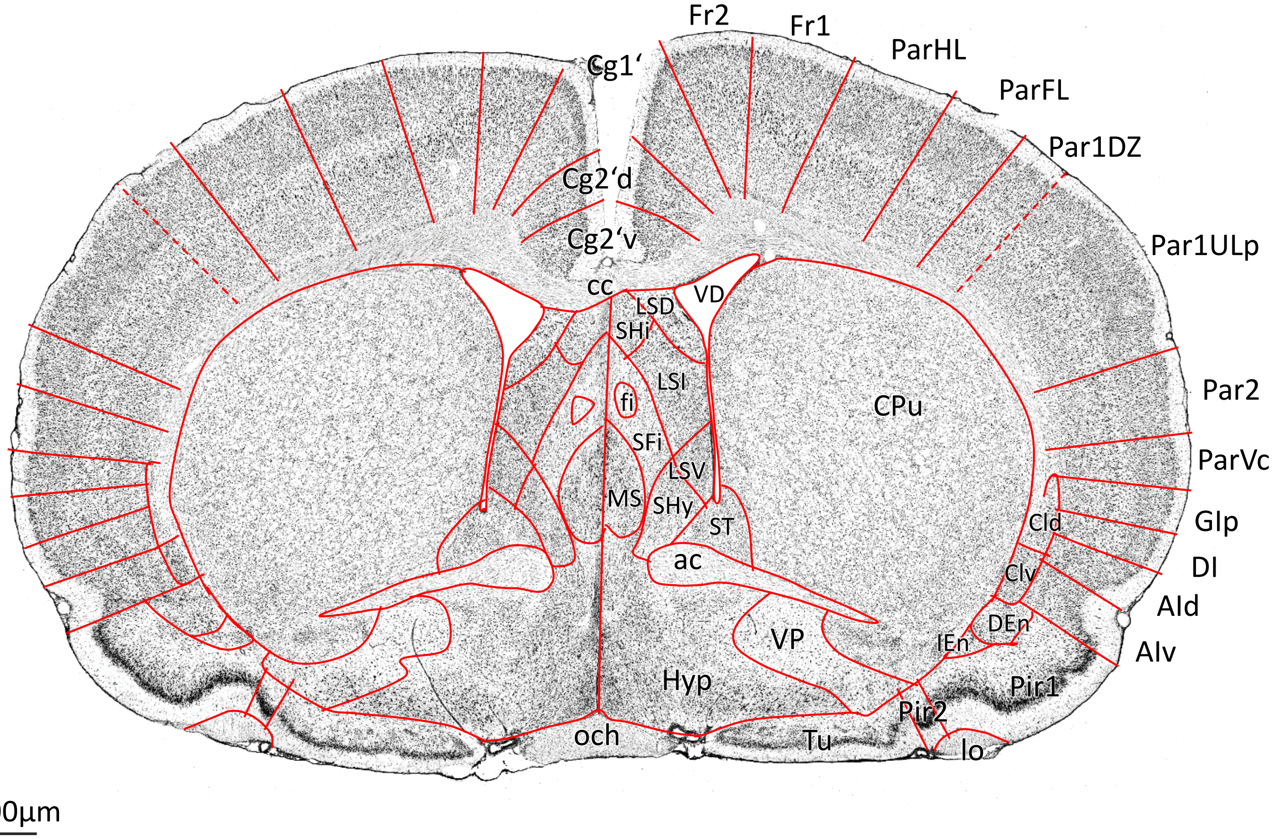

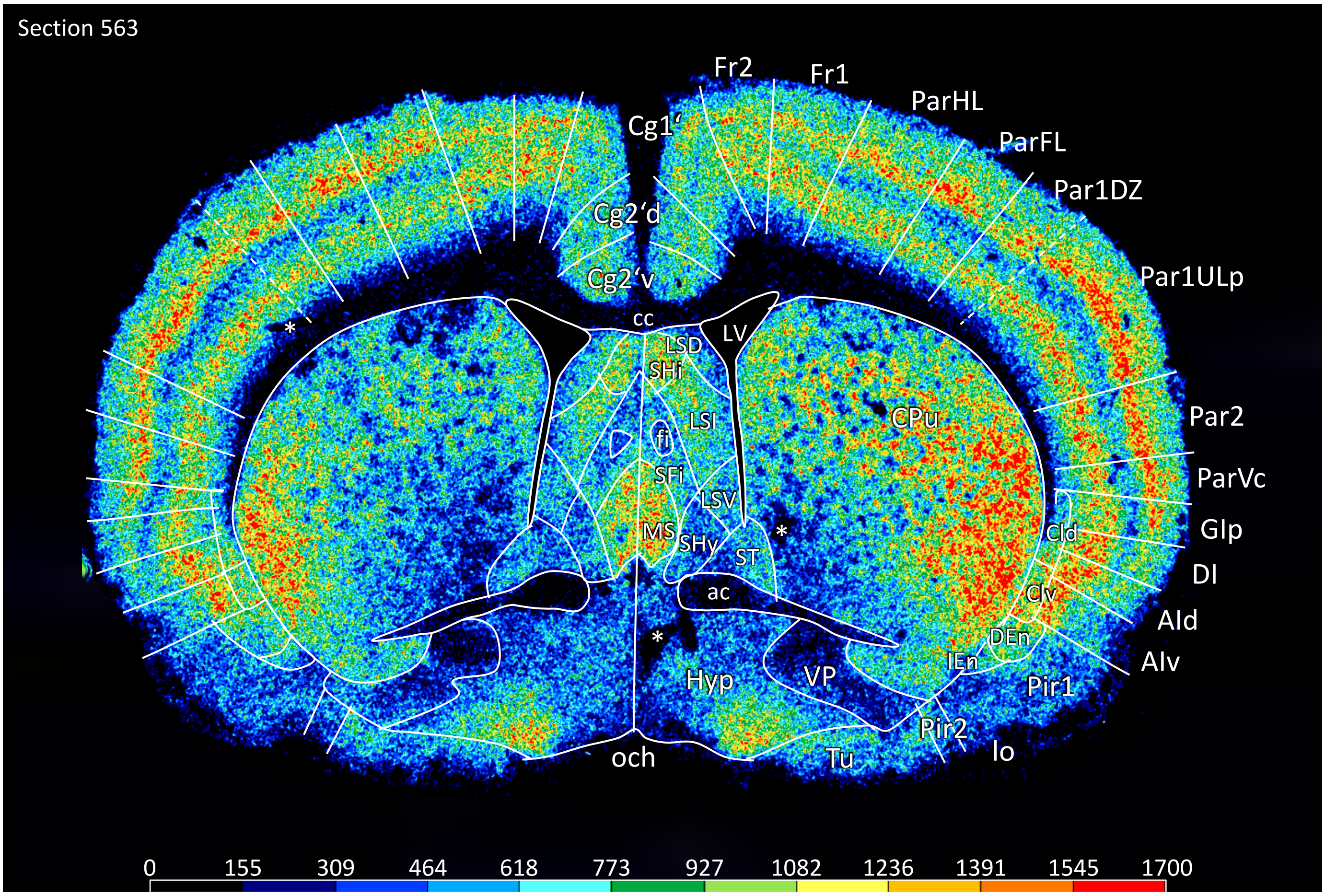

Level c15

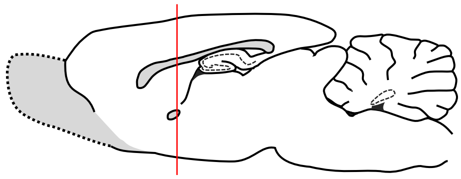

Section 574

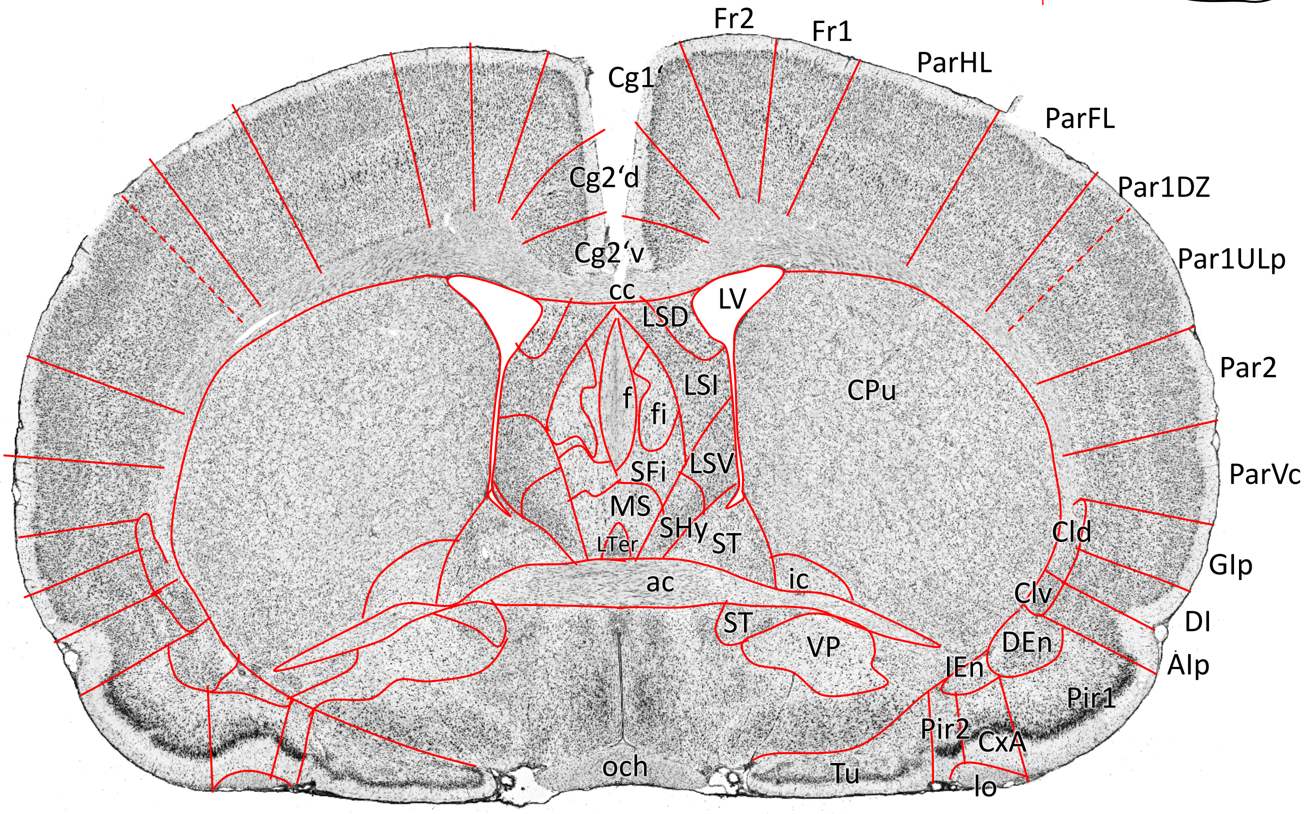

500µm

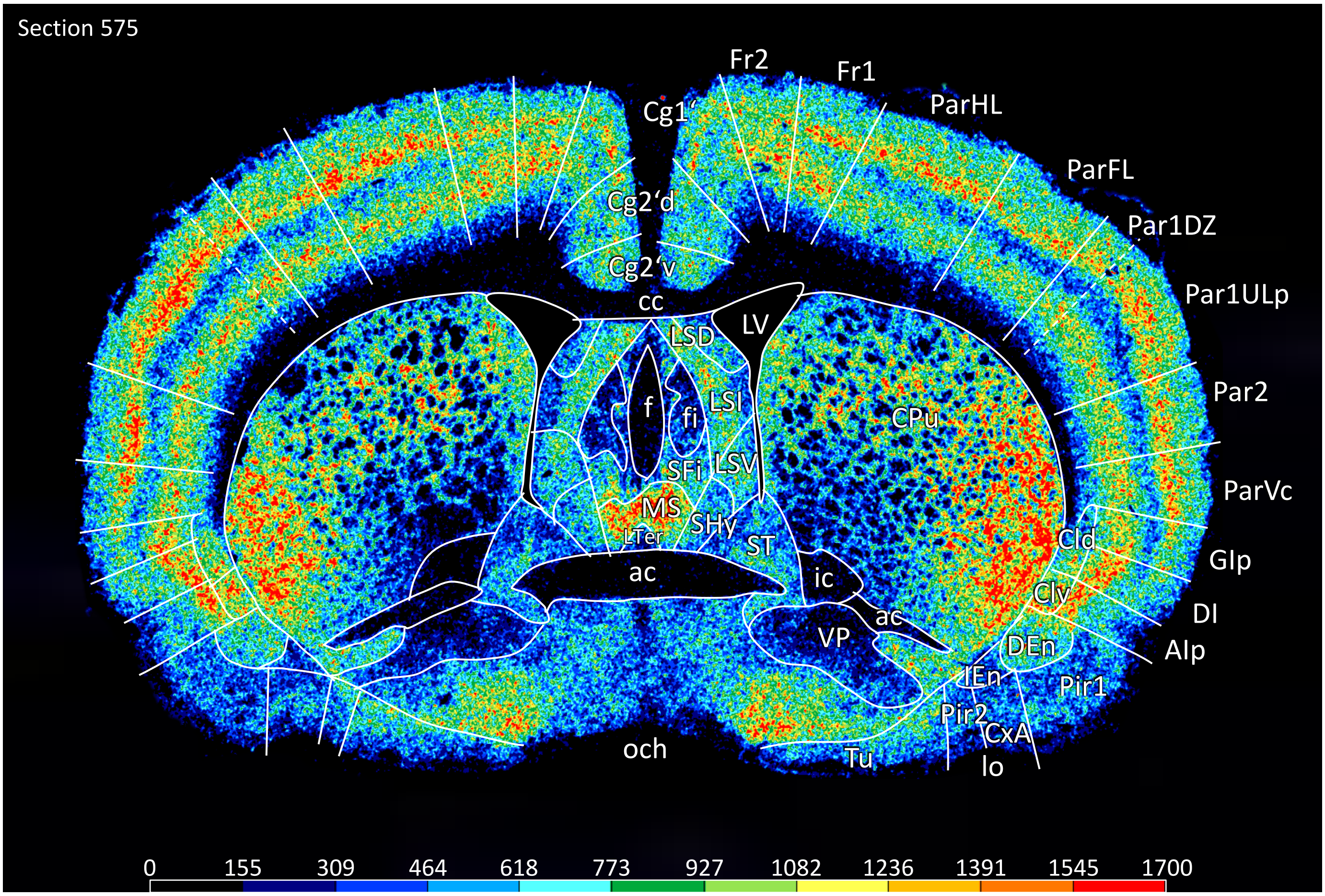

Level c16

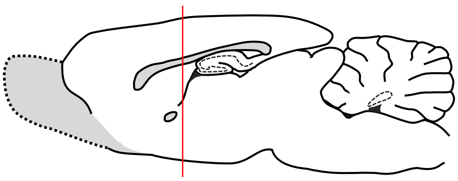

Section 598

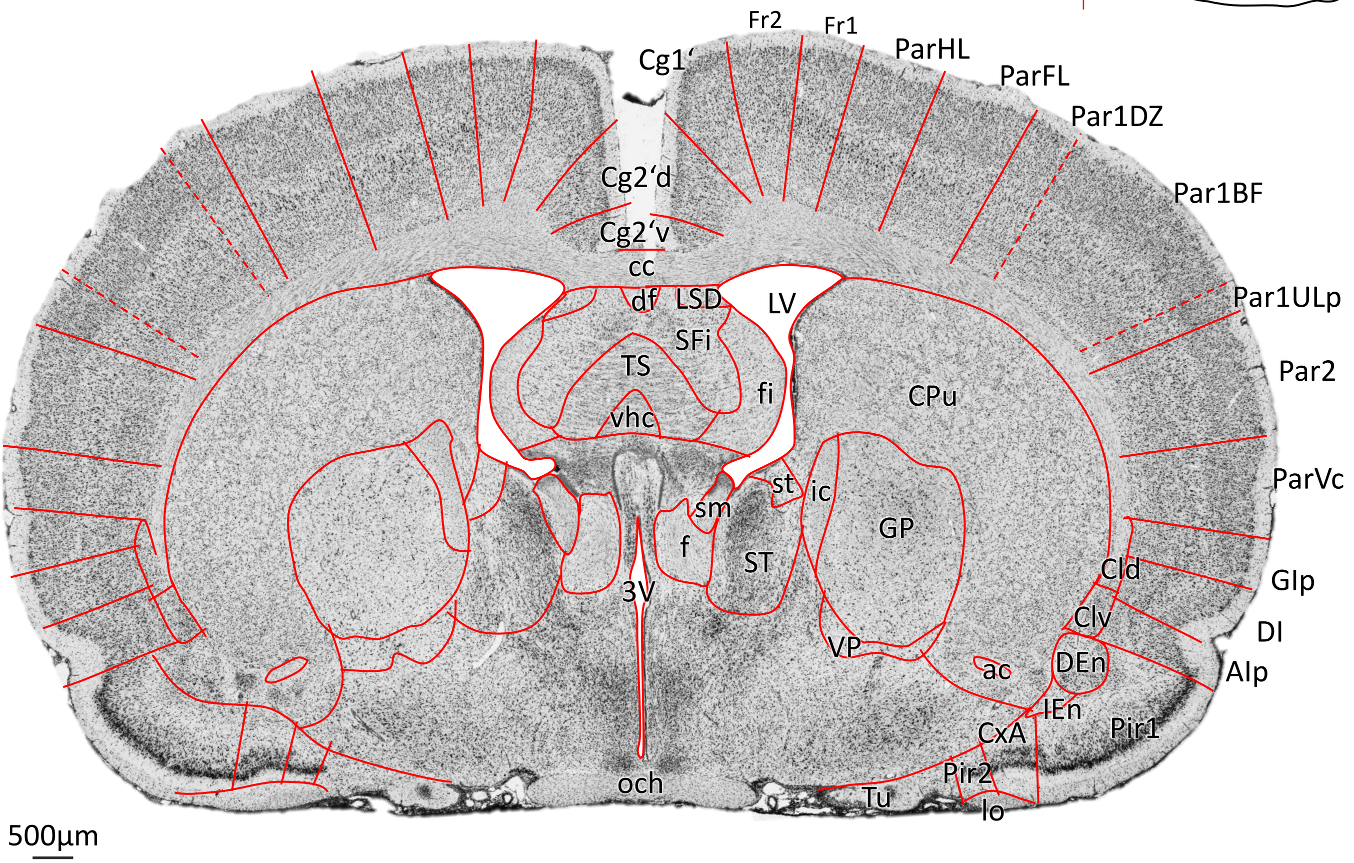

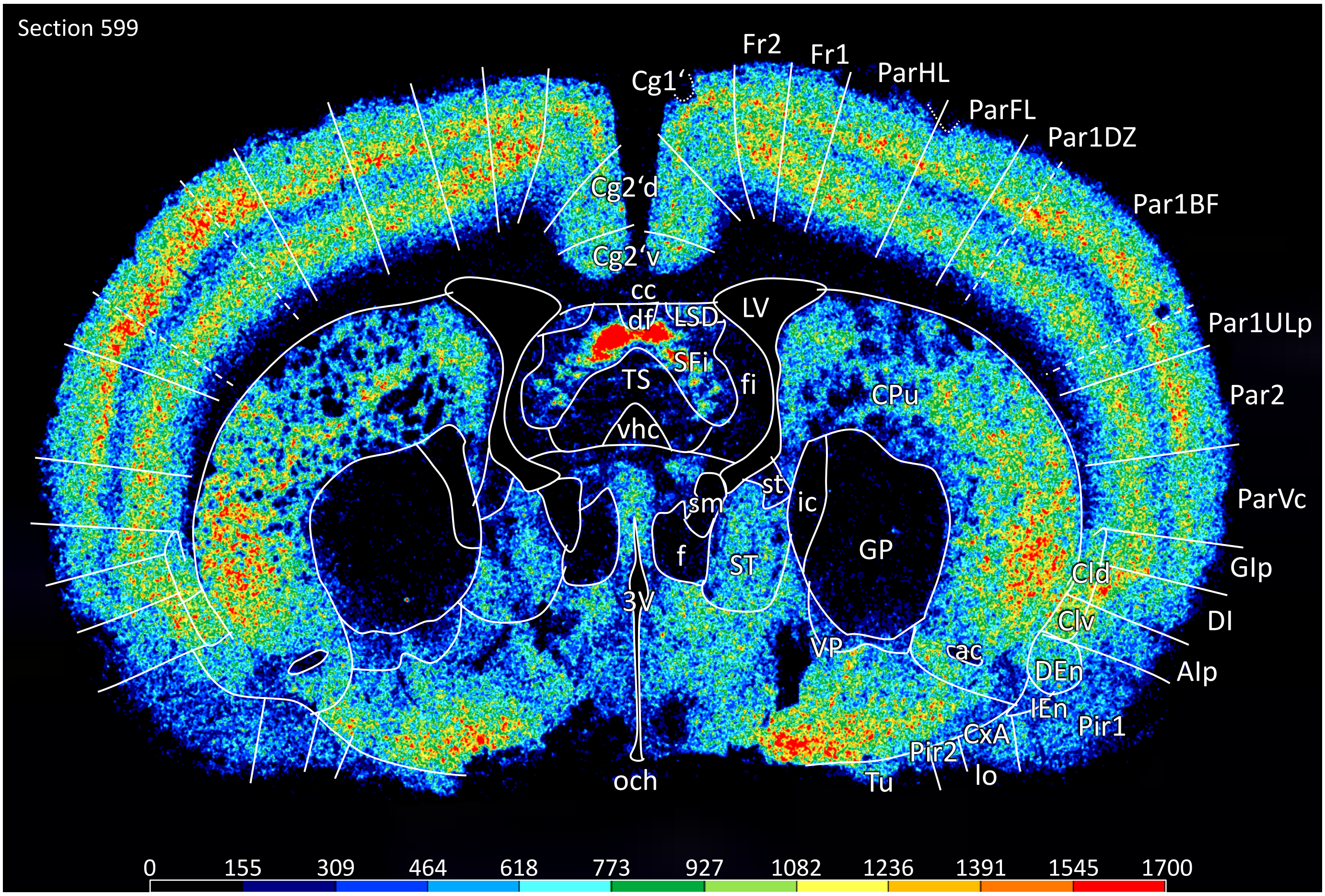

Level c17

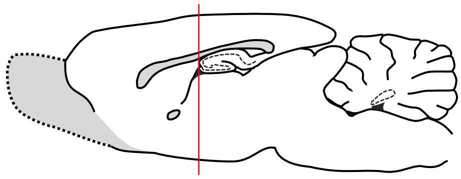

Section 634

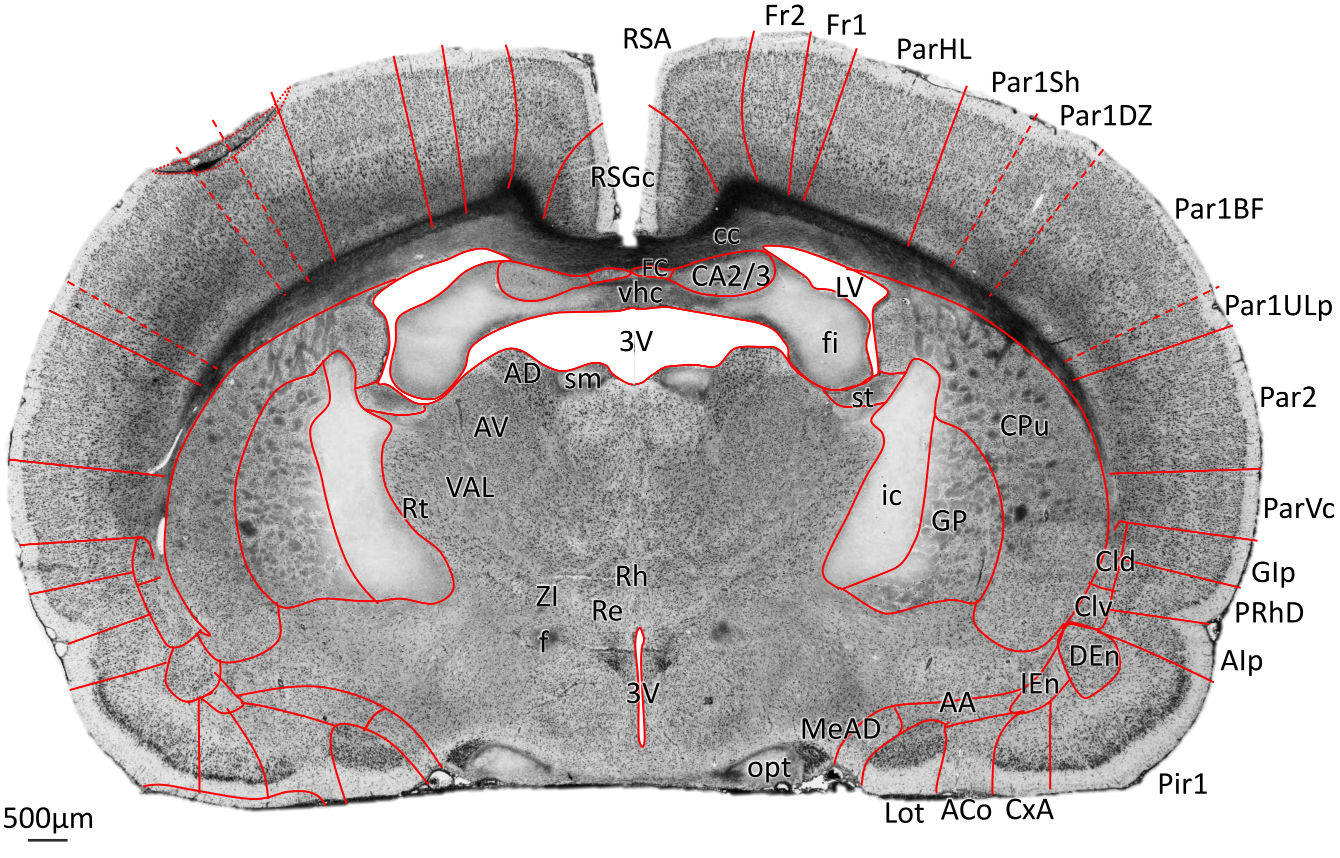

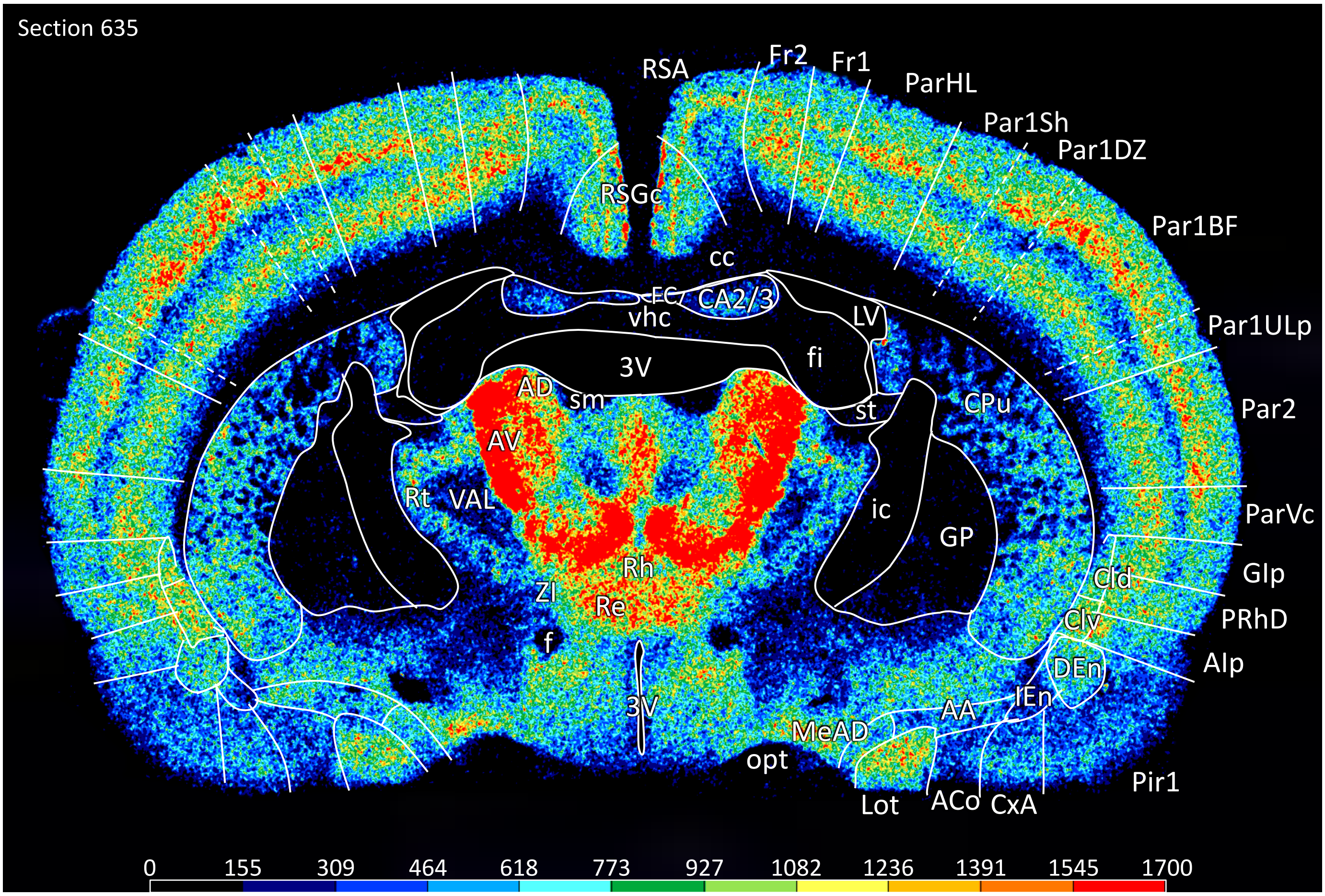

Level c18

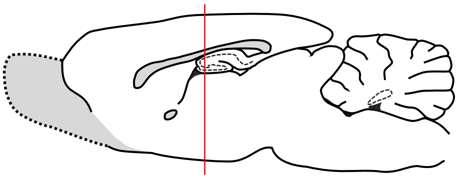

Section 658

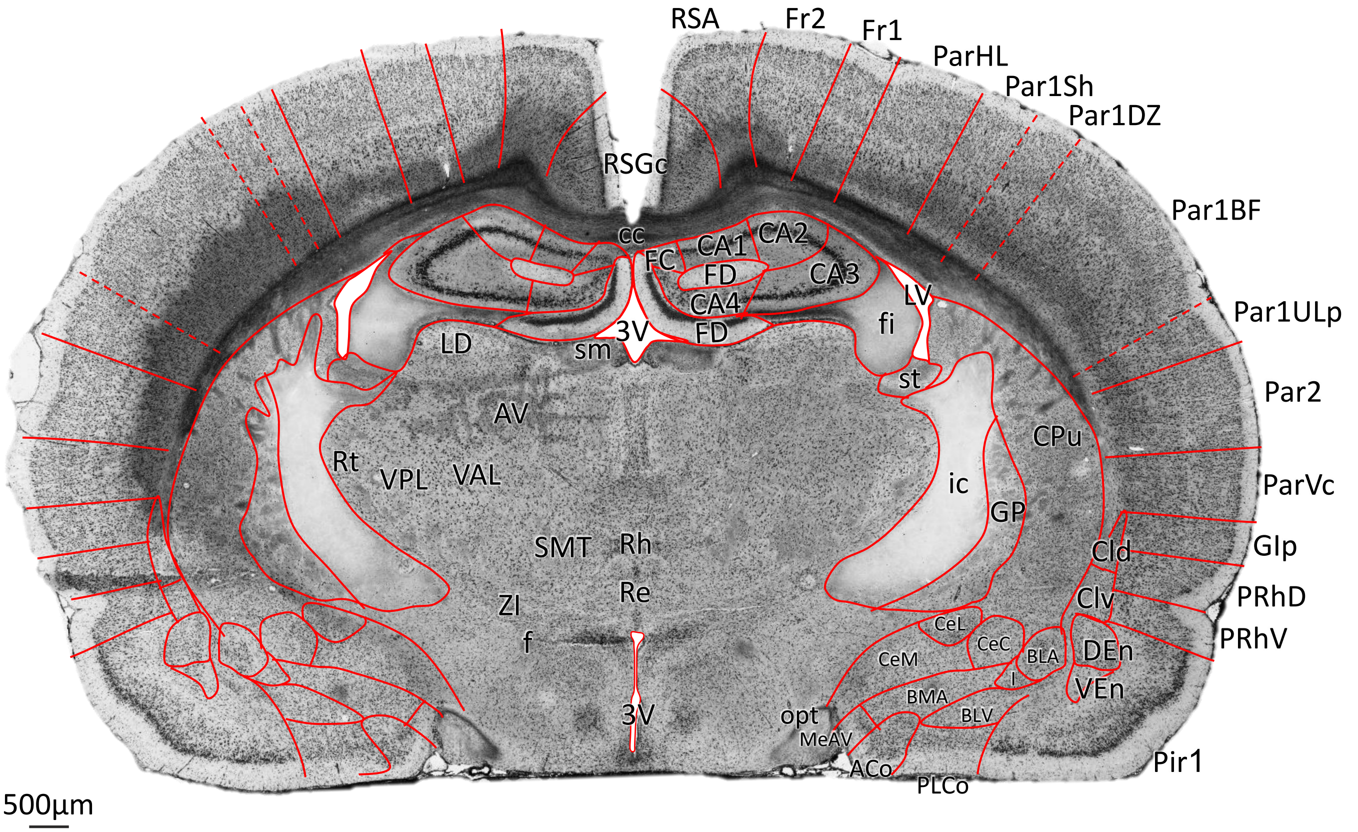

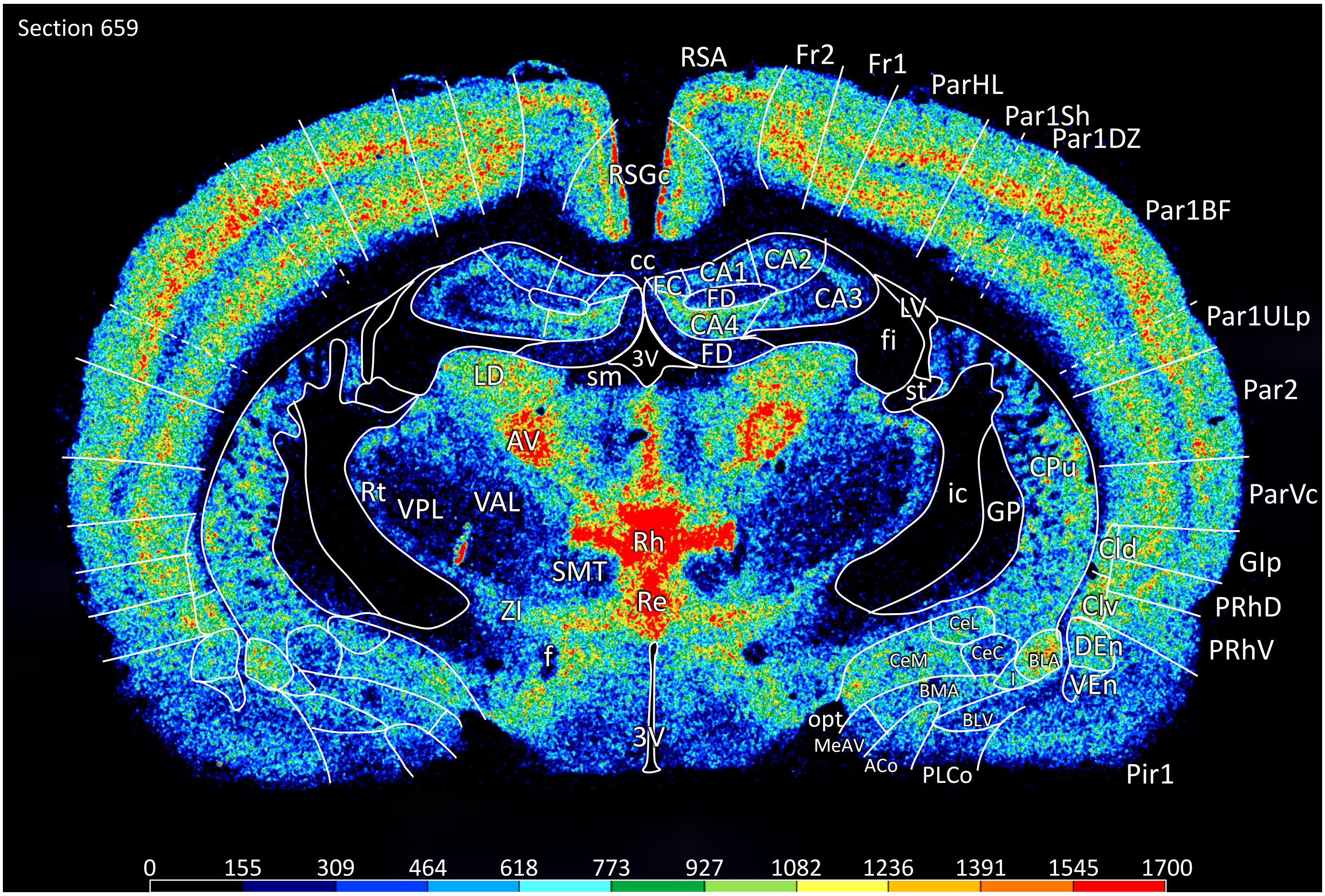

Level c19

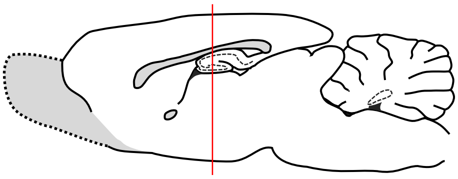

Section 682

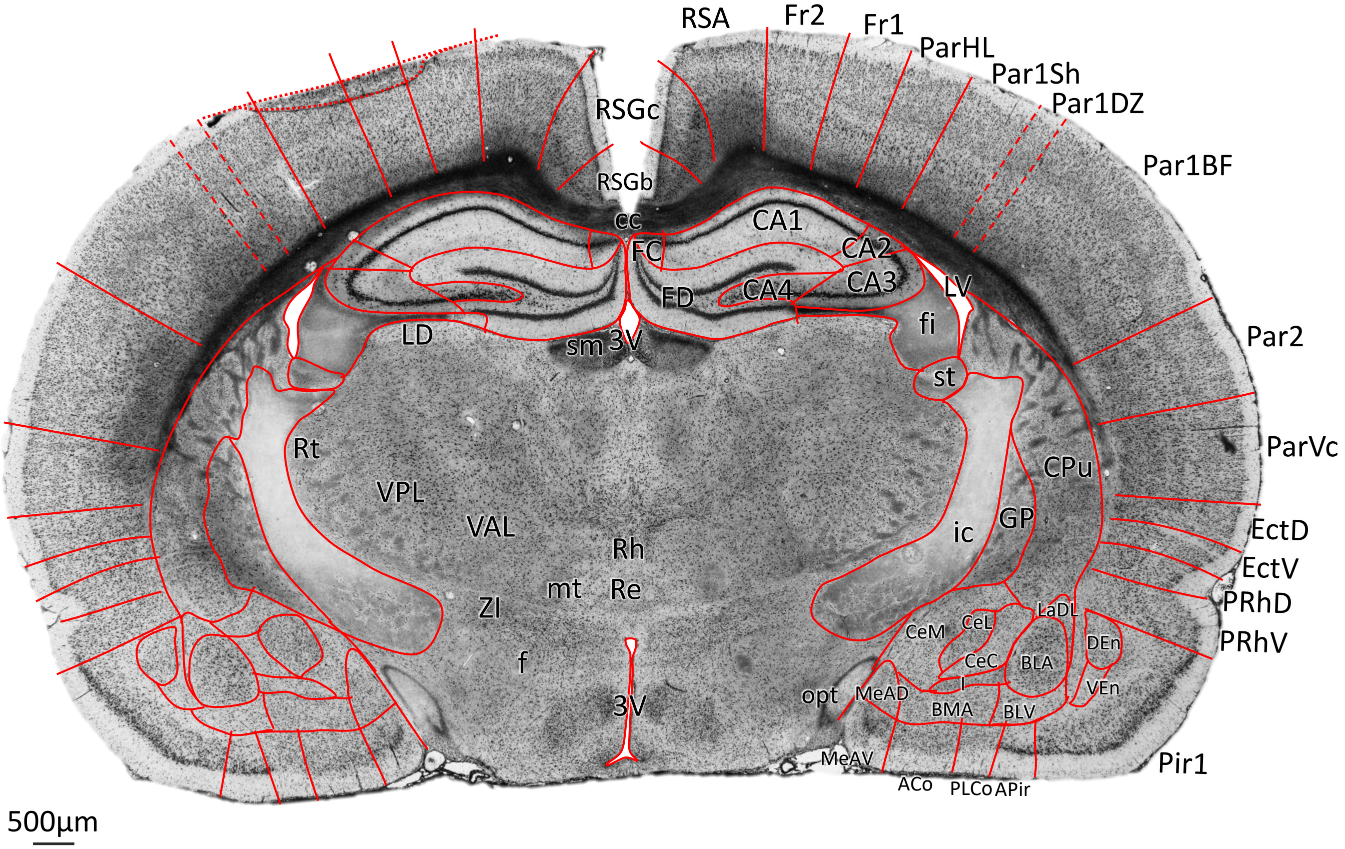

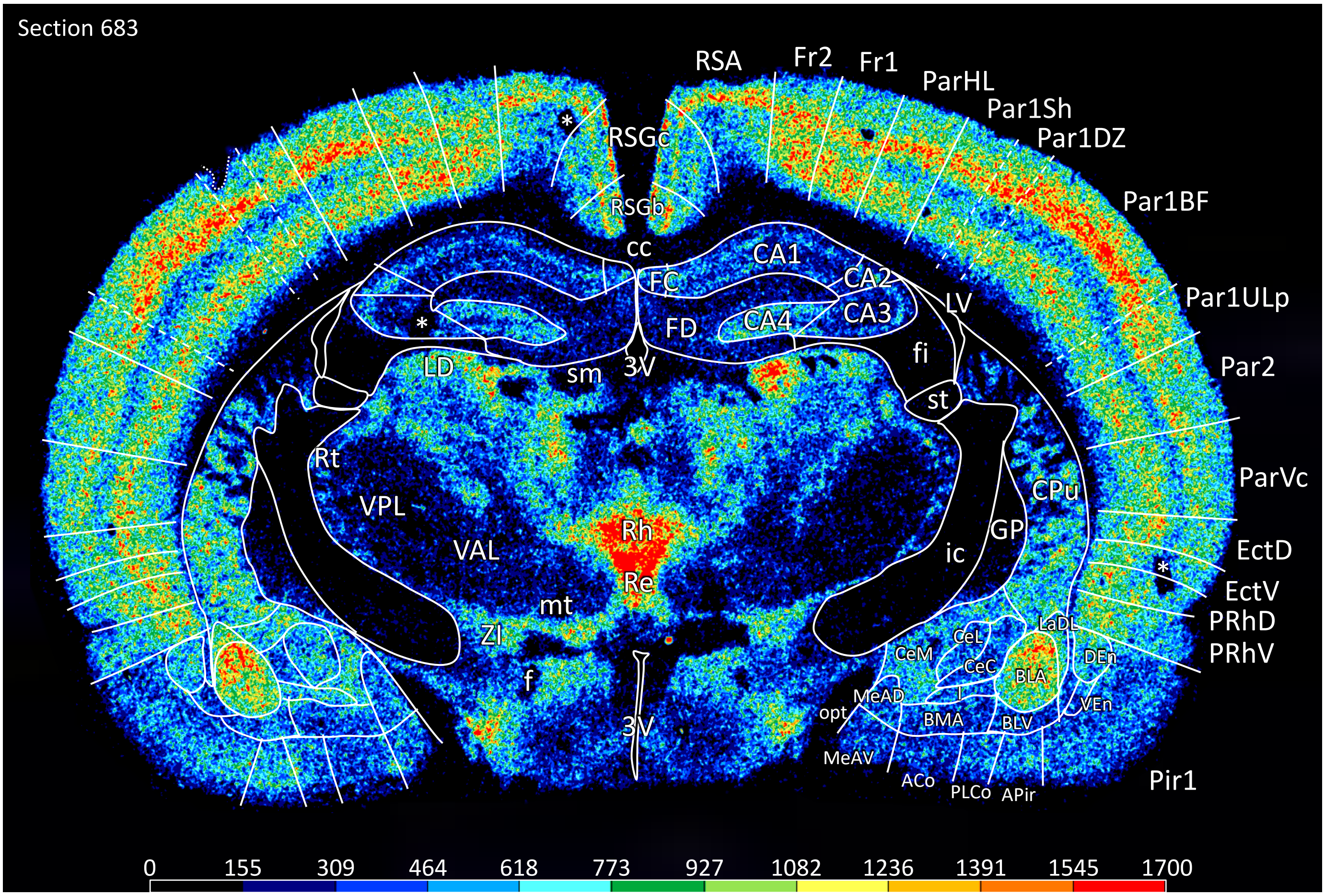

Level c20

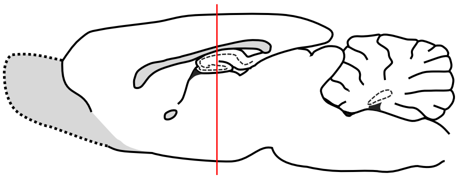

Section 694

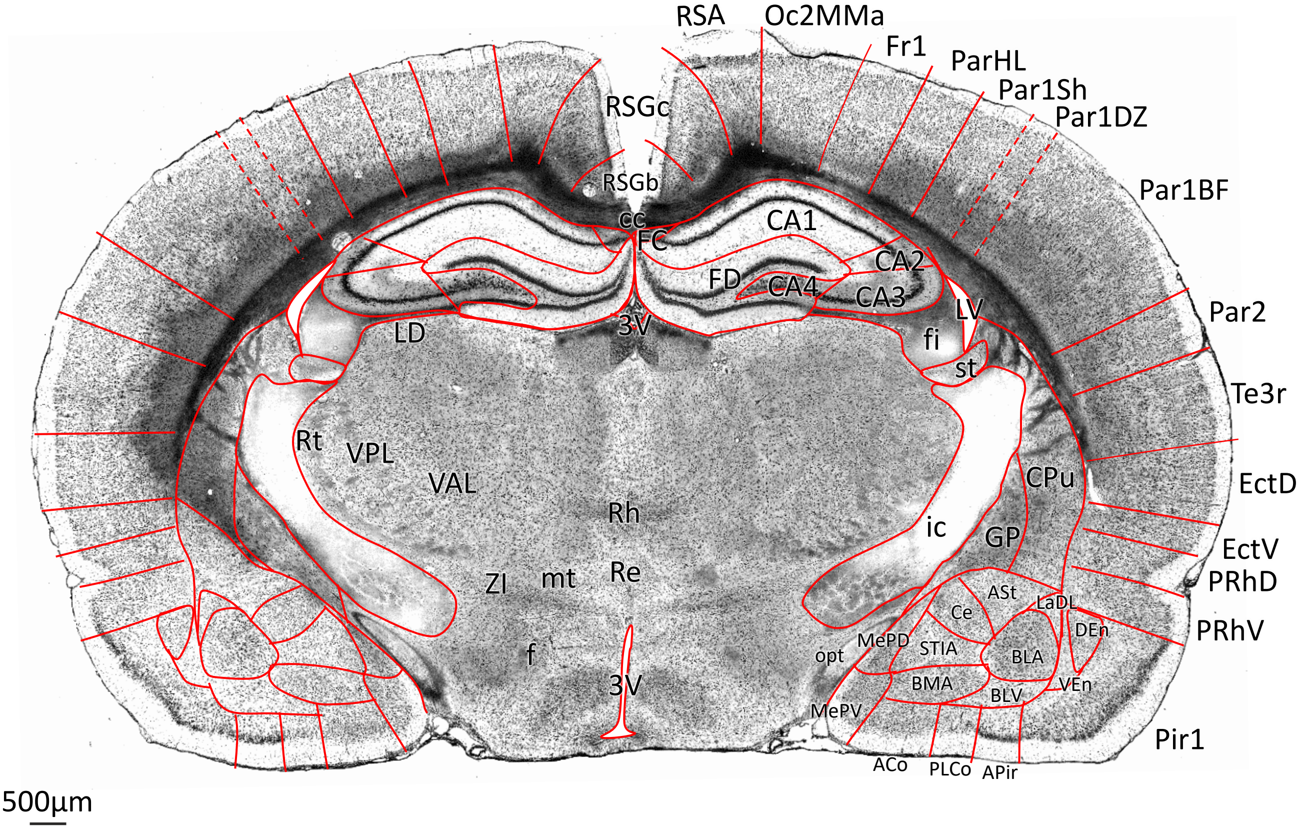

Section 695

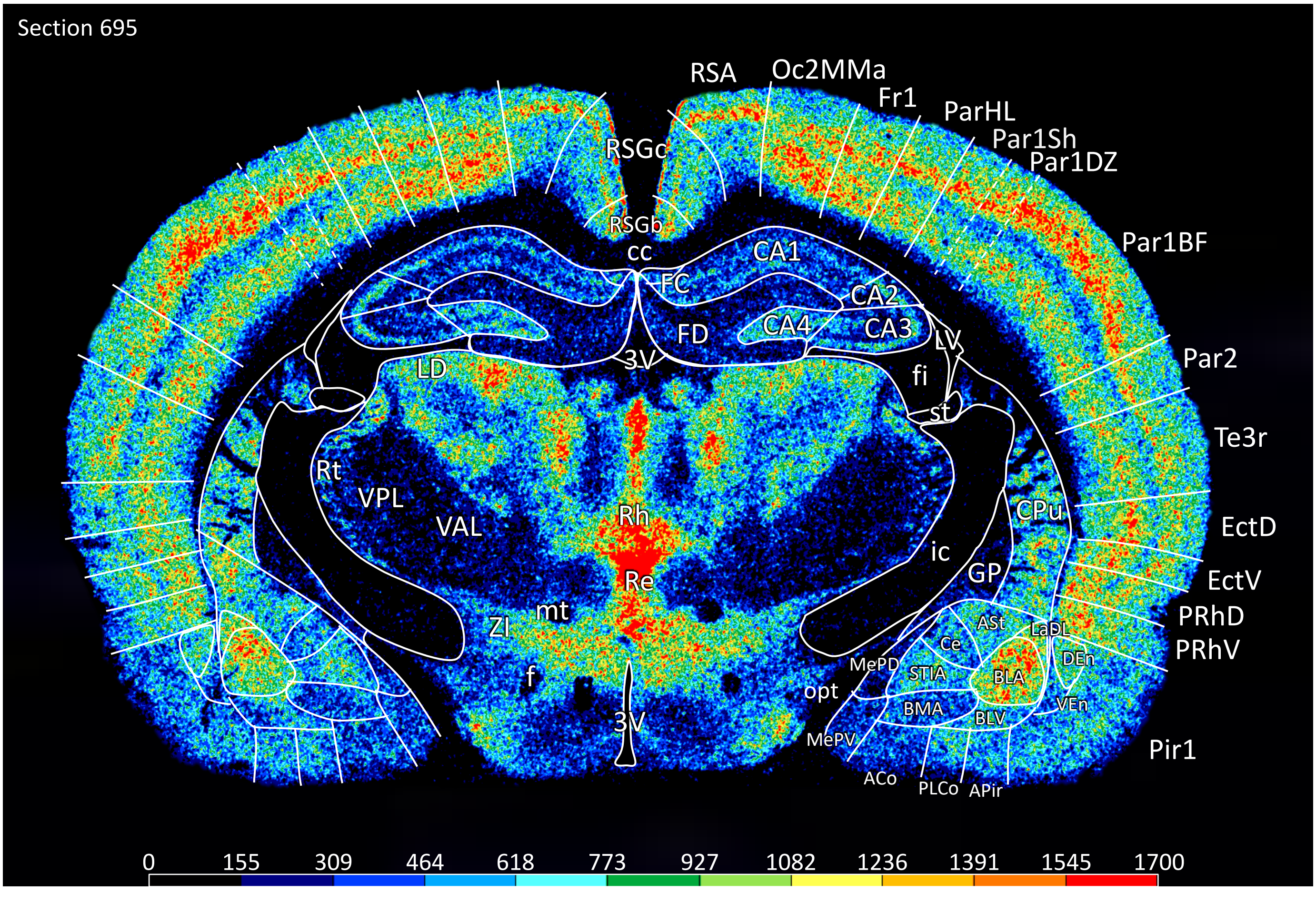

Level c21

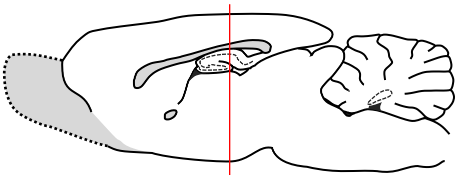

Section 727

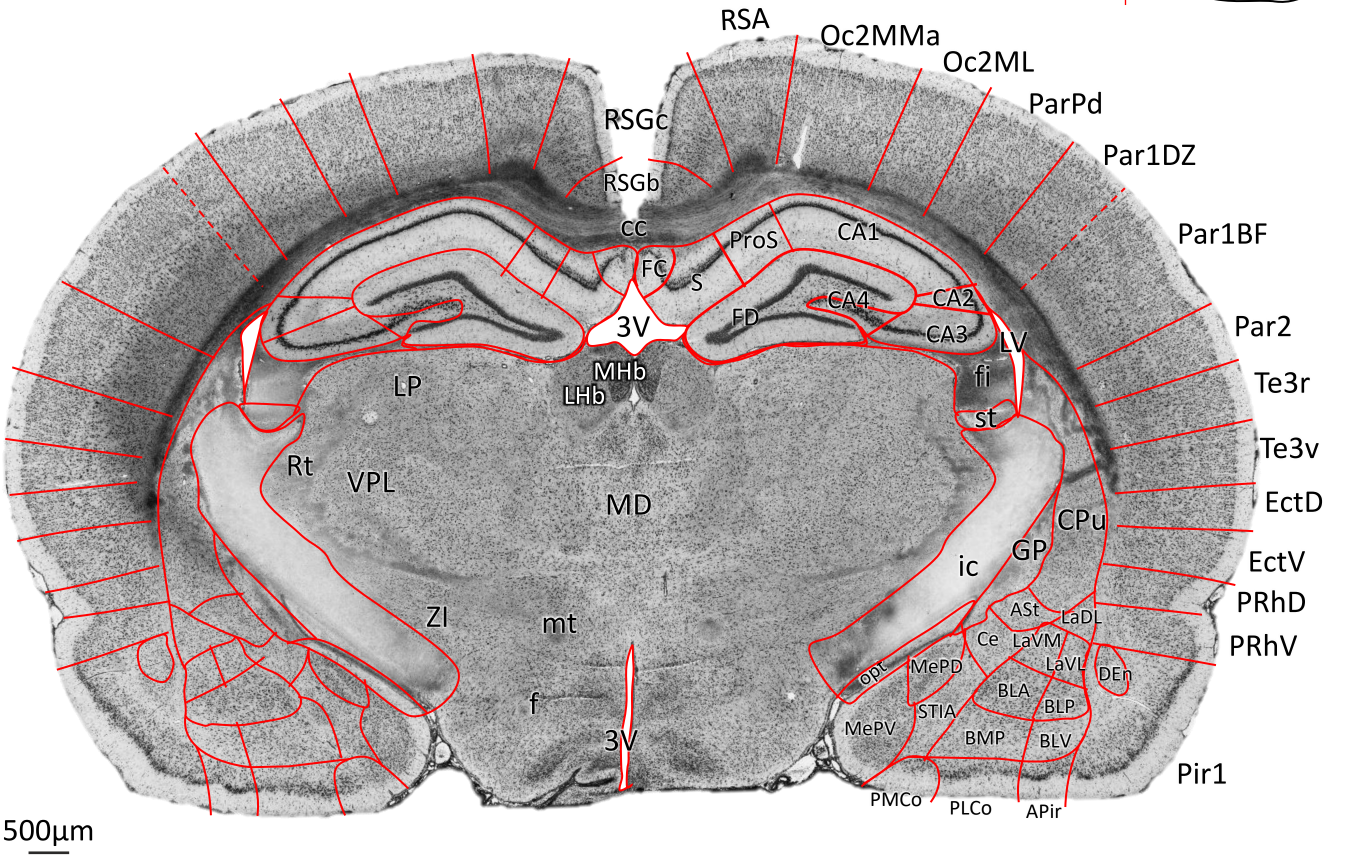

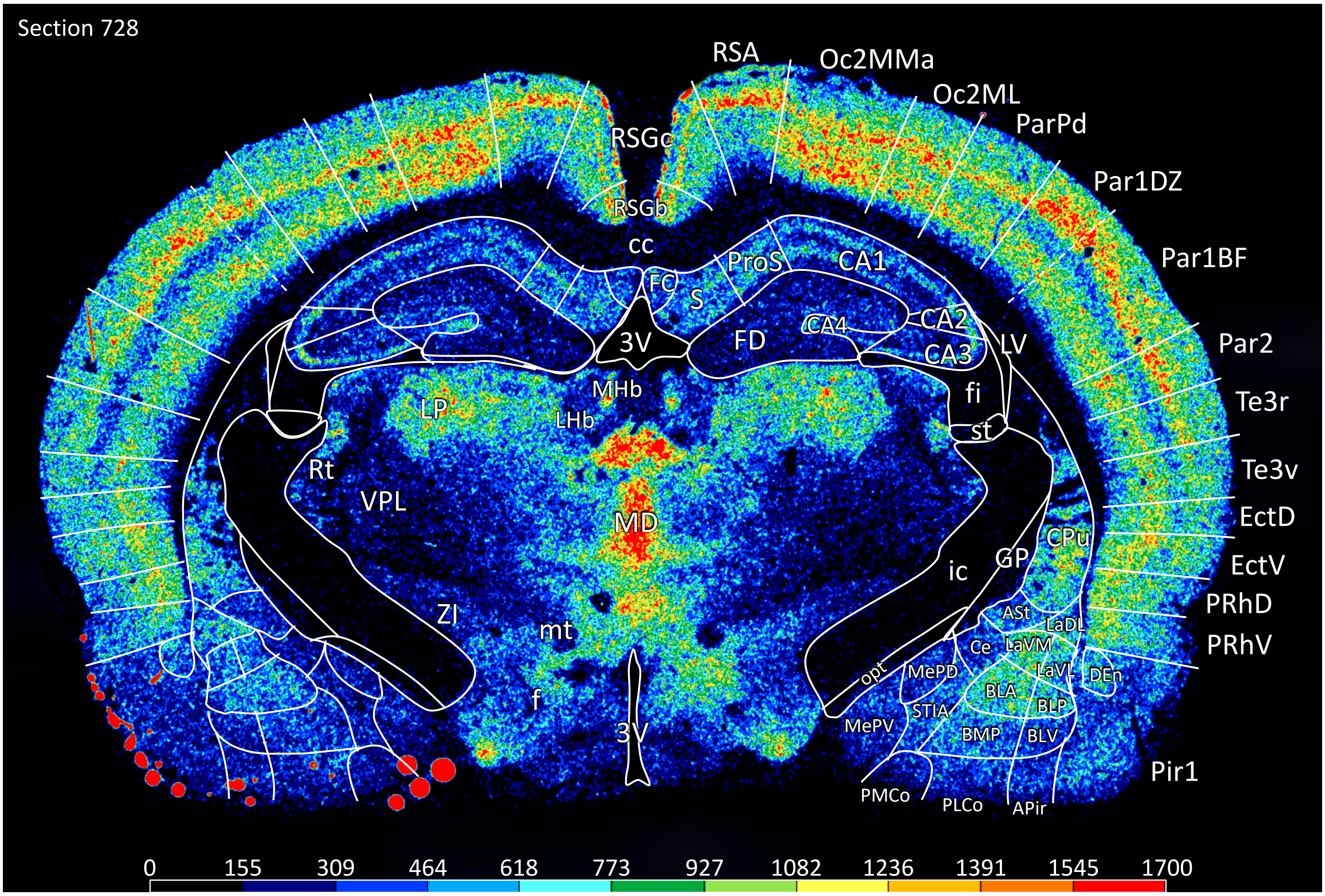

Level c22

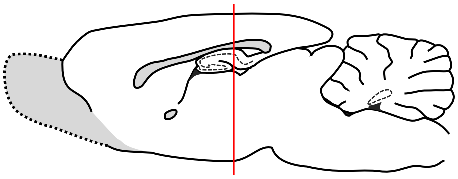

Section 739

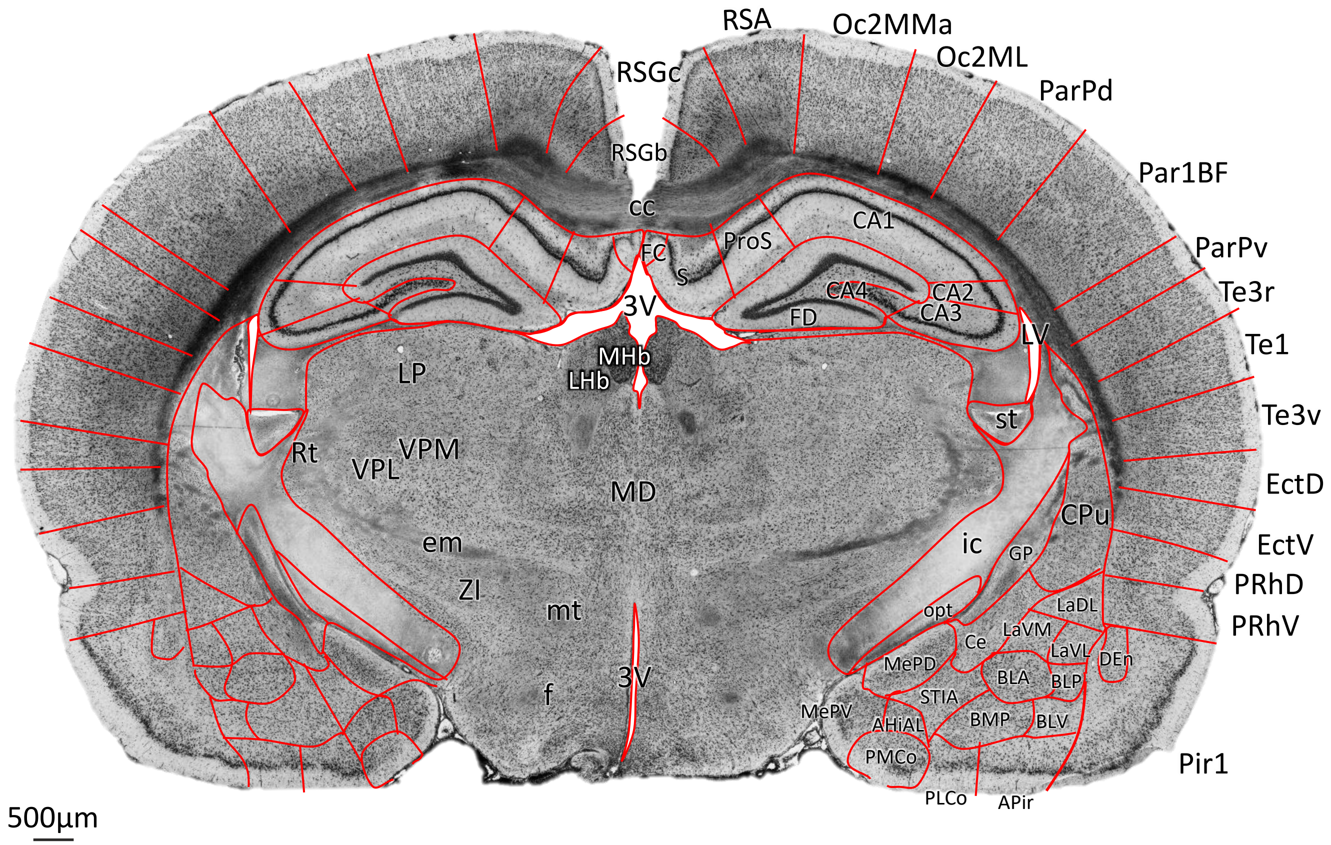

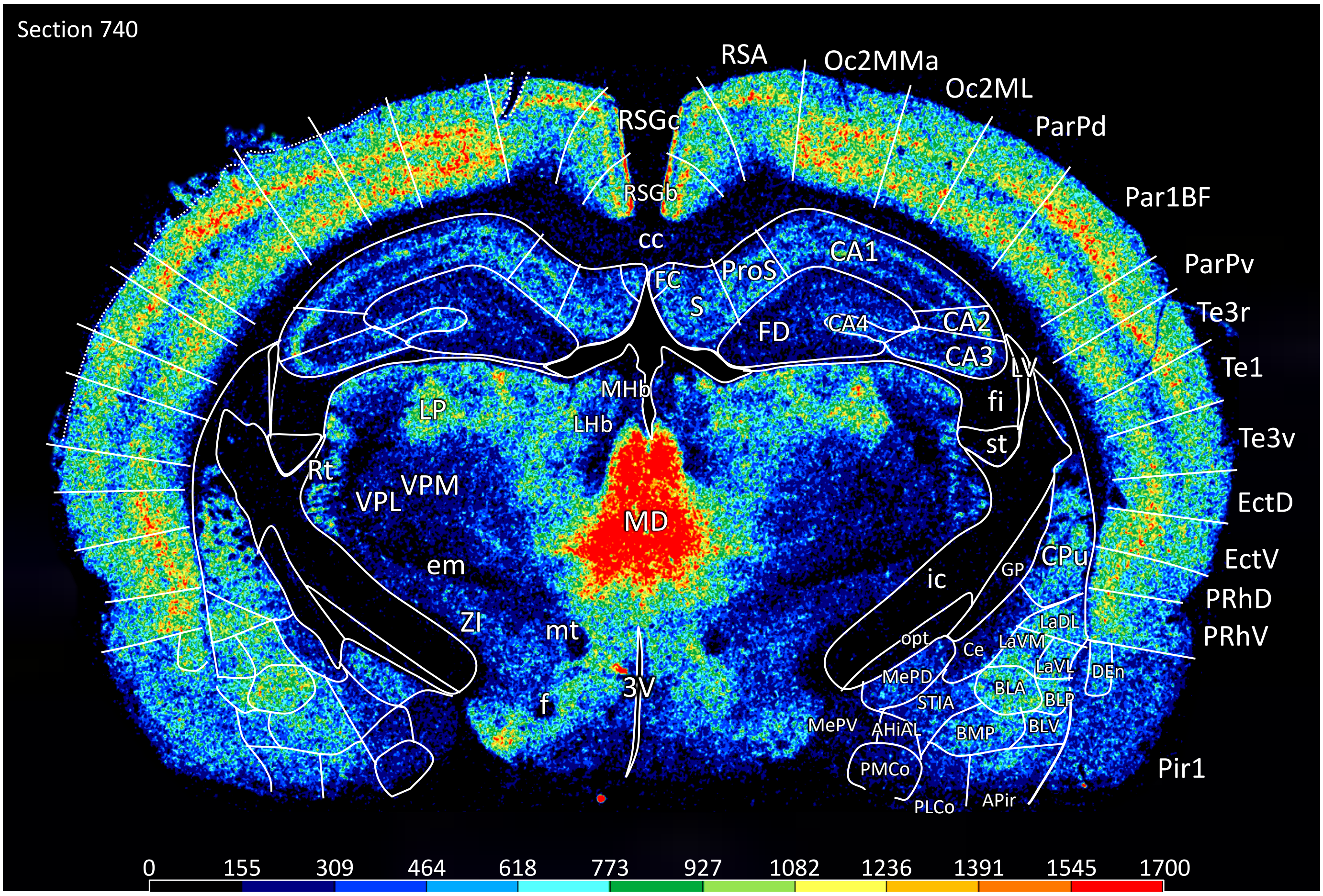

Level c23

Section 754

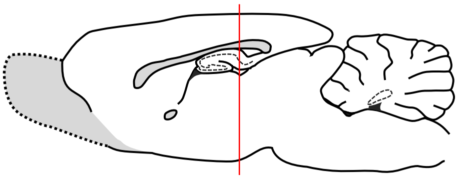

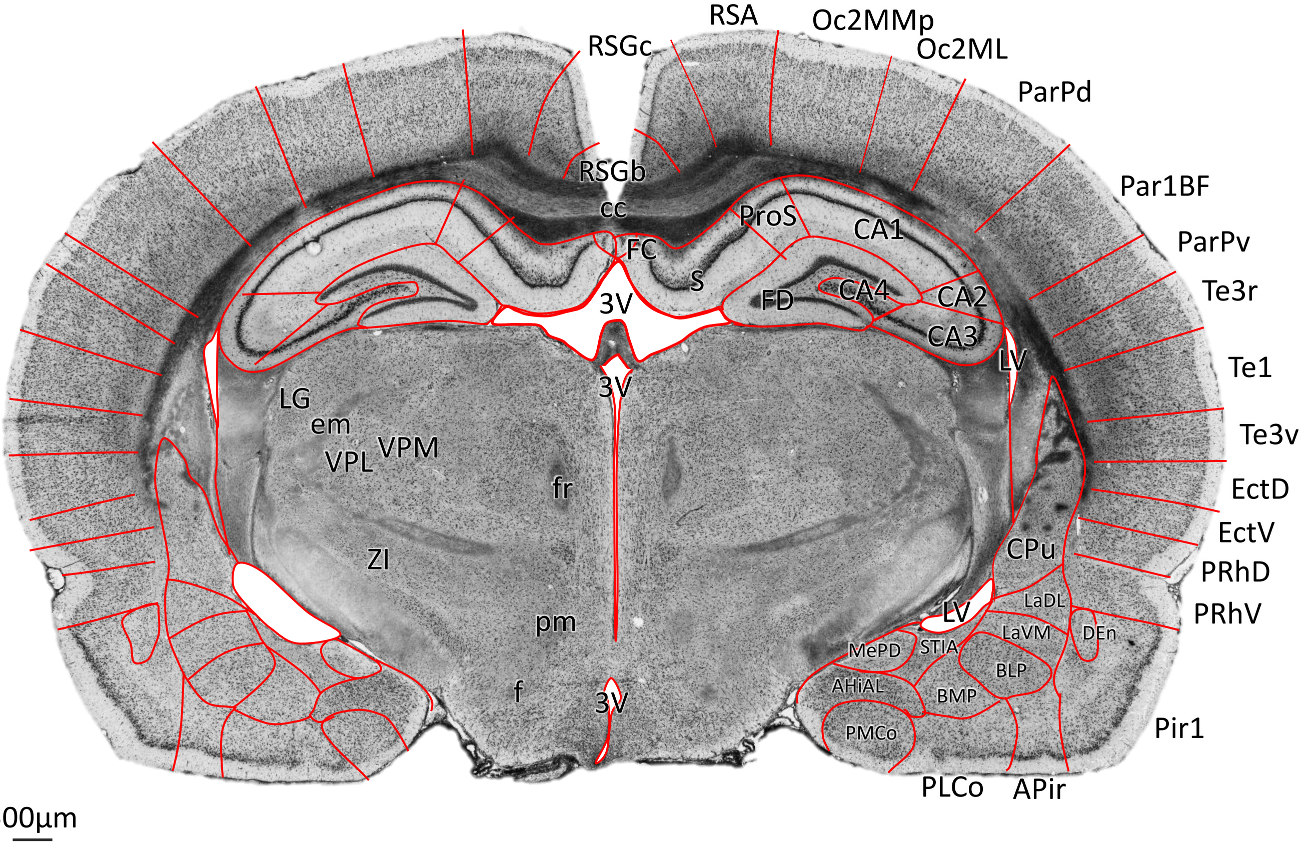

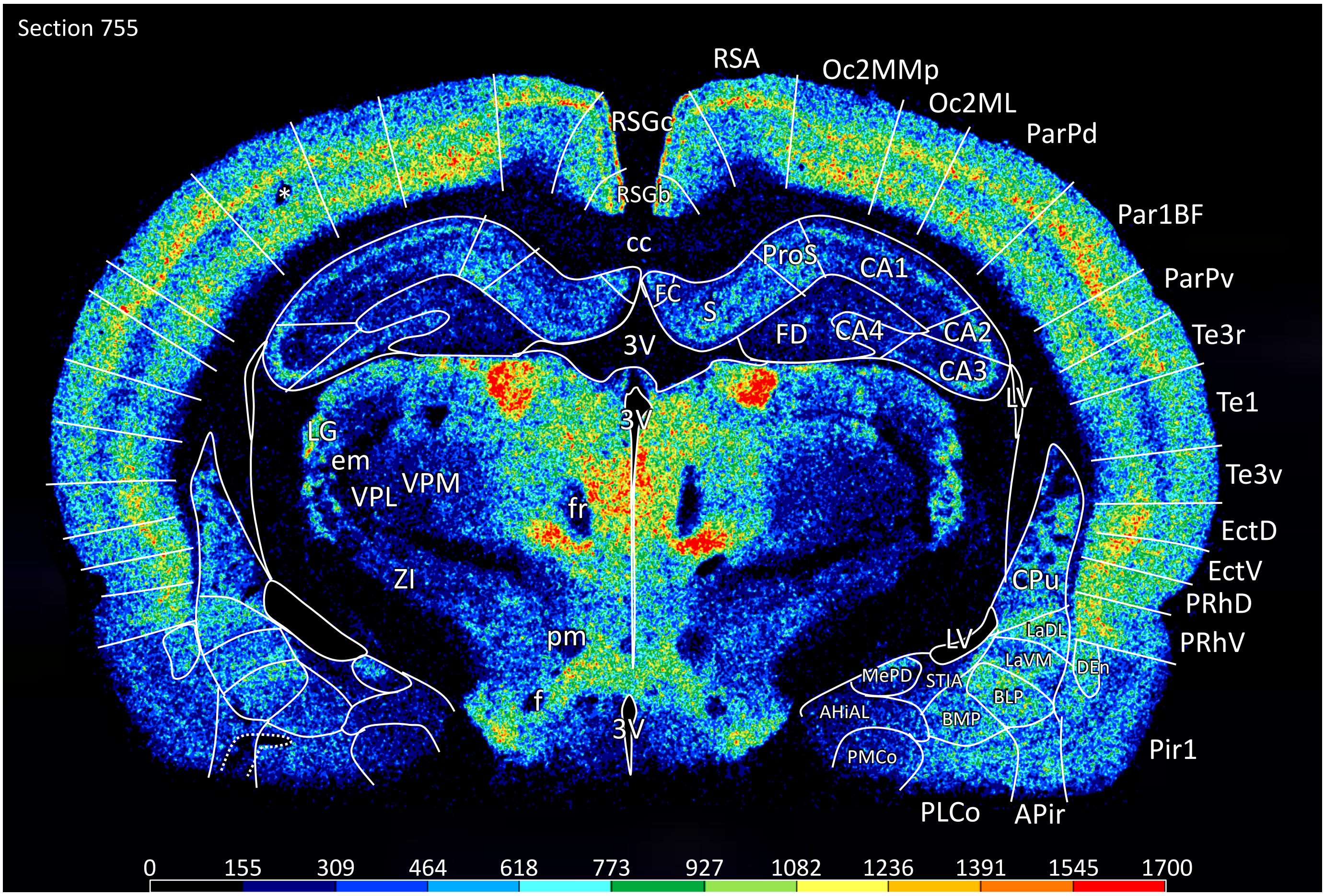

Level c24

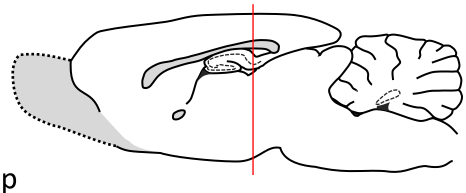

Section 769

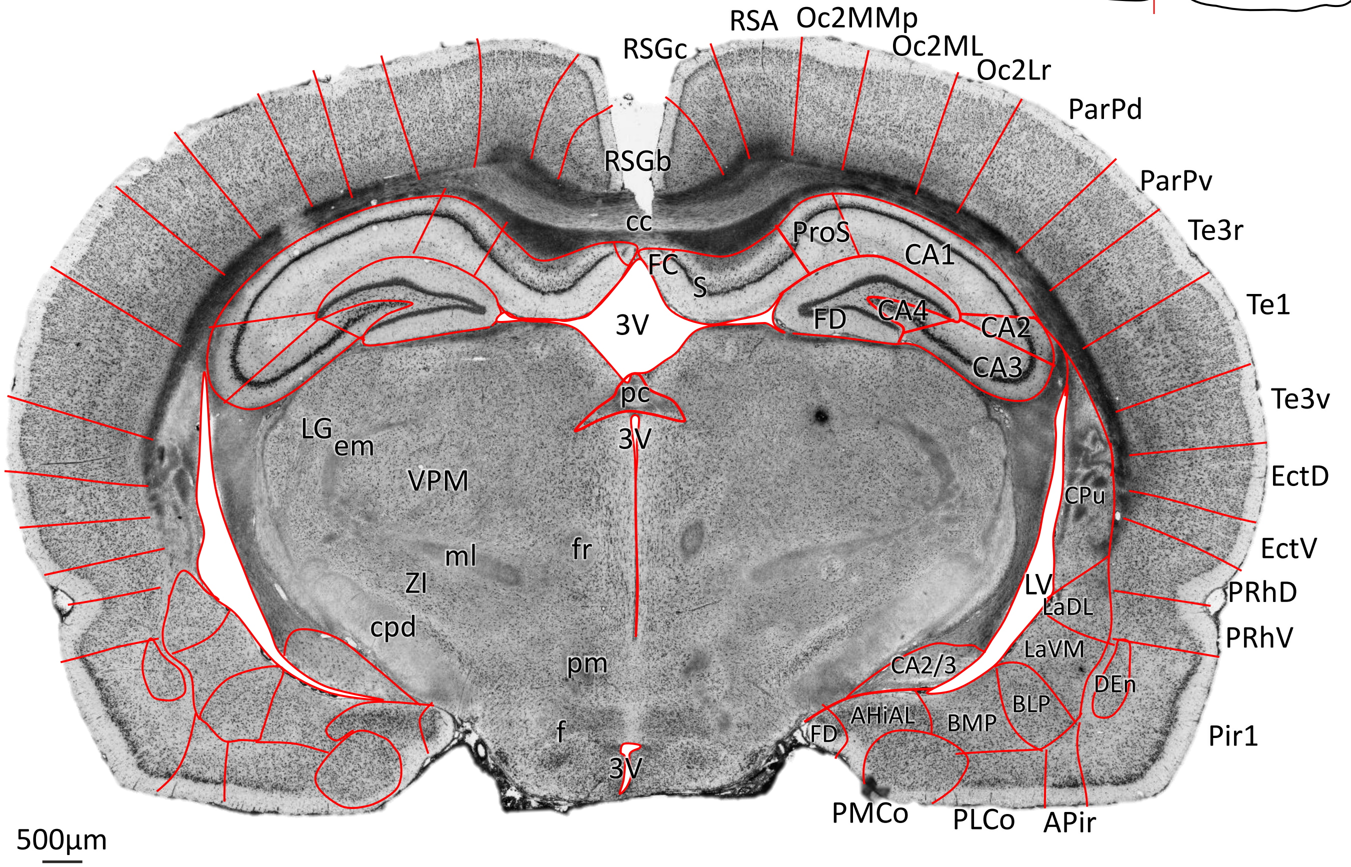

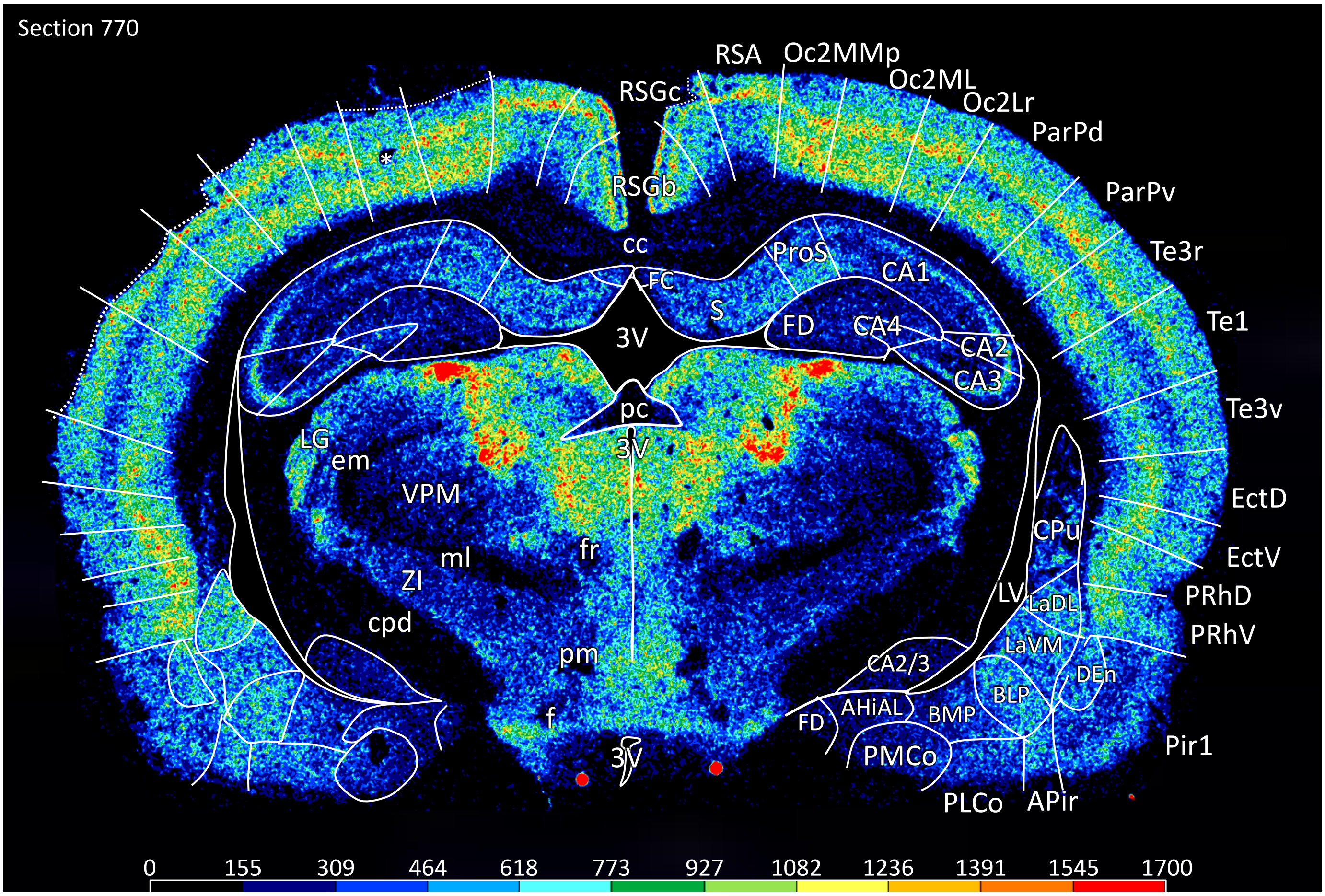

Level c25

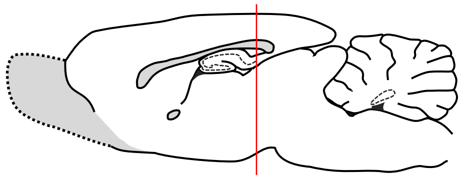

Section 793

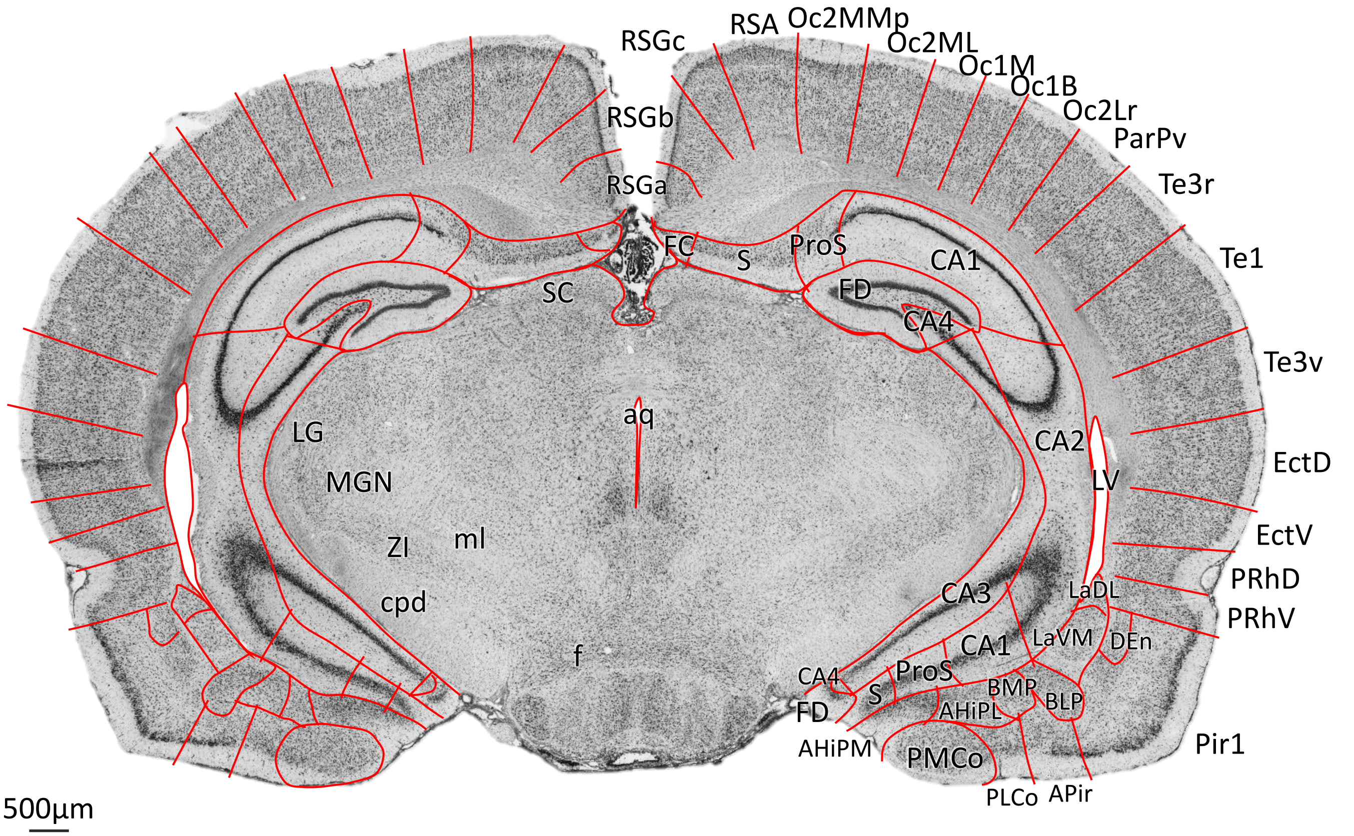

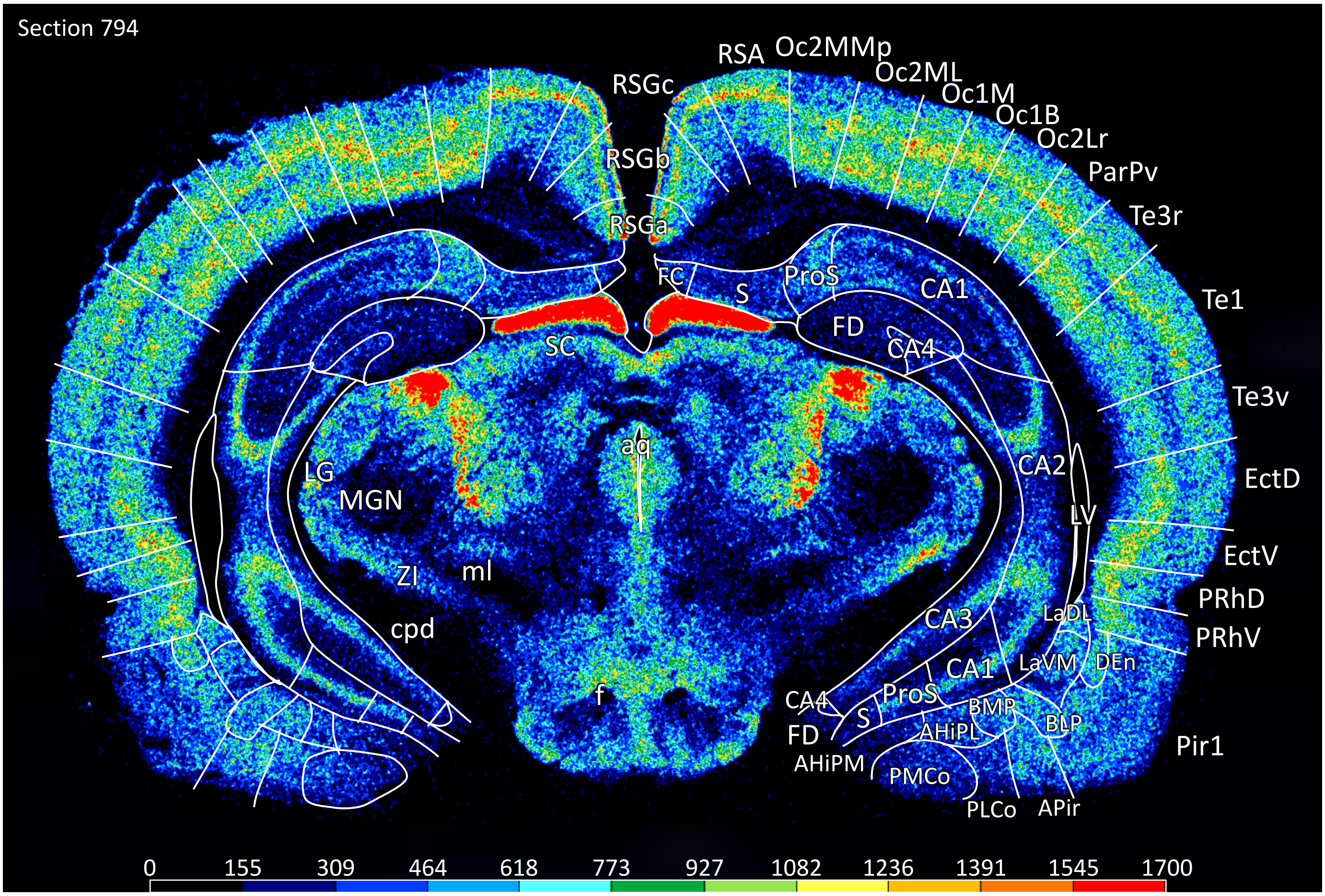

Level c26

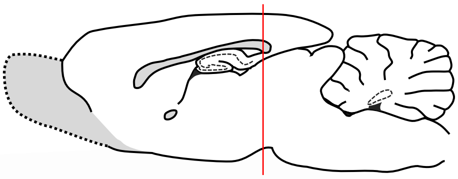

Section 817

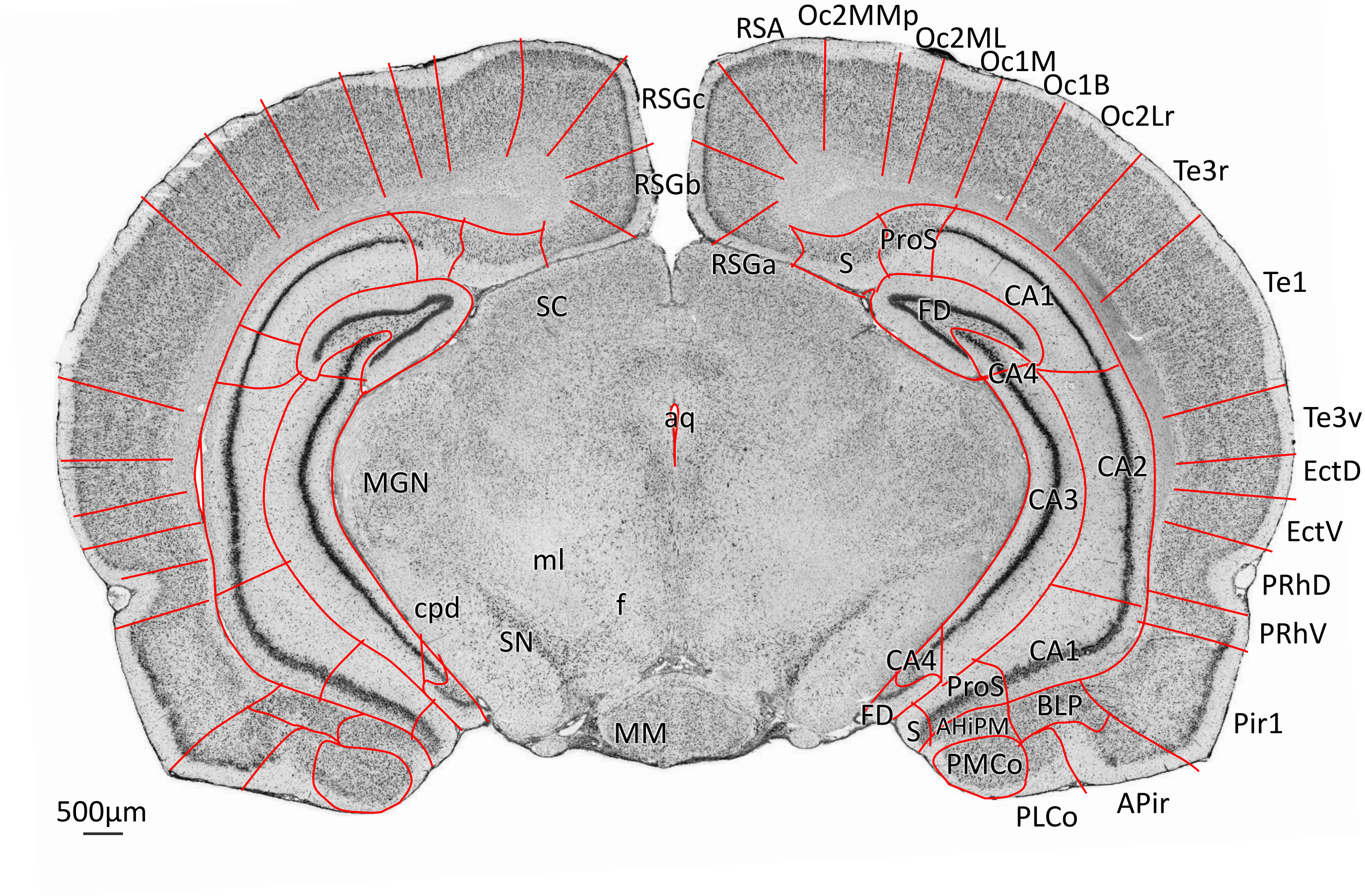

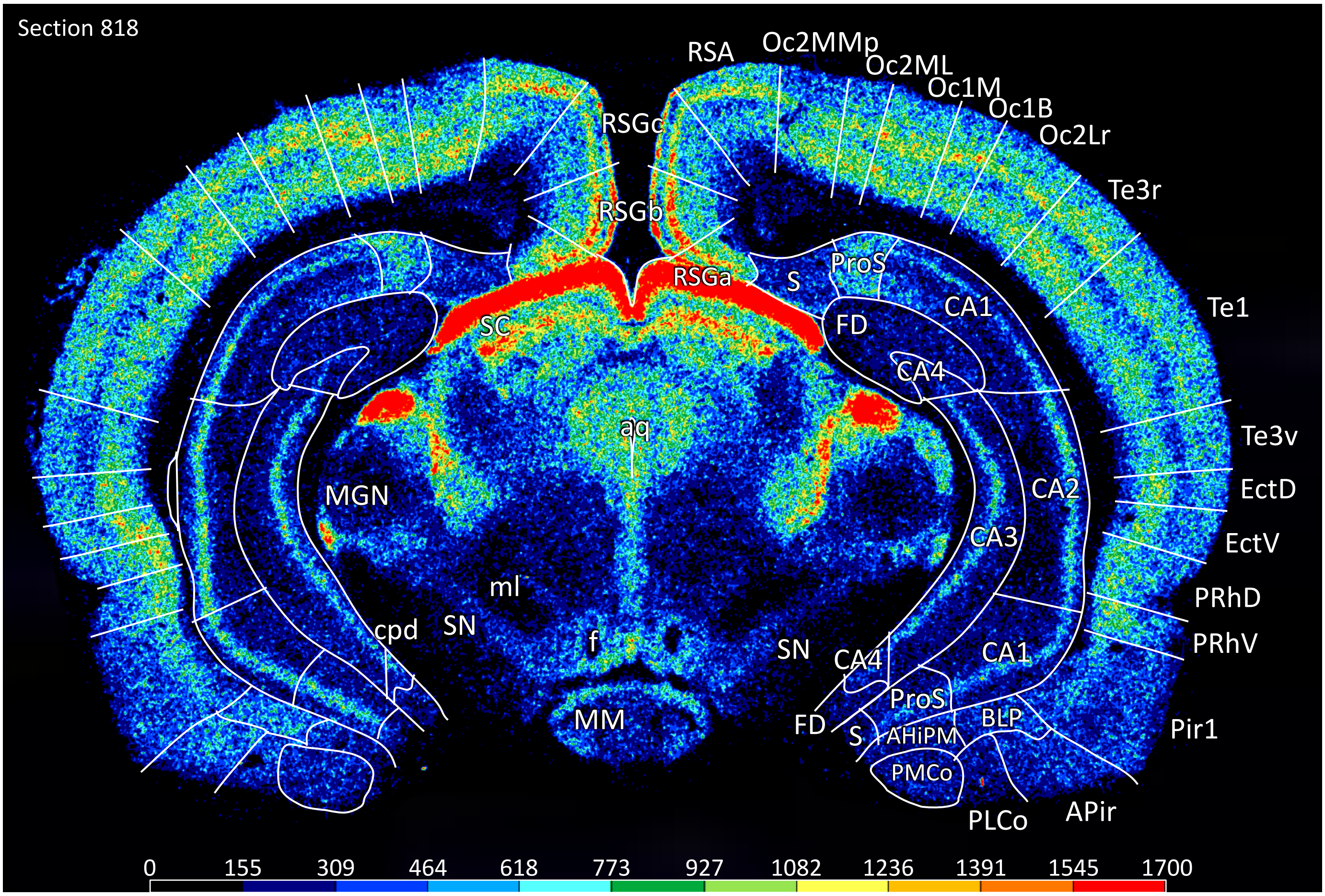

Level c27

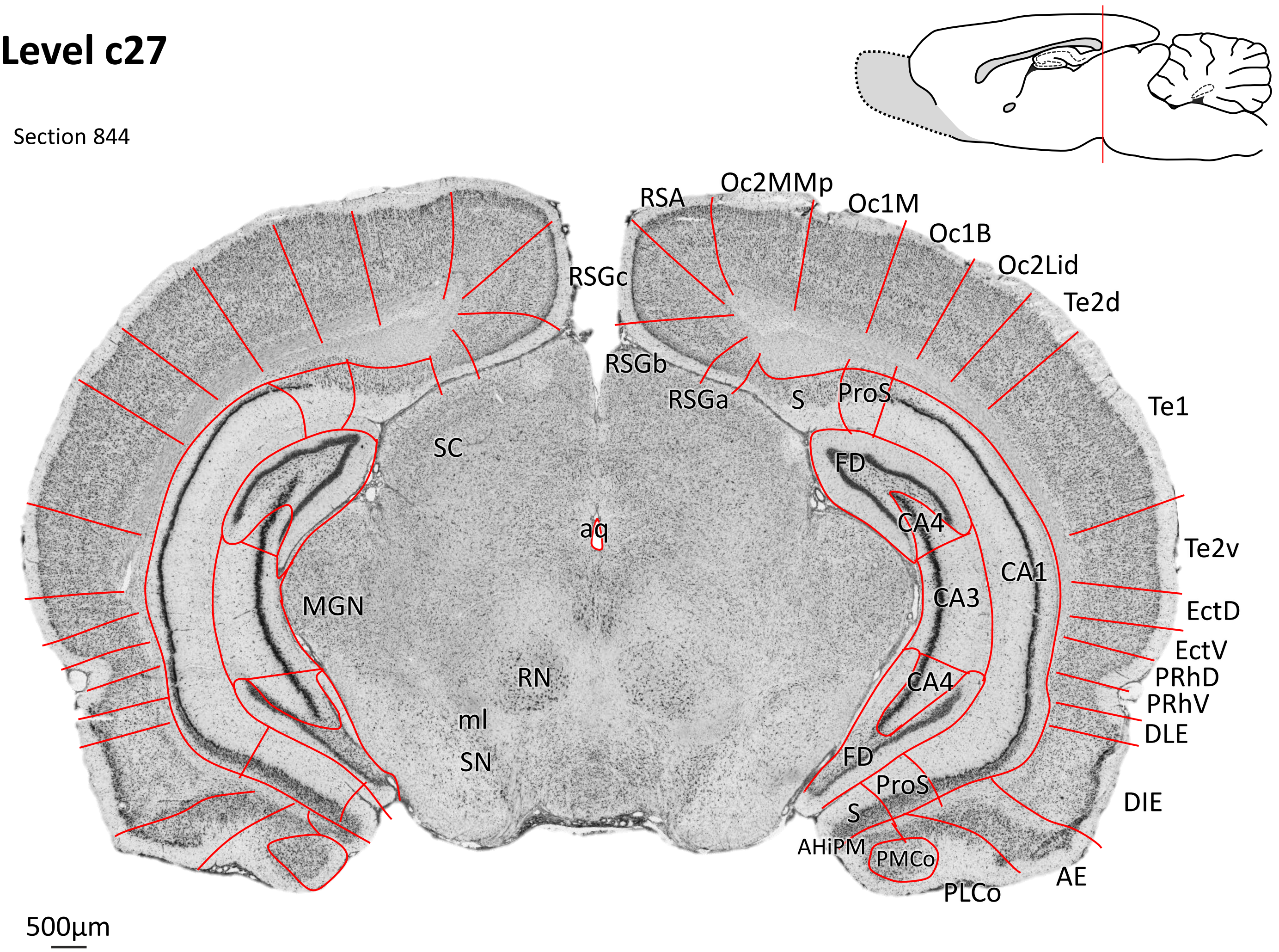
Section 844
RSA
Oc2MMp
Oc1M
Oc1B
Oc2Lid
Te2d
RSGc
RSGb
RSGa
S
ProS
Te1
SC
FD
aq
CA4
Te2v
CA1
MGN
CA3
EctD
EctV
RN
CA4
PRhD
PRhV
ml
DLE
SN
FD
ProS
DIE
S
AHiPM
PMCo
AE
PLCo
500µm

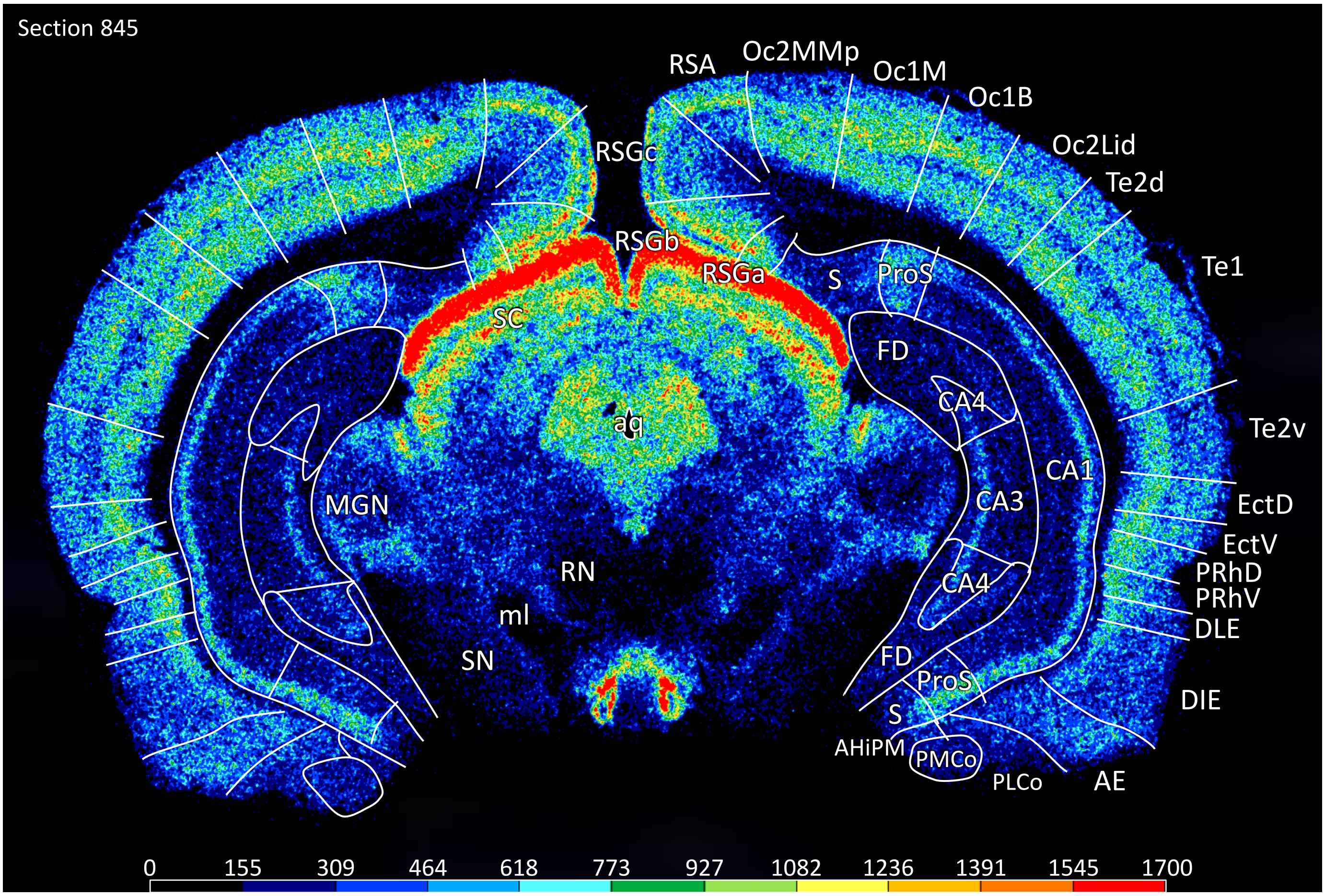
Section 845
RSA
Oc2MMp
Oc1M
Oc1B
RSGc
Oc2Lid
Te2d
RSGb
RSGa
S
ProS
Te1
SC
FD
aq
CA4
Te2v
CA1
MGN
CA3
EctD
EctV
RN
PRhD
CA4
PRhV
ml
DLE
SN
FD
ProS
DIE
S
AHiPM
PMCo
PLCo
AE
0
155
309
464
618
773
927
1082
1236
1391
1545
1700

Level c28

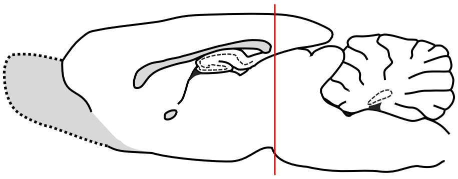

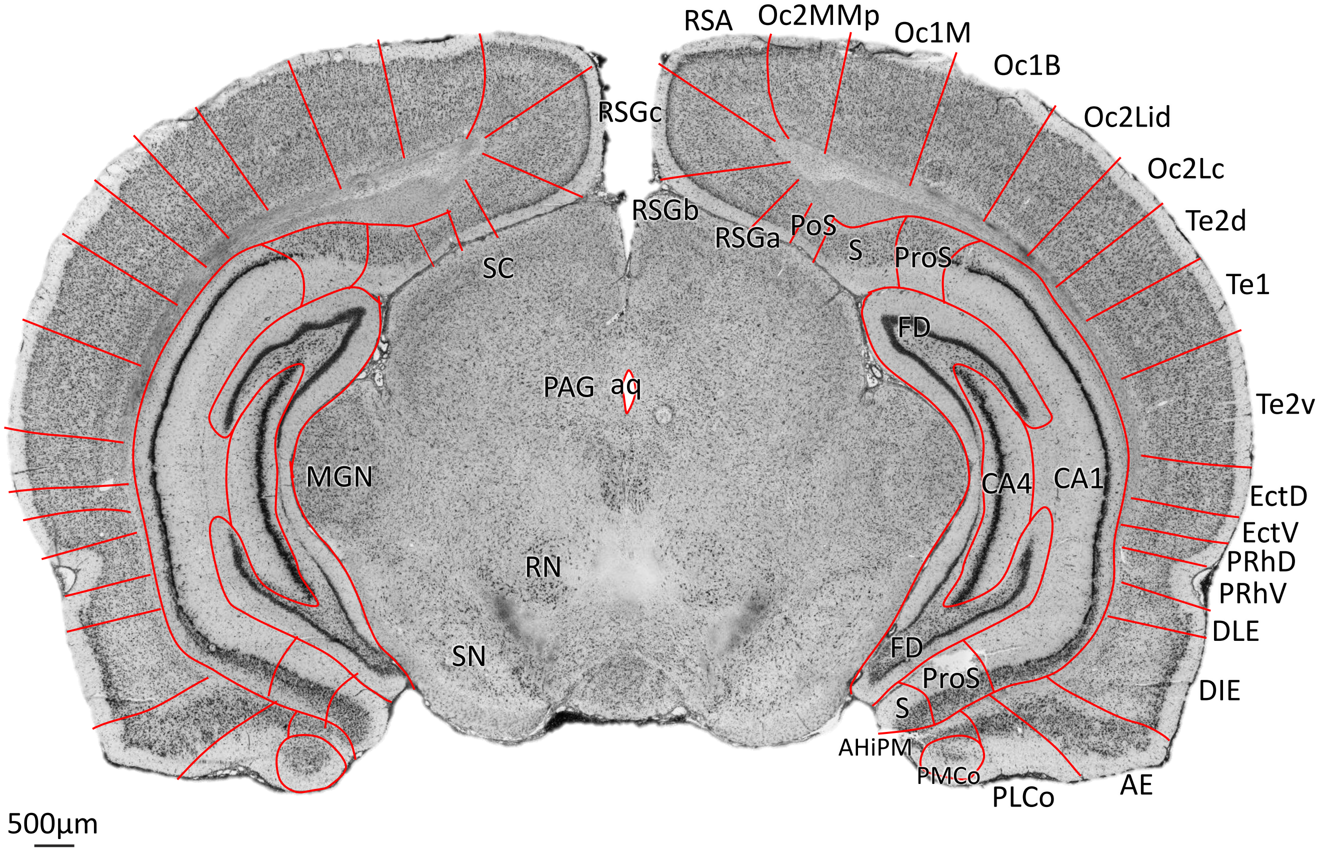
Section 850
RSA
Oc2MMp
Oc1M
Oc1B
Oc2Lid
Oc2Lc
Te2d
Te1
Te2v
RSGc
RSGb
RSGa
PoS
S
ProS
SC
FD
PAG
aq
MGN
CA4
CA1
EctD
EctV
PRhD
PRhV
DLE
DIE
RN
SN
FD
ProS
S
AHiPM
PMCo
PLCo
AE
500µm

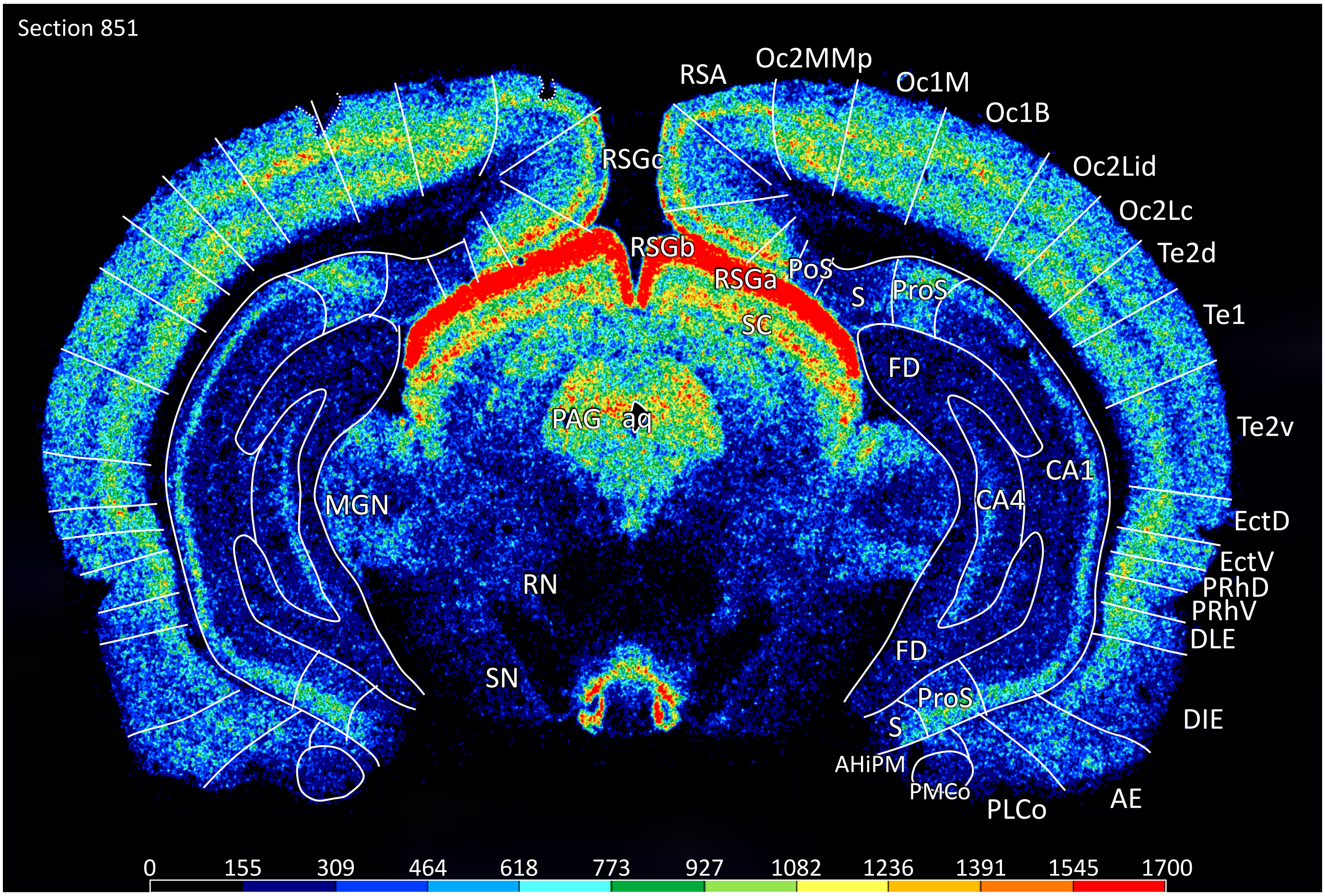
Section 851
RSA
Oc2MMp
Oc1M
Oc1B
Oc2Lid
Oc2Lc
Te2d
Te1
Te2v
RSGc
RSGb
RSGa
PoS
S
ProS
SC
FD
PAG
aq
MGN
CA1
CA4
EctD
EctV
PRhD
PRhV
DLE
DIE
RN
SN
FD
ProS
S
AHiPM
PMCo
PLCo
AE
0
155
309
464
618
773
927
1082
1236
1391
1545
1700

Level c29

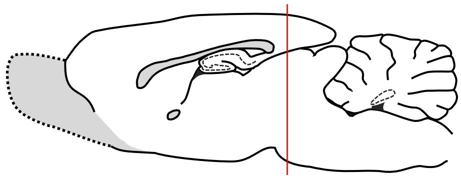

Section 880

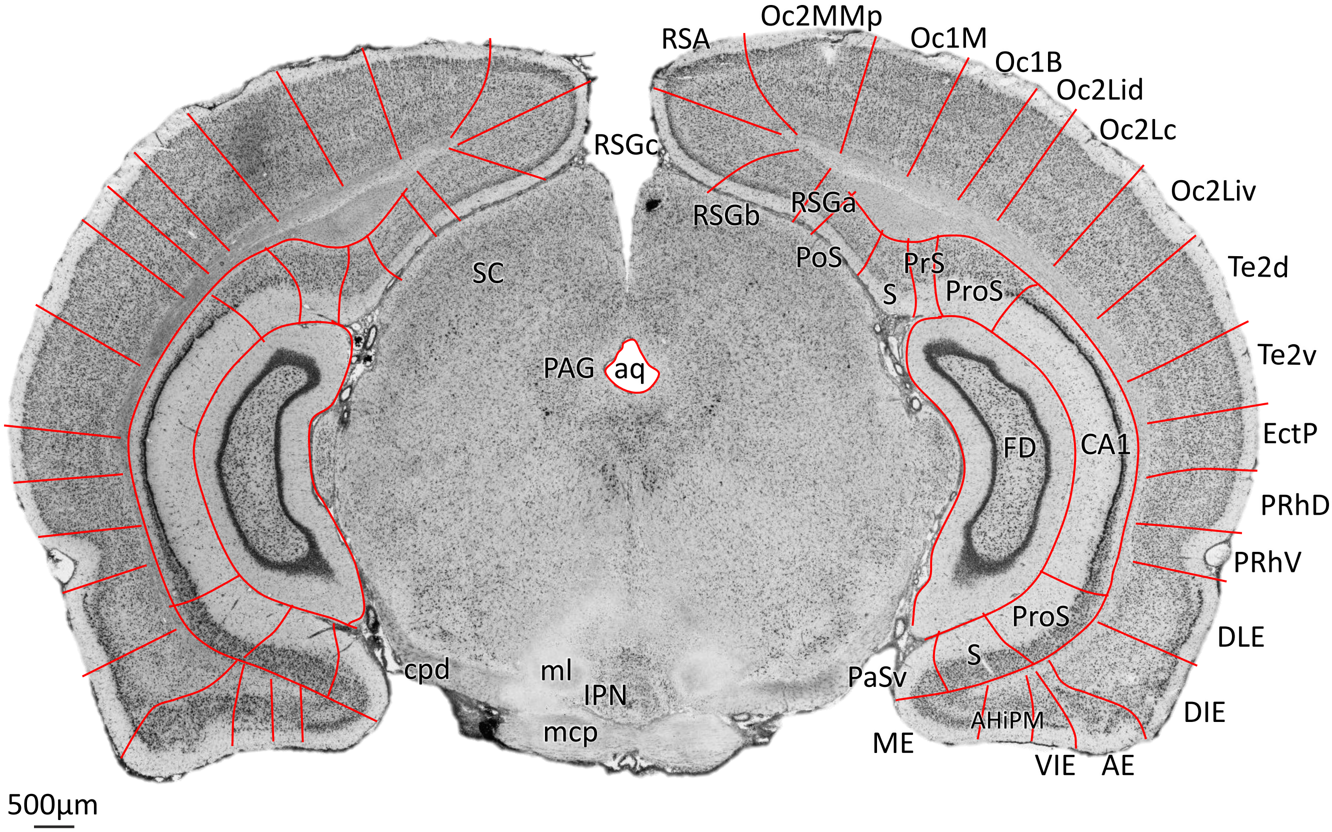

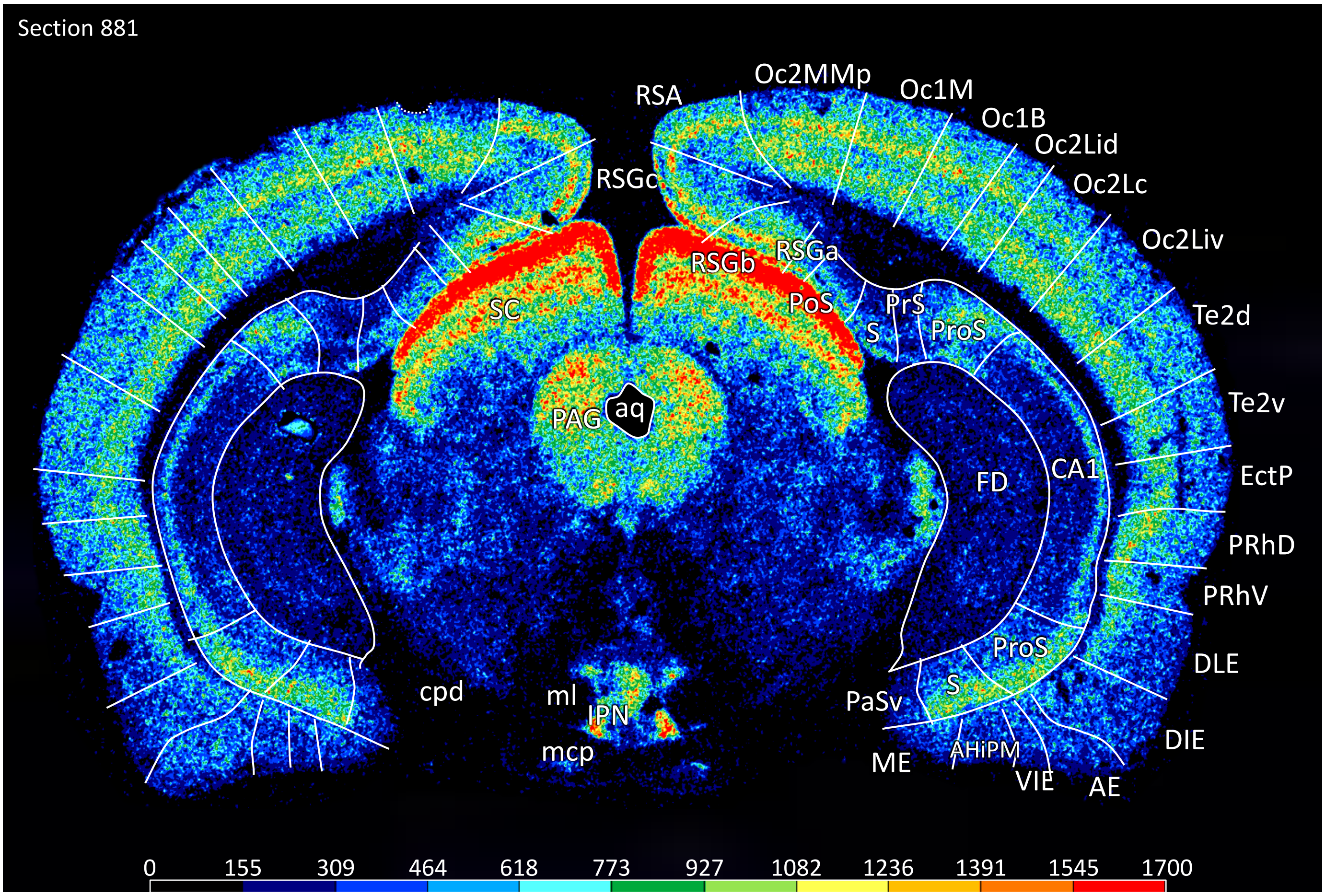

Level c30

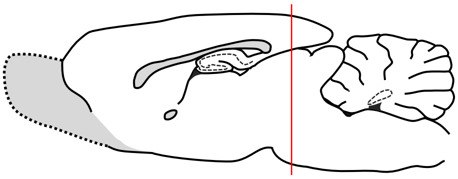

Section 904

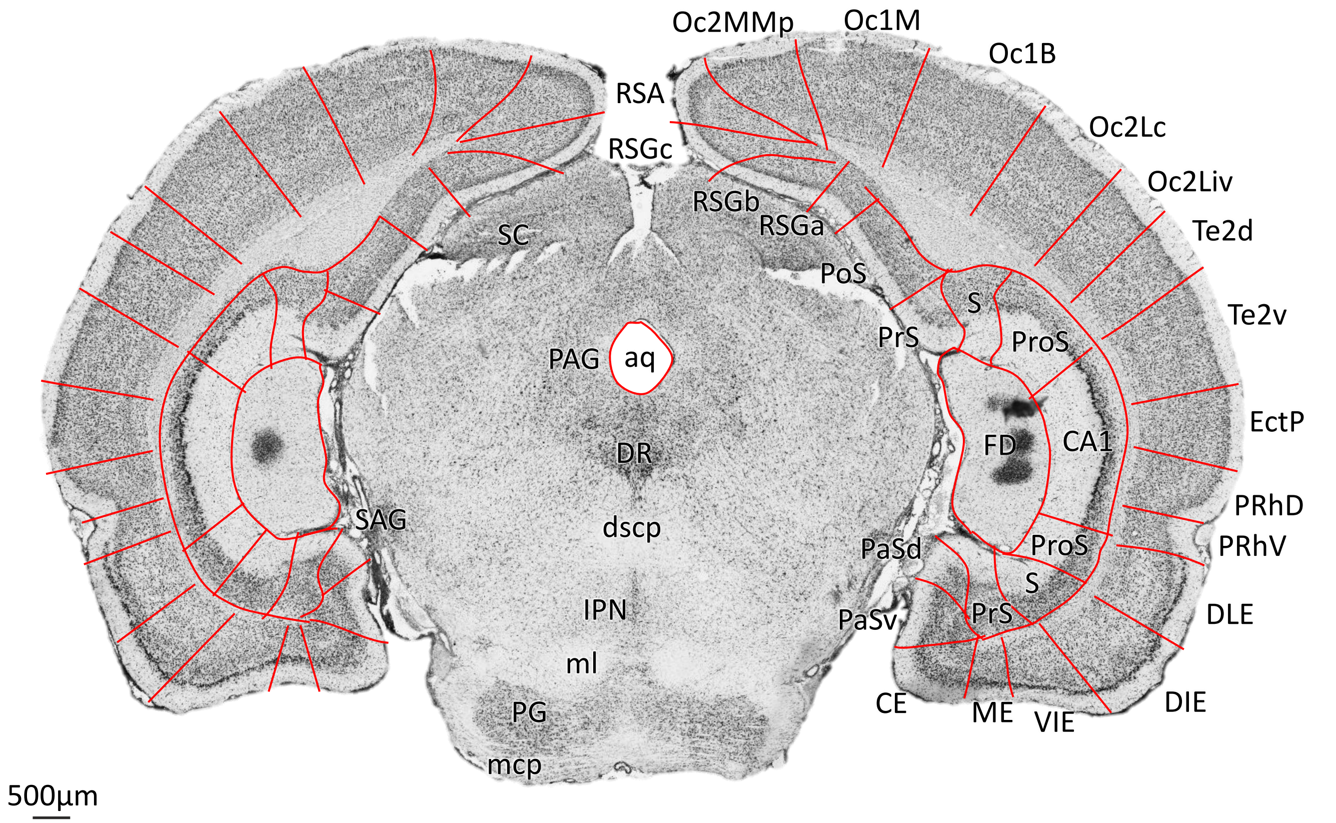

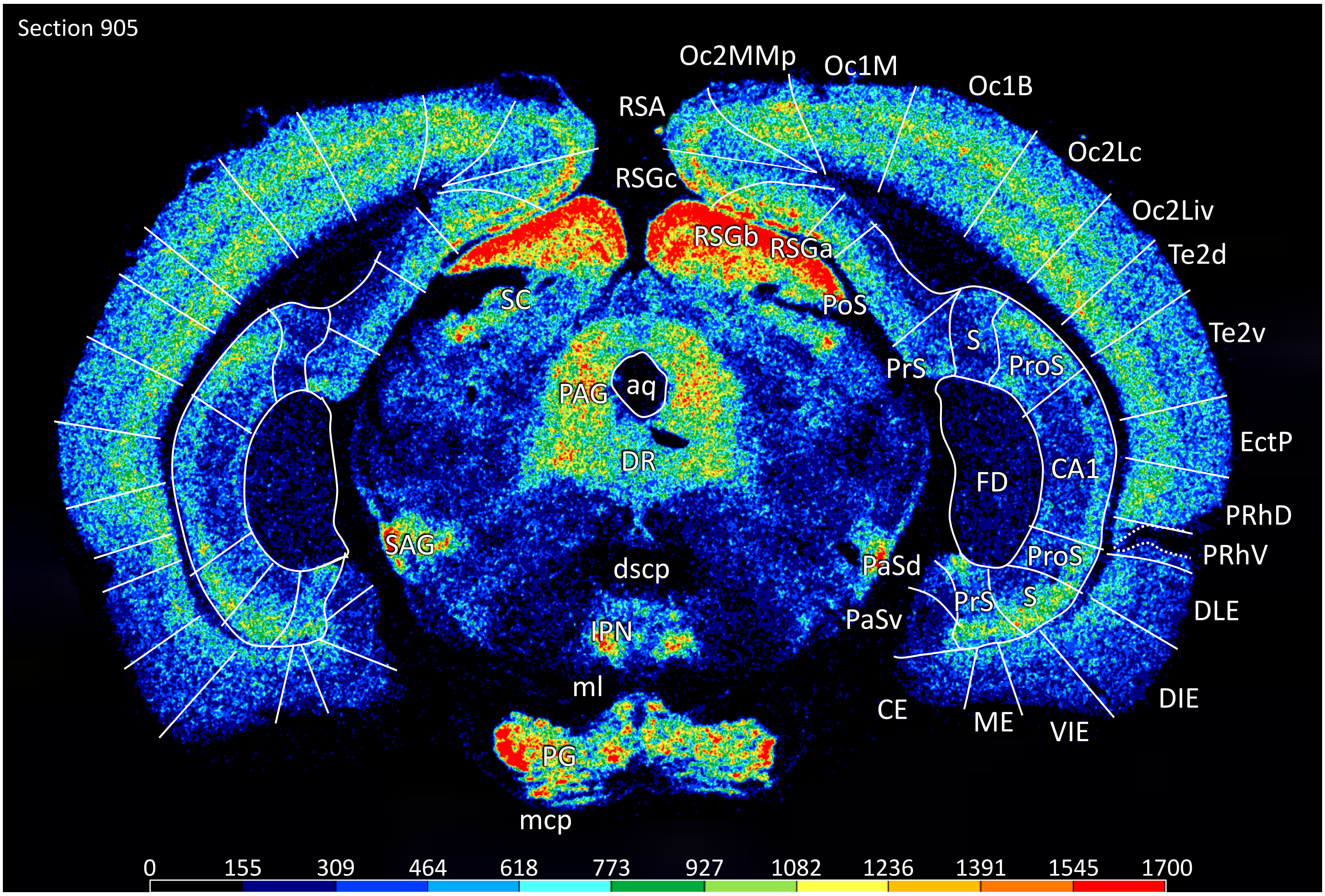

Level c31

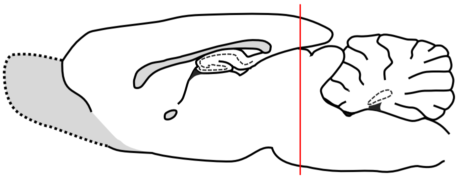

Section 925

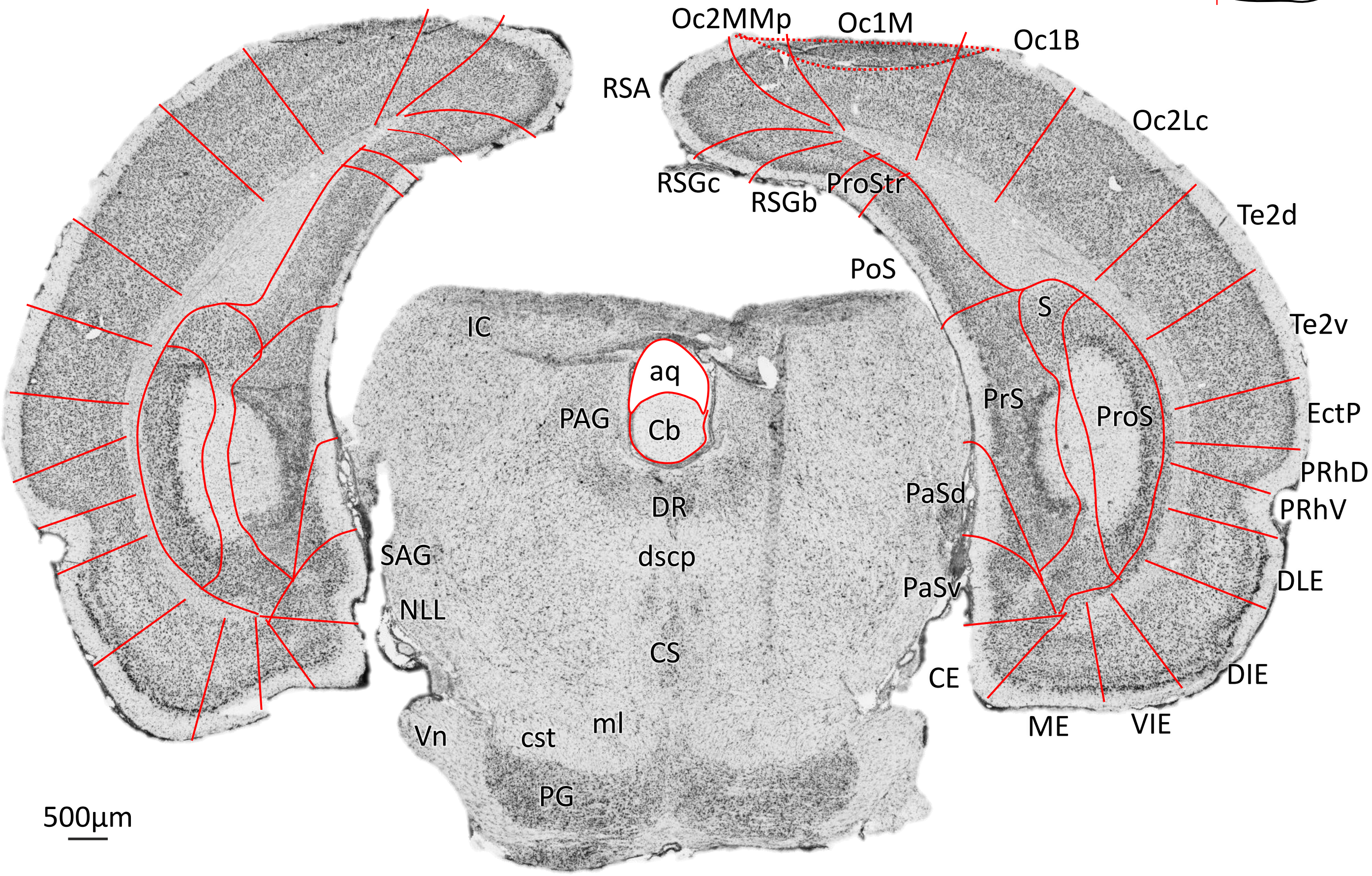

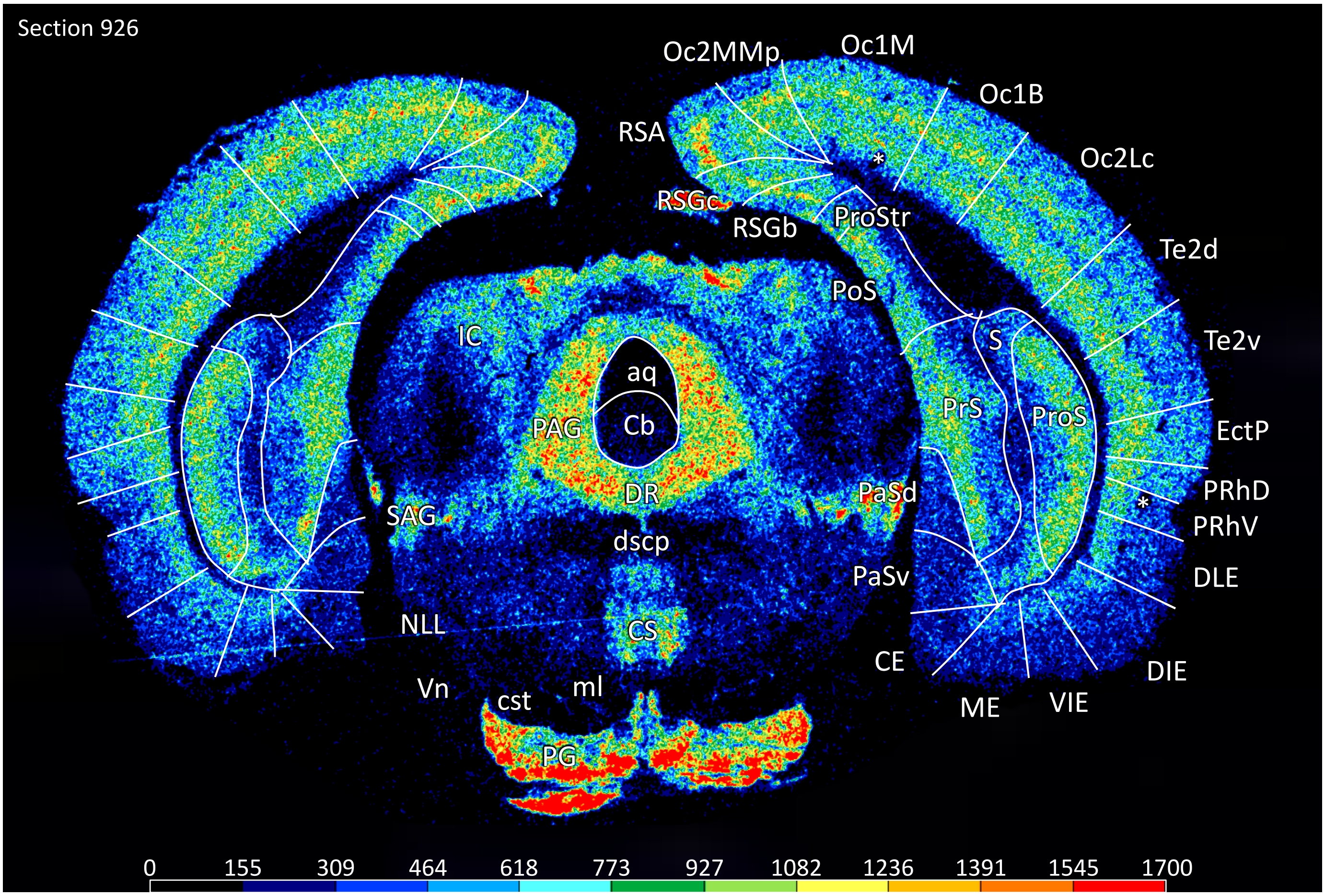

Level c32

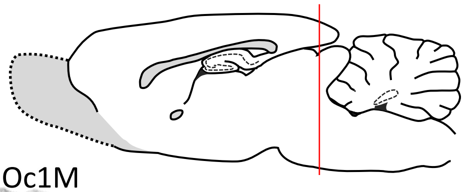

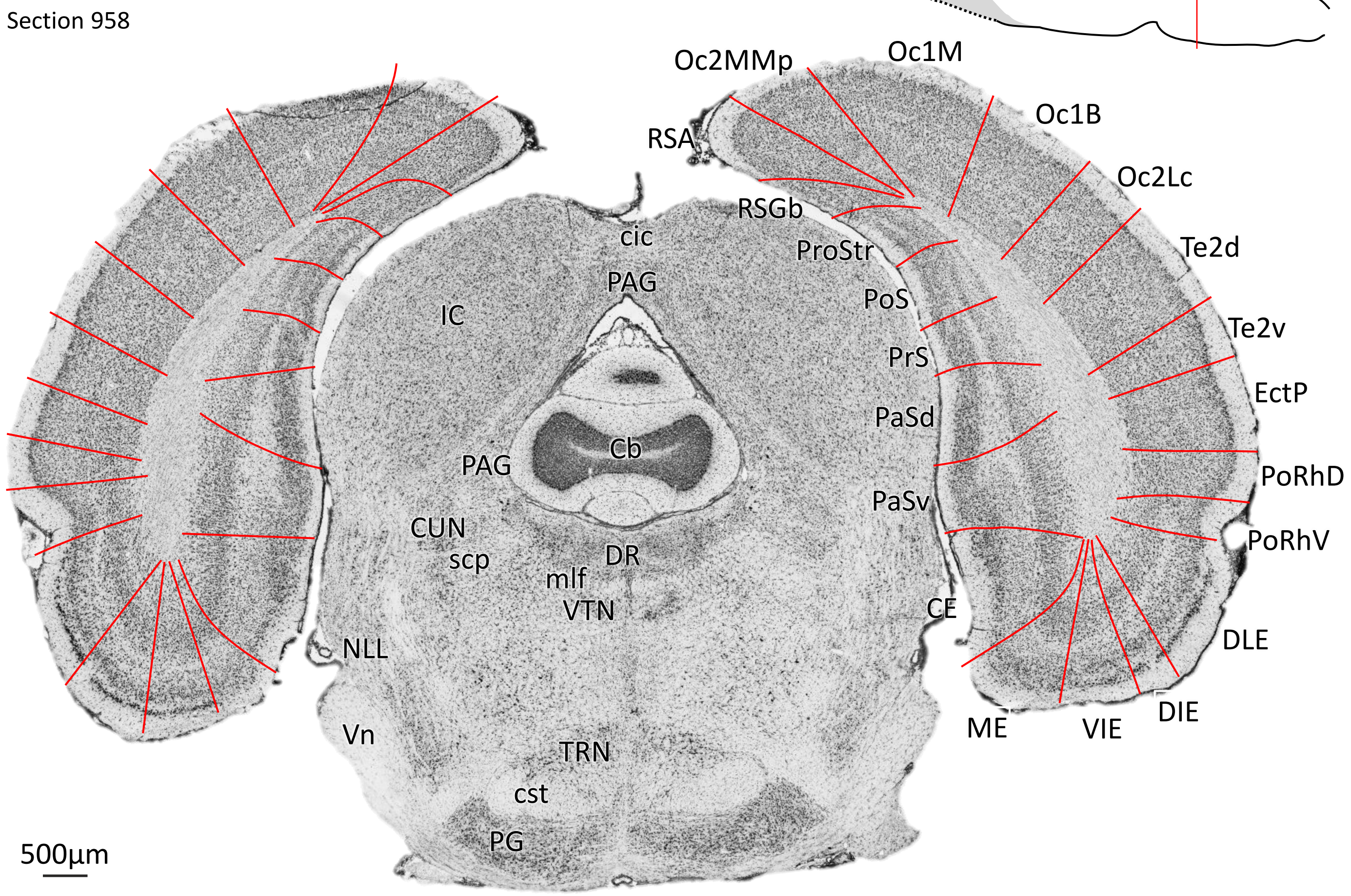
Section 958
Oc2MMp
Oc1M
Oc1B
RSA
Oc2Lc
RSGb
Te2d
cic
ProStr
PAG
PoS
Te2v
IC
PrS
EctP
PaSd
Cb
PAG
PoRhD
PaSv
CUN
PoRhV
scp
DR
mlf
VTN
CE
DLE
NLL
DIE
ME
VIE
Vn
TRN
cst
PG
500µm

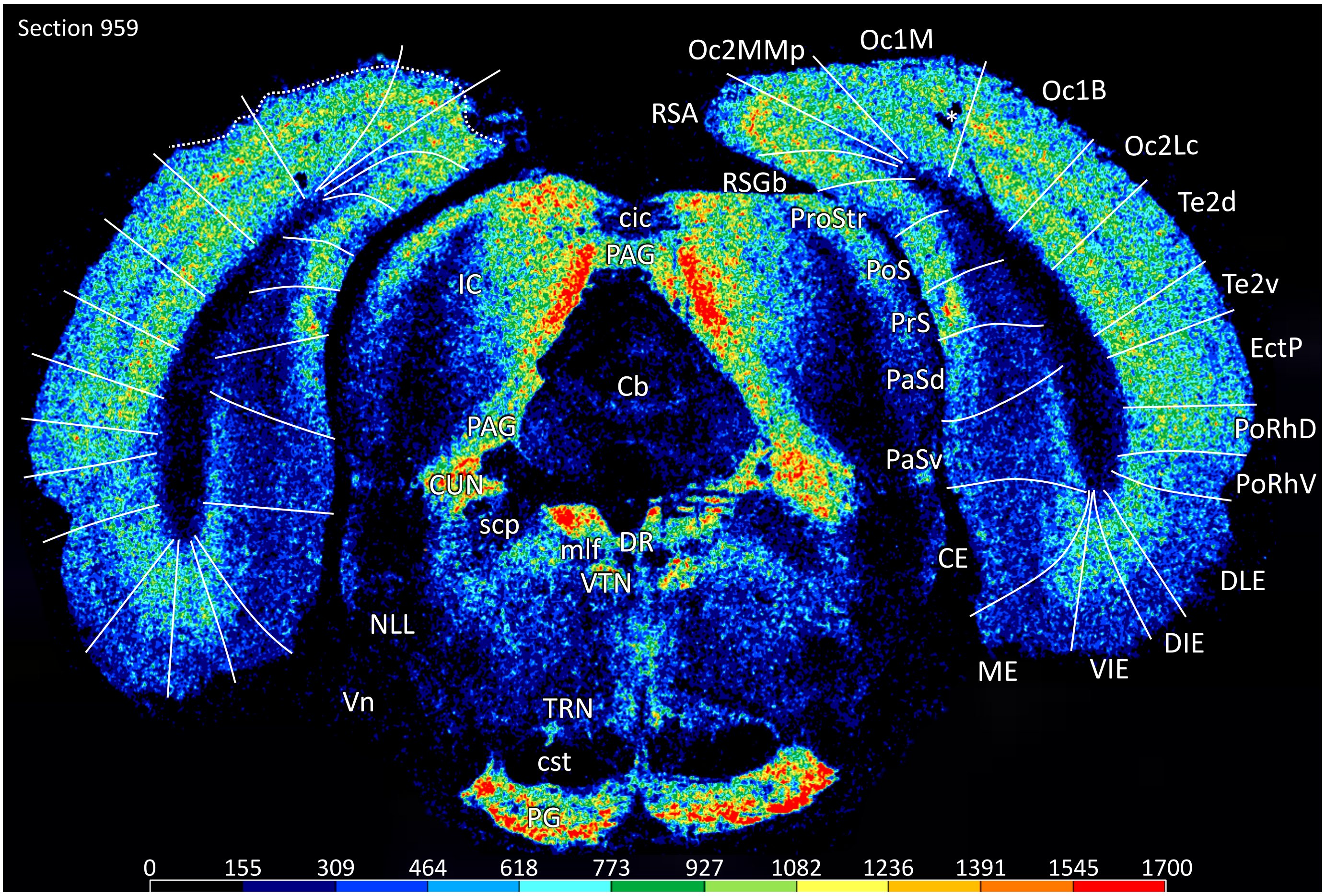
Section 959
Oc2MMp
Oc1M
Oc1B
RSA
Oc2Lc
RSGb
cic
Te2d
ProStr
PAG
PoS
IC
Te2v
PrS
EctP
PaSd
Cb
PoRhD
PAG
PaSv
PoRhV
CUN
scp
DR
mlf
VTN
CE
DLE
NLL
DIE
ME
VIE
Vn
TRN
cst
PG
0
155
309
464
618
773
927
1082
1236
1391
1545
1700

Level c33

Section 991

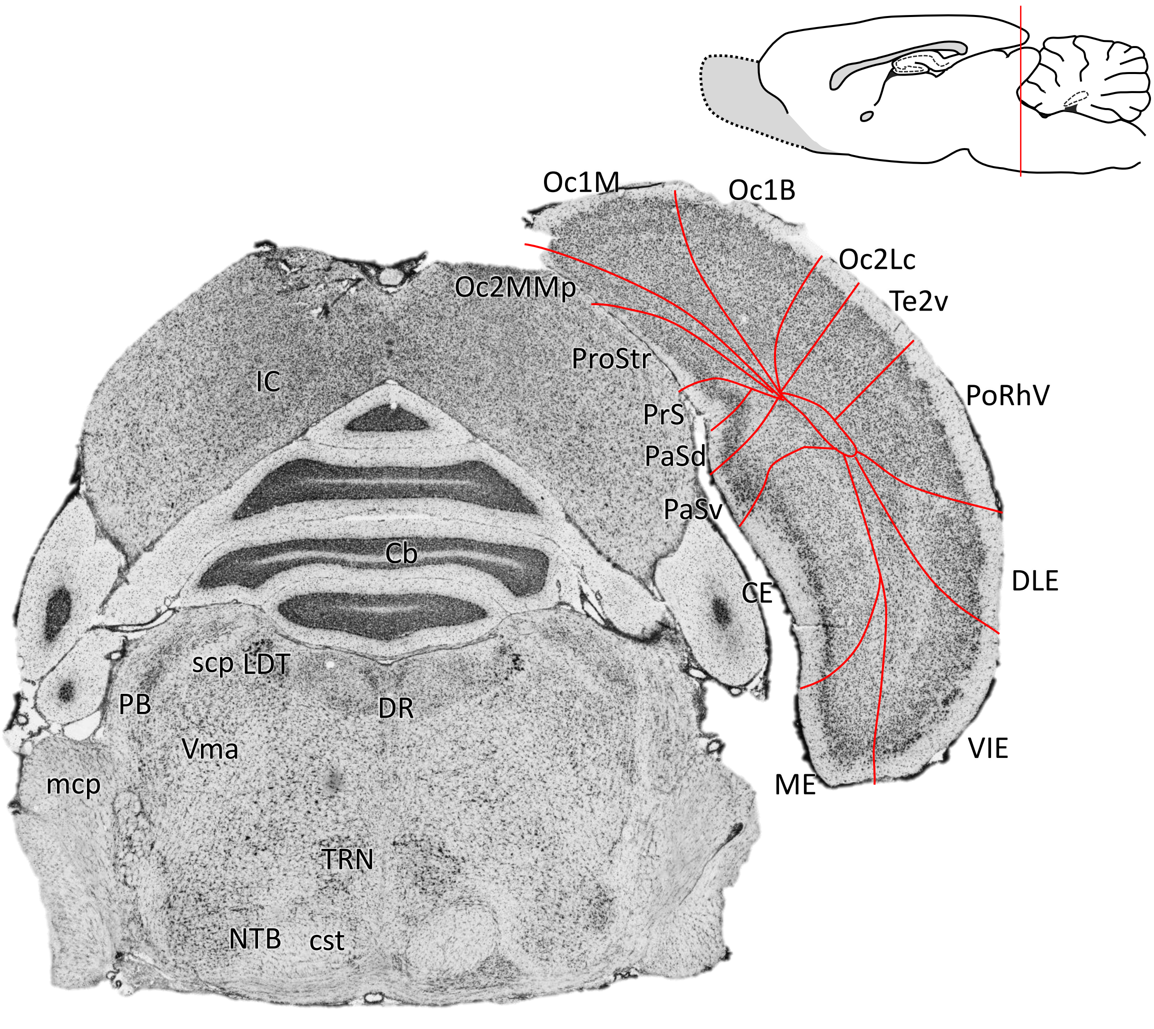

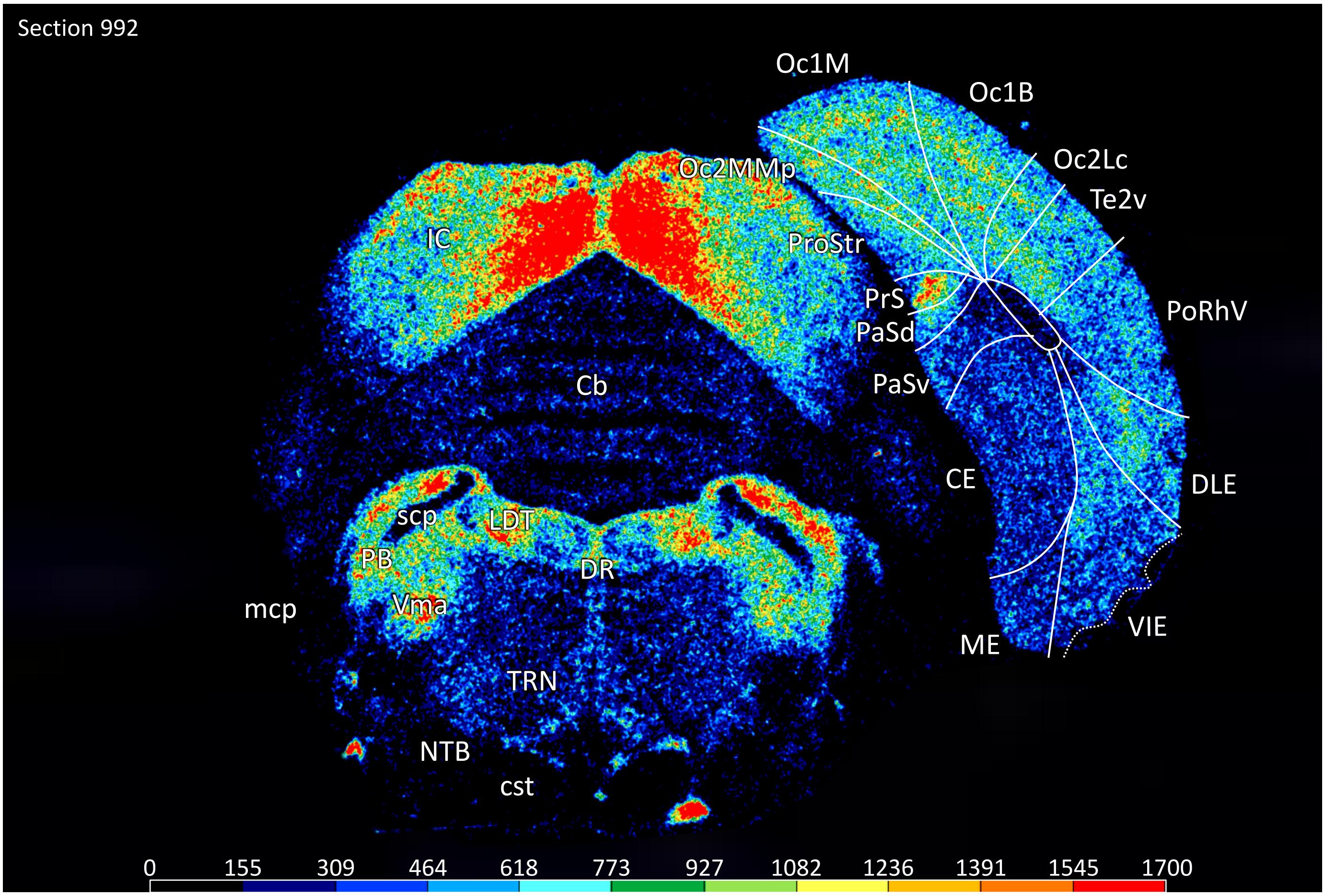

Level c34

Section 1021

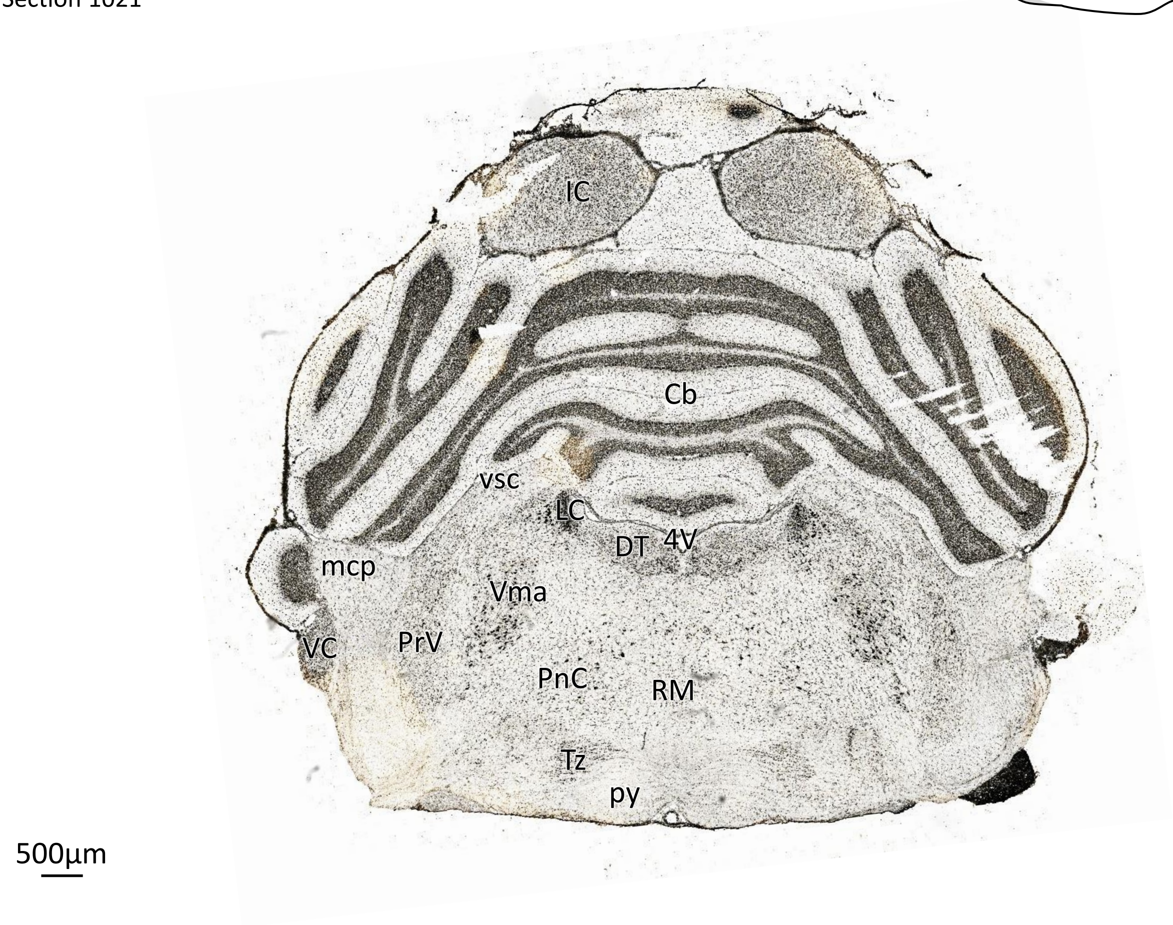

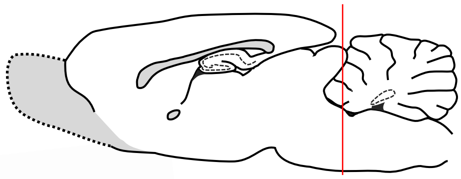

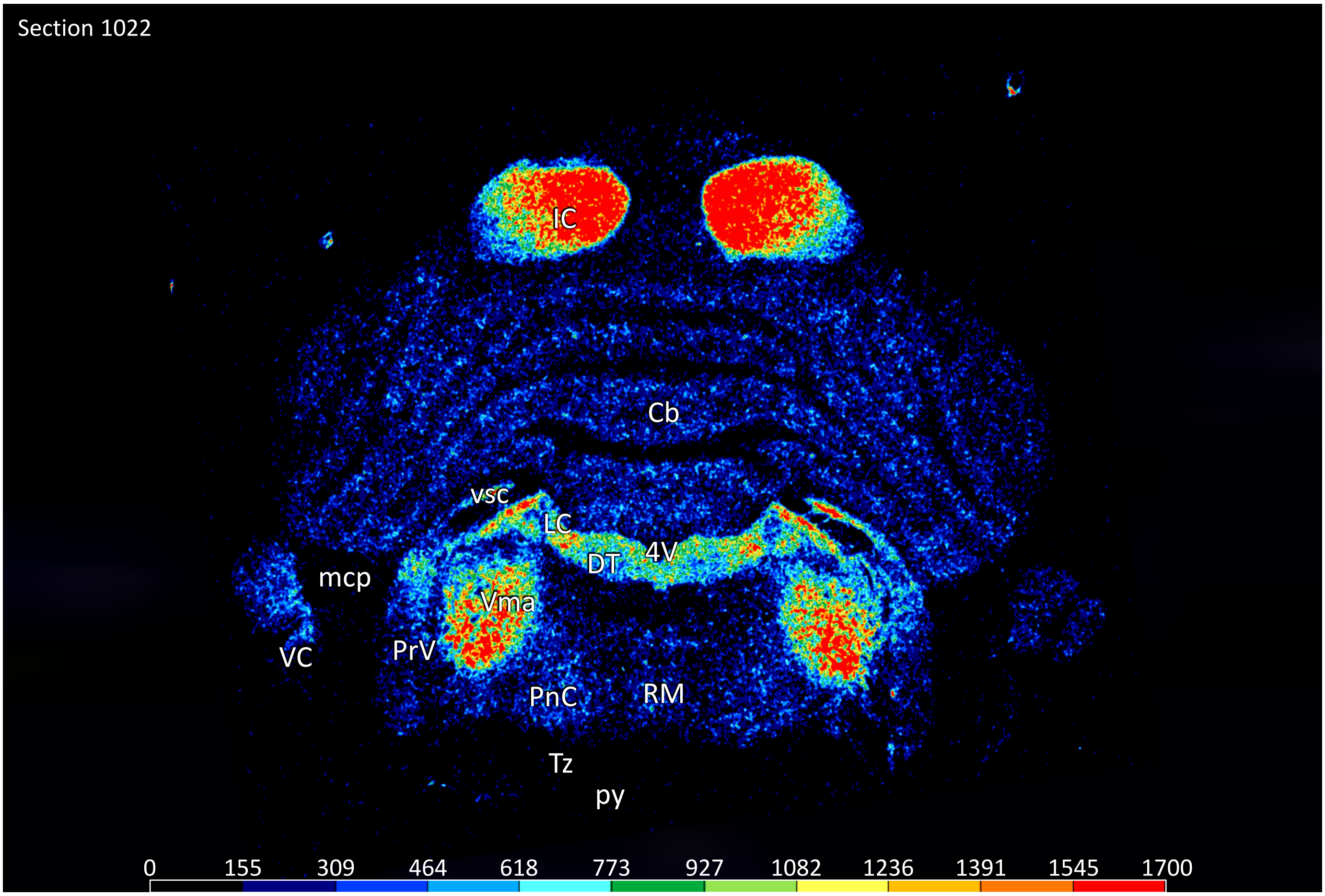

Level c35

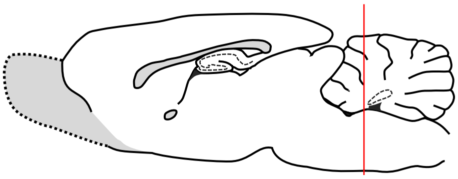

Section 1066

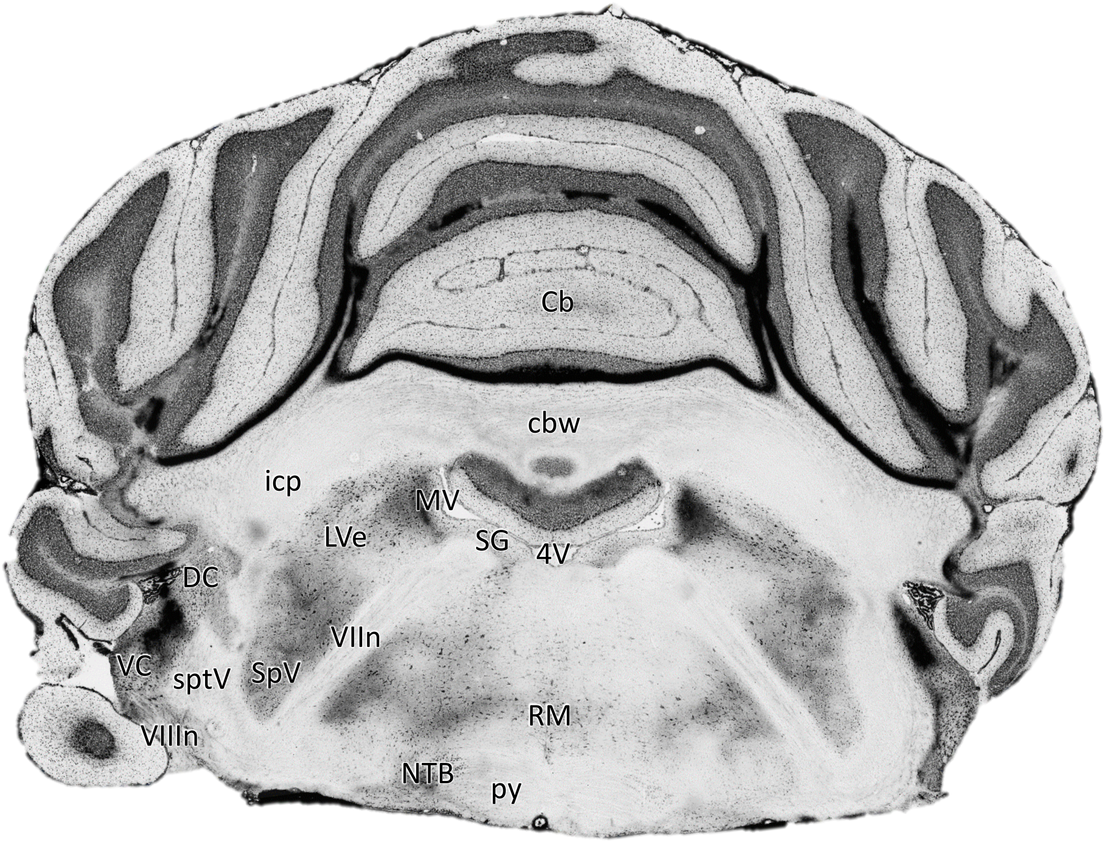

500µm

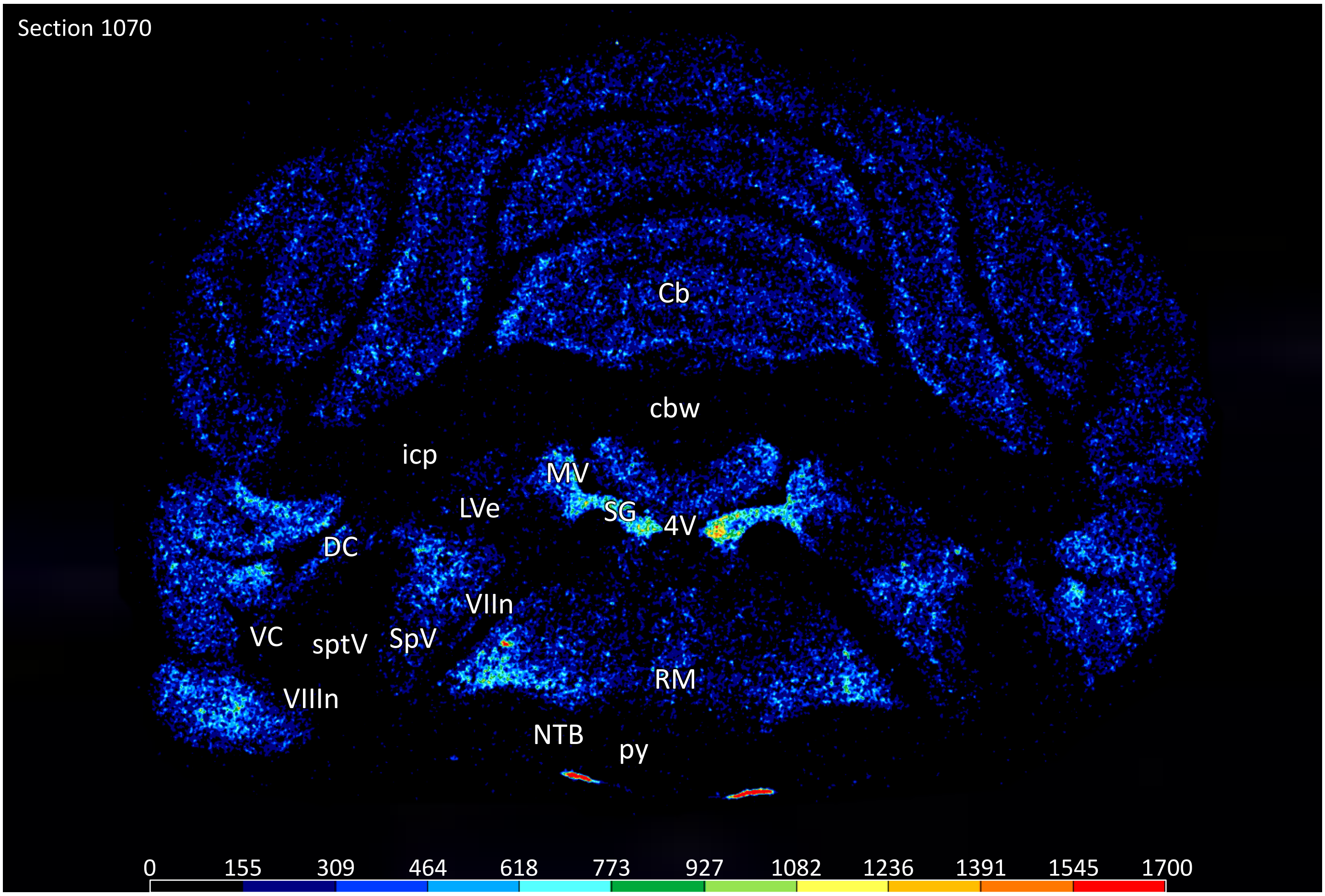

Level c36

Section 1145

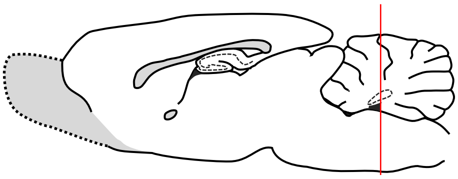

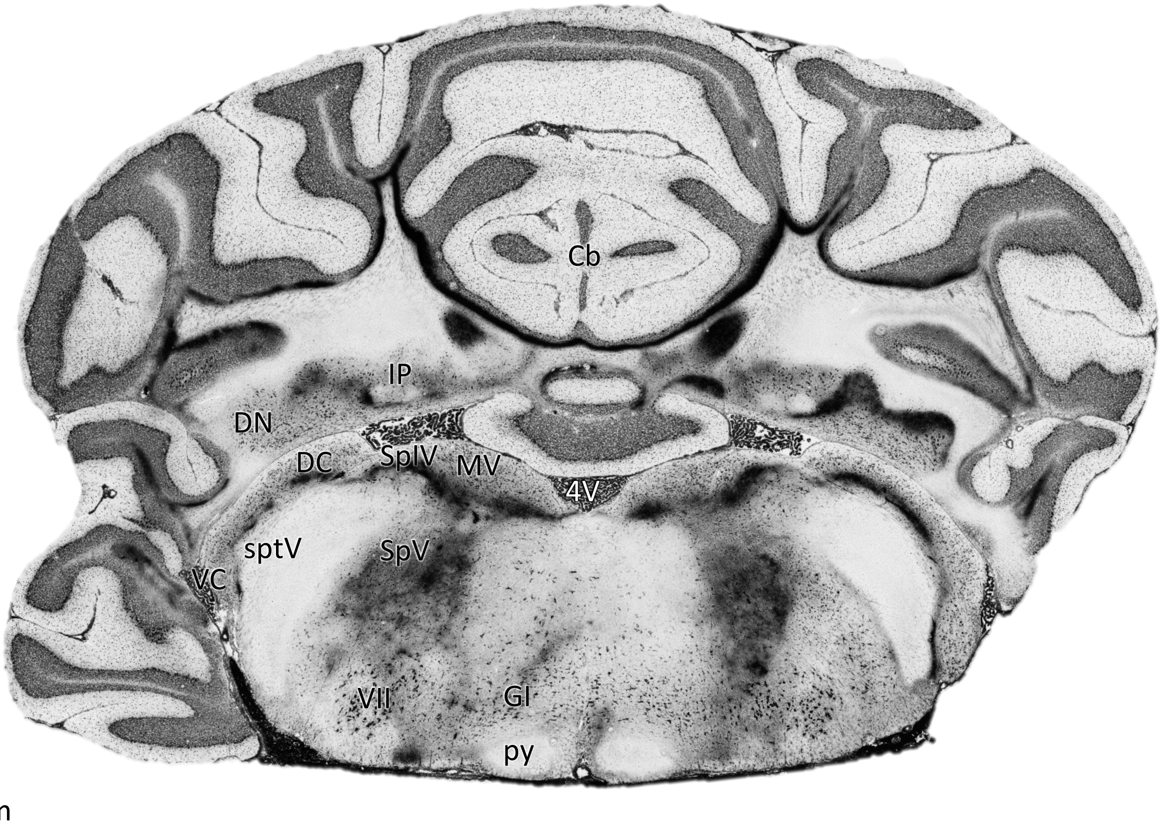

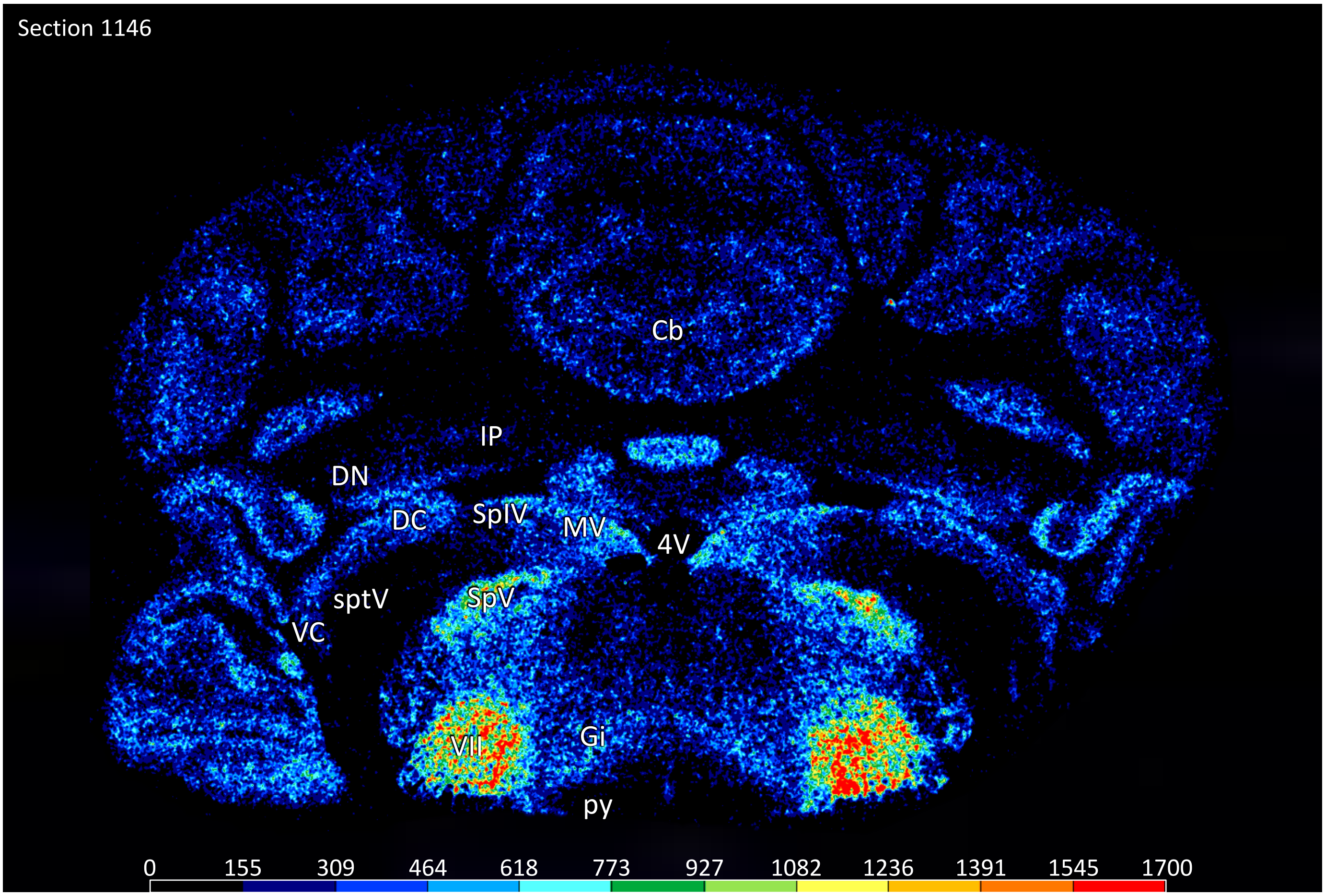

Level c37

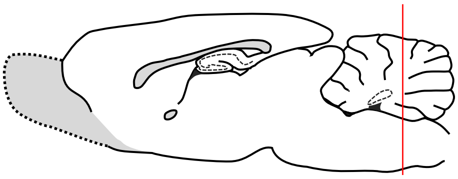

Section 1199

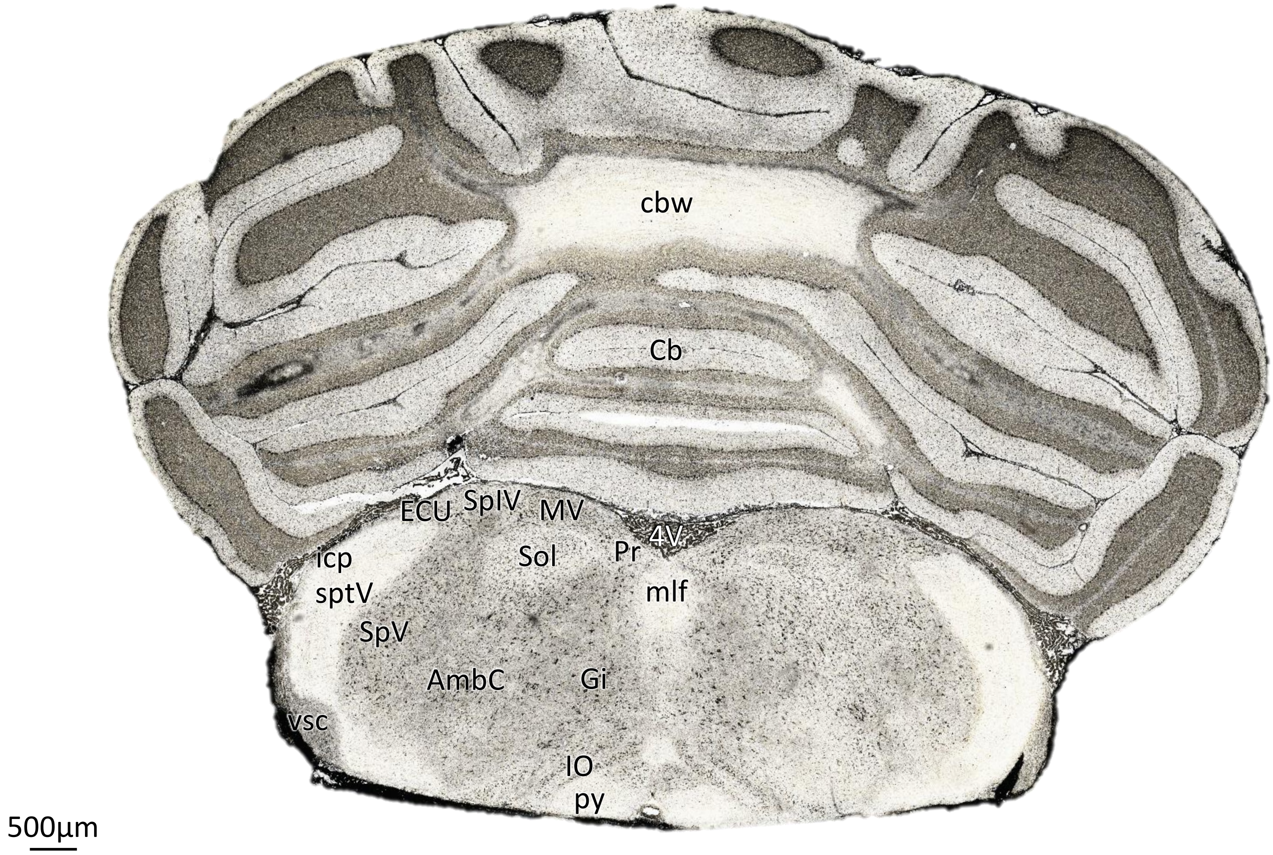

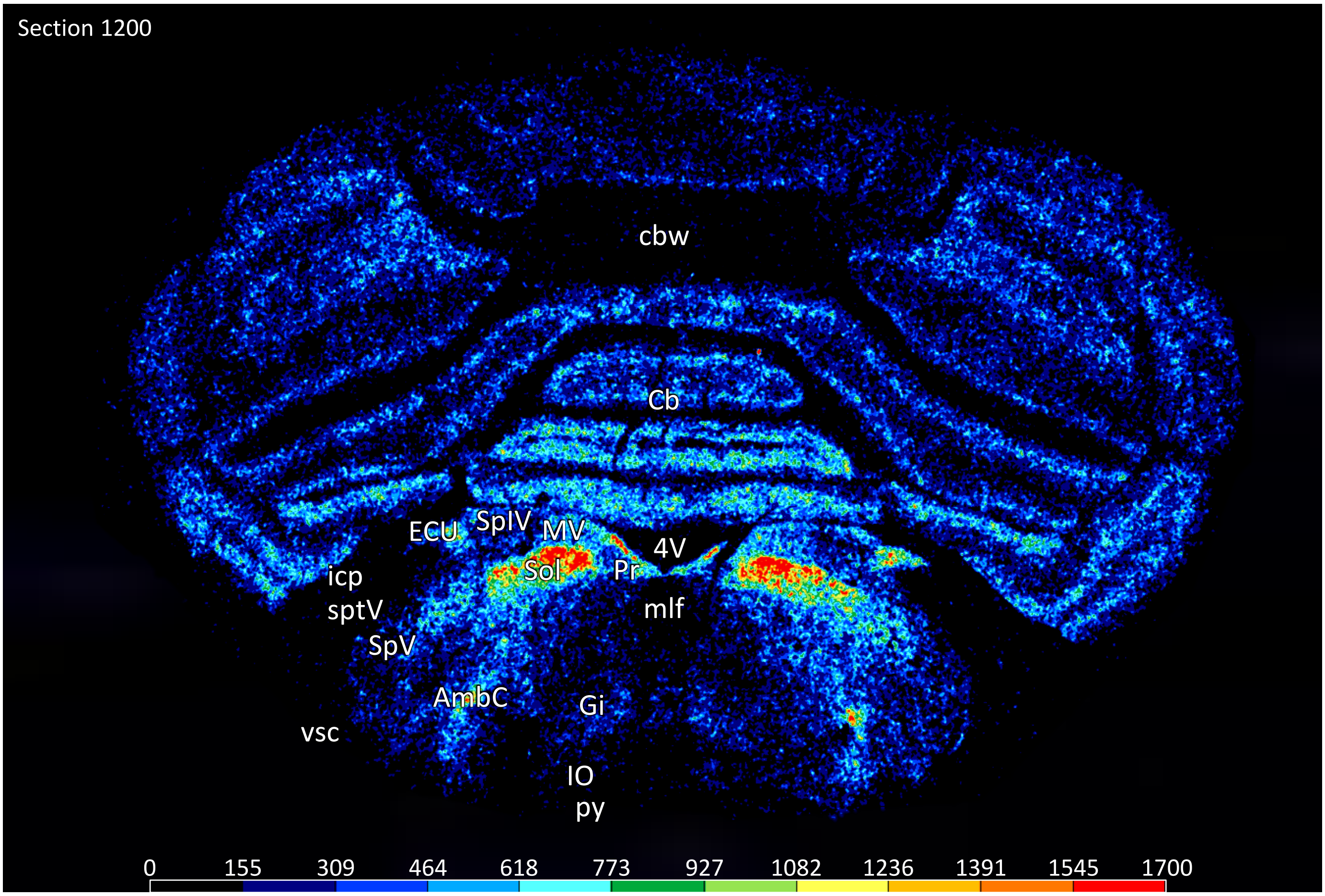

Level c38

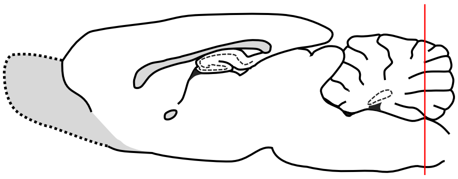

Section 1263

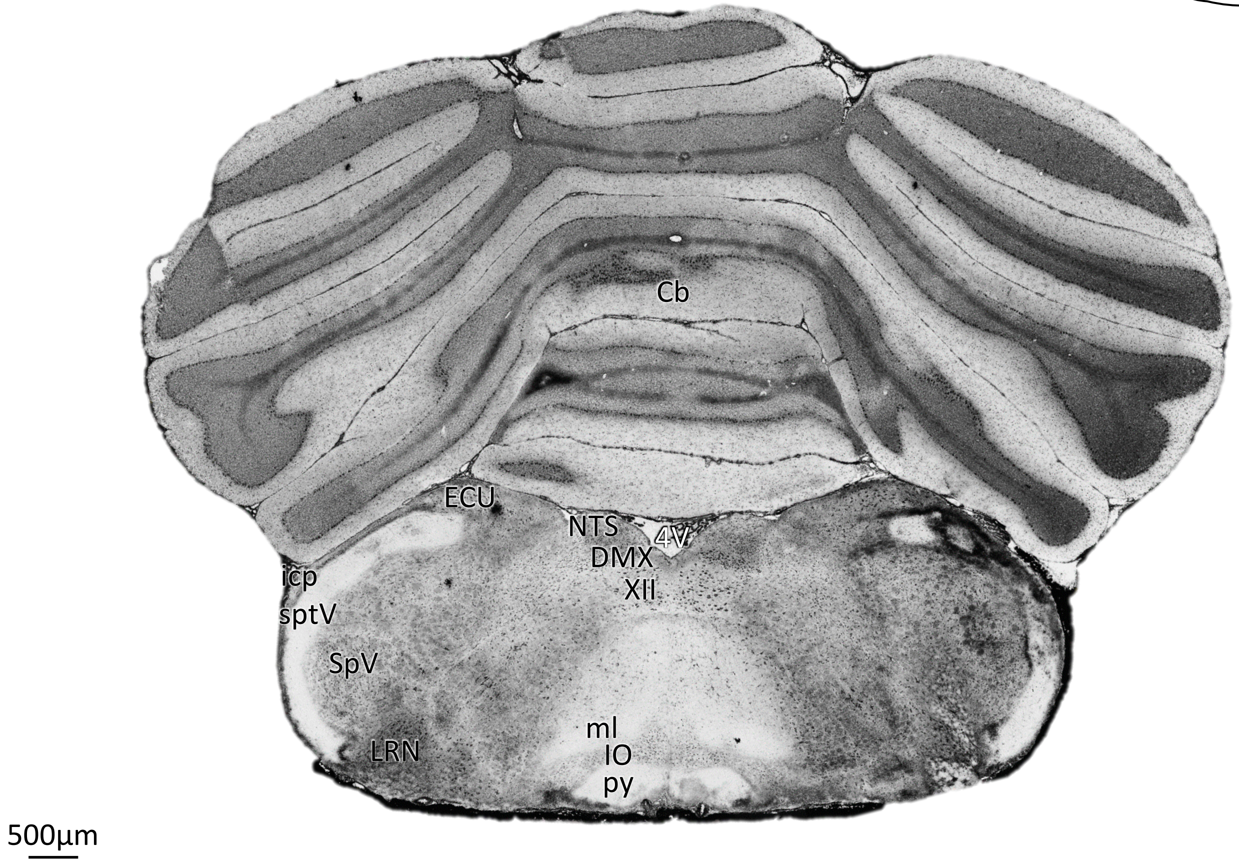

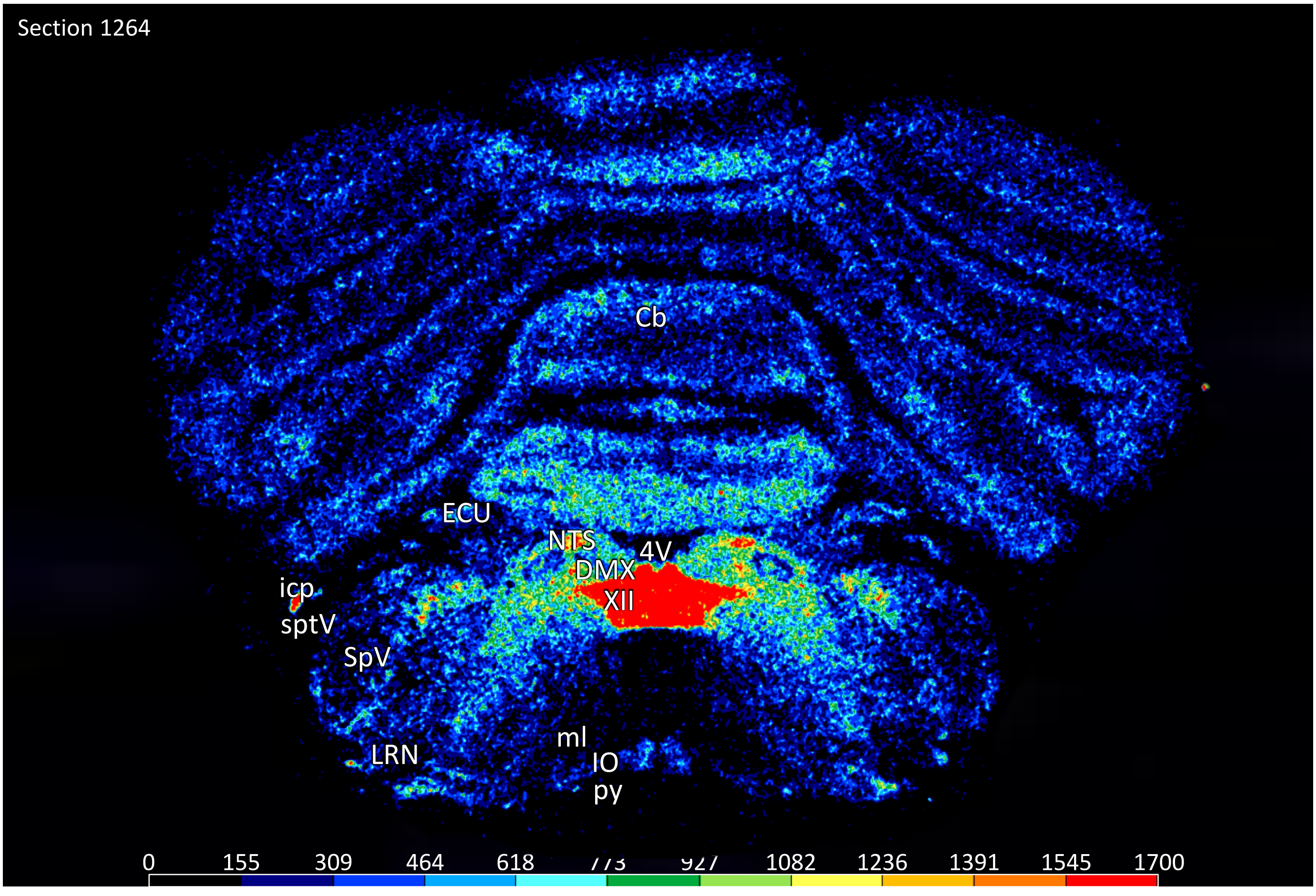

Level c39

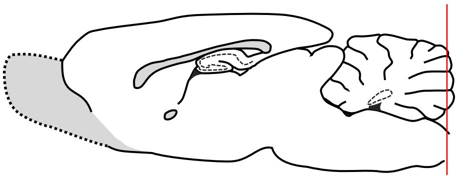

Section 1341

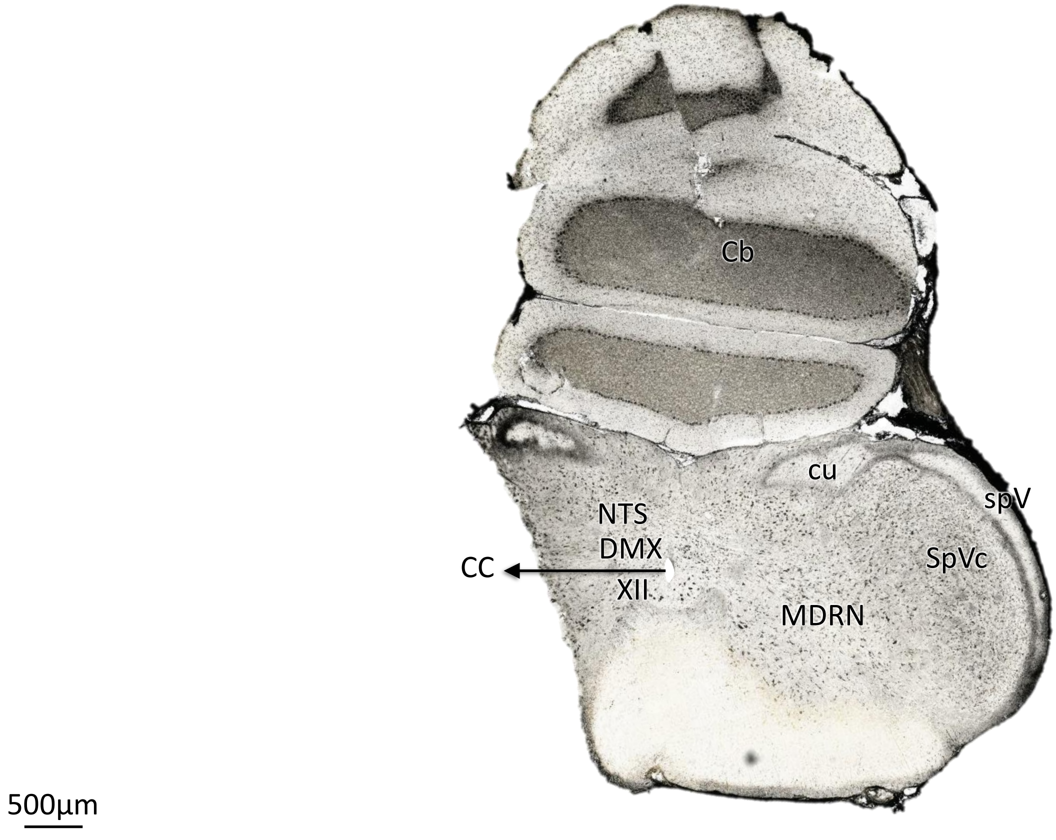

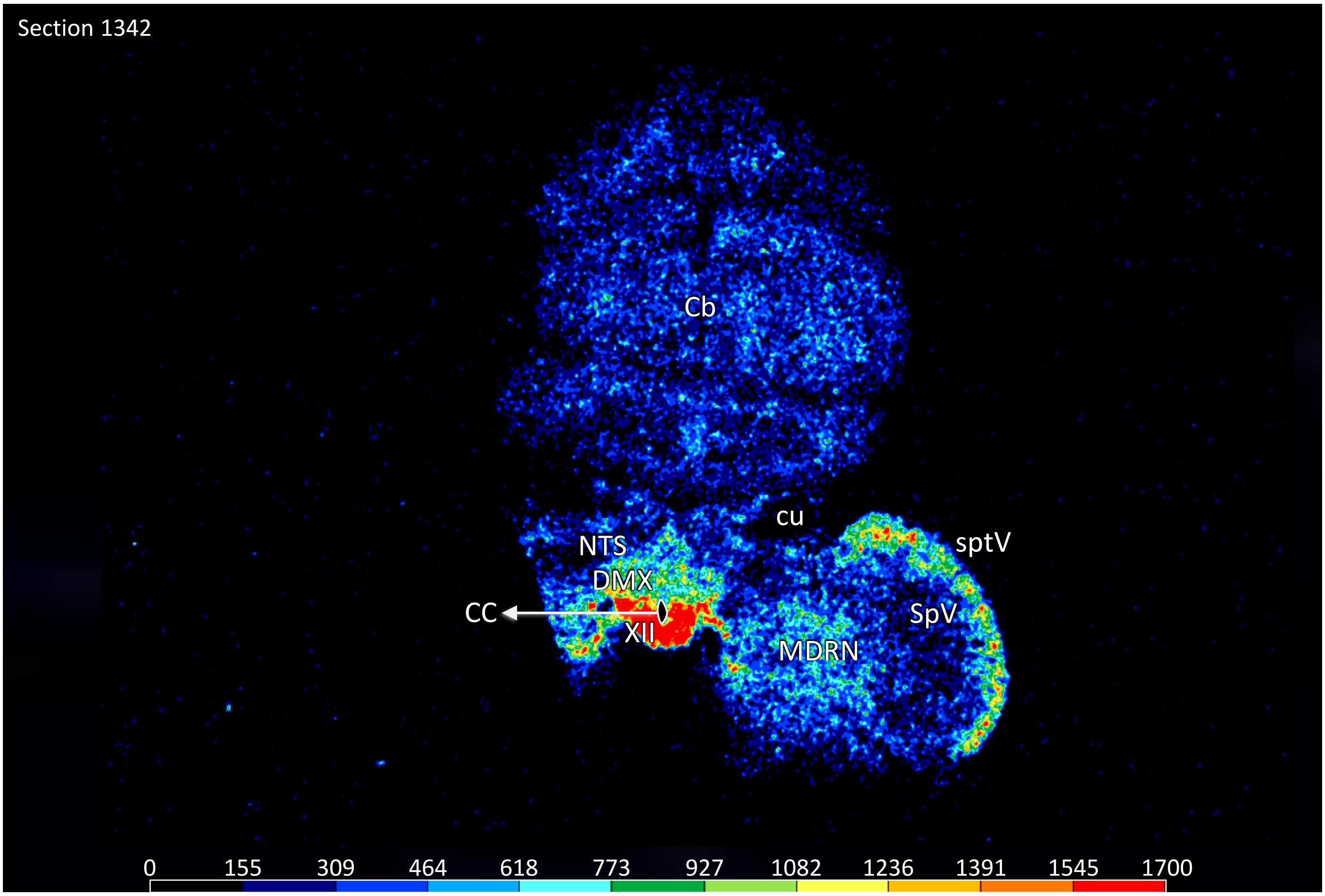

Sagittal series

Level s1

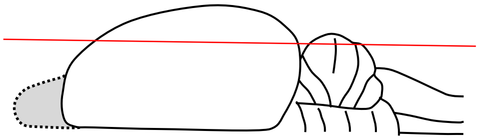

Section 93

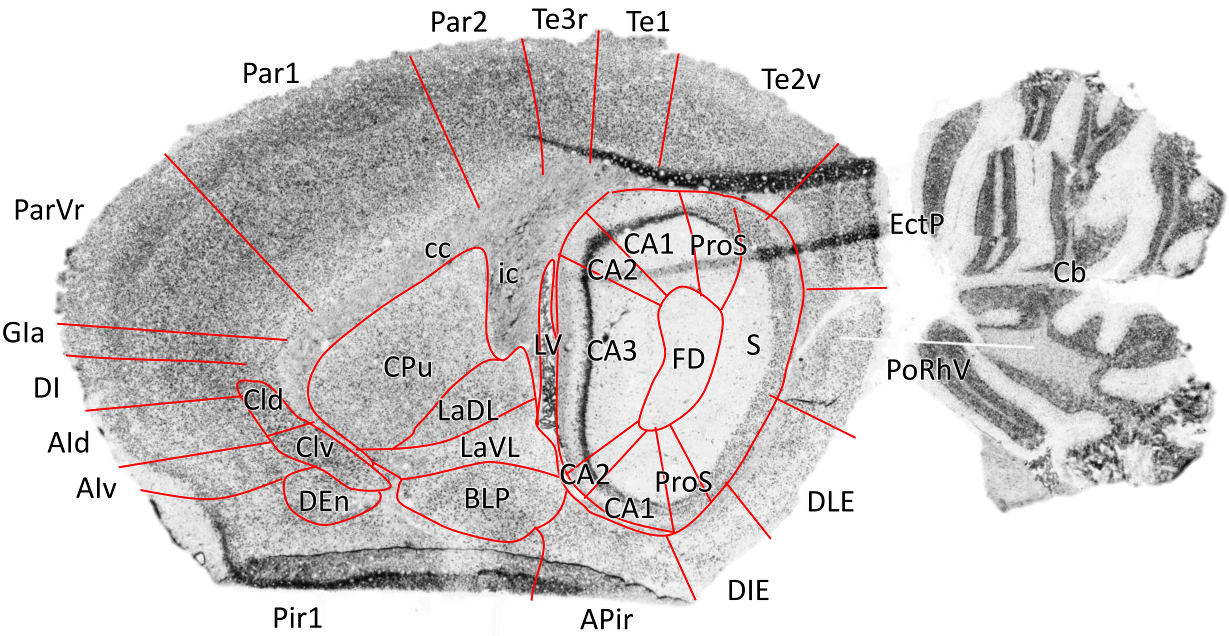

500µm

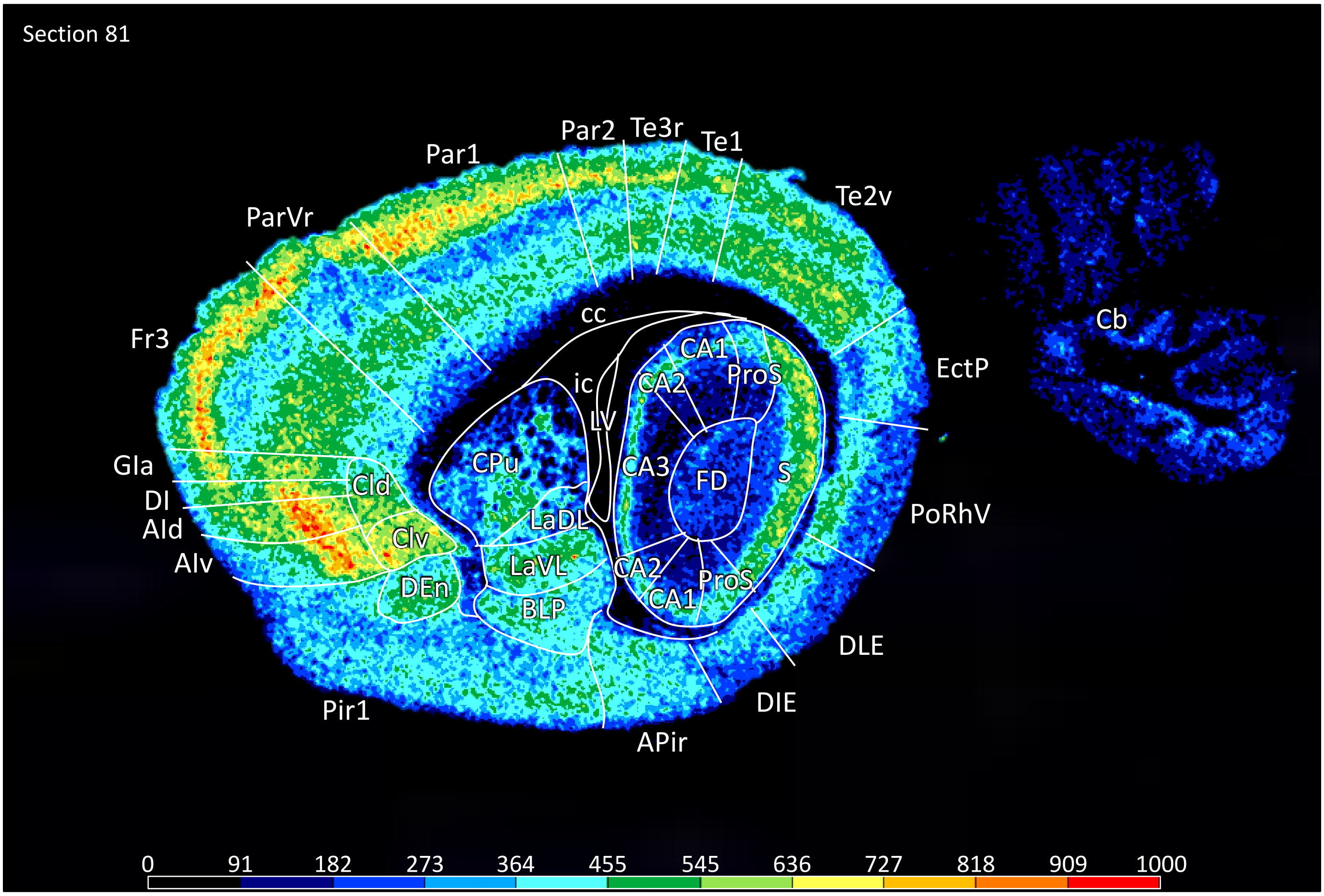

Level s2

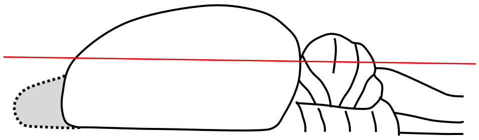

Section 117

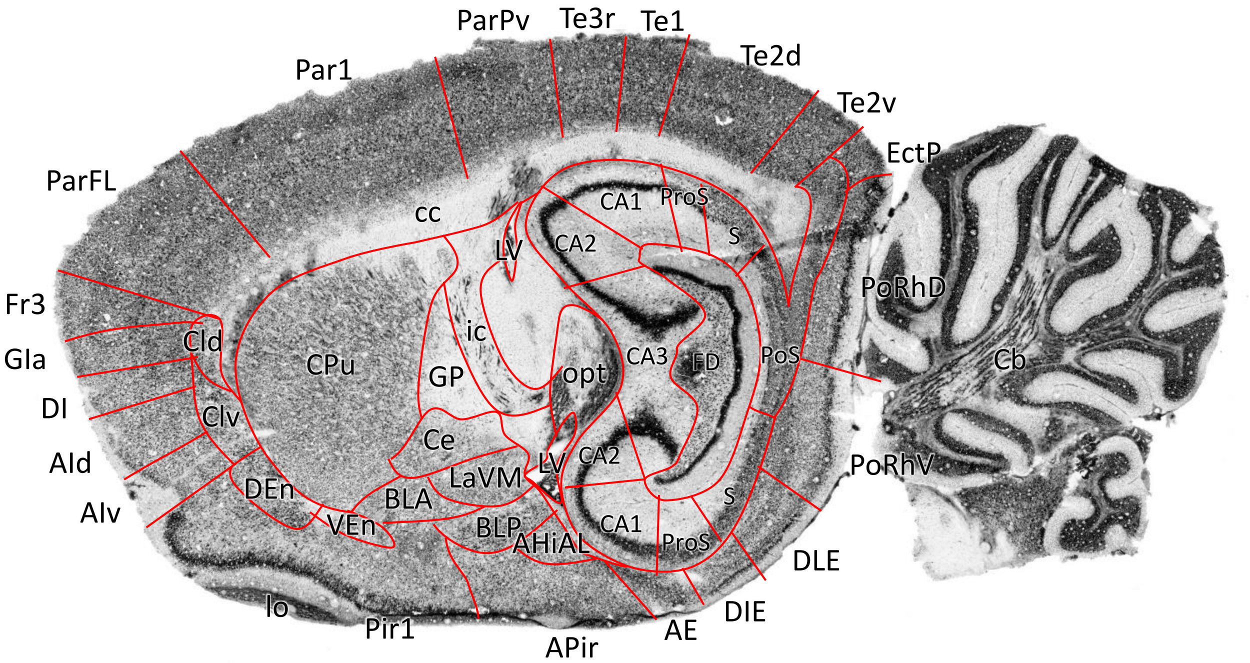

500µm

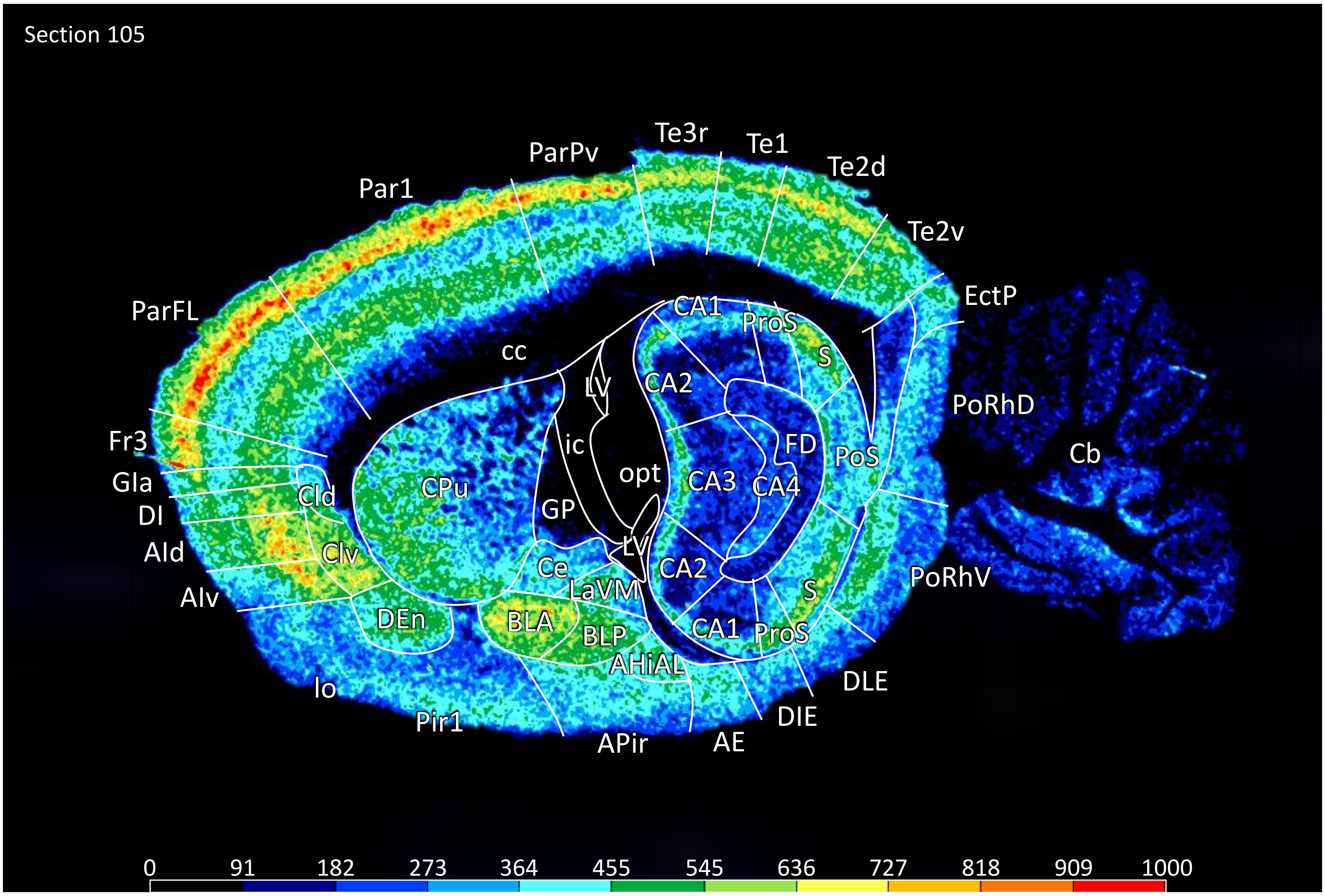

Level s3

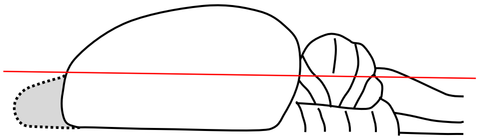

Section 141

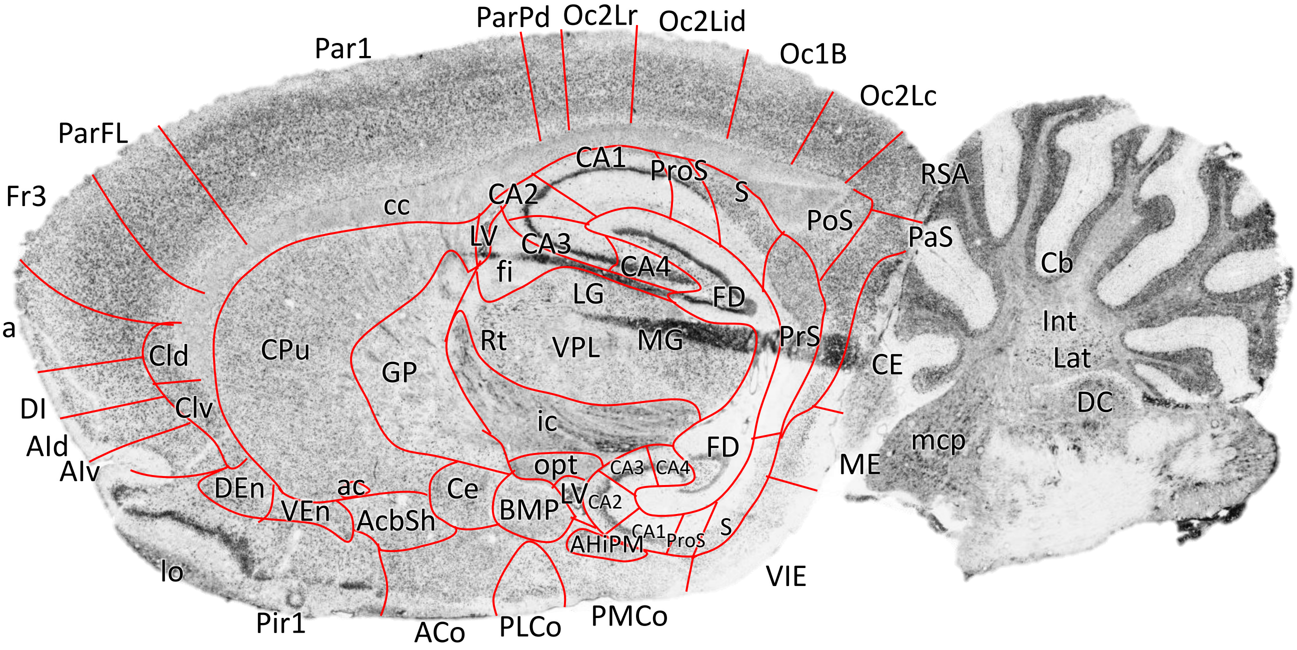

500µm

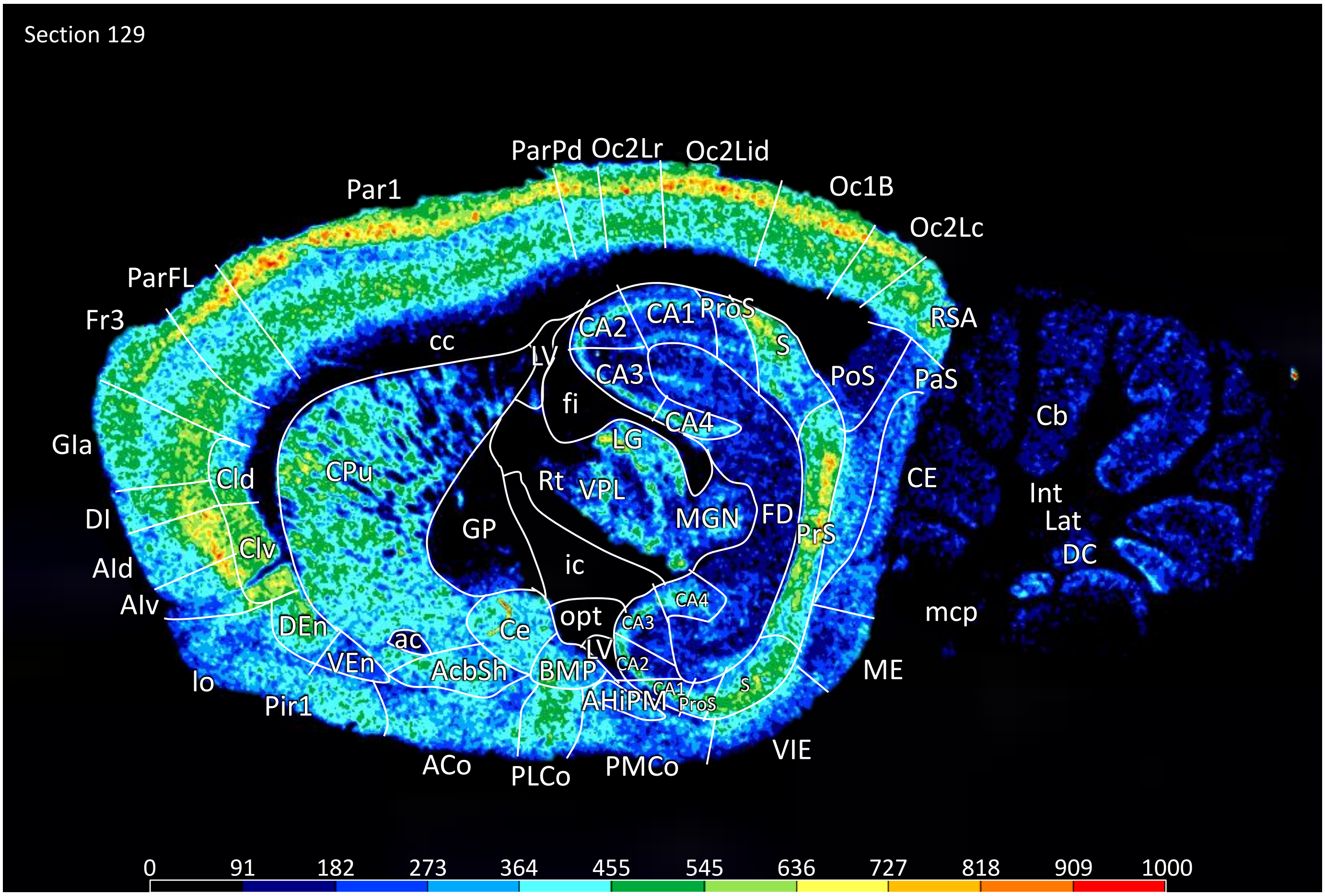

Level s4

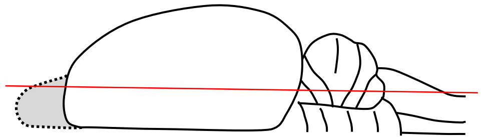

Section 237

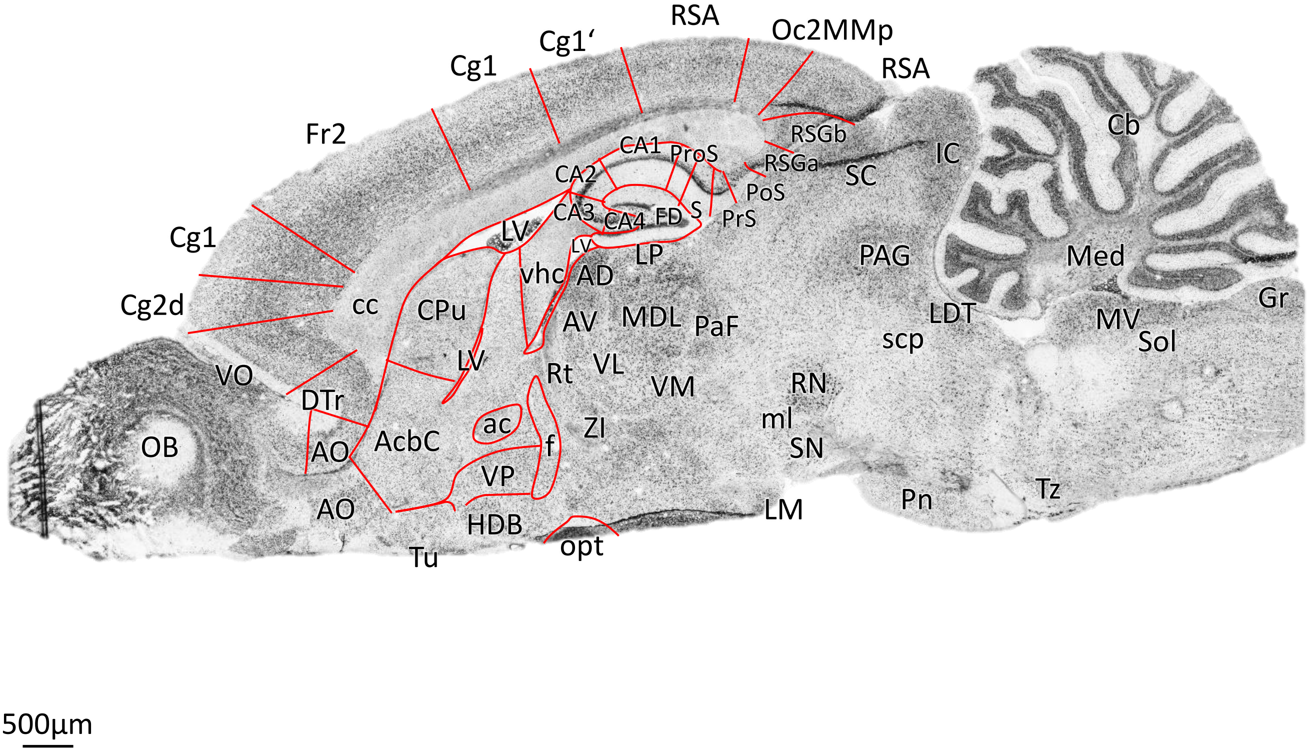

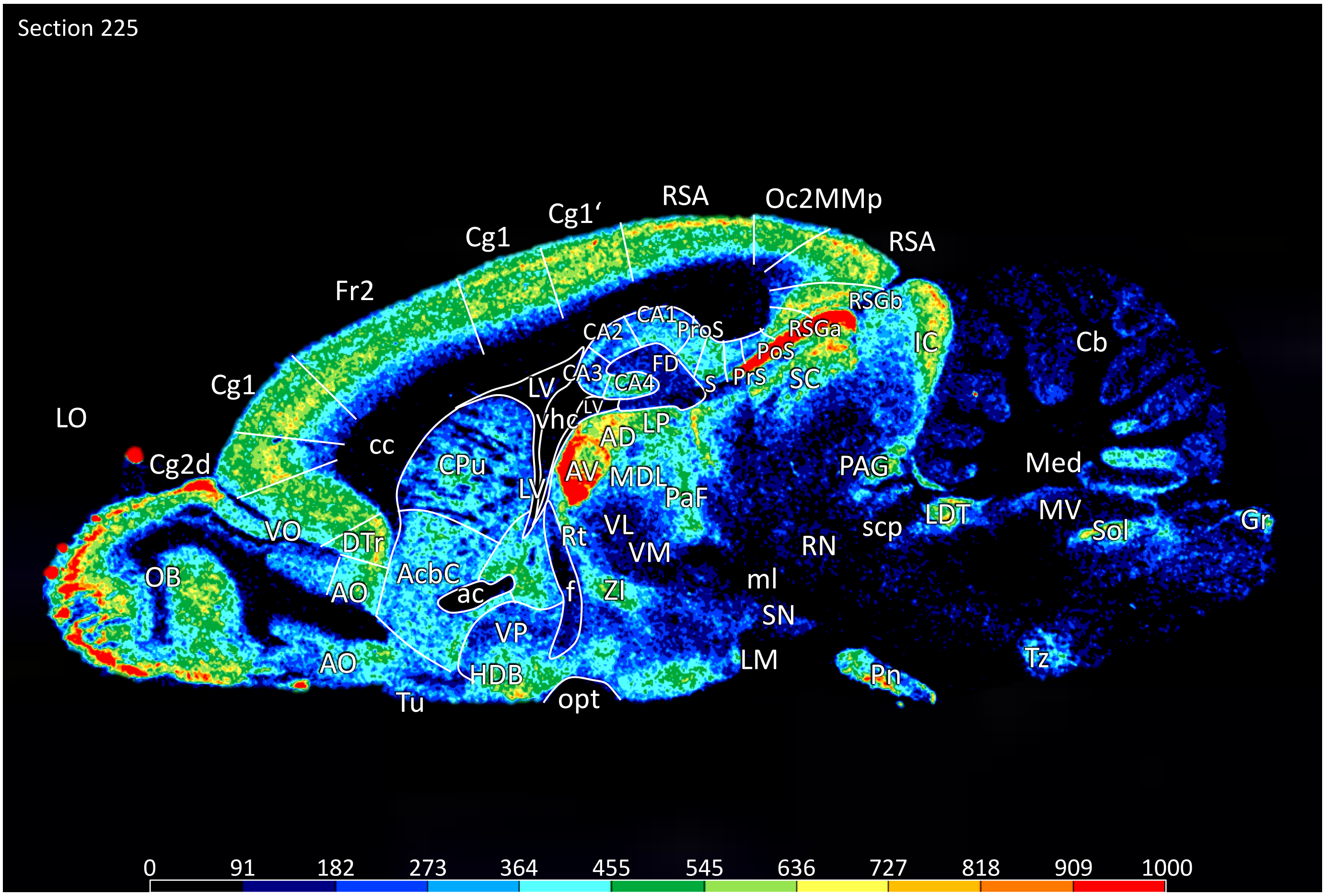

Level s5

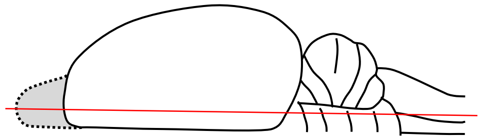

Section 261

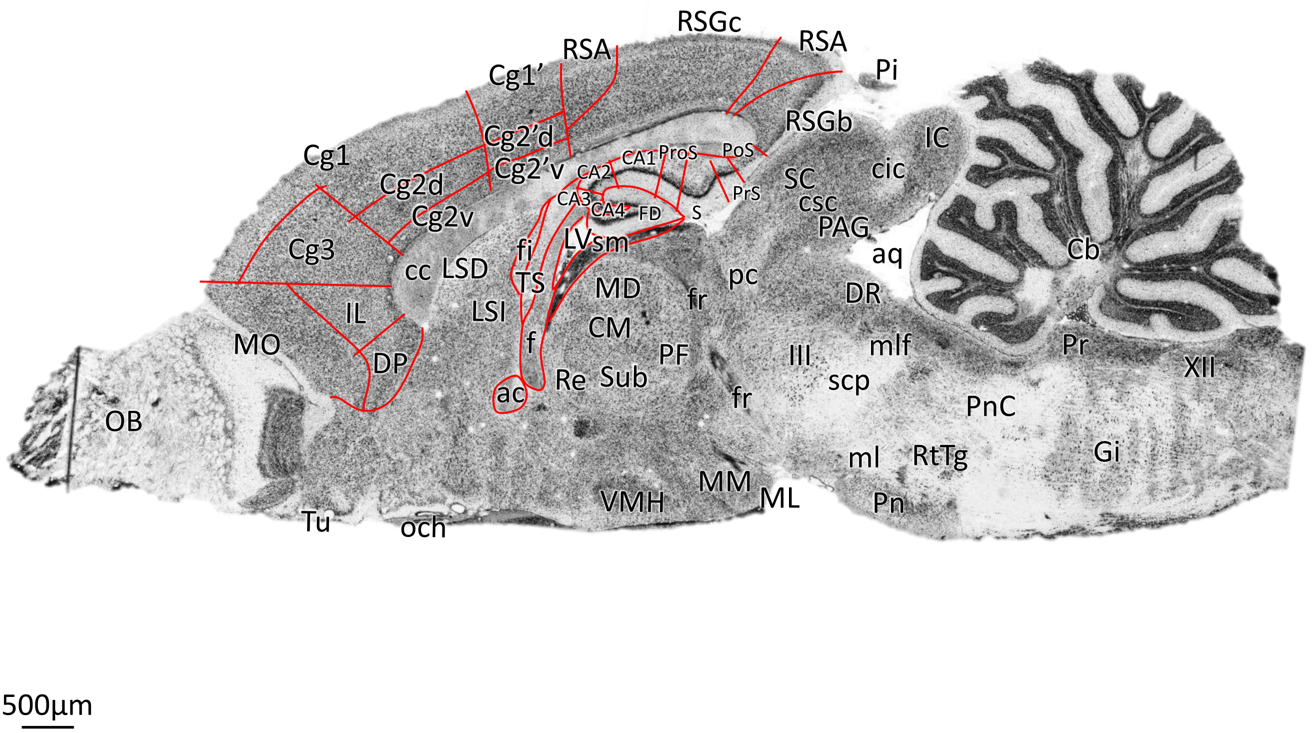

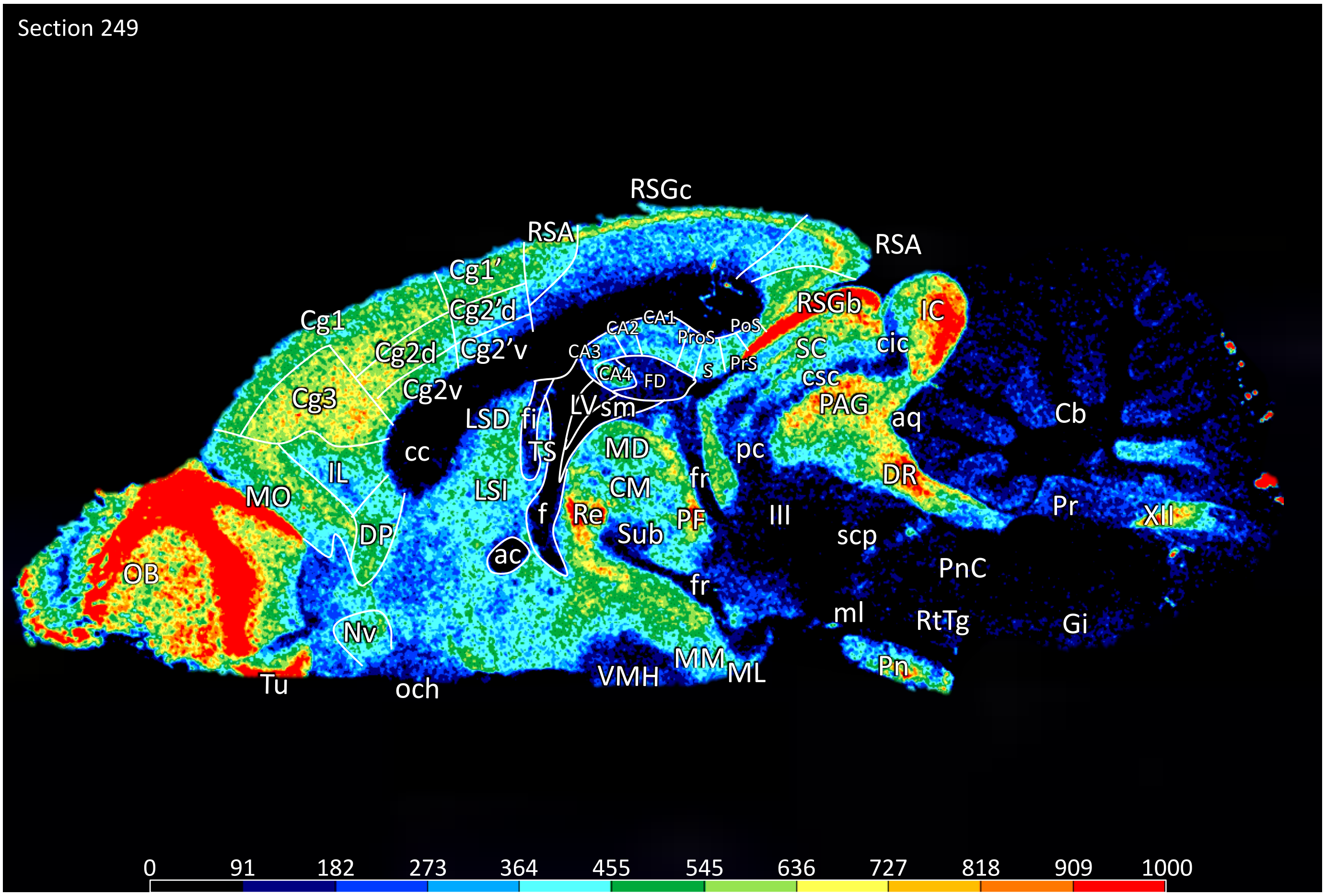

Horizontal series

Level h1

Section 45

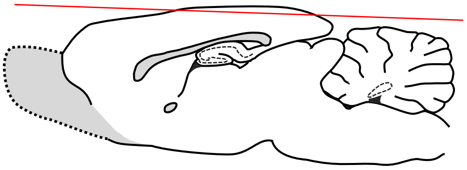

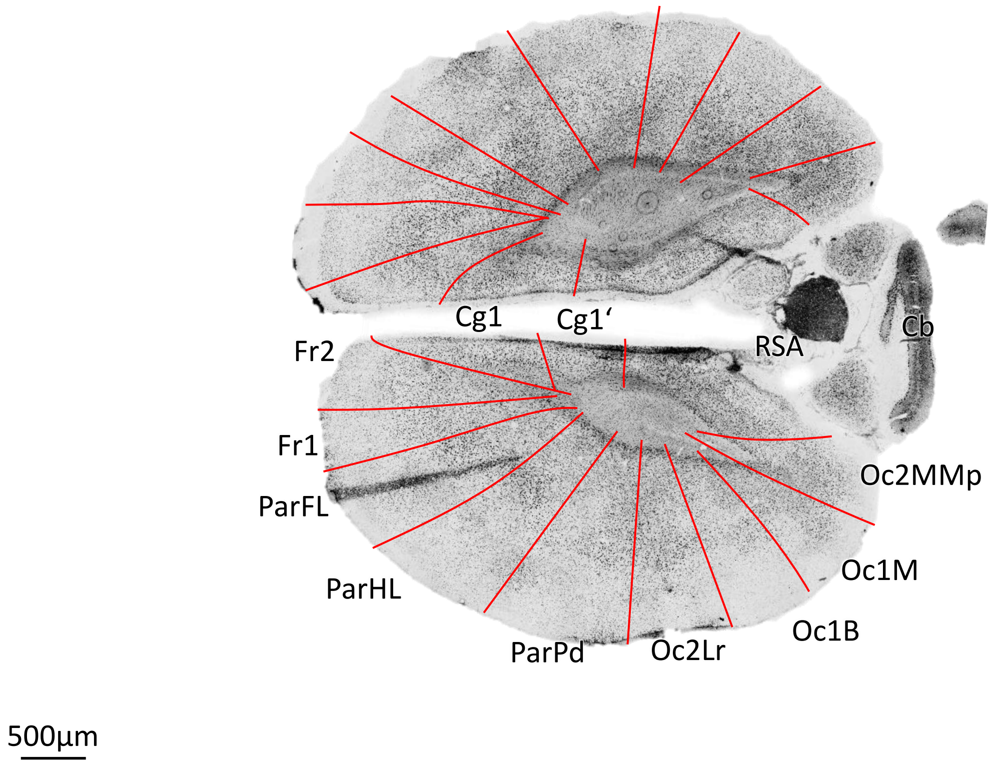

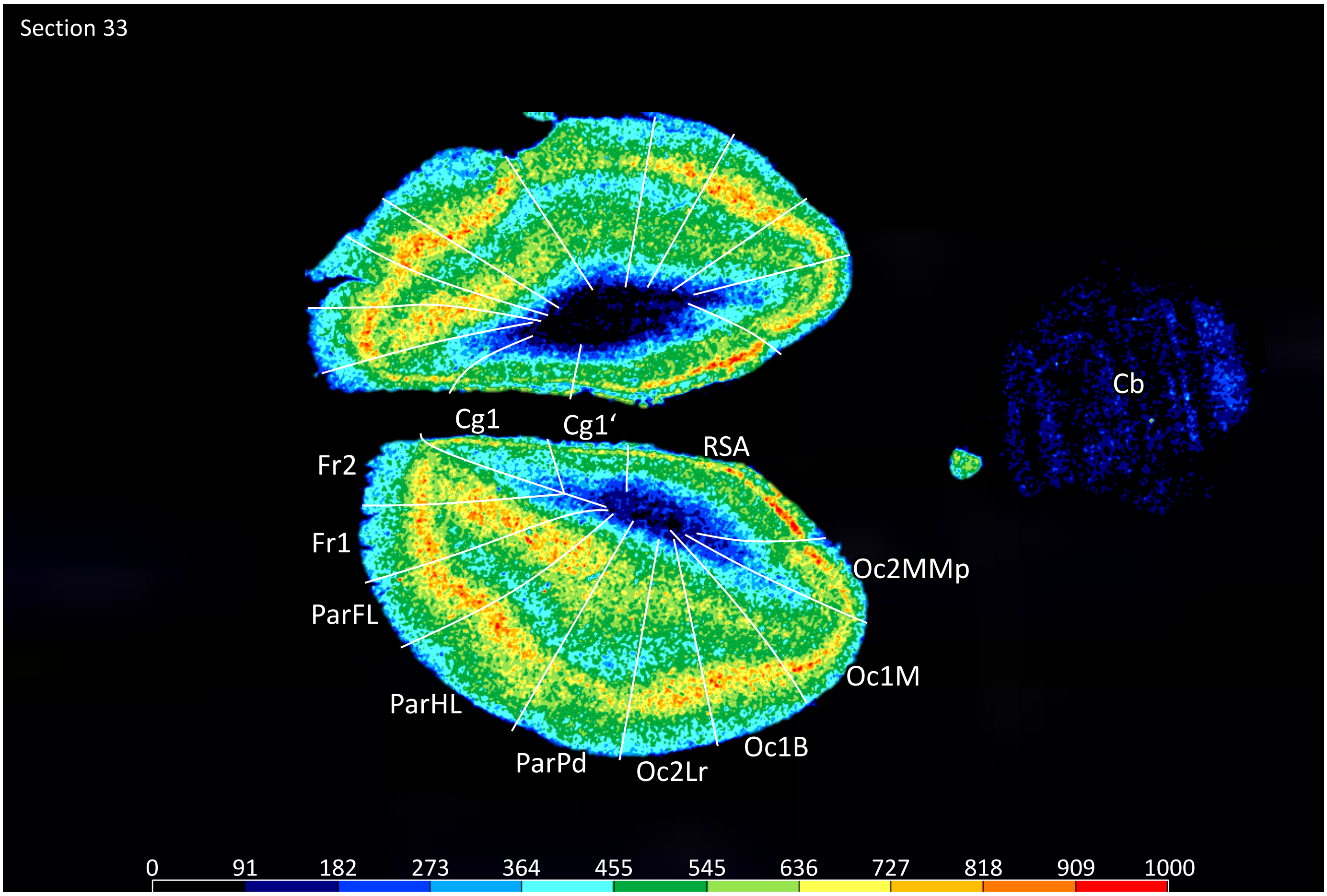

Level h2

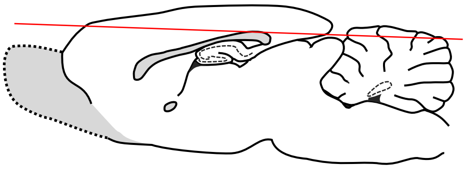

Section

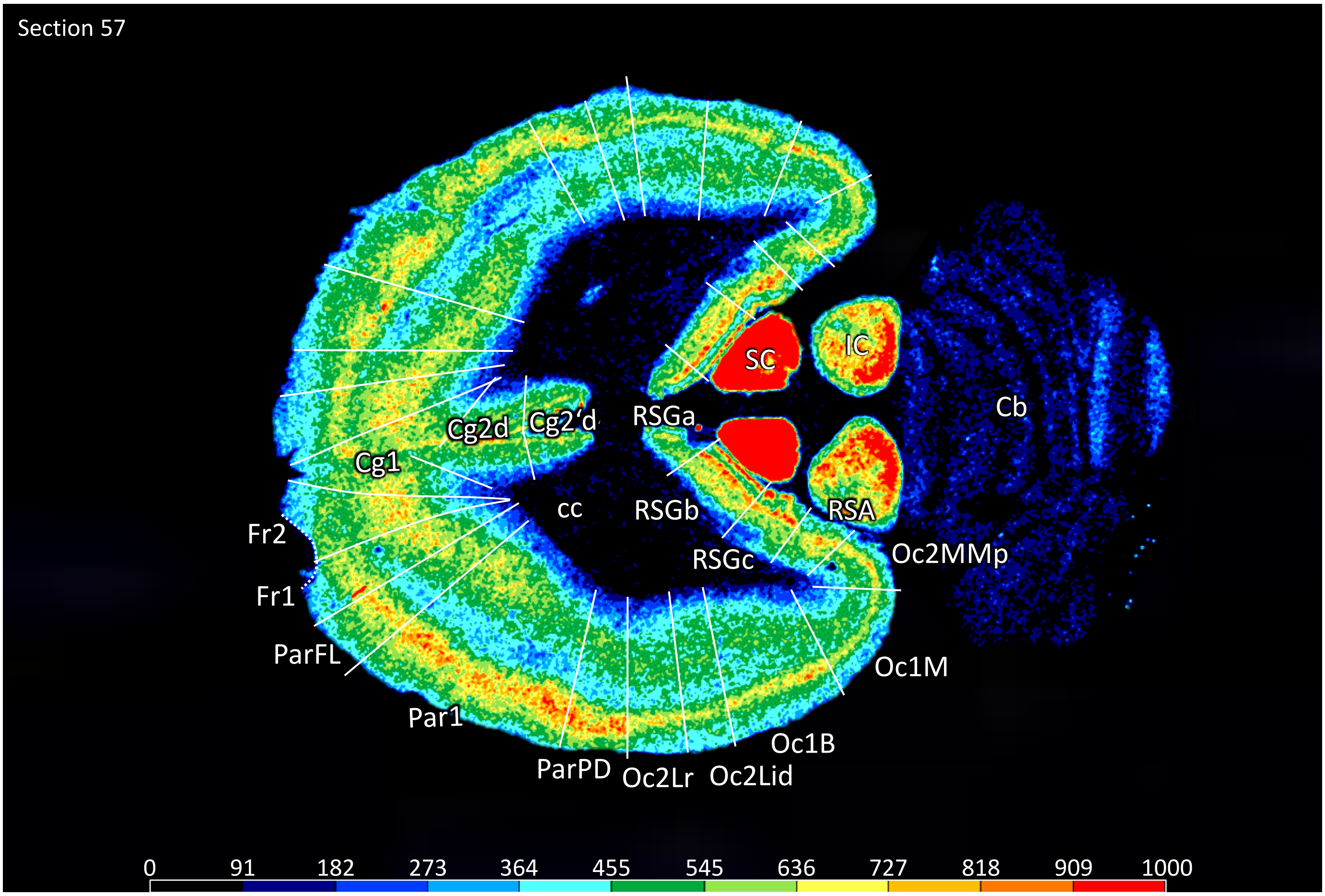

Level h3

Section 69

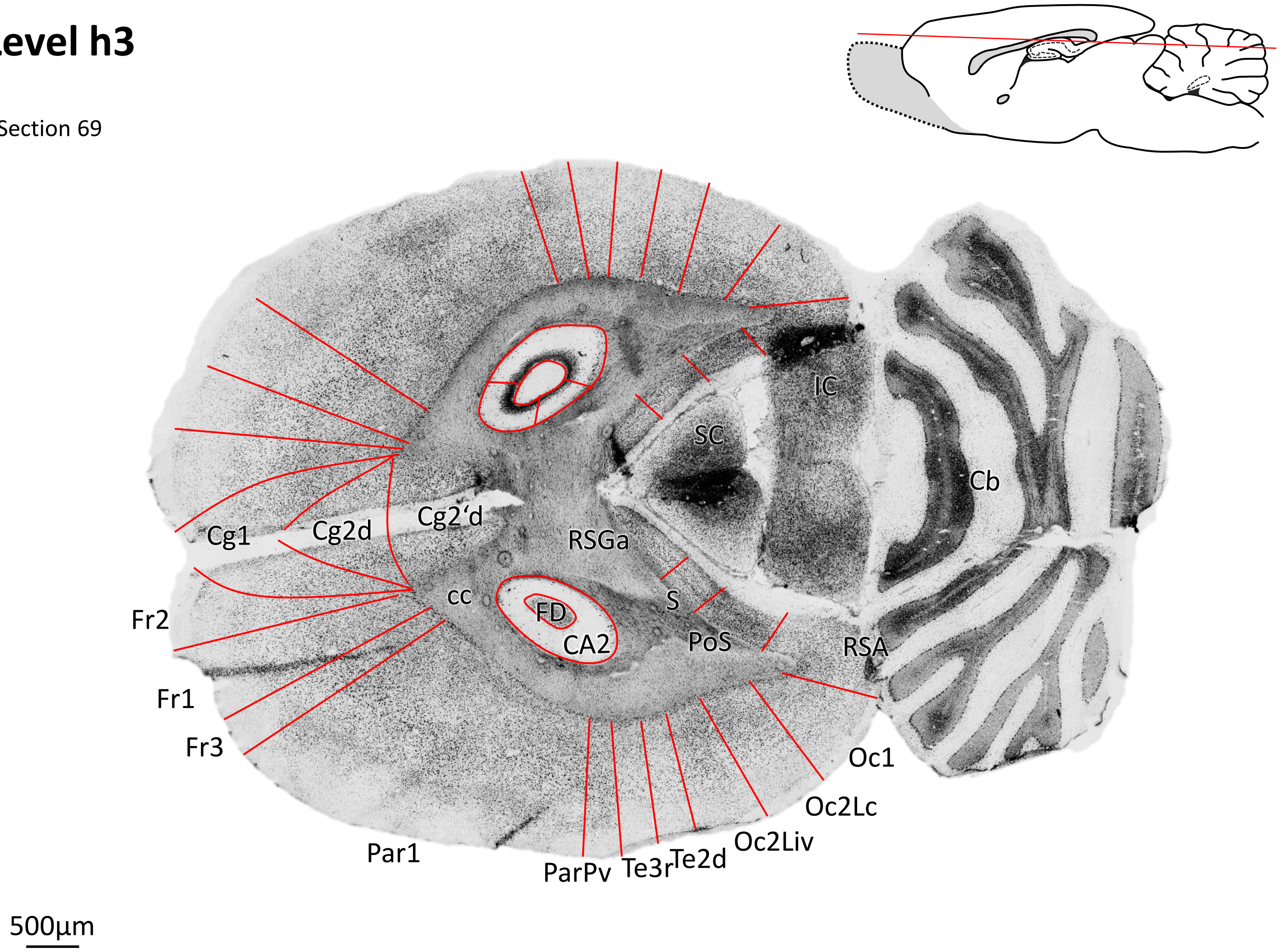

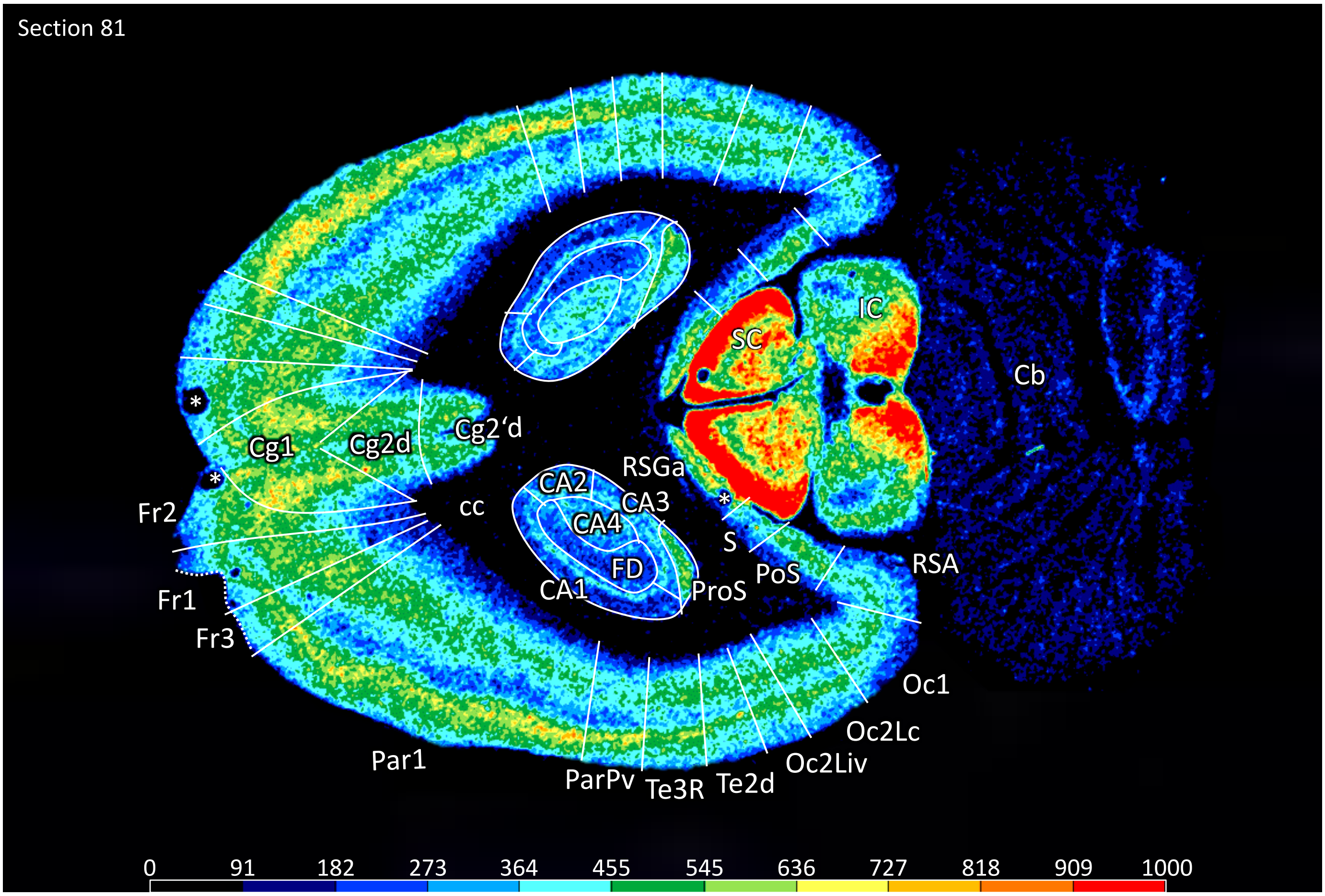

Level h4

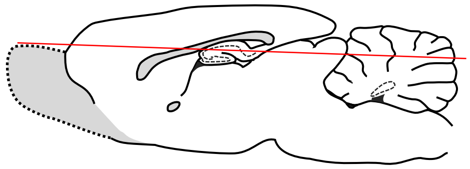

Section 117

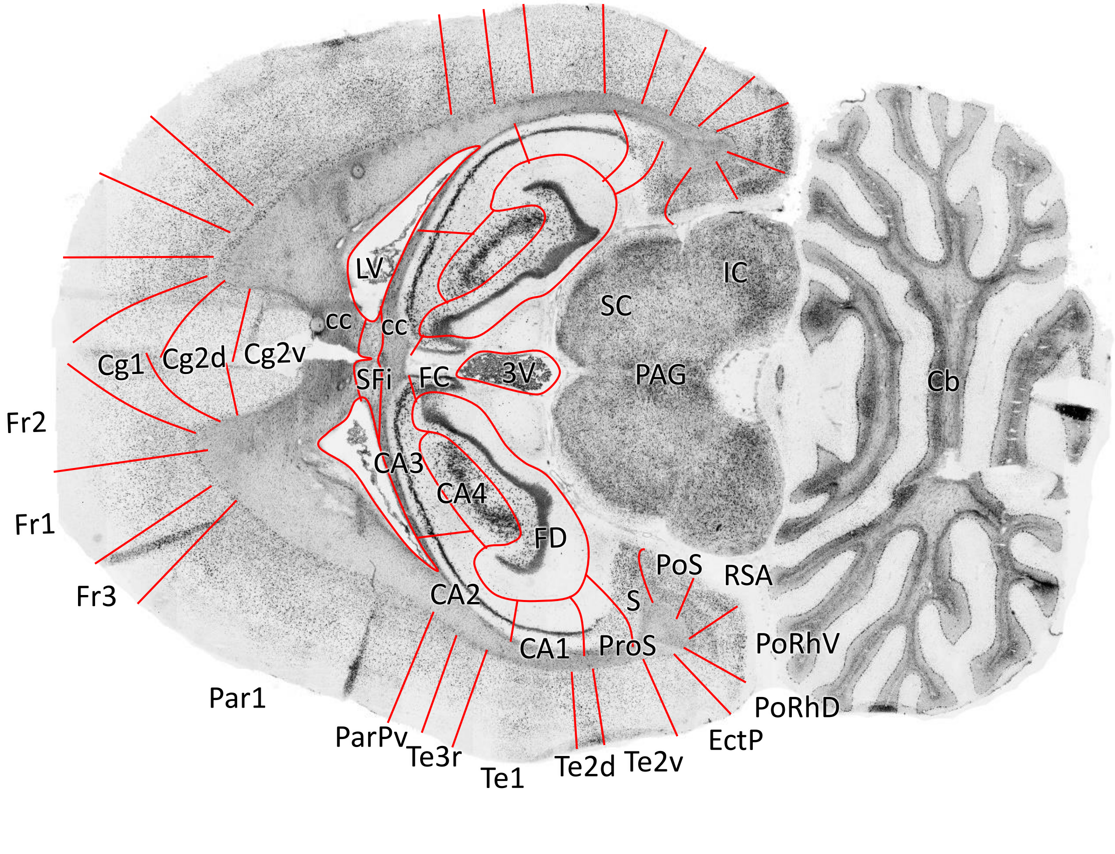

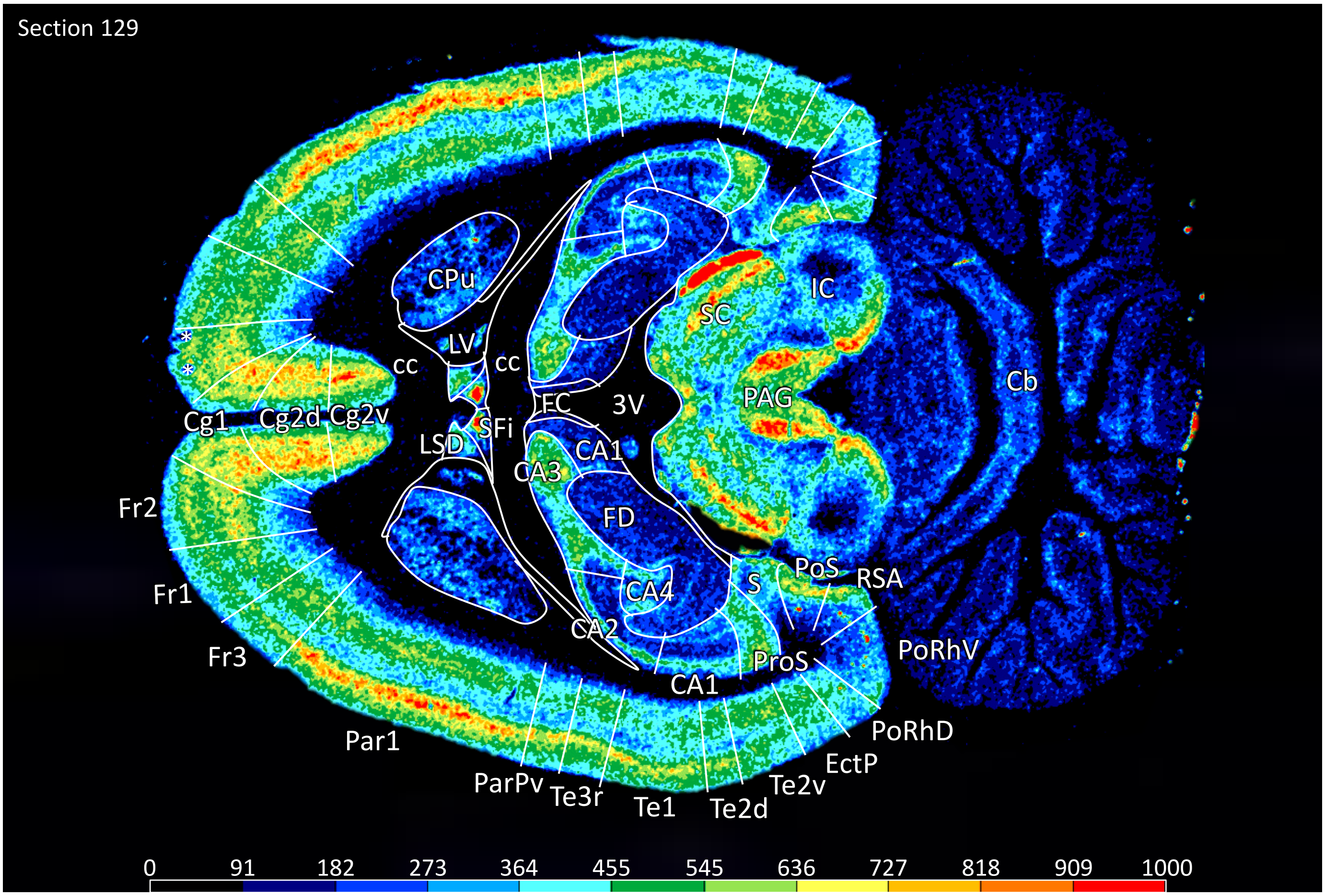

Level h5

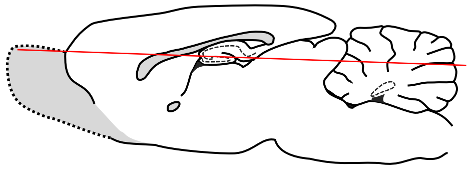

Section 165

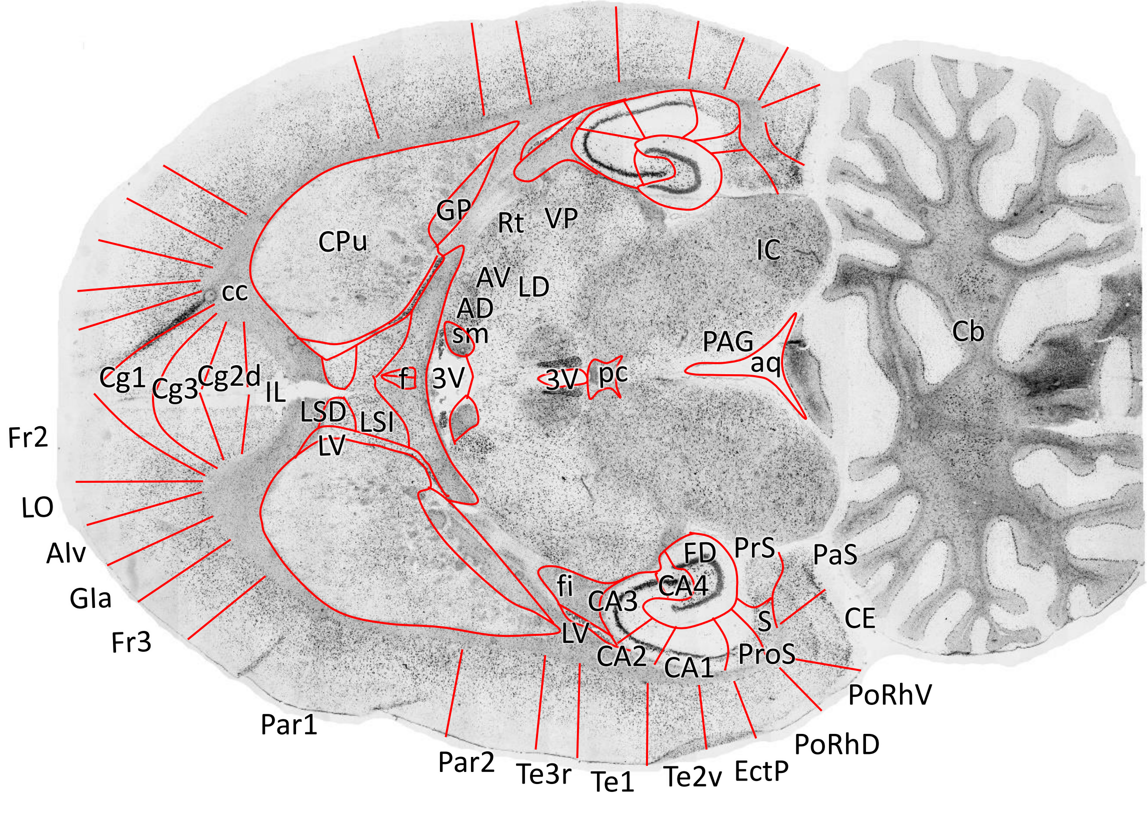

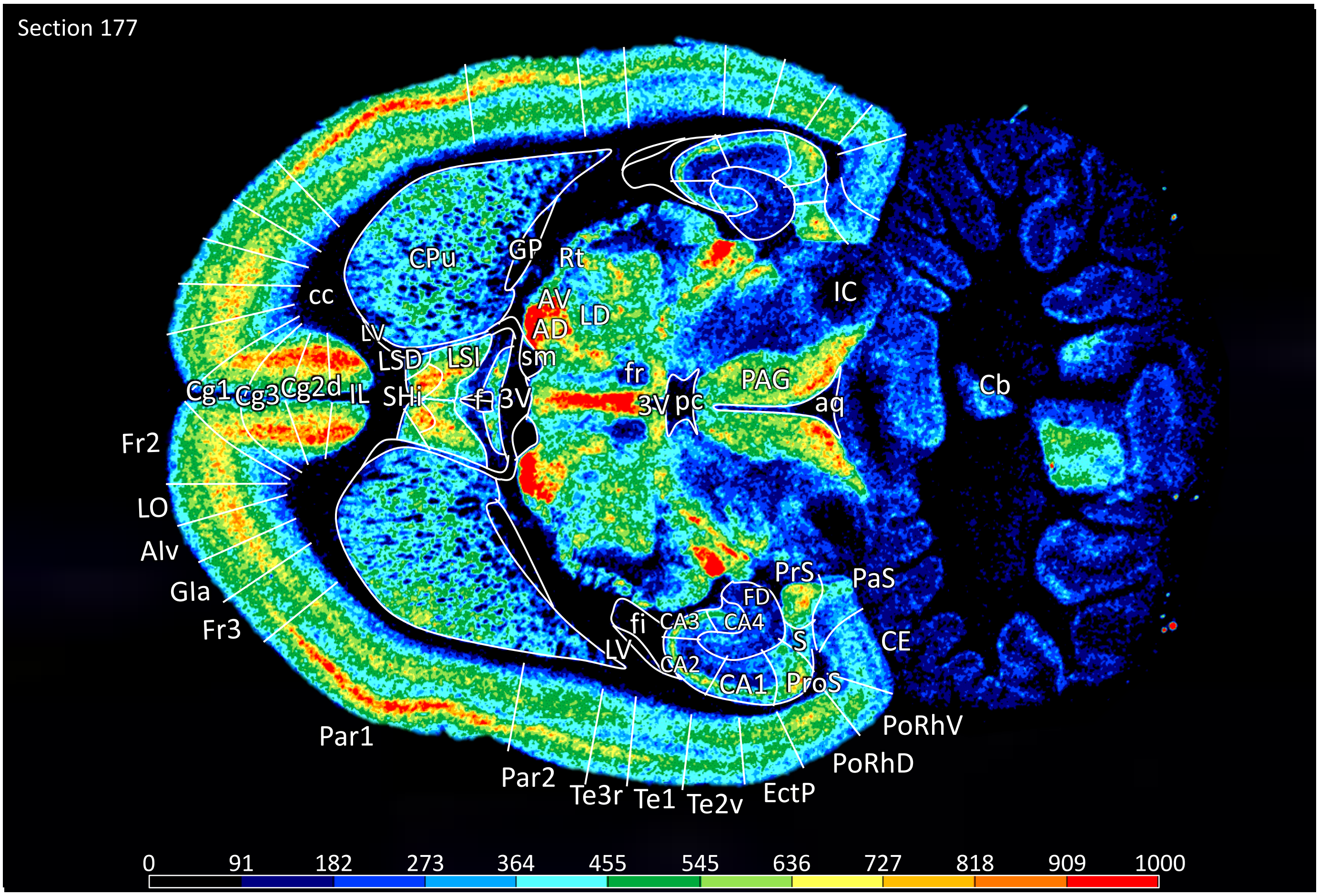

Level h6

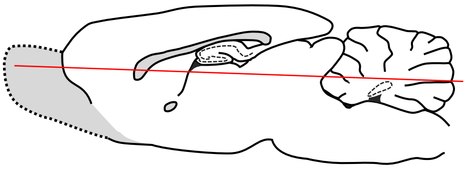

Section 213

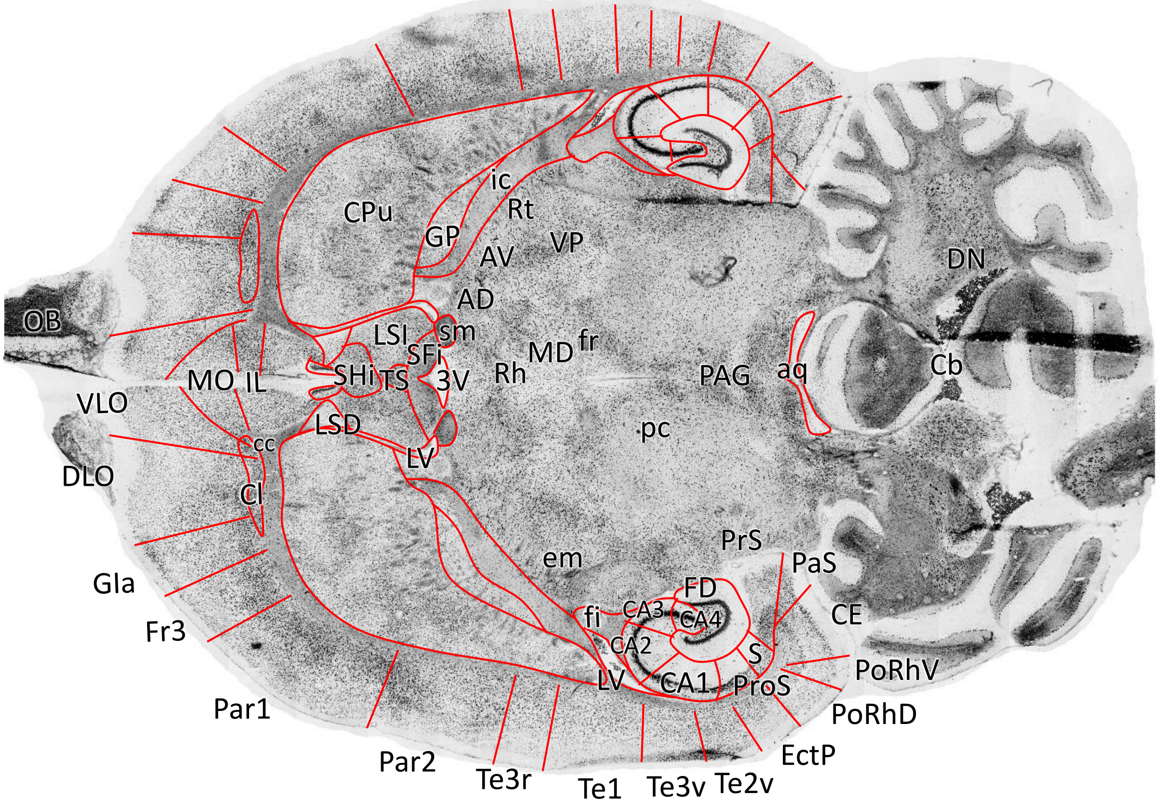

500µm

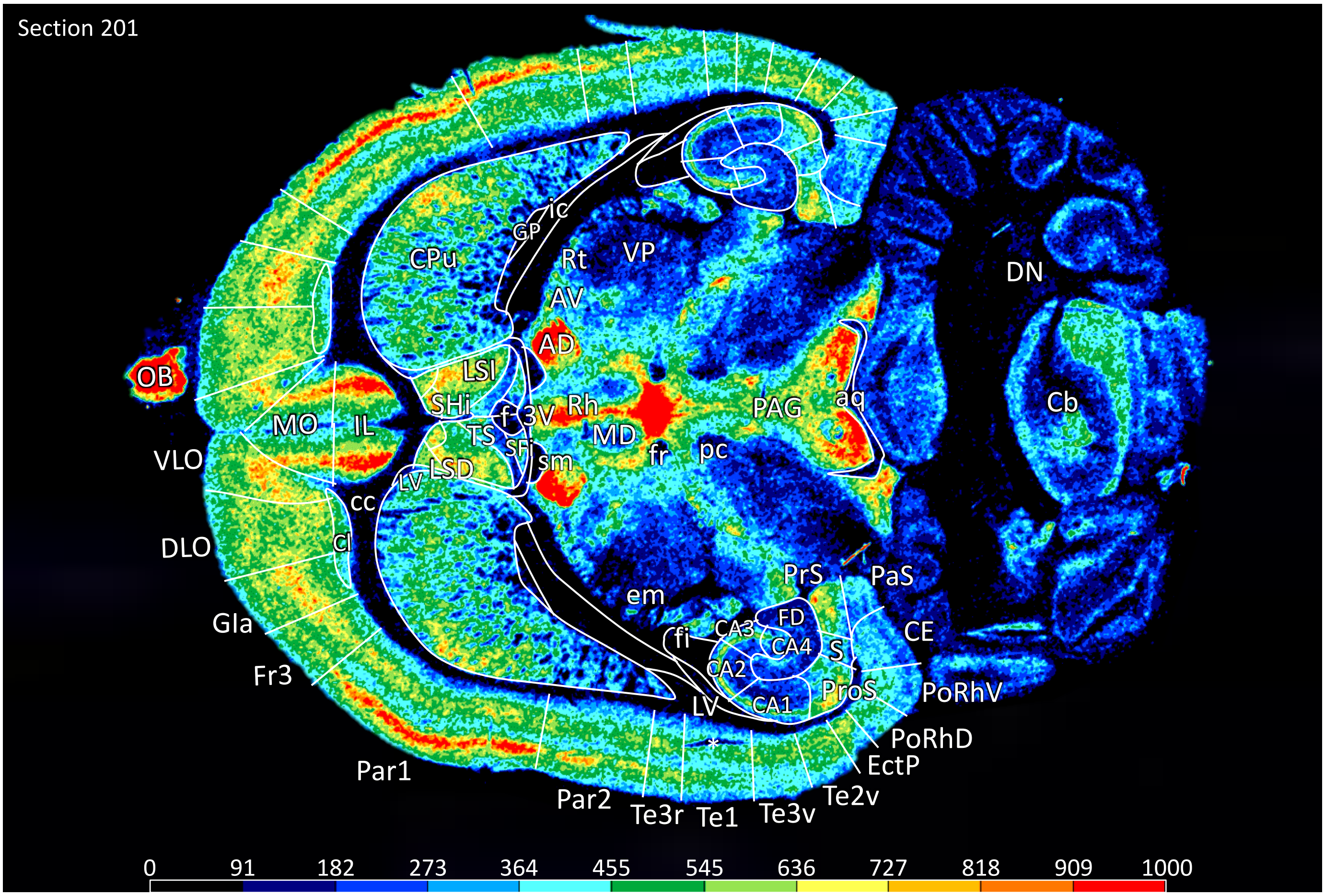

Level h7

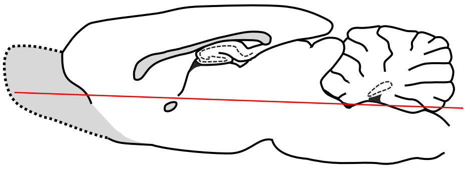

Section 237

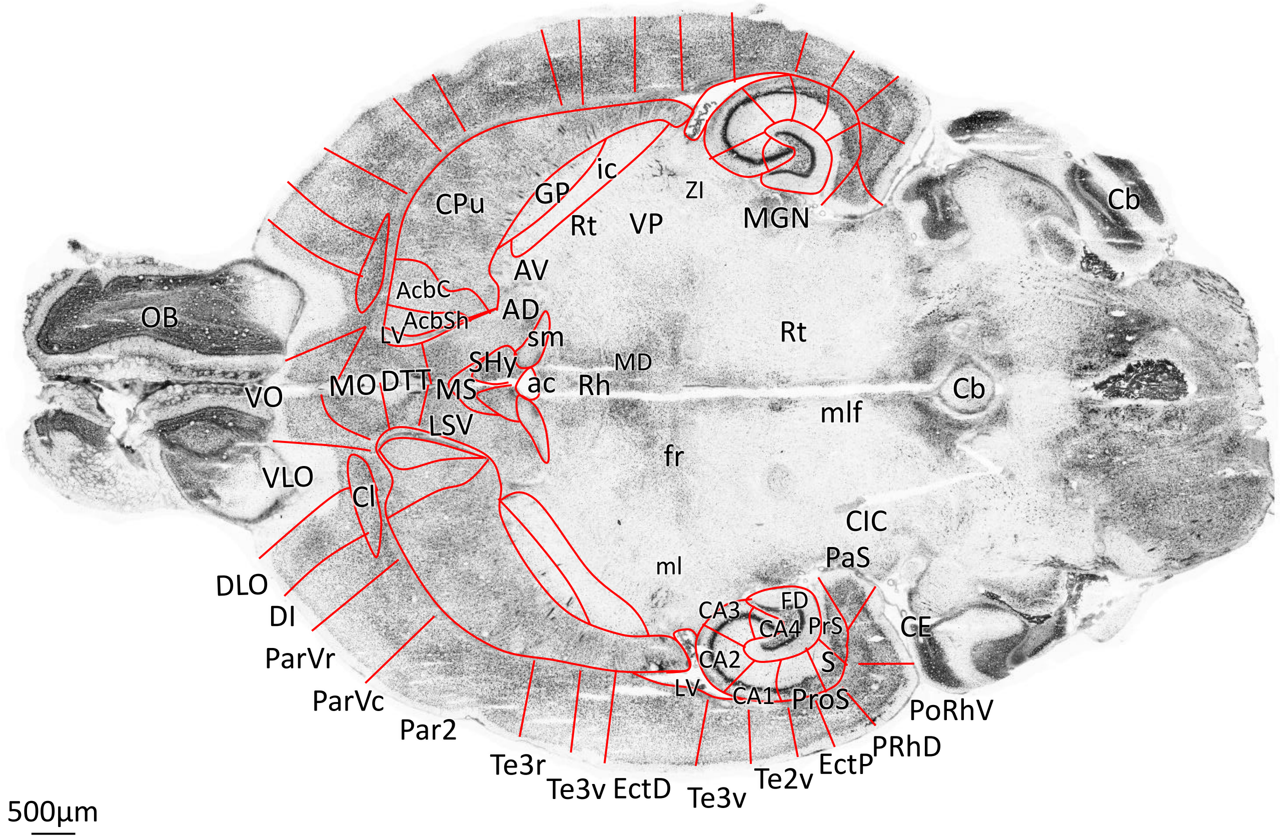

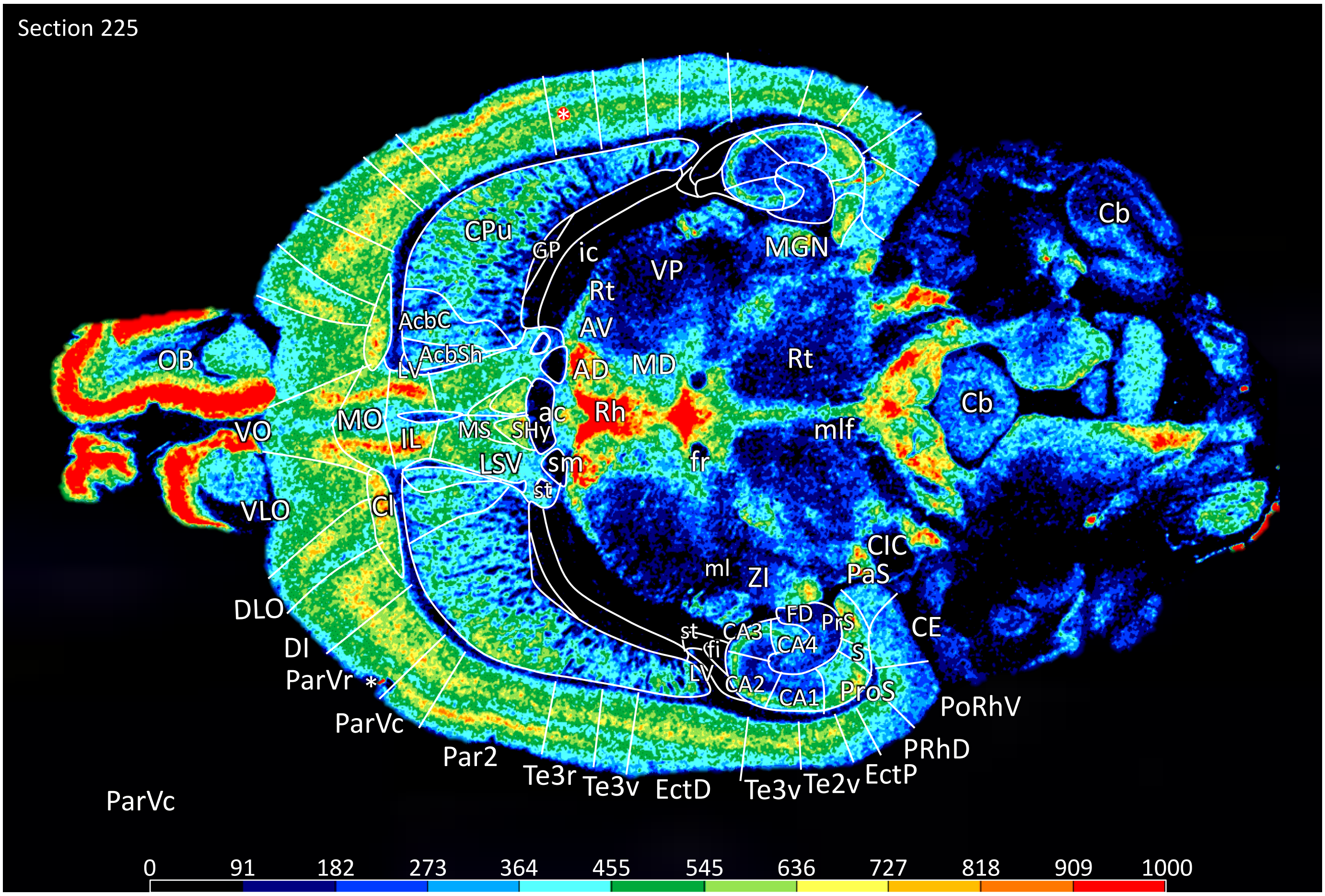

Level h8

Section 261

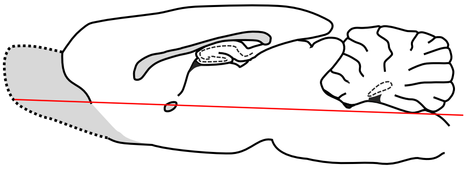

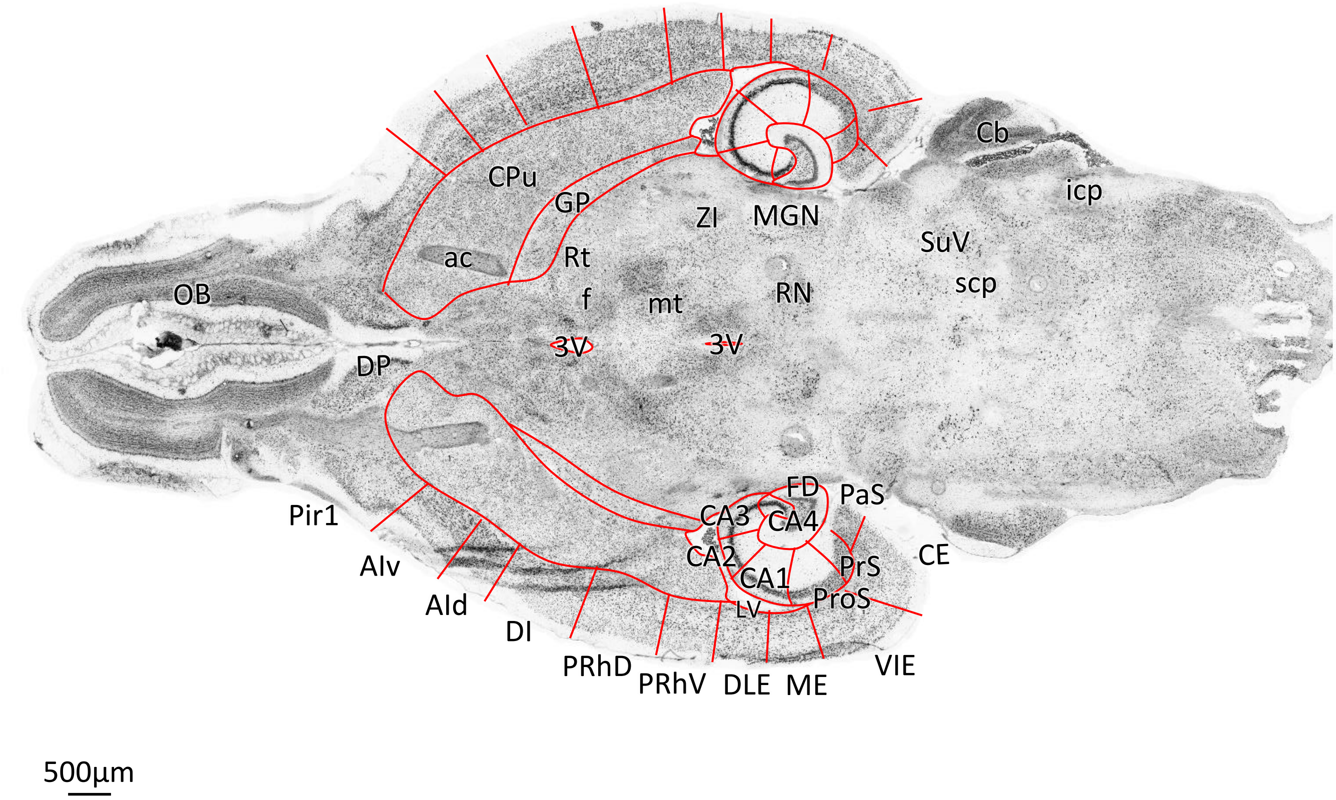

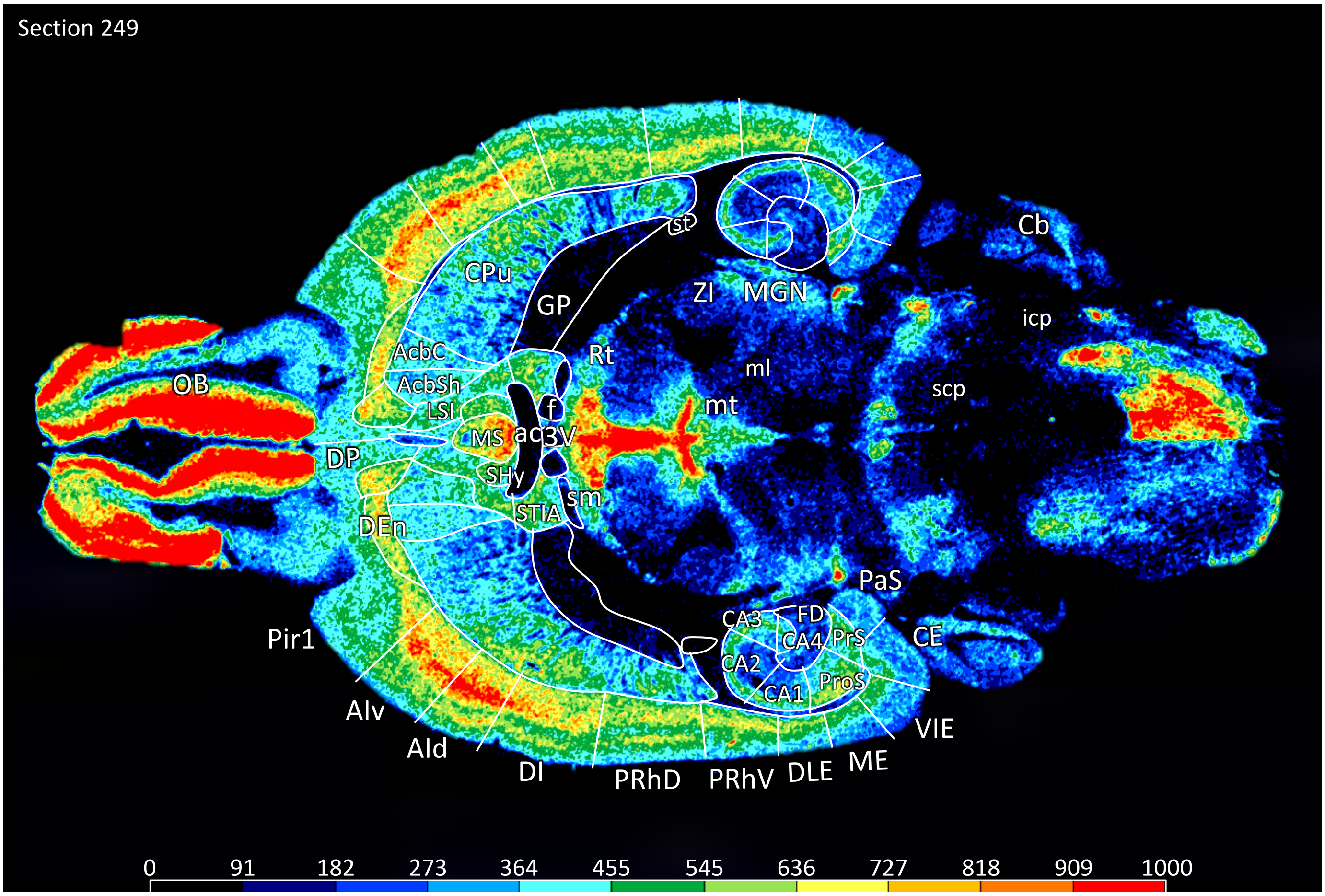

Level h9

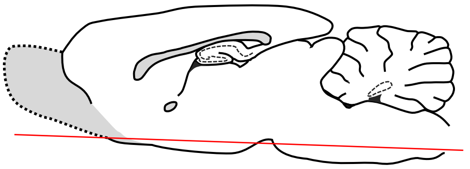

Section 285

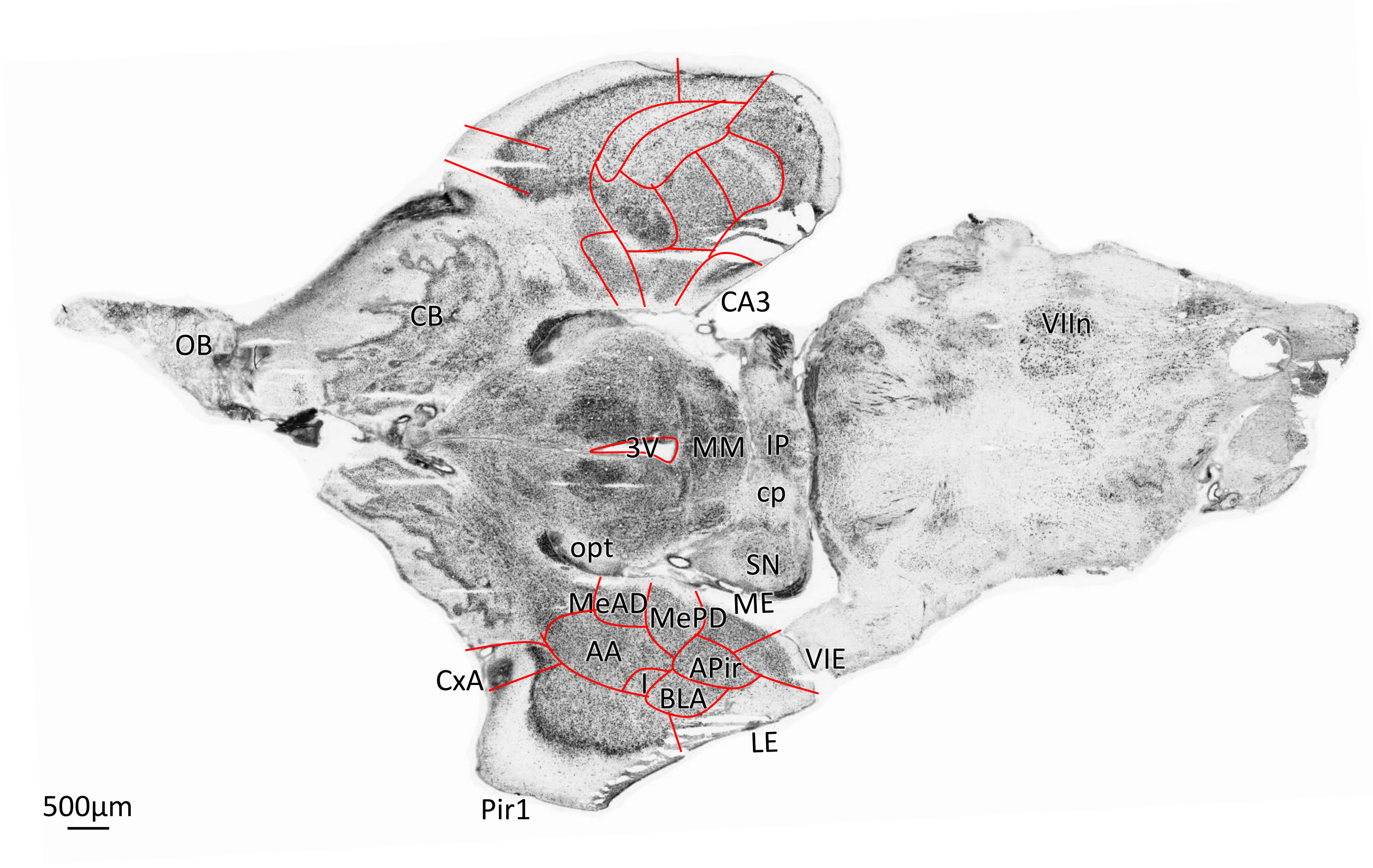

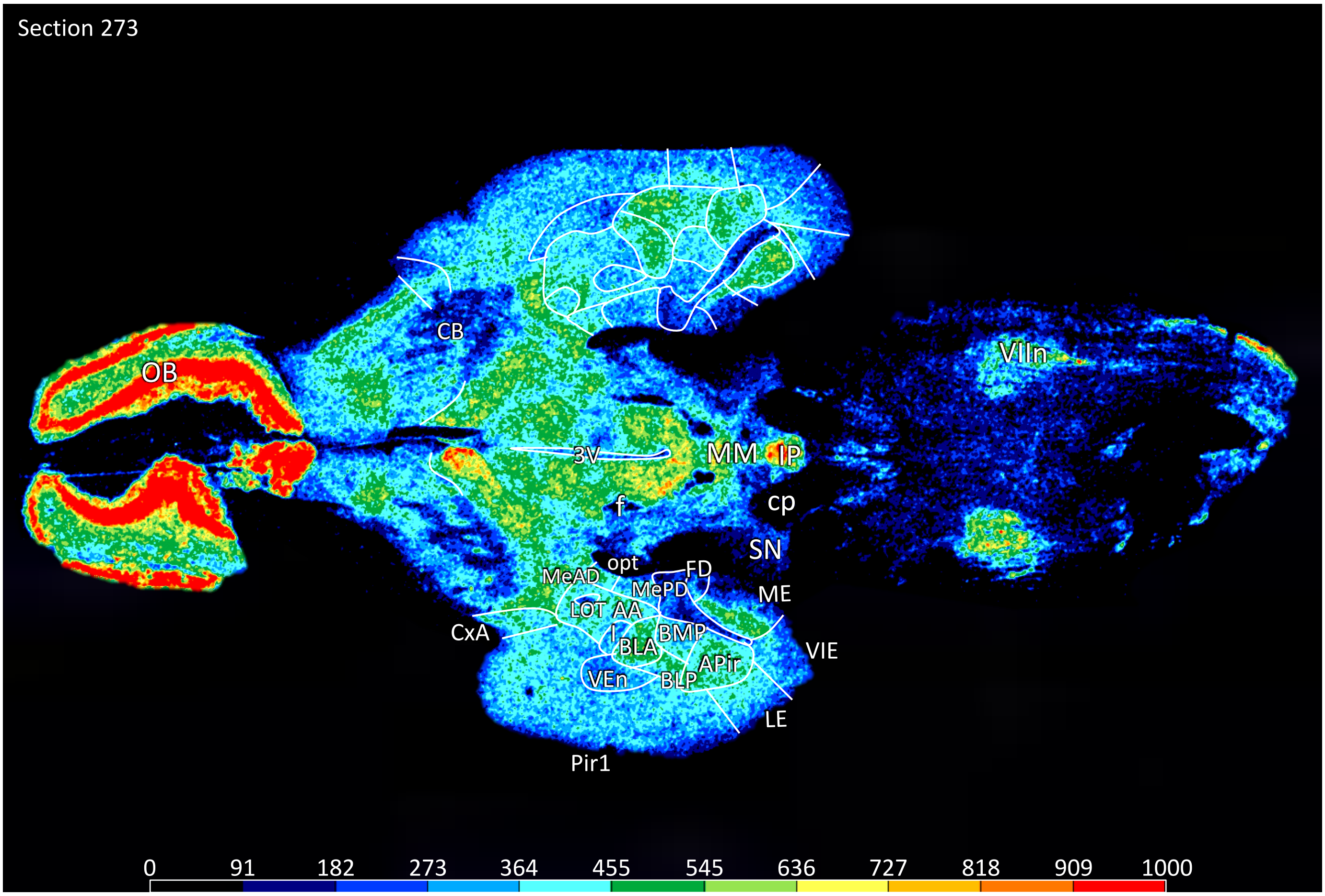

Supplement: Supplementary file 1 — Supplementary file1 (PDF 56679 kb) [file 429_2023_2654_MOESM1_ESM.pdf]
